# Automated simultaneous assignment of bond orders and formal charges

**DOI:** 10.1186/s13321-019-0340-0

**Published:** 2019-03-06

**Authors:** Ivan D. Welsh, Jane R. Allison

**Affiliations:** 1 Centre for Theoretical Chemistry and Physics, Institute of Natural and Mathematical Sciences, Massey Unversity, Private Bag 102904, Auckland, New Zealand; 2 School of Biological Sciences, University of Auckland, Private Bag 92019, Auckland, New Zealand; 3 BiomolecularInteraction Centre, University of Canterbury, Private Bag 4800, Christchurch, New Zealand; 4 Maurice Wilkins Centre for Molecular Biodiscovery, University of Auckland, Private Bag 92019, Auckland, New Zealand

**Keywords:** Bond order, Formal charge, Fixed parameter tractable, Graph theory

## Abstract

Bond orders and formal charges are fundamental chemical descriptors. In cheminformatic applications it is necessary to be able to assign these properties to a given molecular structure automatically, given minimal input information. Here we describe a method for determining the bond order and formal charge assignments from only the atom types and connectivity. Our method utilises a graph theoretical description of electron positions. Each electron position assignment is scored according to lookup tables of atomic and bond dissociation energies derived from quantum chemical calculations. We tested three different optimisation methods—local optimisation, an A* pathfinding method, and an FPT optimisation method utilising tree decompositions—for finding the best electron position assignment, from which the bond orders and formal charges are extracted. We show that our method can assign bond orders and formal charges at a high degree of accuracy across a wide range of molecules from two different databases, and that the FPT algorithm provides the best combination of speed and accuracy.

**Electronic supplementary material:**

The online version of this article (10.1186/s13321-019-0340-0) contains supplementary material, which is available to authorized users.

## Introduction

The ability to assign chemical characteristics such as bond orders and formal charges is crucial to many higher-order algorithms in computational chemistry and cheminformatics. The formal charge is the charge assigned to an atom in a molecule assuming that electrons in all chemical bonds are shared equally between atoms, and the bond order of a bond is the number of chemical bonds between a pair of atoms. Both properties can be easily deduced from the Lewis structure of a molecule, which shows how the valence electrons are arranged amongst the atoms and bonds of the molecule.

The best way to determine the Lewis structure of a molecule is to calculate the actual electronic density distribution and then use the Natural Bond Orbital method [1] to obtain bond orders and formal charges. However, this approach can be computationally expensive, and with the advent of large databases of organic molecules, such as the Protein Data Bank (PDB) and Cambridge Structural Database (CSD), the need for fast automated schemes became pertinent. As such, over the last few decades, a number of such schemes have been developed. The COBRA program of Leach et al. [2] uses a backtracking search algorithm to automatically assign bond orders. IDATM from Meng and Lewis [3] can be used to determine the connectivity and hybridisation state of atoms based on input three-dimensional (3D) coordinates. Baber and Hodgkin follow a similar scheme, but can also assign bond orders [4]. Lang et al. assign bond orders based on characteristic bond lengths, bond angles and torsion angles, [5] as do Hendlich et al., who also include small functional group identification to help avoid incorrect assignments due to erroneous or ambiguous geometrical data [6]. All of these methods, however, require accurate 3D coordinate information. The methods of Froeyen and Herdewijn [7] and Labute [8] could theoretically be used on structures with only atom type and connectivity information, but they were developed primarily for use when 3D coordinate information, albeit for only the heavy atoms, is provided.

Wang et al. developed a heuristic method to determine bond orders based on arbitrary penalty scores [9]. The biochemical algorithms library (BALL) software from Dehof et al. is an extension of this work. Dehof et al. used the same penalty scores as Wang et al., and developed three exact solvers guaranteed to find a bond order assignment with minimum total penalty score, as well as allowing enumeration of all possible bond order assignments with minimum total penalty score [10]. Both of these methods only require element type and atom connectivity information when all hydrogen atoms are included. As formal charges and bond orders are somewhat co-dependent, the absence of formal charges in the input molecule could result in incorrect atom types being perceived, leading to incorrect bond order assignments. Theoretically, formal charges can be back calculated from bond order assignments (if they are correct), however there can be situations where ambiguous formal charge assignments are possible, and there is no guarantee that the calculated formal charges will match the required total molecular charge.

As part of a broader molecular dynamics automated parameterisation scheme, we have developed a new method for the simultaneous assignment of formal charges and bond orders. In order to allow both of these properties to be assigned in situations where the available coordinates are not energetically favourable, our method requires only chemically plausible atomic coordinates, along with element type, atom connectivity, and the total charge of the molecule. The combination of atom connectivity and total molecular charge fixes the protonation state of the molecule. We take advantage of the fact that in essence, formal charges and bond orders are descriptions of the positions of valence electrons within a molecule. Minimising some function of the electron positions thus results in an optimal formal charge and bond order assignment. Given that electron positions are involved, the obvious choice of function is one derived from high-level quantum chemical calculations.

We describe here the function that is minimised and three different optimisation methods: local optimisation, the A* pathfinding method, and an FPT optimisation method utilising tree decompositions. We first check the self-consistency of the algorithms, then compare the performance of the A* method in our software and in the BALL software of Dehof et al., and lastly compare to the bond order and formal charge assignments from two molecular databases in terms of both the speed and the accuracy with which these reference data are reproduced, as well as the level of theory required for the quantum chemical calculations such that the scoring function identifies the correct assignments. We find that the FPT algorithm provides the best balance between efficiency and accuracy. Using FPT, our method attains similar accuracy to that of Dehof et al., but without the need to provide the formal charge.

The code described here is available on Github at https://github.com/allison-group/indigo-bondorder. It is written in C++, utilising the Boost graph library, [11] and requires a C++-14 compliant compiler. For ease of use, Python bindings are provided for using version 2.2.2 of the pybind11 library [12].

## Methods

Our bond order and formal charge assignment scheme determines an optimal assignment of electron positions for a molecule by minimising a score that is a function of the electron assignment. We first describe how the chemical features of the molecule are represented and the initialisation of the electron position assignments. We then outline the electron assignment scoring function, which depends upon scores associated with the formal charge state of each atom and the order of each bond. Lastly, we describe the three different methods used to optimise the electron position assignments, giving rise to the formal charge and bond order assignments for the molecule.

### Initialisation

A query molecule can be submitted in any of the standard chemical formats able to be parsed by Open Babel, so long as the number and type of each atom, including hydrogen atoms, their connectivity, and the total molecular charge are provided explicitly. Some file formats allow for implicit hydrogen atoms through the use of atom typing. This information is encoded internally as a molecular graph, so that a graph theoretic approach can be used to optimise the electron position assignment.

The total number of electrons whose position must be optimised is calculated from the number of valence electrons according to

1 $$\begin{aligned} e_T = -q_T - 2N_B + \sum _{i = 1}^{N_A} \nu _i, \end{aligned}$$

where $e_T$ is the total number of electrons to place, $q_T$ is the total molecular charge, $N_A$ and $N_B$ are the numbers of atoms and bonds in the molecule, respectively, and $\nu _i$ is number of valence electrons of atom *i*, which is known from its elemental type. The $-2N_B$ component accounts for each bond in the molecule requiring two electrons in order to have a bond order of at least one.

The positions that electrons can occupy in a molecule, encoded as a multiset of graph vertices and edges, ${\mathcal{P}}$, are determined as follows. Each element has a target valency, set to eight for all elements other than hydrogen, for which it is two, and phosphorous and sulfur atoms involved in at least three bonds, for which it is set to ten and twelve respectively. In this way, we allow for hypervalent representations of functional groups such as phosphates and sulfates, while representing all other groups, such as nitros, in charge separated form. The multiplicity of a given vertex *v* in ${\mathcal{P}}$ is given by $v \in ^{m_v} {\mathcal{P}}$ where

2 $$\begin{aligned} m_v = \tau _v - 2\delta (v) \end{aligned}$$

with $\tau _v$ being the target valency of the element associated with *v* and $\delta (v)$ the degree of *v*. The multiplicity of a given edge $e = (u,v)$ in ${\mathcal{P}}$ is given by $e \in ^{m_e} {\mathcal{P}}$ where

3 $$\begin{aligned} m_e = \min \left( \tau _u - 2\delta (u), \tau _v - 2\delta (v)\right) . \end{aligned}$$

### Electron assignment scoring

Let $G=(V,E)$ be a molecular graph. An *electron assignment* is a map $c_p:V\cup E \rightarrow {\mathbb Z}_{\ge 0}$ where $c_p[x]$ is the total number of electrons placed on member $x \in V \cup E$. The *score* of the electron assignment is then given as the sum of scores for each vertex and edge.

4 $$\begin{aligned} S = \sum _{x \in V\cup E} \Gamma [k_x] \end{aligned}$$

These scores are stored in a lookup table, $\Gamma$, using bit shifting to generate a unique 32-bit unsigned integer key, $k_x$. If a given key is not found in the lookup table, a default score of $\infty$ is given.

For a vertex *v*, the lookup table key depends on the element of the corresponding atom and its formal charge, calculated as

5 $$\begin{aligned} F(v) = \nu _v - c_p[v] - \sum _{u \in N(v)} \frac{c_p[(u,v)]}{2} \end{aligned}$$

where *N*(*v*) is the set of neighbouring vertices of *v*. The first seven bits of the key are set as per the binary value of the atomic number. The next four bits are set to the binary value of the magnitude of the calculated formal charge. Finally, the twelfth bit is set if the formal charge is negative, and left unset if it is positive. If the valence state of an atom exceeds its target valence, the score of that atom is set to $\infty$, and no key is calculated.

For an edge *e*, the lookup table key depends on the elements of the two vertices the edge is between, and the number of electrons assigned to the edge, $c_p[e]$. In the same manner as the vertex key, the first seven bits of the key are set to the binary value of the atomic number of one of the vertices. The next seven are set to the binary value of the other vertex. Finally, bits fifteen through eighteen inclusive are set as per the binary value of the number of electrons assigned to the edge. As each edge will always have at least two electrons assigned, there is no overlap between the set of possible keys for the vertices and edges.

These simple methods for key determination were chosen as the corresponding scores can be easily determined from quantum chemical calculations. We note that there is sufficient flexibility in using 32-bit unsigned integer keys for more complex key generation methods to be used, incorporating optimised key–score pairs.

At present, scores have only been calculated for the elements—hydrogen, carbon, nitrogen, oxygen, fluorine, phosphorus, sulfur, chlorine, and bromine—and bonds—single, double, and triple—that most commonly occur in bio-organic molecules, but as these scores are derived from quantum chemical calculations, they can easily be supplemented as required.

#### Formal charge score

In a crude sense, atoms with formal charges can be described as ions with a charge equal to the formal charge. Therefore, formal charge scores were determined from quantum chemical calculations of atomic/ionic energies. For each element, scores for all possible formal charge states were calculated. For example, carbon can have formal charge states ranging from $+\,4$ (all valence electrons removed) to $-\,4$ (electrons added until valence shell is an octet), thus we consider $\hbox {C}^{4+}$, $\hbox {C}^{3+}$, $\hbox {C}^{2+}$, $\hbox {C}^+$, $\hbox {C}^0$, $\hbox {C}^-$, $\hbox {C}^{2-}$, $\hbox {C}^{3-}$ and $\hbox {C}^{4-}$. While in normal molecules, it is highly unlikely that the majority of these formal charges are viable, they are included for completeness and to help guide the optimisation methods away from unrealistic electron assignments.

The atomic energy depends on the spin state of the atom or ion. Looking at carbon again, there are four valence electrons in an electron configuration of $1\hbox {s}^{2}2\hbox {s}^{2}2\hbox {p}^{2}$. The lowest energy spin state is the triplet state, with the two electrons in the 2p shell unpaired in degenerate orbitals. Both singlet and quintet states are conceivable, but they are higher energy states. We therefore consider only the lowest energy spin state for a given formal charge of each element. The scores were then determined as being the difference between the lowest energy spin state, and a reference energy of the neutral atom in either the singlet or doublet state, depending on its number of electrons. All calculations of atomic/ionic energies were calculated with the CR-CCL method, [13, 14] utilising the def2-SVPD and def2-TZVPPD basis sets [15]. Calculations were performed using the GAMESS-US version 18AUG 2016(R1) software [16, 17]. The scores are given in Additional file 1: Tables S1 and S2.

#### Bond order score

Bond dissociation energies are a natural basis for the bond order scores. The bond dissociation energy is defined as the change in enthalpy when a bond is homolytically cleaved. For example, the bond dissociation energy of the $\hbox {C}{-}\hbox {O}$ bond in methanol is given by the enthalpy change associated with the reaction

$$\begin{aligned} \hbox {H}_{3}\hbox {C}{-}\hbox {OH}{\mathop{\longrightarrow}\limits^{[\Delta {}H]}} \hbox {H}_{3}\hbox {C}^{.} + \hbox {HO}^{.}. \end{aligned}$$

The bond dissociation energy is computed by calculating the energy difference between a molecule containing the bond of interest and the two fragments produced by homolytic cleavage of the bond. We create the simplest possible molecule containing each bond type as determined by a stick drawing. This is just the two atoms involved, with hydrogen atoms added to fill the valence positions of non-hydrogen atoms. For example, to determine the score to use for a $\hbox {C}{\equiv }\hbox {C}$ bond, the bond dissociation energy of ethyne is calculated. The score for each bond is determined as being the difference between the bond dissociation energy and the bond dissociation energy of the highest order bond considered between the two elements involved.

The structure of each molecule and the fragments produced by homolytic cleavage are geometry-optimised at the MP2 [18] level of theory, followed by a single point energy calculation with the CR-CCL method, [13, 14] utilising the def2-SVPD and def2-TZVPPD basis sets [15]. Where appropriate, calculations were performed both with and without accounting for the basis set superposition error [19]. Calculations were performed using the GAMESS-US version 18AUG 2016(R1) software [16, 17]. The scores are given in Additional file 1: Tables S3–S6.

### Formal charge and bond order determination

Once an optimised electron assignment has been calculated, determining the formal charge on each of the atoms and the bond order of all of the bonds is simple. For each atom, its formal charge is calculated as shown in Eq. (5). For each edge, *e*, the bond order is calculated as

6 $$\begin{aligned} O(e) = \frac{c_p[e]}{2}. \end{aligned}$$

### Optimisation methods

Finding the formal charge and bond order of a molecule requires minimising the value of *S* given in Eq. (4). Three different optimisation techniques for finding the lowest scoring electron assignment were tested: a steepest descent local optimisation method (“Local optimisation” section), an A* pathfinding based method (“A*” section) and an FPT optimisation method utilising tree decompositions (“Fixed parameter tractable (FPT)” section).

#### Local optimisation

Local optimisation acts similarly to a steepest decent gradient optimisation method. It is a greedy method that searches for an optimal electron assignment by finding the lowest scoring neighbour of a given electron assignment and iteratively applying this neighbour search until there are no lower scoring neighbours. Computationally, it is a relatively cheap optimisation method, and will always converge, but not necessarily to the global minimum.

*Setup* An initial electron assignment is generated as follows. For each possible electron assignment position $p \in {\mathcal{P}}$, a score is calculated and then an electron is assigned to the position which has the lowest score. This is iteratively applied, assigning a single electron at a time, until $e_T$ electrons have been assigned, giving the initial electron assignment.

*Neighbour searching* Local optimisation determines the score change that would result when going from one electron assignment to each neighbouring electron assignment, which are determined as follows. The multiset of possible positions for electrons to be assigned, ${\mathcal{P}}$, is converted to a set *P*, i.e. duplicate members are removed. Every member $p \in P$ is checked to determine if it contains electrons in the current electron assignment, i.e. $c_p[p] \ne 0$, meaning that it can be used as an electron source. If it can, all other members $q \in P \setminus p$ are checked to determine if they can hold another electron, i.e. $\text {mult}({\mathcal{P}},q) - c_p[q] > 0$, meaning that *q* can act as a target electron position. A neighbour of the current electron assignment is produced by moving an electron from a source position to a target position. Thus, all the neighbours of an electron assignment are given by the set of electron assignments produced from all possible source–target pairs. If multiple electron assignments with the same score exist, the neighbour searching can search the neighbours of all of the assignments, instead of just one of the assignments.

*Score minimisation* Determining an optimal electron assignment using the local optimisation method is straightforward. The score of each neighbour assignment is determined. If at least one of the neighbour(s) has a lower score, the neighbour search is repeated using the lowest scoring neighbour(s) as the initial electron assignments. This iterative update of the electron assignment proceeds until there are no neighbours with a lower score, in which case the optimisation process has converged to a local minimum.

#### A*

An A* approach was one of the three optimisation methods utilised by Dehof et al. for bond order assignment [10]. Such an approach is taken here for comparison purposes.

A* is a path-finding algorithm for determining a minimum cost path between a start, *s*, and end, *t*, location [20]. It employs a search heuristic as a means to guide the path finding process towards more promising paths. The list of vertices to search from is stored in a priority queue, meaning the most promising vertices are searched first. The priority is determined by assigning a cost, $f(r) = g(r) + h(r)$, where *g*(*r*) is the real cost of the path $s\dots r$ and *h*(*r*) is an heuristic estimate of the cost of the path $r\dots t$, to each visited vertex *r*. If the cost of a vertex *r* exceeds some upper limit value, that vertex will not be added to the priority queue. Here, this upper limit is calculated as the score of the initial local optimisation electron assignment (see “Local optimisation” section) plus one. Obviously, the nature of the heuristic function will influence the efficiency of the search algorithm. To be guaranteed to obtain a minimum cost path, the heuristic must be *admissible*, meaning ‘optimistic’. That is, the true cost of the path $r\dots t$ cannot be lower than *h*(*r*).

Let $P \subset {\mathcal{P}}$ be the set of unique possible positions to assign electrons. The score minimisation problem given the molecular graph $G = (V,E)$ can be formulated into a |*P*|-level tree *T*, i.e. the path from the root vertex to a leaf will be of length |*P*|. Each level of the tree represents a possible position for electrons to be assigned. A vertex at level *k* has $m + 1$ neighbours, where $m = \text {mult}({\mathcal{P}},w)$ and $w \in P$ is the position associated with level $k+1$, to allow for all possible electron counts placed in *w*, from 0 to *m*.

To formulate the scoring functions *g*(*r*) and *h*(*r*), some additional definitions must be made. A partial electron assignment, *R*(*r*), is denoted as the set of pairs (*j*, *n*) where *j* is a member of the path $s\dots r$ and $n \in \{0,\dots ,\text {mult}({\mathcal{P}},j)\}$ is the number of electrons assigned there. *R*(*r*) also contains pairs (*x*, 0) for all elements $x \in V \cup E \setminus P$.

*Q*(*r*) is the set of *calculable* members $x \in V \cup E$ at vertex *r*. *x* is deemed *calculable* at vertex $r \in T$ if the following conditions are met:

1. $x \notin P \setminus R_i$, where $R_i$ is the set of first members of *R*(*r*);
2. If $x \in V$, condition 1 holds for all neighbours of *x*;
3. If $x \in E$, the pair $x = {y,z} \subseteq Q(r)$.

As condition 3 is a requirement for determining the calculability of bonds, the calculability of all atoms is determined first.

*Cost Functions* Each vertex that is visited through the A* search is assigned a cost, $f(r) = g(r) + h(r)$. The exact cost of the path $s\dots r$, *g*(*r*), is defined as

7 $$\begin{aligned} g(r) = \sum _{x \in Q(r)} \Gamma [k_x] \end{aligned}$$

where $\Gamma [k_x]$ is the score of member $x \in V \cup E$ with partial electron assignment *R*(*r*), as defined in “Electron assignment scoring” section. If the number of electrons assigned in *R*(*r*) is greater than $e_T$, $g(r) = \infty$.

The heuristic cost of the path $r\dots t$, *h*(*r*) is defined as

8 $$\begin{aligned} h(r) = \sum _{x \in Q(r)^\complement } \min \left\{ a \in B(x) : \Gamma [k_{x,a}]\right\} \end{aligned}$$

where $Q(r)^\complement$ is the complement of *Q*(*r*).

If $x \in E$, such that $x = \{y,z\}$ then *B*(*x*) is the set of possible numbers of electrons to assign to *x* and $k_{x,a}$ determines the key for *x* given *R*(*r*) with an additional *a* electrons assigned to *x*. *B*(*x*) is given by

9 $$\begin{aligned} B(x) = \left\{ b \in \left\{ 0,\dots ,\text {mult}({\mathcal{P}},x)\right\} : V(y) + b \le \tau _y \wedge V(z) + b \le \tau _z \right\} \end{aligned}$$

where *V*(*y*) is the valence of *y* in the partial electron assignment *R*(*r*).

If $x \in V$, then *B*(*x*) is the set of formal charge values *x* can attain given *R*(*r*), and $k_{x,a}$ determines the key for *x* assuming it has a formal charge of *a*. In this case, *B*(*x*) is given by

10 $$\begin{aligned} B(x) = \left\{ F(x) + b \in \{0, \dots , \tau _x - V(x) \} \right\} \end{aligned}$$

where *F*(*x*) is the formal charge of *x* given *R*(*r*) calculated as per Eq. (5).

#### Fixed parameter tractable (FPT)

In a similar vein to Dehof et al. [10], we also implement an FPT based approach. Given a molecular graph $G = (V,E)$, which is a tree, the electron assignment problem can be easily solved using dynamic programming, i.e. recursively splitting the problem into smaller sub problems and solving the sub problems. Not all molecular graphs are trees, but their generally sparse nature means that they are ‘tree-like’.

Given a graph $G = (V, E)$, the *tree-decomposition* of *G*, $(T, {\mathcal{V}})$, where *T* is a tree and ${\mathcal{V}} = (V_t)_{t \in T}$ is a family of vertex bags $V_t \subseteq V$ indexed by the nodes *t* of *T*. $(T, {\mathcal{V}})$ satisfies the following three conditions:

(i) $$V = \bigcup _{t\in T}V_t$$;
(ii) for every edge $e \in E$ where $e = \{u,v\}$ there exists a $t \in T$ such that $e \subseteq V_t$;
(iii) $V_{t_1} \cap V_{t_3} \subseteq V_{t_2}$ whenever $t_1,t_2,t_3 \in T$ satisfy $t_2 \in t_1Tt_3$.

**Fig. 1:**
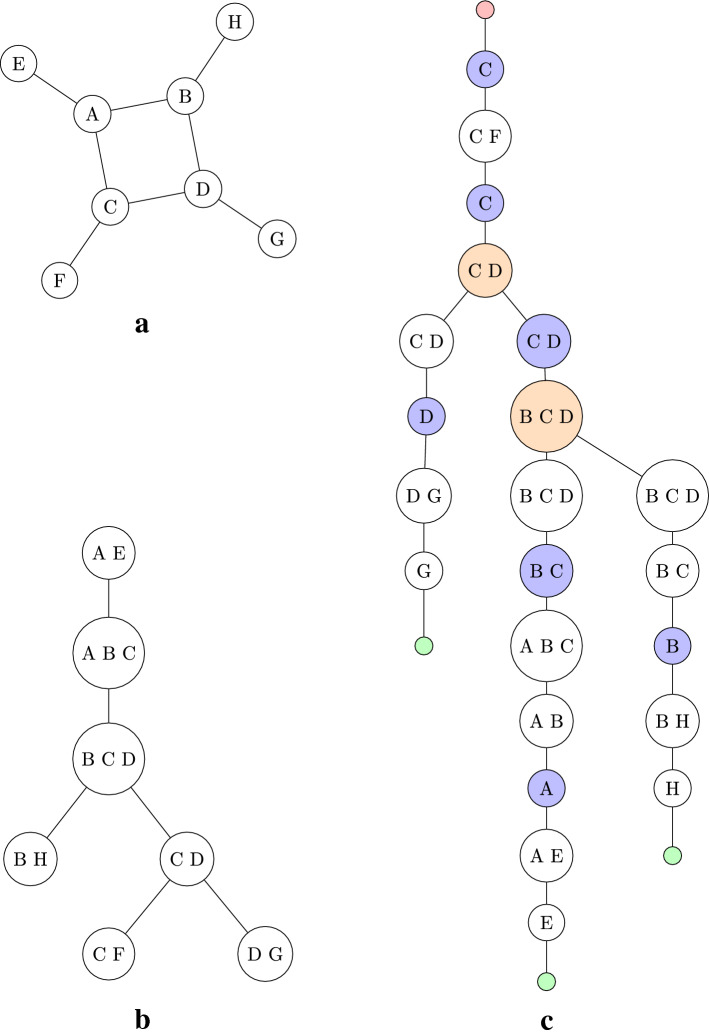
A graph **a** $G = (V,E)$ with $V = \{\text {A,B,C,D,E,F,G,H}\}$ and $E = \{\text {(A,B),(A,C),(A,E),(B,D),(B,H),(C,D),(C,F),(D,G)}\}$, a tree-decomposition **b** $(T,{\mathcal{V}})$ of *G* and **c** a nice tree-decomposition. The nodes of the nice tree-decomposition are coloured red for the root node, green for leaf nodes, white for introduce nodes, blue for forget nodes and orange for join nodes

The *width* of $(T,{\mathcal{V}})$ is the number $\max \big \{|V_t| - 1 : t \in T\big \}$. This width gives the fixed parameter. Figure 1b shows a tree-decomposition of the graph in Fig. 1a. It has a width of two.

In order to simplify the algorithm, the concept of a *nice* tree-decomposition is used. A tree-decomposition $(T, {\mathcal{V}})$ is called nice if it satisfies the following conditions:

1. *T* is rooted at a leaf node *r* and $V_r = \emptyset$;
2. For every leaf $l \in T$, $V_l = \emptyset$;
3. Every node $t \in T$ has at most two children;
4. If $t \in T$ has two children, *c* and *d*, then $V_t = V_c = V_d$ and *t* is known as a join node;
5. If $t \in T$ has one child, *c*, then one of the following conditions is true:
  - $V_t \subset V_c$ and $|V_t| = |V_c| - 1$ and *t* is known as a forget node with forgotten vertex $v_{t_f} {:}{=} V_c \setminus V_t$.
  - $V_t \supset V_c$ and $|V_t| = |V_c| + 1$ and *t* is known as an introduce node with introduced vertex $v_{t_i} {:}{=} V_t \setminus V_c$.

Forget and introduce nodes are defined in relation to the path from a leaf node to the root node. Figure 1c shows a nice tree decomposition produced from the tree decomposition in Fig. 1b.

The electron assignment optimisation process requires scores to be determined for both atoms and bonds. As a nice tree-decomposition introduces and forgets vertices, the edges of *G* must be treated as vertices. To do this, the edges are explicitly added into the bags of the tree-decomposition of *G*, at the expense of a larger width. An edge is introduced at the same time the first of its vertices is introduced, and forgotten once the second vertex is forgotten.

*Algorithm* A tree-decomposition of a molecular graph is obtained using the GreedyFillIn upper-bound heuristic described by Bodlaender and Koster, [21] and converted to a nice tree decomposition. Optimisation of electron assignment then proceeds as follows. Let $t \in T$ be a node of the nice tree-decomposition of a graph. Then ${\mathcal{X}}_t$ is the set of forgotten vertices $v_{s_f} \in V_s$ associated with the forget nodes of the subtree $T_s$ induced on *T* below (and including) the node *t*. The total number of electrons to assign $e_T$ and the multiset ${\mathcal{P}}$ of positions at which the electrons can be assigned are determined as per “Initialisation” section. Each node *t* is given a score table $S_t$ indexed by the ordered pair $(l,k) \in L_t \times K_t$ where

11 $$\begin{aligned} L_t&= \{n_\text {min},\dots ,n_\text {max}\}, \end{aligned}$$

12 $$\begin{aligned} n_\text {min}&= \max \{0, e_T - |{\mathcal{P}}| + |{\mathcal{P}} \cap {\mathcal{X}}_t|\}, \end{aligned}$$

13 $$\begin{aligned} n_\text {max}&= \min \{e_T, |{\mathcal{P}} \cap {\mathcal{X}}_t|\}, \end{aligned}$$

14 $$\begin{aligned} K_t&= X_1 \times \cdots \times X_j, \end{aligned}$$

15 $$\begin{aligned} X_j&= \{(j,k):j\in V_t, 0 \le k \le \text {mult}({\mathcal{P}}, j)\}, \end{aligned}$$

with $S_t[l,k]$ being the minimum score of forgotten vertices ${\mathcal{X}}_t$ with $l \in L_t$ forgotten electrons and the additional constraint of further partial electron distribution $k \in K_t$. Beginning from the leaves of the nice tree-decomposition, and scoring only when all children of a node have been scored, the algorithm distinguishes the kind of each node and determines the score matrix as follows:

*Leaf node* Leaf nodes are empty sets, so the score table of a leaf node is also empty.

*Introduce nodes *Let $t \in T$ be the introduce node with child *c*, and $x_t = {\mathcal{X}}_t \setminus {\mathcal{X}}_c$. Then

16 $$\begin{aligned} S_t[l,k] = {\left\{ \begin{array}{ll} S_c[l,k\setminus x_t] & {} \text {if } k\setminus x_t \ne \emptyset , \\ \infty & {} \text {otherwise}. \end{array}\right. } \end{aligned}$$

*Forget nodes* Let $t \in T$ be the forget node with child *c*, and $x_t = {\mathcal{X}}_c \setminus {\mathcal{X}}_t$. Then

17 $$\begin{aligned} S_t[l,k] = \min _{\begin{array}{c} n \in \{0, \dots , \text {mult}({\mathcal{P}}, x_t)\} \\ p \in L_c : p + n = l \end{array}} \Big \{E(x_t,k \cup {\mathcal{X}}_t,n) + S_c[p, k \cup x_t]\Big \} \end{aligned}$$

where $E(x_t,k \cup {\mathcal{X}}_t,n)$ is the score of $x_t$ with *n* electrons positioned and the partial electron distribution $k \cup {\mathcal{X}}_t$.

*Join nodes* Let $t \in T$ be the parent of *c* and *d* with $V_t = V_i$ for $i \in {c,d}$. Then

18 $$\begin{aligned} S_t[l,k] = \min _{(p,q) \in L_{c} \times L_{d} : p+q = l} \Big \{S_{c}[p,k] + S_{d}[q,k]\Big \} \end{aligned}$$

*Root node* Each nice tree decomposition has only one root node $r \in T$ which is formally a forget node. However the score table of the root node is unpopulated as $K_r = \emptyset$. Rather than fill a score table, the minimised electron assignment score is determined. Let *c* be the child of *r* with $x_r = {\mathcal{X}}_c$. Then the minimum score is given by

19 $$\begin{aligned} S_\text {min} = \min _{\begin{array}{c} n \in \{0, \dots , \text {mult}({\mathcal{P}}, x_r)\} \\ p \in L_c : p + n = e_T \end{array}} \Big \{S(x_r,{\mathcal{X}}_r,n) + S_r[p, x_r]\Big \} \end{aligned}$$

### Practical optimisation

There are a number of techniques which can be used to optimise the electron assignment algorithms described above. As opposed to implementation optimisation techniques which do not affect the outcome of the algorithms, only the computational cost of performing them, these are considered practical optimisations as they could influence the results obtained, but generally would not be expected to do so.

*Electron pairs* A simple means to optimise the algorithms is to utilise electron pairs instead of single electrons. Generally, one would expect electrons to be found in pairs anyway: two electrons per bond order and lone pairs of electrons on atoms. By explicitly assigning pairs, the search space can be massively reduced, leading to an increase in performance. This optimisation is recommended, is the default setting, and is used for all results presented here.

*Pre-placing electrons* In the majority of molecules, there are a number of elements to which a minimum number, larger than zero, of electrons are expected to be assigned. For example, the halogens would be expected to have three lone pairs of electrons assigned when they are bonded to only one other atom. By placing an expected minimum number of electrons on various atoms of the molecule before undertaking the optimisation, the search space of the algorithms is reduced, and so the optimisation cost is reduced.

### Reference formal charge and bond order assignments

Validation of the accuracy of our method requires reference data, i.e. a set of molecules for which both formal charge and bond order properties are already known. The reference data must also represent aromatic bonds in kekulised form, i.e. alternating single and double bonds, and hydrogen atoms must be explicitly present or able to be added automatically. Two molecular databases that fit these requirements were chosen as reference data sets: the MMFF94 validation suite [22] and the KEGG Drug Database [23]. These databases were previously used to validate the bond order assignment methods developed by Dehof et al. [10].

All molecules in each reference data set were parsed to extract the element and connectivity information required for our internal graph theoretic representation (see “Initailisation” section). In cases where one structure file contained multiple molecules, the molecules were treated separately. Molecules were discarded if they contained dangling bonds due to being monomer units, if they contained three or fewer non hydrogen atoms, if they contained elements not included in the score tables, if they contained an odd number of valence electrons, or if they were identical to a previously parsed molecule.

The MMFF94 validation suite contains 761 structures for small molecules and ions, 698 of which are derived from the CSD. The native CSD structures were manually modified by the authors, by assigning bond orders and formal charges and, where appropriate, adding missing hydrogen atoms to complete the valence [22]. Formal charges and bond orders are available in either hypervalent or dative representation, with the hypervalent representation used here. After filtering using the rules described above, 691 unique molecules were identified. Canonical SMILES strings for these structures are given in Additional file 1: Table S7.

The KEGG Drug Database contains a large number of drug like molecules [23]. Its structure files contain only 2D coordinate information, meaning that they are a perfect test set for connectivity-only bond order and formal charge assignment. Hydrogen atoms are not explicitly present in the structure files. Rather, they are implicitly given through providing types to the heavy atoms. Accordingly, explicit hydrogen atoms were added to the molecules using this atom type information [24]. After filtering using the rules described above, 5676 unique molecules were identified. Canonical SMILES strings for these molecules are given in Additional file 1: Table S8.

## Results and discussion

We first discuss the consistency of the three optimisation algorithms described above by comparing the optimised electron assignment scores that they provide. Then we discuss the accuracy of the FPT algorithm in regards to its ability to reproduce the formal charge and bond order assignments provided by the two reference databases.

### Algorithm consistency

For the consistency tests described here, the scores used were derived from calculations performed using the def2-SVPD basis set, without accounting for the basis set superposition error. This set of scores was chosen as for self-consistency tests it does not matter whether the optimised score corresponds to the true formal charge and bond order state, rather only that the algorithms are correctly determining the lowest-scoring state. Electron pairs were utilised for maximum performance. Electrons were not pre-placed.

As expected, we find that the A* and FPT algorithms have 100% agreement in regards to the optimised score, with both molecular databases. This is reassuring as it indicates correct implementation of the two algorithms. In relation to these, the local optimisation algorithm performs remarkably well. For the MMFF94 database, there is 81% agreement with the A* and FPT algorithms, and for the KEGG drug database, there is 91% agreement with the A* and FPT algorithms. Such high percentage accuracies indicate that using the highly efficient local optimisation algorithm could potentially be acceptable in extremely high throughput applications where overall speed is more important than accuracy.

### Algorithm efficiency

**Fig. 2:**
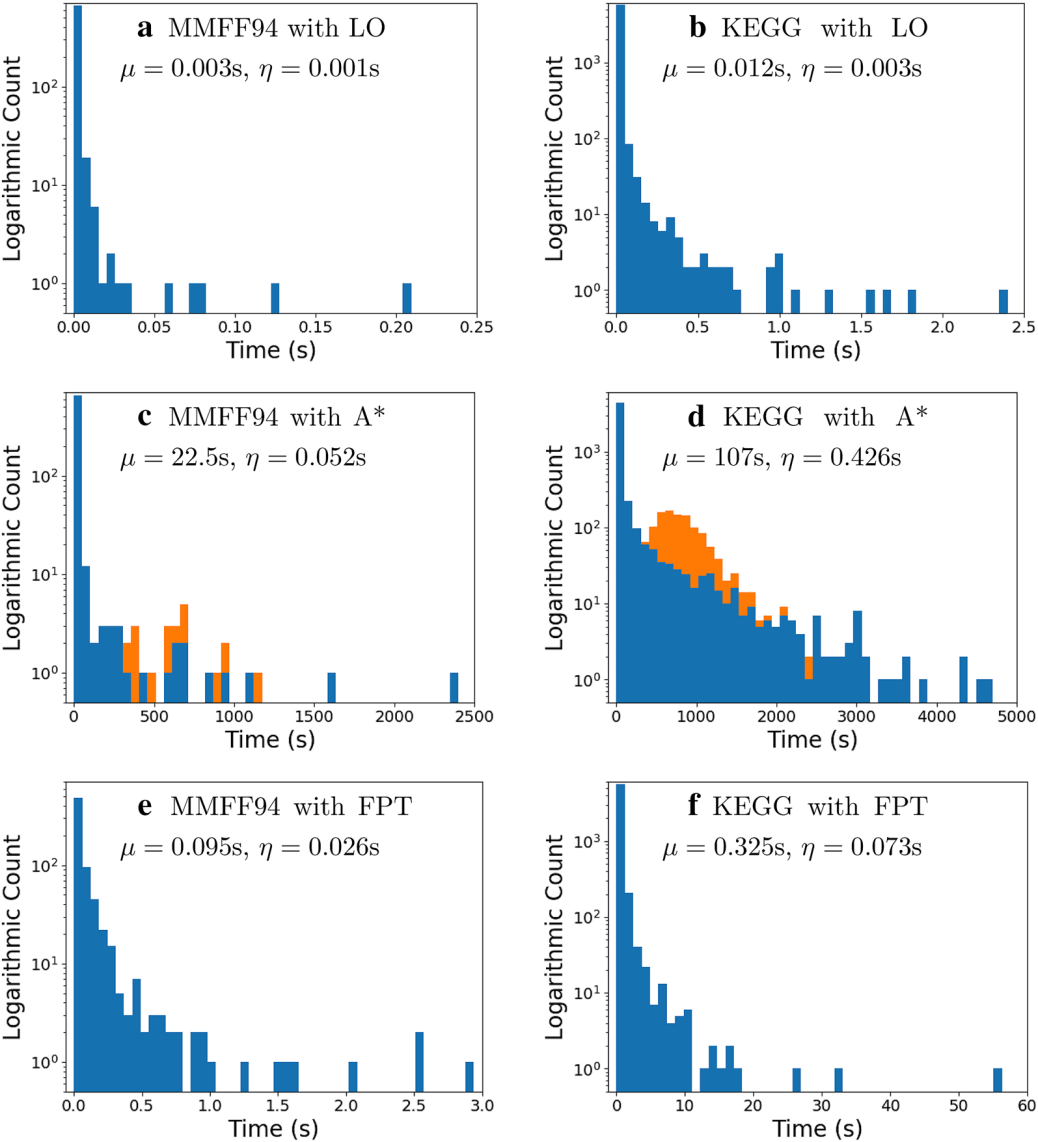
Histograms of algorithm execution time for the three optimisation algorithms and two data sets. For all plots, the horizontal axis shows the execution time of the algorithm in seconds and the vertical axis shows the base-ten logarithm of the count for each bin. Fifty bins of even width were used. Mean ($\mu$) and median ($\eta$) values for the distributions in blue are provided. **a**, **b** Show the time distributions for the MMFF94 and KEGG databases when optimised with the Local Optimisation algorithm. In both cases, a single outlier with an execution time in excess of 200 s is excluded from the plots. **c**, **d** Show the time distributions for the MMFF94 and KEGG databases when optimised with the A* algorithm. The orange bins indicate molecules that failed to complete optimisation due to a 1024 MB memory limit imposed on the A* search queue. **e**, **f** Show the time distributions for the MMFF94 and KEGG databases when optimised with the FPT algorithm

We next consider the efficiency of each algorithm, defined as the average time required to find the lowest score for a single molecule. The distributions of calculation times for each algorithm and each dataset are shown in Fig. 2.

From the distributions, we can tell that the local optimisation algorithm is the most efficient with a maximum 90-th percentile execution time of only 0.015 s, followed by the FPT algorithm with a maximum 90-th percentile execution time of 0.656 s and finally the A* algorithm with a maximum 90-th percentile execution time of 212 s when molecules that failed to complete optimisation due to a 1024 MB memory limit imposed on the A* search queue are excluded. All algorithms show exponential decay in the execution time, showing that in the majority of cases, one would expect any of the algorithms to give a result in a reasonable time period. This is further reinforced by the low median execution times.

Local optimisation, Fig. 2a and b, was the stand-out efficiency algorithm, though both databases had a single outlier molecule which took longer than 200 seconds to optimise. In these two cases, the initial electron assignment required a large number of neighbour search steps before a distribution with non-infinite score was discovered, which could then be minimised. Due to the extremely long computational time required for these two molecules, an upper limit on the execution time of 5 s was implemented for the local optimisation algorithm.

The A* algorithm, Fig. 2c and d, is the least efficient of the three algorithms. A desirable A* search would be narrow throughout the search tree. The breadth of the search is primarily controlled by the heuristic function, where a better heuristic would result in a narrower search. However, even with a near-optimal heuristic, there can be cases where the leaves of the search tree have final scores with fractions of a percent difference between them. In these cases, the path through the search tree becomes broad, and so the overall algorithm efficiency decreases. Due to this, a memory limit of 1024 MB was imposed on the A* algorithm. This limit means that when the memory allocated for the queue exceeds the given amount, the algorithm halts without returning a solution. Molecules which triggered this limit are shown in the orange distributions in Fig. 2c and d, where the execution time is the time taken before the limit was triggered. We note that there are molecules which did not trigger the memory limit but took longer to complete than the triggering time of any triggered molecules. This can be attributed to larger molecules requiring deep searches that are not necessarily as broad as other smaller molecules, so they can complete without triggering the memory limit.

Finally, the FPT algorithm, Fig. 2e and f is a highly efficient algorithm that is also guaranteed to locate the global minimum score value. Though the vast majority of molecules take less than a second to determine an optimal score using the FPT algorithm, there are a small number of molecules which take in excess of ten seconds to complete. The execution times of these molecules are so long as their tree-decomposition contains wide join nodes that have a large number of potentially forgotton electrons. These large join nodes come about primarily due to areas within the molecule which can contain a broad range of electron counts, especially when the width of the tree-decomposition is high, such as highly conjugated aromatic systems or high electron density areas like sulfate or phosphate groups.

### Comparison to reference assignments

The true measure of the accuracy of each algorithm is its ability to reproduce the formal charges and bond orders of the molecules in the reference data sets. Here we only consider the FPT algorithm as “Algorithm consistency” section showed that it is far more efficient than the A* algorithm whilst still providing a global minimum score.

Additionally, we compare our results to the A* method described by Dehof et al. [10] as implemented in BALL [25]. The A* method is utilised as the FPT method is not accessible through the provided Python bindings.

We note that the score of two resonance structures will be identical, whereas the formal charges and bond orders will not, even though each resonance state is a valid, and minimum score, formal charge and bond order assignment. As the reference data only contains formal charge and bond order assignments for a single resonance structure, the FPT algorithm was modified to be capable of producing all possible resonance structures for a given molecule. However, due to the combinatorial explosion possible when there are multiple, disjoint resonance substructures within a molecule, and the resulting increase in intermediate computational load such an explosion would have on the algorithm, an upper limit to the number of resonance structures obtained of 32 was implemented.

For the comparisons to the reference data sets, a calculated formal charge and bond order assignment, from either the FPT algorithm described here or the A* algorithm described by Dehof et al., is deemed correct if one of the up to 32 assignments returned by the algorithm exactly matches that of the reference data. In some cases, there will be more than 32 potential resonance structures. If the reference assignment is not matched within these first 32 results returned, that molecule is regarded as failing for the algorithm, regardless of whether or not the returned assignments are correct resonance structures for the reference assignment. The results of these comparisons are given in Table 1.

**Table 1:** Percentage of optimised electron assignment scores for which the corresponding formal charge and bond order state matches the reference state

Database	FPT algorithm	BALL
MMFF94 (691)	93.49% (646)	84.80% (586)
KEGG (5676)	98.13% (5570)	93.59% (5312)
Overall (6367)	97.63% (6216)	92.63% (5898)

Counts are given in brackets. The BALL column gives the accuracy results obtained when only checking if the bond orders obtained match the reference data

Scores for our FPT algorithm were derived from quantum chemical calculations using the def2-SVPD or def2-TZVPPD basis set, with or without correction for basis set superposition error. All four sources of scores performed identically and as such, the cheapest level of theory is recommended, and used for the results presented here. Additionally, the scores associated with a $\hbox {C}^0$ atom and a $\hbox {C}^{-}$ atom were swapped so as to make a neutral carbon always be more favourable than a charged carbon. This increased the overall accuracy from 95.63 to 97.63%.

These results show that our method has better accuracy than that of Dehof et al. across both databases. Our accuracy is similar accuracy to that of other state of the art bond order assignment methodologies, [26–29] while additionally assigning formal charges. We note that because Dehof et al.’s method is not designed for determining formal charges as well as bond orders, the accuracy values that we report for their algorithm should be taken as an upper limit of accuracy, as only correct assignment of bond orders are checked. Any check of formal charge correctness, for example through back calculation from the bond orders, will not be able to exceed these accuracy levels, as the bond order checking is a subset of the combined bond order and formal charge checking.

The method presented by Dehof et al. makes use of arbitrary, but empirically optimised, penalty scores for their bond order assignment, whereas the method presented here utilises scores derived directly from high-level quantum chemical calculations, other than the single swap of the $\hbox {C}^0$ and $\hbox {C}^{-}$ scores. This direct derivation makes our scoring function easily extensible to other atom and bond types. Empirical optimisation of the scores used here could increase the accuracy of our algorithm, but at the expense of ease of extensibility.

The 151 molecules for which the FPT algorithm failed to generate a correct assignment include two major groups of chemically similar molecules. The first group consists of 103 molecules containing at least one nitrogen atom assigned a formal charge of $-\,1$ in the reference data. Overall, 106 such molecules are found in the reference data, showing that only three were correctly assigned by our algorithm. The formal charges and bond orders of nearly all of these molecules were not correctly assigned, indicating that the relative scores for neutral and negatively-charged nitrogen atoms, combined with the scores for single and double bonds involving nitrogen, are unable to produce correct assignments. These nitrogen atoms are generally located in groups such azides or diazos. These groups are generally presented as containing nitrogen atoms with positive and a negative charge adjacent to one another, whereas our algorithm assigns them both neutral charges. Additionally, they are joined by a double bond in the reference data but by a single bond in our results. This illustrates how our scoring system leads to a preference for fewer formal charges.

The second group comprise molecules where some of our underlying assumptions do not hold. For example, for protonated acetone, the MMFF94 database assigns the oxygen atom a formal charge of $+\,1$ and places a double bond between the oxygen and central carbon atom, whereas our algorithm assigns a formal charge of $+\,1$ to the central carbon atom and assumes that an oxygen atom bonded to two other atoms will have two lone pairs of electrons. Along with the backbone sigma bond electron pairs, this means that there are no missing electron pairs to assign, thus leading to the positive formal charge on the central carbon and concomitant single rather than double bond.

## Conclusion

We have developed a method for determining optimal bond order and formal charge assignments utilising electron assignment scores derived from atom/ion and bond dissociation energies calculated with high-level quantum chemical methods, and tested their performance using three different optimisation methods—local optimisation, an A* pathfinding algorithm, and an FPT algorithm.

While the FPT algorithm is less efficient than local optimisation, its greater accuracy was considered the more important feature. We found no difference in the accuracy of the FPT algorithm when the electron assignment scores were derived from calculations at difference quantum chemical levels of theory, indicating that extension of the scoring function to additional element and bond types need only consider performing quantum chemical calculations at the lowest level of theory used here.

In comparison with the state of the art method of Dehof et al., the FPT algorithm developed here performs remarkably well, attaining relatively similar accuracy levels. We also show that the scores provided here can be easily optimised in order to increase the accuracy, though doing so will remove the extensibility of scores derived from quantum chemical calculations. Our method is well suited to use in computational chemistry and cheminformatic applications where the user supplies only minimal information, as it requires only atom types and connectivity.

## Additional file

**Additional file 1.** Calculated atom and bond score tables, and canonical SMILES strings of the test molecules.
