# Supplementary material for: Automated simultaneous assignment of bond orders and formal charges
Source: J Cheminform. 2019 Mar 6;11:18. doi: 10.1186/s13321-019-0340-0 (PMC6419789; doi:10.1186/s13321-019-0340-0)
Supplement: Supplementary file 1 — Additional file 1. Calculated atom and bond score tables, and canonical SMILES strings of the test molecules. [file 13321_2019_340_MOESM1_ESM.pdf]

Supplementary Information for "Automated simultaneous assignment of bond orders and formal charges"

Ivan D Welsh[1,2] and Jane R Allison*[1,2,3,4]

[1]Centre for Theoretical Chemistry and Physics, Institute of Natural and Mathematical Sciences, Massey University Auckland, Private Bag 102904, 0632 Auckland, New Zealand
[2]School of Biological Sciences, University of Auckland, Private Bag 92019, Auckland, New Zealand
[3]Biomolecular Interaction Centre, University of Canterbury, Private Bag 4800, Christchurch 8140, New Zealand
[4]Maurice Wilkins Centre for Molecular Biodiscovery, University of Auckland, Private Bag 92019, Auckland, New Zealand

Contents

Table S1: Scores for atoms with formal charges computed using the def2-SVPD basis set (page 2).
Table S2: Scores for atoms with formal charges computed using the def2-TZVPPD basis set (page 3).
Table S3: Scores for bonds computed using the def2-SVPD basis set (page 4).
Table S4: Scores for bonds computed using the def2-SVPD basis set and accounting for the basis set superposition error (page 5).
Table S5: Scores for bonds computed using the def2-TZVPPD basis set (page 6).
Table S6: Scores for bonds computed using the def2-TZVPPD basis set and accounting for the basis set superposition error (page 7).
Table S7: Canonical SMILES strings for the molecules in the MMFF94 data set used for testing (page 8).
Table S8: Canonical SMILES strings for the molecules in the KEGG data set used for testing (page 17).

Code developed and presented in the manuscript is available at `https://git.io/vH7hz`.

*j.allison@auckland.ac.nz

Table S1: Scores for atoms with formal charges computed using the def2-SVPD basis set.

H^-	0.02013	H^0	0.00000	H^+	0.49928
C^{4-}	1.45670	C^{3-}	0.66591	C^{2-}	0.20712
C^-	-0.07677	C^0	-0.05938	C^+	0.34948
C^{2+}	1.24107	C^{3+}	2.98409	C^{4+}	5.32103
N^{3-}	0.90604	N^{2-}	0.33772	N^-	-0.02650
N^0	-0.10641	N^+	0.42292	N^{2+}	1.50554
N^{3+}	3.24192	N^{4+}	6.06052	N^{5+}	9.61128
O^{2-}	0.11254	O^-	-0.12968	O^0	-0.08409
O^+	0.40147	O^{2+}	1.68570	O^{3+}	3.69651
O^{4+}	6.52685	O^{5+}	10.67027	O^{6+}	15.68525
F^-	-0.12113	F^0	0.00000	F^+	0.63130
F^{2+}	1.89498	F^{3+}	4.19052	F^{4+}	7.38363
F^{5+}	11.55460	F^{6+}	17.27203	F^{7+}	24.00196
P^{3-}	0.67413	P^{2-}	0.21734	P^-	-0.04018
P^0	-0.06960	P^+	0.30853	P^{2+}	1.02731
P^{3+}	2.12335	P^{4+}	3.99512	P^{5+}	6.35594
S^{2-}	0.03364	S^-	-0.11824	S^0	-0.05233
S^+	0.30977	S^{2+}	1.16010	S^{3+}	2.42590
S^{4+}	4.14584	S^{5+}	6.78903	S^{6+}	9.98929
Cl^-	-0.12658	Cl^0	0.00000	Cl^+	0.46359
Cl^{2+}	1.31675	Cl^{3+}	2.76026	Cl^{4+}	4.69375
Cl^{5+}	7.15408	Cl^{6+}	10.68369	Cl^{7+}	14.83826
Br^-	-0.12165	Br^0	0.00000	Br^+	0.42202
Br^{2+}	1.18501	Br^{3+}	2.46653	Br^{4+}	4.16950
Br^{5+}	6.32020	Br^{6+}	9.45716	Br^{7+}	13.14507

Table S2: Scores for atoms with formal charges computed using the def2-TZVPPD basis set.

H^{-}	-0.00264	H^{0}	0.00000	H^{+}	0.49981
C^{4-}	1.10560	C^{3-}	0.48600	C^{2-}	0.12642
C^{-}	-0.09020	C^{0}	-0.05385	C^{+}	0.35759
C^{2+}	1.24978	C^{3+}	3.00539	C^{4+}	5.37111
N^{3-}	0.70853	N^{2-}	0.20738	N^{-}	-0.06710
N^{0}	-0.10055	N^{+}	0.43125	N^{2+}	1.51559
N^{3+}	3.25361	N^{4+}	6.09490	N^{5+}	9.68634
O^{2-}	0.09397	O^{-}	-0.12915	O^{0}	-0.08232
O^{+}	0.40942	O^{2+}	1.69748	O^{3+}	3.71155
O^{4+}	6.54831	O^{5+}	10.72593	O^{6+}	15.79333
F^{-}	-0.11934	F^{0}	0.00000	F^{+}	0.63230
F^{2+}	1.90703	F^{3+}	4.20886	F^{4+}	7.40644
F^{5+}	11.59413	F^{6+}	17.35880	F^{7+}	24.15246
P^{3-}	0.47698	P^{2-}	0.11858	P^{-}	-0.07367
P^{0}	-0.06395	P^{+}	0.32033	P^{2+}	1.04397
P^{3+}	2.14834	P^{4+}	4.03146	P^{5+}	6.41082
S^{2-}	0.02470	S^{-}	-0.12008	S^{0}	-0.05080
S^{+}	0.32100	S^{2+}	1.17760	S^{3+}	2.45359
S^{4+}	4.18644	S^{5+}	6.84375	S^{6+}	10.06529
Cl^{-}	-0.12662	Cl^{0}	0.00000	Cl^{+}	0.46802
Cl^{2+}	1.33168	Cl^{3+}	2.78608	Cl^{4+}	4.73544
Cl^{5+}	7.21469	Cl^{6+}	10.76125	Cl^{7+}	14.93864
Br^{-}	-0.12263	Br^{0}	0.00000	Br^{+}	0.42725
Br^{2+}	1.20276	Br^{3+}	2.49427	Br^{4+}	4.20793
Br^{5+}	6.36814	Br^{6+}	9.51383	Br^{7+}	13.21067

Table S3: Scores for bonds computed using the def2-SVPD basis set.

C−C	-0.15291	C=C	-0.27810	C≡C	-0.40270
C−H	-0.17404	C−N	-0.14340	C=N	-0.25116
C≡N	-0.36505	C−O	-0.15198	C=O	-0.27907
C−P	-0.11521	C=P	-0.18584	C≡P	-0.26547
C−S	-0.11820	C=S	-0.19788	H−N	-0.17752
H−O	-0.19299	H−P	-0.13348	H−S	-0.14536
N−N	-0.11113	N=N	-0.19335	N≡N	-0.33520
N−O	-0.10397	N=O	-0.17890	N−P	-0.11862
N=P	-0.15815	N≡P	-0.20025	N−S	-0.10161
N=S	-0.13377	O−O	-0.07599	O=O	-0.17301
O−P	-0.13497	O=P	-0.19210	O−S	-0.10182
O=S	-0.17044	P−P	-0.08651	P=P	-0.11807
P≡P	-0.15283	P−S	-0.09656	P=S	-0.13096
S−S	-0.08676	S=S	-0.13372	Br−Br	-0.06626
Br−Cl	-0.06827	Br−F	-0.08855	Br−H	-0.14444
Br−N	-0.08229	Br−O	-0.07551	Br−P	-0.09768
Br−S	-0.07593	Br−C	-0.11578	C−Cl	-0.13052
C−F	-0.17733	Cl−Cl	-0.06780	Cl−F	-0.08043
Cl−H	-0.16247	Cl−N	-0.08910	Cl−O	-0.07507
Cl−P	-0.11142	Cl−S	-0.08294	F−F	-0.04591
F−H	-0.21700	F−N	-0.10916	F−O	-0.06877
F−P	-0.16600	F−S	-0.11864	H−H	-0.16613

Table S4: Scores for bonds computed using the def2-SVPD basis set and accounting for the basis set superposition error.

C−C	-0.14485	C=C	-0.27090	C≡C	-0.39622
C−H	-0.17145	C−N	-0.13471	C=N	-0.24229
C≡N	-0.35592	C−O	-0.14347	C=O	-0.26999
C−P	-0.10658	C=P	-0.17688	C≡P	-0.25695
C−S	-0.10833	C=S	-0.18802	H−N	-0.17393
H−O	-0.18879	H−P	-0.12958	H−S	-0.14091
N−N	-0.10223	N=N	-0.18375	N≡N	-0.32536
N−O	-0.09553	N=O	-0.16797	N−P	-0.10894
N=P	-0.14858	N≡P	-0.19063	N−S	-0.09117
N=S	-0.12430	O−O	-0.06878	O=O	-0.16589
O−P	-0.12550	O=P	-0.18123	O−S	-0.09185
O=S	-0.15843	P−P	-0.07861	P=P	-0.10971
P≡P	-0.14410	P−S	-0.08769	P=S	-0.12180
S−S	-0.07792	S=S	-0.12616	Br−Br	-0.05798
Br−Cl	-0.06122	Br−F	-0.07919	Br−H	-0.13761
Br−N	-0.07266	Br−O	-0.06598	Br−P	-0.08883
Br−S	-0.06746	Br−C	-0.10523	C−Cl	-0.12052
C−F	-0.17000	Cl−Cl	-0.06038	Cl−F	-0.07323
Cl−H	-0.15659	Cl−N	-0.07982	Cl−O	-0.06654
Cl−P	-0.10277	Cl−S	-0.07483	F−F	-0.04068
F−H	-0.21349	F−N	-0.10145	F−O	-0.06220
F−P	-0.15731	F−S	-0.10923	H−H	-0.16612

Table S5: Scores for bonds computed using the def2-TZVPPD basis set.

Br−Br	-0.07774	Br−Cl	-0.08190	Br−F	-0.09598
Br−H	-0.14805	Br−N	-0.08979	Br−O	-0.08459
Br−P	-0.10613	Br−S	-0.08819	Br−C	-0.12095
C−C	-0.15215	C−Cl	-0.13490	C−F	-0.17778
C−H	-0.17734	C−N	-0.14225	C−O	-0.15201
C−P	-0.11819	C−S	-0.12217	Cl−Cl	-0.08521
Cl−F	-0.09154	Cl−H	-0.16769	Cl−N	-0.09728
Cl−O	-0.08631	Cl−P	-0.12090	Cl−S	-0.09746
F−F	-0.05453	F−H	-0.22120	F−N	-0.11131
F−O	-0.07488	F−P	-0.17418	F−S	-0.12760
H−H	-0.17292	H−N	-0.18088	N−N	-0.11148
N−O	-0.10592	N−P	-0.12405	N−S	-0.10817
H−O	-0.19670	O−O	-0.08106	O−P	-0.14314
O−S	-0.11114	H−P	-0.13722	P−P	-0.09212
P−S	-0.10515	H−S	-0.15018	S−S	-0.09934
C=C	-0.28395	C=N	-0.25506	C=O	-0.28278
C=P	-0.19396	C=S	-0.20642	N=N	-0.19762
N=O	-0.18440	N=P	-0.16929	N=S	-0.14599
O=O	-0.18188	O=P	-0.20883	O=S	-0.18892
P=P	-0.12907	P=S	-0.14502	S=S	-0.15312
C≡C	-0.42049	C≡N	-0.37818	C≡P	-0.28179
N≡N	-0.34742	N≡P	-0.21513	P≡P	-0.16920

Table S6: Scores for bonds computed using the def2-TZVPPD basis set and accounting for the basis set superposition error.

Br−Br	-0.07253	Br−Cl	-0.07827	Br−F	-0.09073
Br−H	-0.14372	Br−N	-0.08401	Br−O	-0.07888
Br−P	-0.10189	Br−S	-0.08366	Br−C	-0.11532
C−C	-0.15022	C−Cl	-0.13227	C−F	-0.17572
C−H	-0.17646	C−N	-0.14014	C−O	-0.14967
C−P	-0.11640	C−S	-0.11951	Cl−Cl	-0.08290
Cl−F	-0.08916	Cl−H	-0.16569	Cl−N	-0.09453
Cl−O	-0.08340	Cl−P	-0.11867	Cl−S	-0.09491
F−F	-0.05322	F−H	-0.21939	F−N	-0.10913
F−O	-0.07280	F−P	-0.17200	F−S	-0.12469
H−H	-0.17291	H−N	-0.17960	N−N	-0.10899
N−O	-0.10376	N−P	-0.12181	N−S	-0.10518
H−O	-0.19492	O−O	-0.07877	O−P	-0.14082
O−S	-0.10816	H−P	-0.13608	P−P	-0.09084
P−S	-0.10308	H−S	-0.14783	S−S	-0.09677
C=C	-0.28182	C=N	-0.25265	C=O	-0.28007
C=P	-0.19184	C=S	-0.20381	N=N	-0.19511
N=O	-0.18116	N=P	-0.16692	N=S	-0.14236
O=O	-0.17967	O=P	-0.20583	O=S	-0.18580
P=P	-0.12738	P=S	-0.14290	S=S	-0.15083
C≡C	-0.41714	C≡N	-0.37482	C≡P	-0.27955
N≡N	-0.34502	N≡P	-0.21302	P≡P	-0.16750

Table S7: Canonical SMILES strings for the molecules in the MMFF94 data set used for testing.

CSS
CN=C
CCNO
OC=C
FC=C
CN=O
CPCl
CONOC
FC1CC1
NC#N
NC1CC1
OC=O
c1ncco1
C=COC=O
C=CCC=O
[S-]C=C
CC(=P)C
OC1CNC1
ClC1CCC1
C[NH+]=C
[NH-]C=C
P1PP1
COC(=S)N
C[NH2+]N
O=CC(=O)O
Sc1ccccc1
CC(=S=O)C
CN(F)C
[O-]C=O
NC(=C)C#N
[n-]1nncn1
C1CNNS1
CN1CCN1
NCC(=O)O
c1cnnnc1
NNC(=NN)NN
[O-]C=CC=O
NNC(C)(C)C
CC(=[OH+])C
OS(=O)(=O)O
CC1NCNS1
NC=[NH+]O
C[NH2+]CC#N
FP(OCCC#N)F
[S-]c1nnns1
[S-]C(=S)OC
CC(=[OH+])O
[O-][NH2+]CC
ClC1CSSN1
ONS(=O)(=O)C
O=CN(C=O)C=O
OC1=C(C1=O)O
[O-]c1ccccn1
ON=CC(=NO)Cl
[S-](=S)(=O)C
CSC1=NNC(S1)C
NC(=O)C1CC1=C
c1ccc2c(c1)C2
CC[NH+](C)[O-]
CNP(=O)(C)[O-]
CSC(=S)N=C(N)N
CSC(=[NH2+])NN
NNC(=[NH+]N)NN
ONC(=O)C(=O)NO
COc1nnc(nn1)OC
c1nc2c(s1)ncs2
OC1=CC(=O)C1=O
[O-]C(F)(F)F
Nn1cnc2c1cccc2
CC(=[N+]=[N-])C
C[N-]S(=O)(=O)C
N1CN2C(C1)C2
[S-]C(=S)NC=O
NC(=[N+](C)C)Cl
O=C(N1CC1)N1CC1
ON=C1Cc2c1cccc2
Nc1[nH+]nc(s1)N
BrN1C(=O)CCC1=O
CCCC[n+]1ccccc1

Continued on next page

CN(C(=P)N(C)C)C
NC(=S(=O)=O)N
CNN=Nc1ccc(cc1)C
C[n+]1c(NC)nsc1N
CCOC(=O)c1ncsc1N
COC(=O)C(=O)OC
OCN1N=C(C)N=NC1C
CC(=NC(=S)[S-])N
OCCNC(=S)c1ccco1
N#CC1C(C1C#N)C#N
CCCC1CCOC(S1=O)C
O=Nc1cc(C)ccc1NC
Cc1noc2c1c(N)sn2
N#CC=CS(=O)(=O)C
s1cnc(c1)c1ncsc1
c1csc(n1)c1nccs1
Nn1nc2c(n1)cccc2
CSC(=O)SSSC(=O)SC
NC1=C(CCC1)C(=S)S
S=CN(c1ccccc1)C=S
ON=C(c1ccccc1)C#N
Clp1oc2c(o1)cccc2
CNC(=O)c1noc(n1)C
CC1=NC(=NC(N1)N)N
CC=C1S(=O)CCCS1=O
N#CC1(C)CC1(C)C#N
ClP1Nc2c(S1)cccc2
C#COC(=O)c1ccccc1
OC(=O)C(=O)[O-]
N#CCP(CC#N)CC#N
CNNc1ncnc2c1cccn2
N#CC1CC2C1C(=O)OC2
Cn1c(=O)sc2c1cccc2
CSC(=S)SCCSC(=S)SC
CNC1=[NH+]CCSC1=NO
NC(=O)NC(=[NH2+])N
NC(=O)N1CCS1(=O)=O
O=C1C(=O)C(=O)C1=O
[O-][N+](=O)[O-]
[O-][n+]1onc(c1N)C
COC(=NN=C(OC)OC)OC
CN1CC#CCN(CC#CC1)C
CCN(C(=S)[S-])CC
O=C1OCC1=Cc1ccccc1
Cn1cnc2c1c(=O)cns2
O1C(C1C1OC1)C1OC1
Clc1cnn(n1)c1ccccc1
COC(=N)c1nc(ns1)C#N
O=Cc1c[nH]c2c1cccc2
COP(=O)(C1SCSCS1)OC
OC1C2CCC2Oc2c1cccc2
O=Cc1ccccc1OCC(=O)O
CN1CC[N+](P1Cl)(C)C
CSC(=C(C#N)C#N)[O-]
NC(=[NH2+])c1ccccc1
s1cc2c(c1)sc1c2csc1
CN(C(=PC(F)(F)F)F)C
COC1(C#N)CC1(OC)C#N
Brc1cn2c(c1)C=CC2=O
N#Cc1snc2c1SSSSS2
Clc1ccc2c(c1)ssc2=S
[O-][N+](=O)C(=NO)C
[N-]=[N+]=Nc1nsnc1N
c1[nH]nc(n1)n1cnnc1
NNS(=O)(=O)c1ccccc1
N#CC(N=NC(=O)N)(C)C
CN1S(=O)C(S1=O)(F)F
[O-]C(=O)C(=O)[O-]
s1nc2c(n1)ccc1c2nsn1
OC1CC2CC(C1)OC(=O)O2
NNC(=S)Nc1ccc(cc1)Cl
[O-][N+](=O)c1nonc1N
CCN1N=Nc2c(C1O)cccc2
[O-]C(=O)C=CC(=O)O
[S-]C(=S)NNC(=S)[S-]
CSc1nc(N)oc(=O)c1C#N
COc1ccc(cc1)C=[NH+]O
O=C1C=CN(C1)c1ccccc1
S1CCSCc2ccc(CSCC1)s2
c1ccc2c(c1)P1CCP2CC1
Clc1nc2c(s1)c(N)ncn2

Continued on next page

N#Cc1ccsc1n1cccc1C=O
Nn1ccc(n1)c1ccccc1
CC=C=CC1(C)CCCCC1=NO
N#CC(c1ccc(cc1)N)C#N
CCOCc1c[nH+]c(nc1N)C
ClCP1(=O)COc2c1cccc2
CC1=N[NH2+]C(C1)(C)C
[O-]NC(=O)C(=O)N[O-]
[O-]C(=O)CC(=O)[O-]
C#CC(C(C)(C)C)OS(=O)C
OCC12OC3C2N(CC1Br)CC3
N#Cc1occ2c1cc1OCOc1c2
O=c1[nH][nH]c2c1cccc2
COc1nc(OC)[nH]c(=O)n1
[S-]C(=S)NC(=[NH2+])C
CCCSc1nc2NCCn2c(=S)n1
CCC(N(C(=O)C)O)C(=O)O
Cc1nc(c(s1)c1ccccc1)O
O=CC1=COC2OC1OC=C2C=O
COc1nc(N)nc2c1ncn2C
O=NN1CCN(c2c1non2)N=O
O=C(c1ccccc1)Sc1nnns1
Oc1cccc2c1c1c2cccc1O
CN(Cc1c[nH]c2c1cccc2)C
[S-]C(=S)NCCNC(=S)[S-]
OC(=O)CCC(=O)C(=O)[O-]
CC(N=CC=NC(C)(C)C)(C)C
NN1C(=O)c2c(C1=O)cccc2
CSC(=NO)c1cccc[n+]1C
CC1(O)CCC(P1(=O)O)(C)O
O=c1cc(Cl)c2c(o1)cccc2
[O-][n+]1oncc1c1ccccc1
Cn1ccc(=C(C)C)c(=O)cn1
N#CSC(C(SC#N)(F)F)(F)F
C1N2CN3CN1C[NH+](C2)C3
O=S1c2ccccc2C(N1O)(C)C
Cc1ccc(cc1)S(=O)(=O)Cl
CN(C(C(=S)[S-])N(C)C)C
OOC1NC(=O)NC(=O)C1(C)O
O=C1N(NC1(C)C)c1ccccc1
ON(S(=O)(=O)C)c1ccccc1
COc1csc(c1)c1scc(c1)OC
COC1OCC2C(C1OP(=O)O2)O
c1[nH+]cc2c(c1)cn[nH]2
CC(=Nc1ccccc1O)c1cccs1
ON=C(c1ccccn1)c1ccccc1
O=S1(=O)NCc2c(C1)cccc2
[O-]P(=O)C[n+]1ccccc1N
CC(=O)c1oc2c(c1N)cccc2
CON=C(c1csc(n1)N)C(=O)N
N#CN=[N+](c1ccccn1)[O-]
C[N+]1(C)CC[N+](C1)(C)C
C=C1NC(=O)C(=C1C(=O)C)N
CC(=O)C1=C(N)C(=N)OC1=C
CN(C(=O)c1cccc(=O)n1O)C
S=c1cc(n(n1c1ccccc1)C)C
CC(C1C(=O)NC1c1ccccc1)O
COC(=O)C(C(C1CC=NO1)O)O
CCS(=O)(=O)NS(=O)(=O)CC
COC1C(OC)OCC2C1OC(=S)O2
C=C=C1C(NC1=O)Cc1ccccc1
[O-]C(=O)C1C2C1CCCCC=C2
C[n+]1cnc(c2c1nc[nH]2)N
CC1=Cc2c(S1(=O)=O)cccc2
ClC1(Cl)N2CCCC1(Cl)CCC2
[S-]C(=S)c1n(C)cc[n+]1C
COc1cc(C#CC(=O)O)ccc1OC
COc1oc(nc1C#N)Nc1ccccc1
CNC(=O)C1(CN1Cl)C(=O)OC
CN(C=CC=CC=[N+](C)C)C
COC(=O)c1ccccc1S(=O)C
ClSc1ccccc1[N+](=O)[O-]
Cc1oc2c(c1)oc(=O)c(c2)C
O=C(c1ccccc1)NS(=O)(=O)C
N#Cc1cnc2n3c1ncnc3ccc2
CCOC(=O)c1c(C)nn2c1nnn2C
O=C1c2nsnc2C(=O)c2c1nsn2
CC(=NO)C(=NO)N1CC1C(=O)N
S=C1N=NC(N1c1ccccc1)(C)C
CCC1C2CC(S1(=O)=O)C1C2O1
FC1(F)C2C1CC1C(C2)C1(F)F

Continued on next page

CN(c1oc2c([n+]1C)cccc2)C
c1ccc2c(n1)Sc1c(O2)nccn1
[O-][n+]1onc(c1)c1ccccc1
O=S1(=O)CC2C(C1)C1C2C=C1
CC(=O)N1C=CS(=O)(=O)C=C1
O=S(=O)(n1cccc1)c1ccccc1
O=C1NC(=O)NP(=O)(N1)[S-]
ON=C(c1no[n+](c1C)[O-])C
SCCN1C(=S)NC2(C1=O)CCCC2
S=C(NNC(=S)[OH2+])[OH2+]
COc1n(C)cnc1[N+](=O)[O-]
S=c1scc([nH]1)c1ccccc1
OCC1CC(C1)([NH3+])C(=O)O
CCCCNC(=O)Nc1nsc2c1cccc2
[O-]C(=O)C1([NH3+])CC1
COc1ccc(cc1)C(=O)c1cocc1
CS(=O)(=O)On1nnc2c1cccc2
[O-]S(=O)(=O)[n+]1ccccc1
BrC1=Cc2c(S1(=O)=O)cccc2
Oc1ccccc1C(=S)N1CCCCC1
Cc1cccc(c1C)n1c(C)csc1=S
CNC(=O)ON(C(=O)NC)C(=O)C
[NH3+]NS(=O)(=O)c1ccccc1
ONC=C1C(=O)CC(CC1=O)(C)C
CCCCCCCSc1ncnc2c1nc[nH]2
O=c1[nH]cnc2c1c1CCCCc1s2
CSC(=S)C1=C(SC)SC1(SC)SC
CN(c1cc[n+](cc1)C(=O)C)C
N#Cc1ccc2c(n1)c1ccccc1o2
BrCC1(CC1(Cl)Cl)c1ccccc1
NS(=O)(=O)Cc1noc2c1cccc2
[O-]C(=O)C(C(C(=O)O)O)O
CC(=O)Oc1c(Cl)ccc(c1Cl)Cl
CCSC(=O)CNC(=O)c1ccccc1
N#CC(=C(N(C)C)SC)c1ccccc1
CCOC(=O)C1(CN1OC)C(=O)N
CS(=O)(=O)N1OC1c1ccccc1
CC(C1OP(=O)(Cl)OCC1(C)C)C
CC(N1CCCOP1(=O)F)c1ccccc1
O=P1(OCCCO1)OP1(=O)OCCCO1
O=CC1=CCC2C1C(=O)C2(Cl)Cl
CC(=O)C1=CCCC2[NH2+]C1CC2
CN1CCOC2(C1OCCN2C)c1ccco1
Cc1c(C=Cc2nccnc2)ssc1=S
CC(P1NNP(C1)C(C)(C)C)(C)C
CSC(=[N+](C)[O-])c1ccccc1
CC1OP(=S)(c2c1cccc2)N(C)C
O=C1c2ccccc2NS(=O)(=O)N1C
CNC(=O)C12CC(C1)CN2C(=O)C
[O-][N+](=O)C(=C(SC#N)C)C
Clc1nn2ccnc2c2c1N=NC2(C)C
CN(C1(C#N)CC1(C#N)N(C)C)C
Cc1ccc(cc1)S(=O)(=O)N(C)C
COC(=O)Cc1cc(=O)nc2n1ccs2
CCn1c[nH+]cc1[N+](=O)[O-]
[O-][n+]1onc2c1ccc1n2nnn1
CC#COS(=O)(=O)c1ccc(cc1)C
COSc1ccccc1[N+](=O)[O-]
c1ccc(cc1)CC1=[NH+]CCN1
c1ccc(cc1)n1nc2c(n1)nccn2
COC1OC(C2C1OC(O2)(C)C)C=O
C=C1C2C3C2C(=C)C2C1C2C3=C
CCOC(=O)c1cc2c([nH]1)cco2
c1cc[nH]c(c1)c1cccc[nH]1
c1ccc2c(c1)Sc1c(N2)cccc1
CN1CCOSC1=NC(=O)c1ccccc1
O=S1(=O)CC=C(C1)c1ccccc1
OC(=O)CCC(=NNC(=S)N)C(=O)O
N#CC1=CC=C(N=C(C=C1)OC)C=C
O=C1C2OC2C(c2c1cccc2C)(C)O
O=CNC(=O)c1ccn2c1NC(=O)CC2
CC1=CC(=O)c2c(C1=O)n(C)nc2
O=C(c1ccc(cc1)Cl)CC1CCC1
CC(=NO)C(=NO)n1cccc(c1=O)C
O=c1n(C)c(=O)n2n1C1C=CC2C1
CN(S(=O)(=O)C)S(=O)(=O)C
c1ccn2c(c1)nc1c2n2ccccc2n1
ON(c1ccc(cc1)C(=O)C)C(=O)C
CC(CC1NC(=O)C2N(C1=O)CC2)C
CSc1nn(c(n1)N)c1c(C)cccc1C
CC(=O)OC1(C)C2C1C=C(C2=O)C

Continued on next page

Table S7 – *Continued from previous page*

OC(=O)Cn1cc[n+](c1)CC(=O)O
CC1(OC1c1ccccc1)S(=O)(=O)C
COC1=C(C1(C)C)c1ccc(cc1)Cl
CN(Sc1ccccc1[N+](=O)[O-])C
[NH3+]CC(P(=O)(O)[O-])(F)F
OC(=O)Cc1c[nH]c2c1cc(O)cc2
O=C1N2c3ccccc3SC2(C1(C)C)C
N#CC(=CN1OCC=CC1)SC(C)(C)C
CCOC(=O)Nc1ncc(o1)c1ccccc1
O=Nc1c(N)n(C)c(=O)n(c1=O)C
CC(OP(=S)(NC(=S)C)OC(C)C)C
CCCN1CCC=C2C1CCc1c2nc(s1)N
N#CC(=c1sc(=CC(=O)C)s1)C#N
S=C1[N-]S(=O)(=O)c2c1cccc2
CC1(C)N=C(SC1=S=O)c1ccccc1
[O-][Cl+3]([O-])([O-])[O-]
CC1CC2C=CC(S(=O)N2C(=O)O1)C
N#CN=C1C=CC(=NC#N)c2c1cccc2
c1ccc2c(c1)c1nc3nnnn3nc1cc2
CN(c1[nH]cnc2c1[nH]c[nH]2)C
NC(=O)C1(CN1c1ccccc1)C(=O)N
COc1cc(OC)cc2c1cc(o2)C(=O)C
CN(c1nnn(c(=O)n1)c1ccccc1)C
ClCCn1nnc2n(c1=O)cnc2C(=O)N
ClC1=C2COC(=O)N2C(=CC=C1)Cl
ON1C23CCCCC3NC3C1(CCCC3)ON2
CON=C(C(Cl)(Cl)Cl)Nc1nonc1N
CC1=NN(P2C1C1C=CC2C1)C(=O)C
OCC1OC(C(C1O)O)[N+](=O)[O-]
O=c1[nH]c[nH+]c2c1c[nH]n2
S=C1NC(C2(N1CCS2)N(C)C)(C)C
CCC(C1=NCCS1)(NC(=S)N(C)C)C
CCOC(=O)C1=C(N)C(N=C1C)(C)O
O=C1OCCN1S(=O)(=O)Nc1ccccc1
O=C(C(=O)N)NSSSNC(=O)C(=O)N
c1ccc2c(n1)ccc1c2n2cccc2cn1
[O-]C(=O)C(C(=O)O)Cc1ccccc1
COP(=O)(C(=NO)c1ccccc1)[O-]
CC(=C)C=C=C1CC(O)C2C(C1O)O2
COC(=C(C#N)N=Cc1ccc(cc1)O)N
N#CN([N+](=O)[O-])Cc1ccccc1
CC(=O)OCC1OCN2C(C1)CC(=O)O2
S=C(n1nc(cc1C)C)n1nc(cc1C)C
CC1=C(C)OP(=O)(O1)n1ncnc1
C1CN2C(=NC(C2)c2ccccc2)S1
NC(=S)NN=C(C(=O)O)C(=O)[O-]
NC(=O)c1csc(n1)C1OC(C=C1)CO
CN1c2ncn(c2C(=NS1(=O)=O)N)C
Oc1nc2[nH+]cc(c(=O)n2cc1C)C
O=C1CC(C)(C)Nc2n1c1ccccc1n2
Brc1ccc(c(c1)C=Nc1ccccn1)O
CCSC(=S)C(NC(=O)c1ccccc1)C
CP1N2CCCN1CCN1P(N(CC2)CCC1)C
ClC(S(=O)(=O)C)C(=O)c1ccccc1
CC1C(C)(C)P(=S)(C1(C)C)N(C)C
CN=C1SCC2N1C(SC2)c1nc[nH]c1C
CN1C=C(C(=O)N)C(=O)N2C1CCCC2
FC12C3CC(C2(C3CC1(F)F)F)(F)F
OCC1C=CC(O1)n1cnc2c1ncnc2N
[O-]C(=C([N+](=O)[O-])Cl)OCC
[S-]C(=S)c1ccc(cc1)C(=S)[S-]
O=c1[nH]c(=O)c2c(n1C)n[nH]n2
O=C1CC(N1S(=O)(=O)C)c1ccccc1
ClN=C1NC(=O)N(C(C1(Cl)Cl)O)C
CC1OC(=O)N=S(=O)(C1)c1ccccc1
N#CC(=Cc1ccccc1)[N+](=O)[O-]
O=C1Oc2c(=C1C)n1c(=CCCC1)c2C
CN1CCP2C1(C)P1C2(C)N(CC1)C
CC(P(=O)(O)O)(P(=O)(O)[O-])O
COc1cccc2c1C(O)[N+](C2C)(C)C
NC(=O)C1=CP(=O)(OC1(C)C)[O-]
Cc1n[n+]([O-])nc(c1)c1ccccc1
CCOP1(=O)NC(=NC#N)N(C1(C)C)C
O=c1nc2n(c(=O)n1C)ccc(=O)n2C
NC=[NH+]CCSc1ccccc1C(=O)[O-]
O=[S-](=O)C([N+](CC)(CC)CC)F
CC(=O)N=c1sc(nn1C)S(=O)(=O)N
COc1cc(ccc1O)C(=O)c1ncc[nH]1
O=S=C(C12CCC(C2(C)C)CC1=O)Cl
c1ccc2c(c1)nc1c(n2)C2C3C1C23
OC(=O)CC1(CC1(Br)Br)c1ccccc1

Continued on next page

Table S7 – *Continued from previous page*

[O-]C(=O)C(CCC[NH+]=C(N)N)N
OC1OCc2c1c([O-])c([nH+]c2)C
O=c1[nH]c(=O)n(cc1F)C1CCCO1
CNS(=O)(=O)c1ccccc1c1nnc(s1)N
CCOc1[nH]c(=O)c2c(c1C#N)CCCC2
[O-][N+]1(CCCCC1)CCN1CCCCC1
OC(=O)CCSSCC(C(=O)[O-])[NH3+]
CS(=O)(=O)C1=NC=CC2CC1C=CC=C2
O=C1C=CC2=NC(C=C(C2=C1)C)(C)C
OC1OC(CS(=O)(=O)O)C(C(C1N)O)O
OCC1OC(=CC(C1O)O)[N+](=O)[O-]
CNc1nc[n+](c2c1[nH]c(=O)n2C)C
CS(=O)C12OC2C(CC(C1)(C)C)(C)C
N#Cc1cc(c2ccncc2)c([nH]c1=O)C
Cc1ccc(cc1)c1sc(=O)n2c1SCC2=O
O=COC1C2CC3C1C1C(C2)C3C(=O)C1
COC=C(S(=O)(=O)C(=COC)C#N)C#N
[O-][N+](=O)c1ccc(c2c1non2)Cl
OCCc1sc[n+](c1C)Cc1cnc(nc1N)C
COC(=O)C12C3C2(C1C3=O)C(=O)OC
O=P1(Nc2c(N1)cccc2)c1ccccc1
Clc1c2CSCc3n2n(c1=O)c(=O)c3Cl
ClS(=O)(=O)N=C1OC2C=CC3C1C3C2
FC(C(=O)[O-])CC(C(=O)O)[NH3+]
[O-]C(=O)C(=NNC(=S)N)c1ccccc1
COP(=O)(C1NC(SC1(C)C)C(C)C)OC
[O-]C(=O)c1c[nH]c(=O)[nH]c1=O
c1ccc2c(c1)[nH]c1c2sc2c1cccc2
[O-][N+](=O)C1(C(=O)O)C(C1C)C
Cc1nn(c(c1)C)c1nc2c(s1)cccc2
OCC1OC2SC(C#N)C(OC2C(C1O)O)C
O=C1NCCN1c1ncc(s1)[N+](=O)[O-]
[O-]N=C(c1ccncc1O)[N+](=O)[O-]
COC(=O)C1(Cl)CC1(C#N)SC(C)(C)C
O=C1CCC(=O)OCC2=CCN3C2C(O1)CC3
CON(S(=O)(=O)C)N(S(=O)(=O)C)OC
[N-]=[N+]=Nc1nn2nnnc2c2c1cccc2
[O-]C(=O)C1c2onc(c2CC[NH2+]1)O
NC(=O)c1ccc(cc1)NN=[N+](C)[O-]
O=C(C(C)(C)C)OC1NC(=O)C1=C(C)C
CSC(=C[N+](=O)[O-])Nc1ccccc1Cl
CC(=O)N1N=C(SC1c1ccccc1)S(=O)C
[O-]C(=O)C(CCS(=O)(=N)C)[NH3+]
O=C1NS(=O)(=O)OC(=C1C)c1ccccc1
C1CC1C[NH+]1CC[NH+](CC1)CC1CC1
OC(C(Cl)(Cl)Cl)ON=C(c1ccccc1)N
Nc1nc2[nH]cnc2c(n1)[n+]1ccccc1
CN(c1cccc2c1c(ccc2)[NH+](C)C)C
O=c1[nH]c(=O)n2n1c(=O)[nH]c2=O
O=[S-](=S)C12CC3CC(C2)CC(C1)C3
COc1c2oc(=O)cc2c(c(c(c1F)F)F)F
O=C1C(Cl)(Cl)C(C21CC2)(C)C1CC1
[O-][N+](=O)c1c(N)[nH][nH]c1=O
CC(C1NS(=O)C2C1C(=O)N(C2=O)C)C
[S-]C(=S)[N-]S(=O)(=O)c1ccccc1
CCN(P1(=S)N(C)C(=S)N(C1=C)C)CC
O=C1OCCC1=C1C2(CC2)C(=O)C21CC2
O=C1C(=O)C2(C3(C1(C)C23C)C)C
OCC1OC(C(C1O)O)n1c(N)nc(=O)n1C
[C-]#[N+]c1ccc(cc1)[N+]#[C-]
Cc1ccc2c(c1)CN1CN2Cc2c1ccc(c2)C
Nc1c(C)[nH+]c2n1ccnc2OCc1ccccc1
COc1ccc(cc1)Nc1nn2nnnc2[nH]c1=O
CC(=O)OC1C(C)(C)C2C1(O)C(=CC2)C
CN(C(=[OH+])CCCC(=[OH+])N(C)C)C
O=C1[N-]S(=O)(=O)c2c1c(O)ccc2
CC(C(C(=O)[O-])Sc1ccccc1)[NH3+]
[O-][N+](=O)c1cccnc1Sc1n[nH]cn1
[O-]P(=O)(c1ncc[nH]1)c1ncc[nH]1
[O-][N+](=O)c1nccn1CC(CN1CC1C)O
OCC1C(N1S(=O)(=O)c1ccc(cc1)C)CO
[N-]=[N+]=C([N+](=O)[O-])C(=O)O
O=S1(=O)CCS(=O)(=O)C=C1c1ccccc1
N#CC1(C=C1c1ccccc1)[N+](=O)[O-]
[O-]c1on[n+]2c1C(C)(O)c1c2cccc1
OCC1OC(C(C1O)O)c1nnc2n1nccc2N
ClCC1=CS(=O)(=O)N=C(O1)N1CCOCC1
c1coc(c1)CNc1nc[nH+]c2c1[nH]cn2
O=C1SCC1NS(=O)(=O)c1ccc(cc1)Br
COC(=O)C1(CC1Cl)NC(=O)c1ccccc1
CC(=O)C12CC2(C(=O)C)C(=O)CC1(C)O

Continued on next page

Table S7 – *Continued from previous page*

Cn1cnc(c1Sc1ncccc1O)[N+](=O)[O-]
O=C1NC(=O)C2(C3N1CCCN1C3C2N=C1)C
Clc1cc(Cl)c2c(c1)C(C)(Cl)C(=O)N2
OCC1OC2C(C1O)Oc1n2c(C)cc(=O)n1
CCOc1ccc(cc1)C1(CC1(Cl)Cl)C(=O)O
[O-][N+](=NO)CC([NH3+])C(=O)[O-]
CC1=CC([N+](=[N+]1[O-])[O-])(C)C
COC1(COc2c(O1)cccc2)C1=[NH+]CCN1
O=S1(=O)CC2C=CC3C(N1c1ccccc31)N2
OC1CC2C(C1O)C(C2=O)N1C(=O)CCC1=O
N#CC(C(c1cn(C)cc(c1=O)C)C)C(=S)N
O=c1sc(=O)c2c1sc1c(=O)sc(=O)c1s2
COC(=O)C12CCC(C(C1)OC2=O)(Br)C#N
Nc1[nH]c(=O)c2c(n1)[nH]c[nH+]2
N#CC12CC3CC1C(C(S2(=O)=O)(C)C)C3
CSC(=NC(=N[N+](=O)[O-])N)N1CCCC1
NC(=S)C(=N)SSC(C(F)(F)F)C(F)(F)F
[O-]C(=O)c1[nH+]cccc1C(=O)[O-]
OCC1OC(CC1O)n1ncc2c1[nH]ccc2=O
Cc1cc(C)c(c(c1)C[N+](C)(C)[O-])O
N#CC(=c1[nH]nnn1c1ccccc1)C(=O)OC
CCP1C([NH+](C)C)P(C1[NH+](C)C)CC
CC(Nc1[nH+]c2ccc(cc2n2c1nnc2)F)C
CSCCC(C(=O)NC(C(=O)O)C(C)C)NC=O
O=P(c1ccccc1)(c1ccccc1)c1ccccc1
COC(=O)C1C2C(C1C12OCCO1)C(=O)OC
BrC1=CC(=O)C2(C(C1=O)C1C=CC2C1)Br
Nc1ccc(cc1)S(=O)(=O)c1ccc(cc1)N
Cc1ccc(cc1)N[N+]1=NC(=NC1=O)N(C)C
O=C1[N-]C(=O)C2(O1)C(=O)NC(=O)N2C
CCn1nc(C(=O)O)c(=O)c2c1cc1OCOc1c2
Cc1ccc(cc1)[n+]1noc(c1C(=O)C)[O-]
[O-][N+](=O)c1cc2c(o1)c1CCCCc1cc2
OCC1OC(C(C1O)O)n1cnc2c1ncn(c2=N)C
Nc1nc[nH+]c2c1[nH]nc2C1OC(CC1O)CO
[NH3+]CCC[NH2+]CCSP(=O)([O-])[O-]
ClCCN(P1(=O)OCCC(N1)c1cccnc1)CCCl
[O-][N+](=O)n1cnc(c1)[N+](=O)[O-]
O=S1OC(C(C(O1)C(C)(C)C)C)C(C)(C)C
Nc1ccc(cc1)S(=O)(=O)Nc1noc(c1)C
ClC1(Cl)C2C1(C)CP(=O)(C2)c1ccccc1
COC(=O)C1(Br)OC2C1OC1C2OC(O1)(C)C
CC(P(=S)(P(=S)(C(C)(C)C)C)C)(C)C
OCC1OC(C(C1O)O)n1cc(c2n1ccn2)C#N
OCC1OC(C(C1O)O)n1ccn2c1c(C#N)cn2
Nc1ccc(cc1)S(=O)(=O)[N-]c1noc(c1)C
CNC1=C([N+](=NC)[O-])C(C1(F)F)(F)F
O=C1C2(CC2)C(=O)C2(C(=O)C31CC3)CC2
O=C(C([N+](=O)[O-])(F)F)N[NH+](C)C
CC(=O)N1N=C(S(=O)C1c1ccccc1)S(=O)C
NN1C2(C)CC3(CC1(C)N(C(O2)(C3)C)N)C
CS(=O)(=O)ON(S(=O)(=O)C)S(=O)(=O)C
[O-]C1=C(Br)C(=O)C(=C(C1=O)Br)[O-]
CN(C(=S)N=c1nc(sc(n1)N(C)C)N(C)C)C
COC(=O)C1=[N+]([O-])c2c(C1=O)cccc2
COc1cccc(c1c1cc(=O)c2c(o1)cccc2)OC
Nc1[nH+]cnc2c1ncn2CCOCP(=O)(O)[O-]
N#CC1N2C(=O)C(C2S(=O)C1(C)C)(Br)Br
OC(C1(CC[NH2+]1)C(=O)[O-])c1ccncc1
BrC1C2c3ccccc3C(C1Br)C(C2Br)(Br)Br
CC(=O)CCn1nc(nc1N1CC1)[N+](=O)[O-]
OCC1OC(C(C1O)O)n1cnc(n1)S(=O)(=O)N
OCC1OC(CC1O)n1cnc2c1nc(N)nc2S(=O)N
CC(P(=S)(SP(=S)(C(C)(C)C)C)C)(C)C
O=S1(=O)OC(=NS(=O)(=O)O1)c1ccccc1
O=C(NCC(=O)[O-])CNC(=O)C([NH3+])C
CC(Cn1c(=O)n(C)c(=O)c2c1nc[nH]2)C
CCN(C(=NC(=O)C(C(=O)Cl)(Cl)Cl)Cl)CC
CC1C(C)(C)OC(=NS1(=O)=O)C(Cl)(Cl)Cl
O=C(c1ccc(cc1)[N+](=O)[O-])N1CCCCO1
N#CC1=C(N)C(CC(C1)(C#N)C#N)(C#N)C#N
CN(c1ccc(cc1)C(=O)c1ccc(cc1)N(C)C)C
[O-]S(=O)(=O)c1[nH]c2c([nH+]1)cccc2
ClC1=NC2(C(C1(Cl)C1C2C2CC1CC2)Cl)Cl
CN(Nc1scc(n1)CSCCC(=NS(=O)(=O)N)N)C
[O-][N+]1=C(c2ncccn2)C(=O)c2c1cccc2
FC(C1(C)OS(=O)(=O)C=C1N1CCCCC1)(F)F
Clc1ccc(cc1)C(=[N+](C)[O-])Cn1ncnc1
N#CC(=c1ccc(=C(C#N)C#N)c2c1nsn2)C#N
CCc1nnc(s1)[N-]S(=O)(=O)c1ccc(cc1)N
CCOC1=NNC(=O)C2C1C1CC2C2C1c1c2cccc1

Continued on next page

Table S7 – *Continued from previous page*

CN(C(=NP1(=O)OC(=C(O1)C)C)N(C)C)C
O=c1[nH]cnc2c1cn(n2)C(C(O)(C)C)(C)C
[N-]=[N+]=CC(=O)C1(C)C(=O)C2C1CCCC2
CCS(=O)(=O)N(S(=O)(=O)CC)S(=O)(=O)CC
OCC1OC(C(C1O)O)n1nc(nc1N)C(=[NH2+])N
CCOC(=O)C(S(=O)(=O)[O-])[N+](=O)[O-]
[O-][n+]1nn(c2c1cccc2)C(=O)OC(C)(C)C
CCOC1=NC23C(N1Cl)(CCCC2)N=C(N3Cl)OCC
O=C1OC2CCC3(C41ON4S(=O)(=O)C3)C2(C)C
CCOC(=NC(=O)OC)Nc1c[nH]c(=O)[nH]c1=O
CSc1c2CCNC3=C(C(=O)C(=O)c(n1C)c23)Cl
[O-][n+]1ccc(cc1)[N+](=Nc1ccccc1)[O-]
O=C(C(Cl)(Cl)Cl)N1C(C)(C)C(S1(=O)=O)C
Cc1n[nH]c2n([nH]1)c(=O)cc(n2)c1ccccc1
CCOC(=O)N=S1(=O)C(=C(C(=C1Cl)Cl)Cl)Cl
O=C1NN=C(C(C1)C)c1ccc(cc1)n1c[nH+]cc1
[N-]=[N+]=Nc1ccc2c(c1)c(N)nc([nH+]2)N
OCC1OC(CC1O)n1ccc(nc1=O)NC(=O)NC(=O)N
FC(C1(SSC(=N1)Nc1ccccc1)C(F)(F)F)(F)F
COC(=S)[N-][N+]1=C(N(C)C)C(NC1=O)(C)C
Clc1nn(c(n1)Cl)c1ccc(cc1)[N+](=O)[O-]
Nc1cc[n+](cc1)Oc1ccc(cn1)[N+](=O)[O-]
[O-][N+](=O)Nc1nnc([n-]1)N[N+](=O)[O-]
BrC=Cc1cn(C2CC(C(O2)CO)O)c(=O)[nH]c1=O
NC(=Nc1scc(n1)CSCCC(=NS(=O)(=O)N)N)N
[S-]P1(Sc2ccccc2)SP(S1)([S-])Sc1ccccc1
Nc1ccc(cc1)S(=O)(=O)[N-]c1nc(C)cc(n1)C
CN(c1ccc(cc1)N(S(=O)(=O)C)S(=O)(=O)C)C
ClC=C(C#CC1=C(Cl)C(=O)C(=C(C1=O)Cl)O)C
OCC1OC(OC2(C#N)CC(C3C2O3)O)C(C(C1O)O)O
CN1NC(C)(C)n2c(C1=O)c(nc2)[N+](=O)[O-]
ClC(=C1C(=C(Cl)Cl)C(C1(Cl)Cl)(Cl)Cl)Cl
N#CC1=C(C#N)SN(S1)S(=O)(=O)c1ccc(cc1)C
COC(=O)C(c1sc(c(c1Cl)Cl)Cl)(C(=O)OC)Cl
O=S(=O)(c1ccccc1)SSS(=O)(=O)c1ccccc1
N#CC(=C1C(=C(C#N)C#N)C1=C(C#N)C#N)C#N
NC1=[N+](c2ccc(cc2)Cl)C(NC(=N1)N)(C)C
N#Cc1nsc(n1)C(=NSN=C(c1snc(n1)C#N)Cl)Cl
[O-][N+](=O)N1CCN(c2c1non2)[N+](=O)[O-]
CCC(P1(=O)N(C)C2C(N1C)CCCC2)N=[N+]=[N-]
FC(C1=C(OCCOC1(F)F)C(C(F)(F)F)(F)F)(F)F
ClC1C2CC3C1C1(C3(C2(Cl)C(=C1Cl)Cl)Cl)Cl
[C-]#[N+]CCCN(CCC[N+]#[C-])CCC[N+]#[C-]
[O-]C(=O)C1=C(CC2N1C(=O)C2C(O)C)SCCNC=N
COc1cc(=NS(=O)(=O)c2ccc(cc2)N)[nH]c(n1)C
OC(=O)C1=C(c2ccccc2)C(=O)C(=Cc2ccccc2)O1
N#CC(=c1c2nsnc2c(=C(C#N)C#N)c2c1nsn2)C#N
NC(=NS(=O)(=O)N)CCSCc1csc(n1)[NH+]=C(N)N
Cc1[nH]n(c2=NS(=O)(=O)C(=Nc12)c1ccccc1)C
O=NN1CN(CCN(C1)[N+](=O)[O-])[N+](=O)[O-]
OC(=O)C1N2C(=O)CC2S(=O)(=O)C1(C)Cn1ccnn1
ClC(=C1C(Cl)(Cl)C(C(C21OC2Cl)(Cl)Cl)O)Cl
NC(=[NH+]N=C(C1=C(C)[N+](=C(N)N)N=C1)C)N
[O-]C(=O)C[n+]1ncn(c1)N=C(Cc1ccccc1)[O-]
[O-][N+](=O)c1cc(cc2c1n1ccccc1n2)C(F)(F)F
O=C1C(=Cc2cccc(c2)[N+](=O)[O-])N=C2N1CCS2
[O-][NH+]=C1C(=C(CP1(=O)OC)[N+](=O)[O-])C
[O-][N+](=O)c1cc(ccc1C(=O)O)[N+](=O)[O-]
O=c1n(CC2OC2)c(=O)n(c(=O)n1CC1OC1)CC1OC1
[O-]S(=O)(=O)SCC([NH+](C)C)CSS(=O)(=O)[O-]
CN([N+](=O)[O-])CC1(COC1)CN([N+](=O)[O-])C
CN(P1([O-])NN(C)P(N(N1)C)([S-])Oc1ccccc1)C
ClC(=C1SC(OC(S1)C(Cl)(Cl)Cl)C(Cl)(Cl)Cl)Cl
CC(=O)OC1C2C(OC(=O)C)C3C(C1N1N2N31)OC(=O)C
OC1C(O)C(OC1n1cnc(n1)C(=S)N)COS(=O)(=O)N
Oc1c(O)cc[n+](c1c1[n+]([O-])ccc(c1O)O)[O-]
OCC1OC(CC1O)n1cc(c(=O)[nH]c1=O)C1CC1(Br)Br
Clc1nc(nn1C(C(Cl)(Cl)Cl)(Cl)Cl)C(Cl)(Cl)Cl
Cn1nn[n+](c1C(C(C)(C)C)N=N[N-]S(=O)(=O)C)C
O=[S-](=O)C(SC([N+]12CCC(CC1)CC2)(F)F)(F)F
[O-]C(=N[N+]1=C(N(C)C)C(NC1=O)(C)C)C(F)(F)F
[O-][N+](=O)C1OC1C1OC(OC1C1COC(O1)(C)C)(C)C
OCC1OC(C(C1O)O)n1c(=O)n(c2c1nc(N)[nH]c2=O)C
OCC1OC(C(C1O)O)n1cnc2c1nc(N)nc2S(=O)(=O)N
[O-][N+](=O)c1cnn(c1)Cn1ncc(c1)[N+](=O)[O-]
O=C1C2C3C4C1C1C(C2C(C3)C41)(Br)[N+](=O)[O-]
Oc1ccc2c(c1)Oc1c(C32OC(=O)c2c3cccc2)ccc(c1)O
CN(C(=[N+](C)C)OC(=CC(=O)c1ccccc1)c1ccccc1)C
CON=C(C(=O)n1n[n+](c2c1cccc2)[O-])c1csc(n1)N
OCCNC(=O)c1c(C)[n+]([O-])c2c([n+]1[O-])cccc2
[O-][N+](=O)c1ccc2c(c1)c(n[n-]2)[N+](=O)[O-]

Continued on next page

Table S7 – *Continued from previous page*

[O-]S(=O)(=O)Oc1ccc2c3c1[N+](C)(C)CCc3c[nH]2
O=C(C1=C([O-])c2ccccc2S(=O)(=O)N1C)Nc1ccccn1
O=S(c1ccccc1[N+](=O)[O-])c1ccccc1[N+](=O)[O-]
CN(C(=[N+](C)C)P(=O)(C(=[N+](C)C)N(C)C)[O-])C
[N-]=[N+]=C1C(=O)N2N(C1=O)CCCC2C(=O)OC(C)(C)C
ClC1=C(Cl)C(=C(S1=NS(=O)(=O)c1ccc(cc1)C)Cl)Cl
OC(=O)CC1N(NC(=O)c2c1cc(Cl)cc2)c1ccc2c(c1)nns2
CC1=CC(=CC(=S)C(C(F)F)(F)F)SC(=C1)C(C(F)F)(F)F
ClC(=CC(C(=O)O)(Cl)C)C(C([N+](=O)[O-])(Cl)Cl)Cl
CC(=O)C(=NNc1ccc(cc1[N+](=O)[O-])[N+](=O)[O-])C
CC(=O)OC1CC2CC1c1c2c[n+](c(c1)[N+](=O)[O-])[O-]
[O-][N+](=O)n1cnc(n1)N=Nc1ncn(n1)[N+](=O)[O-]
OCC1OC(C2C1OC(O2)(C)C)Nc1nc(Cl)nc2c1nc(Cl)nc2Cl
CN1C(=O)N(C)C(=O)C2(C1C1N(C)C(=O)N(C(=O)C21F)C)F
ClC(C1OP2OC(P1C(O2)C(Cl)(Cl)Cl)C(Cl)(Cl)Cl)(Cl)Cl
ClC(C1OC(SC(S1)(Cl)C(Cl)(Cl)Cl)C(Cl)(Cl)Cl)(Cl)Cl
CC(=C([N+](=O)[O-])[N+](=O)[O-])NC(C(Br)(C)C)(C)C
N#CC1=C([S-])NC(=C(C1c1ccccc1[N+](=O)[O-])C(=O)C)C
[N-]=[N+]=C1C=C(C=C(C1=O)[N+](=O)[O-])[N+](=O)[O-]
N#[N+]c1cc([N+](=O)[O-])c(c(c1[O-])[N+](=O)[O-])Cl
[O-][N+](=O)c1cc2n(ncc2c(c1)[N+](=O)[O-])c1nnn[nH]1
O=C1OC(C1NC(=O)c1cccc(c1O)O)Cc1ccc(cc1)[N+](=O)[O-]
N#CC(=C1C(=C(C#N)C#N)C(=C(C#N)C#N)C1=C(C#N)C#N)C#N
[O-][N+](=O)c1cc(sc1S(=O)(=O)c1ccccc1)[N+](=O)[O-]
ClC(SN=C(N(SC(Cl)(Cl)Cl)SC(Cl)(Cl)Cl)c1ccccc1)(Cl)Cl
[O-]C(=O)C1(CC(C1)([NH3+])C(=O)[O-])S(=O)(=O)c1ccccc1
[O-][N+](=Cc1ccc(cc1[N+](=O)[O-])[N+](=O)[O-])C(C)(C)C
N#CC12C(=O)C(=C(C(=O)C2(C21c1ccccc1c1c2cccc1)C#N)Cl)Cl
N#CC1=C(Nn2c(nnc2c2ccccn2)c2ccccn2)C(=O)C(=C(C1=O)Cl)Cl
ClC12C3C4(C(C1(Cl)Cl)(C1C2(C3(Cl)C(C41Cl)(Cl)Cl)Cl)Cl)Cl
CN(C(=O)COC(=O)Cc1ccc(cc1)OC(=O)c1ccc(cc1)NC(=[NH2+])N)C
OC(=O)CN(c1ccc(cc1[N+](=O)[O-])[N+](=O)[O-])[N+](=O)[O-]
Fc1c(F)nc(c(c1[N+](=[N+](c1c(F)c(F)nc(c1F)F)[O-])[O-])F)F
CON=C(C(=O)NC1C(=O)N2C1SCC(=C2C(=O)O)CSc1nnnn1C)c1csc(n1)N
[O-][N+](=O)C1([N+](=O)[O-])C2C3C4C1C1(C2C3C1C4)[N+](=O)[O-]
[O-][N+](=O)N1C(=O)N(C2C1N(C(=O)C)C(=O)N2[N+](=O)[O-])C(=O)C
FN(C(C)(C)C)c1c(cc(cc1[N+](=O)[O-])[N+](=O)[O-])[N+](=O)[O-]
[O-][N+](=O)C1=CC([N+](=O)[O-])(C2CC2)C(C(C1=O)(C)O)[N+](=O)[O-]
COC1C(=C(N2C1C(C(O)C)C2=O)C(=O)OCc1ccc(cc1)[N+](=O)[O-])Sc1ncccn1
[O-][N+](=O)C1(C)C(=C(Br)C(C(C1=O)(C)[N+](=O)[O-])(C)[N+](=O)[O-])Br
COC1OC(CO[N+](=O)[O-])C(C(C1O[N+](=O)[O-])O[N+](=O)[O-])O[N+](=O)[O-]
[O-][N+](=O)c1[nH]c(nc1[N+](=O)[O-])c1nc(c([nH]1)[N+](=O)[O-])[N+](=O)[O-]
CC(=O)NC=CS(=O)C1=C(C(=O)OCc2ccc(cc2)[N+](=O)[O-])N2C(C1)C(=C(COC(=O)C)C)C2=O
[O-][N+](=O)C1=C(C)C(C)([N+](=O)[O-])C(=O)C(C1(C)[N+](=O)[O-])(C)[N+](=O)[O-]
COC(=O)C1ON2OC1c1cc(cc(c1C2([N+](=O)[O-])[N+](=O)[O-])[N+](=O)[O-])[N+](=O)[O-]

Table S8: Canonical SMILES strings for the molecules in the KEGG data set used for testing.

CCO
CCC
NC#N
NCCN
NCCO
CCCC
NCCS
CCCl
SC#N
O=C=O
CCNCC
CCOCC
ClCCl
CCCCO
O1CC1
C1CC1
O=S=O
CC(O)C
CC(N)C
CC(C)C
CC1CO1
CC(=O)O
NC(=O)N
OCC(O)C
CS(=O)C
OCCNCCO
CC(=O)C
[O-]N=O
NCC(=O)O
OCC(CO)O
OCC(CS)S
CC(=O)NO
NC(=O)NO
N1CCNCC1
CCC(=O)O
Nc1nccs1
SCCN(C)C
CC(=O)OO
OCC(CS)O
OCCN(C)C
NNC(=N)N
C1COC1=O
OCCC(O)C
Oc1ccccc1
CC(N)(C)C
O=CCCCC=O
CCOC(=O)C
C=CCN=C=S
Nc1ccncc1
NC1CCCCC1
NCCC(=O)O
OC(=O)CCl
OCC(=O)CO
OCCOCCOCC
NCCC(CN)C
NCCCC(=O)O
OCc1ccccc1
OP(=O)(O)O
NC1CONC1=O
[O-]C(=O)C
[O-]C(=O)O
OCc1cccnc1
NC1CCCCC1N
[O-]S(=O)O
OCC(N)(C)C
CCC(O)(C)C
[NH2-2]C#N
FCC(F)(F)F
c1ncc[nH]1
CCCCCC(N)C
CNC(=S)NCO
OCCN1CCCC1
CC[NH2+]CC
Nc1cnccc1N
CC(C(=O)O)N
[N-]=[N+]=O
OC(=O)C(O)C
COc1ccccc1O
OCCc1ccccc1

Continued on next page

CS(=O)(=O)O
OS(=O)(=O)O
CC(CCON=O)C
Cc1c[nH]nc1
CC(NC(C)C)C
O=Cc1ccccc1
FC(Cl)(Cl)F
CN(CC(O)C)C
[O-]P(=O)=O
OCc1ccccc1O
[O-]CS(=O)O
[CH2-]C(C)C
[O-]C(=O)CN
[O-]C(=O)CC
OCC(C(=O)O)N
NC(C(=O)O)CS
Oc1ccc(cc1)O
Oc1cccc(c1)O
NCCCCCC(=O)O
OCCN(CCO)CCO
OCC(CO)(CO)N
NNCCc1ccccc1
OC(=O)C(=O)O
NC(C(=O)O)CF
CC(CC(=O)N)C
ClCCN(CCCl)C
Cc1cccc(c1)O
CC(CC(=O)C)C
[O-]c1ccccc1
CC1COC(=O)O1
FC(Cl)(Cl)Cl
Cc1ncsc1CCCl
CC(C#C)(CC)O
CNCCc1ccccn1
OCCOc1ccccc1
CCS(=O)(=O)O
OCC(C(CO)O)O
[O-]C(=O)CCC
OC(=O)C1CCCN1
NCCS(=O)(=O)O
Oc1ccc(cc1)Cl
NCC(CC(=O)O)O
Cn1cc[nH]c1=S
[O-][N+](=O)O
[O-]C(=O)[O-]
COC(=O)C(CS)N
OC(=O)CC(=O)O
ClC(C(=O)O)Cl
[O-]P(=O)(O)O
CN(c1nccnc1)C
C=COCC(F)(F)F
COc1ccc(cc1)O
CCCCCNCC(=O)N
OCCS(=O)(=O)O
CCCCCCCC(=O)O
CC(CCCC(N)C)C
OCCSCc1ccncc1
OCCCC(=O)[O-]
CC=CC=CC(=O)O
NCC(CO)(CO)CN
NCCc1c[nH]cn1
OCC(CO)(CO)CO
O=C1CCC(=O)N1
OC(=O)c1cccs1
OC(=O)C1CSCN1
N(c1nncs1)C#N
OCC(NC(CO)C)C
[O-]S(=O)[O-]
CSCCC(C(=O)O)N
NC(=O)c1cccnc1
OC(=O)c1ccccc1
OC(=O)c1cccnc1
NC(=O)c1cnccn1
OC1COC2C1OCC2O
OC(=O)CCC(=O)O
OCC[N+](C)(C)C
CCC(c1ccccc1)O
CCOC(=O)C(CS)N
S=c1ccccn1[O-]
[O-]C(=O)C(O)C
O=Cc1ccccc1C=O

Continued on next page

Table S8 – *Continued from previous page*

CC(=O)NCC(=O)O
CC(Cc1ccccc1)N
NCC1(CN)CCOCC1
CC(CC(O)(C)C)O
[O-]S(=O)(=O)C
NCC1(CN)CCCCC1
FC(C(F)(F)F)Br
NCCCS(=O)(=O)O
OC(=O)C(F)(F)F
CNC(CC1CCCC1)C
OC(=O)c1cccnc1
NC(C(=O)O)CCCN
NC1CC1c1ccccc1
OCC1CNCC(C1O)O
[O-]S(=O)(=O)O
NC(C(=O)O)C(C)C
CC(C(C(=O)O)N)O
OC(=O)c1ccccc1O
Oc1cc(O)cc(c1)O
NNC(=O)c1ccncc1
CCCC(C(=O)O)CCC
NC(C=C)CCC(=O)O
ClC(C(F)(F)F)Br
OC(=O)C=CC(=O)O
CC1OC(C)OC(O1)C
CC1OC1P(=O)(O)O
COS(=O)(=O)[O-]
NC(=O)c1ccccc1O
CNC(Cc1ccccc1)C
NCCCCC(C(=O)O)N
CCCC(C(=O)N)CCC
NCC(=O)CCC(=O)O
COC(=O)C(=C)C#N
COC(=O)c1cccnc1
CCCCCCCCCCCCCCO
Oc1cccc2c1nccc2
CNC(CC1CCCCC1)C
COC(C(Br)F)(F)F
FS(F)(F)(F)(F)F
OC(=O)c1ccccc1O
OC(=O)c1ccccc1S
OC(=O)Cc1ccccc1
N#CCCS(=O)(=O)C
CNC(CCC=C(C)C)C
NC(C(=O)O)CC(C)C
OCC(C(C(CO)O)O)O
CCC(C(C(=O)O)N)C
COc1cc(C=O)ccc1O
OC(C(Cl)(Cl)Cl)O
ON=Cc1cccc[n+]1C
COC(C(Cl)Cl)(F)F
CCC(C#C)(C=CCl)O
[O-][N+](=O)[O-]
COC(=O)c1ccccc1O
NNc1nncc2c1cccc2
SCCS(=O)(=O)[O-]
CCCCC(c1ccccc1)O
OCCCSCC(C(=O)O)N
NC(=O)CN1CCCC1=O
OC(=O)CCCCC(=O)O
CC=Cc1ccc(cc1)OC
[O-]P(=O)(O)[O-]
CC=CC=CC(=O)[O-]
CN1CCCC1c1cccnc1
Oc1ccc(c(c1)C)Cl
O=C1NC(=N)N(C1)C
NC1CCCC1c1ccccc1
Nc1nnnn1c1ccccc1
CCOS(=O)(=O)[O-]
NCC(=O)CCC(=O)OC
ClC(C(=O)[O-])Cl
[O-]P(=O)(F)[O-]
OC1COC(C(C1O)O)O
CC(=O)NC1CCSC1=O
CCNC(Cc1ccccc1)C
[O-][N+](=O)OCCN
CC(CCCC(O)(C)C)N
C1CCn2c(CC1)nnn2
CC([NH2+]C(C)C)C
CN(c1ccc(cc1)O)C
OCNC(=O)c1cccnc1

Continued on next page

Table S8 – *Continued from previous page*

CCOC(=O)c1cccnc1
NC1=NC(=O)N2C1C2
CCCCCCCC(=O)[O-]
c1ccc2c(cc1)ccc2
OC(=O)CC(C(=O)O)N
Nc1ncnc2c1nc[nH]2
NC(=O)CCCCC1SSCC1
OC(=O)CCCCC1SSCC1
NCC(c1ccc(cc1)O)O
Oc1ncnc2c1cn[nH]2
Nc1nc(=O)[nH]cc1F
CCCCNC(=N)NC(=N)N
NCCc1ccc(c(c1)O)O
CN(C(=N)NC(=N)N)C
CCOc1cc(C=O)ccc1O
OC(c1ccccc1)C(N)C
NCC(c1cccc(c1)O)O
CCOc1ccccc1C(=O)N
CNC(Cc1ccccc1OC)C
[O-]S(=O)(=O)[O-]
[O-]C(=O)c1ccccc1
OC(=O)c1ccc(cc1)N
CN(Cc1ccccc1)CC#C
OCC(COc1ccccc1C)O
CCC(C(CC)C)C(=O)N
C=CCC(Cc1ccccc1)N
OC(=O)CC(C(=O)O)O
COC(=O)C=CC(=O)OC
CCON(C(=S)[S-])CC
CC(Cc1ccc(cc1)O)N
CCOC(C(=O)C(O)O)C
OCC1NCC(C(C1O)O)O
CON=CC1=CCCN(C1)C
Cc1nccnc1N1CCCCC1
CC1NCCOC1c1ccccc1
CC(Cc1ccccc1)(N)C
C#CCOC1CN2CCC1CC2
[O-]N=C(C(=NO)C)C
ON=C1CCCCC1=N[O-]
CC(Cc1cccc(c1)O)N
CCC(OC(=O)N)(CC)C
Cn1ccc(=O)c(c1C)O
O=C1CCC(N1)C(=O)O
Oc1ccccc1c1ccccc1
OC(=O)C(Cl)(Cl)Cl
NC(=O)CC(C(=O)O)N
CCOC(=O)C=CC(=O)O
CCCCCCCCCCCC(=O)O
O=c1ccoc(c1[O-])C
OC(=O)CCC(C(=O)O)N
NC(=O)CCC(C(=O)O)N
CNC(C(c1ccccc1)O)C
CC1=CCC(CC1)C(=C)C
CC(=O)Nc1ccc(cc1)O
SCC(C(=O)O)NC(=O)C
OCC(COc1ccccc1OC)O
C1N2CN3CN1CN(C2)C3
NC(C(S)(C)C)C(=O)O
CNCC(c1cccc(c1)O)O
CC1(CC)CC(=O)NC1=O
FC(OC(C(F)(F)F)F)F
[O-]C(=O)c1ccccc1O
CCc1nccc(c1)C(=S)N
OS(=O)(=O)c1ccccc1
CCCC(C(=O)[O-])CCC
OC(c1ccccc1)C(=O)O
[O-]C(=O)c1ccccc1S
NCC1CCC(CC1)C(=O)O
COC(=O)c1ccc(cc1)O
CC(C(=O)NCC(=O)O)S
CC(CCCC(NC(C)C)C)C
OCCOC(=O)c1ccccc1O
[O-]C(=O)C(=O)[O-]
COc1cc(OC)cc(c1)OC
NC(=O)OCCCc1ccccc1
Clc1c[nH]c(=O)cc1O
CCC(=O)c1ccc(cc1)O
OCc1c(CO)cnc(c1O)C
CC(=O)Oc1cccc(c1)O
C#CCNC1CCc2c1cccc2
NCC(CC(=O)O)CC(C)C

Continued on next page

CCCCCCc1ccc(cc1O)O
OC(=O)C[N+](C)(C)C
NOCc1ccc(c(c1)O)Br
Oc1ccc(c2c1CCC2)Cl
FC(C(Cl)(F)F)(Cl)F
O=c1[nH]oc2c1CCNC2
[O-]c1cccc2c1nccc2
NCCc1cc(O)c(cc1O)O
[O-]C(=O)Cc1ccccc1
COCCCP(CCCOC)CCCOC
CNc1n(C)nc2c1CCCC2
CCCCCCC(C(=O)O)CCC
OC(=O)CCCCCNC(=O)C
CNCC(c1ccc(cc1)O)O
NC(=N)NCC1CCCCCCN1
OCCSCCCCCCCCCCSCCO
[O-][Cl](=O)(=O)=O
CCOC(=S)SSC(=S)OCC
NCc1ccc(cc1)C(=O)O
O=c1ccc2c(o1)cccc2
CCCCOC(=O)C(=C)C#N
CCC(NCCNC(CO)CC)CO
CNC(Cc1ccccc1)(C)C
CNC(Cc1ccc(cc1)O)C
ClCCNP(=O)(NCCCl)O
Cc1ncccc1OCC1CCCN1
[O-]C(=O)C=CC(=O)O
OC(C(C1OC1)O)C1OC1
FC1CC(C1)(N)C(=O)O
NC(C(=O)O)Cc1ccccc1
CC1CCC(C(C1)O)C(C)C
OC(=O)CSCC(C(=O)O)N
NCC1(CCCCC1)CC(=O)O
FC(OC(C(Cl)F)(F)F)F
FC(OC(C(F)(F)F)Cl)F
CCOC(=O)c1ccc(cc1)N
CC(COc1c(C)cccc1C)N
c1ccc(cc1)CC1=NCCN1
Cc1ccc(c(c1)O)C(C)C
CCCc1cc(ccn1)C(=S)N
CCNCC(c1cccc(c1)O)O
CCOC(=O)c1ccc(cc1)O
CCCCCCCCCCCCCCCCCO
CC(C(Cl)(Cl)Cl)(O)C
OS(=O)(=O)NC1CCCCC1
OCCOC(=O)NCc1ccccc1
CC(=O)OC1CN2CCC1CC2
[O-]P(=O)([O-])[O-]
NC1=NCC(O1)c1ccccc1
OC(=O)CCCCCCCC(=O)O
NC1CCC(CC1)c1ccccc1
Oc1cc(C)c(c(c1)C)Cl
Clc1ccccc1CC(N)(C)C
Clc1ccc(c2c1cccn2)O
O=C1NC(=O)C(O1)(C)C
CCCCNC(=NCC)NC(=N)N
C=CCc1ccc(c(c1)OC)O
FC(COCC(F)(F)F)(F)F
CCC(NC(=NC#N)N)(C)C
NC(=N)NCCN1CCCCCCC1
OP(=O)(CP(=O)(O)O)O
ClCCCNC(Cc1ccccc1)C
CC(=O)OCC(OC(=O)C)C
ClCCC(=O)NCc1ccccc1
NC(=O)CN1CC(CC1=O)O
CC(Cc1ccccc1)NCCC#N
CCOc1ccccc1OCC(CO)O
CCCC(CC(CC(=O)O)N)C
OCC1OC(O)C(C(C1O)O)O
O=c1cc[nH]c(=O)[nH]1
CC(OP(=O)(OC(C)C)F)C
OCC(C(C(C(CO)O)O)O)O
NCC(c1ccc(c(c1)O)O)O
OC(C(C(=O)O)O)C(=O)O
NC(=O)NC1NC(=O)NC1=O
Nc1ccc(c(c1)O)C(=O)O
NC(C(=O)O)CC[S+](C)C
ClCCNC(=O)N(CCCl)N=O
Nc1ccc(c(c1)C(=O)O)O
O=C1OC(C(=O)N1C)(C)C
CN1CCCN=C1C=Cc1cccs1

Continued on next page

Table S8 – *Continued from previous page*

NC(=O)OCC[N+](C)(C)C
CCOc1ccc(cc1)NC(=O)C
SC(C(C(=O)O)S)C(=O)O
C#CC1(CCCCC1)OC(=O)N
CCC(N1CCCC1=O)C(=O)N
Clc1ccc2c(c1)nc(o2)O
CC(=O)OCC[N+](C)(C)C
CC(C(c1cccc(c1)O)O)N
NC(=N)NCCCC(C(=O)O)N
CCCOC(=O)c1ccc(cc1)O
OC(=O)CC(C(=O)[O-])O
OCC1OC(O)C(C(C1O)O)F
CC1N(C)CCOC1c1ccccc1
CC(NNC(=O)c1ccncc1)C
[O-]C(=O)c1ccc(cc1)N
CC(COC(=O)C(=C)C#N)C
CCCCc1ccc(nc1)C(=O)N
NC=C1Cc2c(C1=O)cccc2
OCC1OC(O)C(C(C1O)O)N
NOCc1c(C)ccc2c1cccc2
CCCCCCCCC=CCCCCCCCCO
CS(=O)CC(=O)c1ccccn1
CCCOC(=O)c1ccc(cc1)N
[O-]C(=O)CCCc1ccccc1
OC(COc1nccs1)CNC(C)C
Clc1ccc(cn1)OCC1CCN1
[O-]N=C(C(=N[O-])C)C
[O-]N=C1CCCCC1=N[O-]
CN(C1Cc2ccccc2OC1C)C
CN(c1nc(C)c(nc1C)C)C
OC1CN2C(C1O)C(O)CCC2
CC(=O)NCCCS(=O)(=O)O
NNc1nnc(c2c1cccc2)NN
Oc1ccc2c(c1)oc(=O)s2
N=C(N1CCOCC1)NC(=N)N
OC(c1ccccc1)COC(=O)N
CCN(C(=O)c1cccnc1)CC
OC(=O)CC(C(=O)[O-])N
[O-]c1ncnc2c1[nH]nc2
[NH3+]CCCCC(C(=O)O)N
CCOC(=O)n1ccn(c1=S)C
NC(=O)NCCCC(C(=O)O)N
Nc1nc(NC2CC2)nc(n1)N
CCCCOCC(COc1ccccc1)O
[O-]C(=O)CCC(=O)[O-]
NN=c1[nH]ncc2c1cccc2
OCCNC(=O)C=Cc1ccccc1
CCCCCOc1ccccc1C(=O)N
Fc1cnc(c(n1)C(=O)N)O
Nc1c2CCCCc2nc2c1CCC2
FC(C(F)(F)F)C(F)(F)F
NCC(c1ccccc1)CC(=O)O
NC(C(=O)O)Cc1c[nH]cn1
CNCC(c1ccc(c(c1)O)O)O
CC(=O)Oc1ccccc1C(=O)O
OCC(C(C(C(=O)CO)O)O)O
S=c1nc[nH]c2c1[nH]cn2
ClCCNP1(=O)OCCCN1CCCl
CCC1(C)OC(=O)N(C1=O)C
O=C(Cc1ccccc1)NC(=O)N
CC(c1cccc(c1O)C(C)C)C
Fc1c[nH]c(=O)[nH]c1=O
CCCCOC(=O)c1ccc(cc1)N
O=C1N=C(OC1c1ccccc1)N
[O-]S(=O)(=O)c1ccccc1
NC12CC3CC(C2)CC(C1)C3
CC(N(CC#C)C)Cc1ccccc1
NCCCC(C(=O)O)(C(F)F)N
O=C(c1ccccc1)NCC(=O)O
NCCNC(=O)c1ccc(cn1)Cl
COC(=O)CCC[N+](C)(C)C
[O-]C(=O)C=CC(=O)[O-]
Oc1ccc(cc1)S(=O)(=O)O
CCCCOC(=O)c1ccc(cc1)O
ClCC[N+](CCCl)(C)[O-]
Cc1ccc(cc1)S(=O)(=O)O
OCC(C(C(C(C=O)O)O)O)O
O=C1CC(=NN1c1ccccc1)C
OC(=O)Cc1cccc2c1cccc2
CCCCCCOc1ccccc1C(=O)N
CC=NC(C(=O)[O-])C(O)C

Continued on next page

Table S8 – *Continued from previous page*

CCCCOCCOC(=O)c1cccnc1
CNCC(C(C(C(CO)O)O)O)O
CC(NCC(c1ccccc1Cl)O)C
OC(C(N(C)C)C)c1ccccc1
CCC1(C)CC(=O)NC(=O)C1
Nc1c2CCCCc2nc2c1cccc2
CC1OC1P(=O)([O-])[O-]
[O-]C(=O)CCC(C(=O)O)N
COC(=O)c1ccc(cc1)[O-]
CCOc1ccccc1OCC1CNCCO1
Nc1c2ccccc2nc2c1cccc2
COC1(F)C(C1(F)Cl)(F)F
C#Cc1cncc(c1)C1CCCN1C
COc1cc2c(N)ccnc2cc1OC
Cc1ccc(cc1)C12CNCC2C1
NCc1ccccc1Sc1ccccc1CO
[O-]C(=O)C[N+](C)(C)C
CC(Cc1ccc(cc1)Cl)(N)C
Cc1nc(C)c(c(c1Cl)O)Cl
OCCOCn1cnc2c1nc(N)nc2
NCC(=CF)CCc1ccc(cc1)F
c1ccc2c(c1)sc1c2cccc1
COP(=O)(OC=C(Cl)Cl)OC
CC12CCC(CC1)C(O2)(C)C
CC1NC(=O)COC1c1ccccc1
OCC1OC(=O)C(C(C1O)O)O
CCCCCCOC(=O)CCC(=O)CN
NC(=S)C1CCCc2c1nccc2C
CC(c1cccc(=O)c(c1)O)C
OC(=O)CC(CC(=O)O)(O)C
Oc1ccc(cc1)OCc1ccccc1
CN1CCCN=C1C=Cc1sccc1C
OC(=O)C(c1ccc(cc1)O)N
CCCCCCCCCCCCCCCC(=O)O
CNCC(c1ccc(c(c1)O)O)O
Cc1csc(c1NC1=NCCN1)Cl
S=c1nc[nH]c2c1cn[nH]2
CCC1(CC)C(=O)NC=CC1=O
C#CCC1(CCCCC1)OC(=O)N
CCCCNCC(c1ccc(cc1)O)O
SCC(C(=O)[O-])NC(=O)C
[O-]C(=O)CCCCCNC(=O)C
CC1SCC2(O1)CN1CCC2CC1
CC(c1cc(Cl)c(cc1O)C)C
O=c1cccc2n1CC1CNCC2C1
OCC(C(C(C(C=O)N)O)O)O
Oc1cc(C)c(c2c1cccc2)O
CNC(C(c1ccc(cc1)O)O)C
CC1=CCC(CC1O)C(O)(C)C
Nc1ccc(cc1)S(=O)(=O)N
CCOC(=O)N1CSCC1C(=O)O
CNC(=N)NCCCC(C(=O)O)N
OC(=O)CSc1n[nH]c(n1)C
OCC(C1OC(=O)C(=C1O)O)O
OC(=O)C(Cc1ccc(cc1)O)N
O=C1CC2C(C1(C)CC2)(C)C
Oc1ccc2c(c1)oc(=O)cc2C
CCC1C(=O)OCC1Cc1cncn1C
FCOC(C(F)(F)F)C(F)(F)F
S=P(N1CC1)(N1CC1)N1CC1
CNC1(C)C2CCC(C1(C)C)C2
OCCN1CC(O)C(C(C1CO)O)O
OC(c1ccccc1)CNc1ccccn1
NCc1ccc(cc1)S(=O)(=O)N
CCC(=O)c1c(O)cc(cc1O)O
O=C(c1ccccc1)NCCC(=O)O
BrC(C(=O)NC(=O)N)C(C)C
O=CC(C1OC(=O)C(C1O)O)O
BrCC(C(C(C(CBr)O)O)O)O
[O-]S(=O)S(=O)(=O)[O-]
Oc1nc(O)c2c(n1)[nH]nc2
[O-]S(=O)(=O)NC1CCCCC1
OS(=O)(=O)CCS(=O)(=O)O
CCCC(C(=O)NCC(=O)N)CCC
NC(C(=O)O)C1CC(=NO1)Cl
CNCC(=O)c1ccc(c(c1)O)O
OC(c1ccc(cc1)O)CNC(C)C
CCN(C(C(c1ccccc1)O)C)C
OC(c1ccccc1)CNc1ncccn1
C#Cc1ccc(cc1)c1ccccc1F
[O-]C(=O)CP(=O)(O)[O-]

Continued on next page

O=c1ccc2c([nH]1)ccnc2C
Oc1cccc(c1)Oc1nnn[nH]1
C=CC(NC(=S)NNC(=S)NC)C
CCCCCCCCOC(=O)C(=C)C#N
OC(P(=O)(O)O)P(=O)(O)O
CCCNC1CCc2c(C1)sc(n2)N
CCCC(N1CCCC1)Cc1ccccc1
COc1cc2c(CC)ncnc2cc1OC
CON=C(C1CN2CCC1CC2)C#N
CC1(O)CCC(CC1)C(O)(C)C
CN(C(=S)SSC(=S)N(C)C)C
Clc1ccc2c(c1)s[nH]c2=O
CCNC1(CCCCC1=O)c1cccs1
CCCCCCCCCCCCCC(=O)[O-]
Cc1cccc(c1NC1=NCCCS1)C
NC(=O)OCC(c1ccccc1Cl)O
Clc1ccc2c(c1)C(C)CNCC2
CCC(C1(O)CCCCC1)C(=O)O
NC(=N)N(CCOP(=O)(O)O)C
CC(CC(C(=O)O)NC(=O)C)C
Brc1cc(C)c2c(c1O)nccc2
CNC(=O)Oc1cccc2c1cccc2
[O-]C(=O)CSCC(C(=O)O)N
OC1C(O)C(O)C(C(C1O)O)O
CNC(=O)Oc1ccccc1OC(C)C
Fc1cccc(c1NC1=NCCN1)Br
[O-]C(=O)CCCCC(=O)[O-]
ClC(COP(=O)(O)O)(Cl)Cl
O=C1C=C(C)OS(=O)(=O)N1
Cc1ccc(nc1)c1ccc(cn1)C
CCOC(=O)c1ccc(cc1)[O-]
CCCCCCCCCCCCCCCCCC(=O)O
OCCCNC(=O)C(C(CO)(C)C)O
Clc1cccc(c1N=C1NCCN1)Cl
ClCCN(P1(=O)NCCCO1)CCCl
CCCNC(C(=O)Nc1ccccc1C)C
CC[N+](Cc1ccccc1Br)(C)C
OC(=O)c1ccc2c(c1O)cccc2
CCN(C(=O)N1CCN(CC1)C)CC
CCCCCCCCCCCCOS(=O)(=O)O
C1CN2C(=NCC2c2ccccc2)N1
CC[N+](c1cccc(c1)O)(C)C
CC(C[N+](C)(C)C)OC(=O)N
O=C1NS(=O)(=O)c2c1cccc2
O=C(c1ccccc1)OCc1ccccc1
CCC1(CC)C(=O)NCC(C1=O)C
CCN(c1cc(C)nc2n1ncn2)CC
CCN(CCOC(=O)c1cccnc1)CC
CCCCOc1ccc(cc1)CC(=O)NO
CC=CC(=O)N(c1ccccc1C)CC
O=C(c1cccnc1)OCc1ccccc1
OCC(OP(=O)([O-])[O-])CO
[O-]C(=O)C(C(C(=O)O)O)O
OCC([N+](=O)[O-])(CO)Br
Cc1ccc(=O)n(c1)c1ccccc1
COc1ccc(cc1)c1ssc(=S)c1
O=c1cc(n(n1c1ccccc1)C)C
FC(C(F)(F)F)(C(F)(F)F)F
NNC(CCc1ccc2c(c1)OCO2)C
CC(Cc1ccc(cc1)CC(=O)O)C
OC(C[N+](C)(C)C)CC(=O)O
CC(C(=O)Nc1c(C)cccc1C)N
CCN(C(=O)c1cccc(c1)C)CC
CC(C(c1ccc(c(c1)O)O)O)N
CCCOC(=O)c1ccc(cc1)[O-]
CCC(C(=O)NC(=O)N)(CC)Br
CCn1nnc(n1)C1=CCCN(C1)C
CCC(C(=O)NC(=O)N)C(CC)C
NC(=O)CN1Cc2ccccc2OC1=O
Clc1cc(Cl)c2c(c1O)nccc2
CCNC(=O)C=Cc1cccc(c1)Br
NC1=NCC(O1)c1ccc(cc1)Cl
Cc1cccc(c1C)Cc1c[nH]cn1
CC(=O)Nc1ccc(cc1)C(=O)O
C#Cc1c[nH]c(=O)[nH]c1=O
CCC(Cc1c[nH]c2c1cccc2)N
NC(C(=O)O)Cc1ccc(cc1)Cl
NC(=N)NC(CC(C)(C)C)(C)C
CC(COC(=O)c1ccc(cc1)N)C
CCCCCCCCCCCCN1CCCCCC1=O
Clc1ccc(cc1)c1nc[nH]c1C

Continued on next page

Table S8 – *Continued from previous page*

CC(C[N+](C)(C)C)OC(=O)C
CNC(=S)NCCSCc1nc[nH]c1C
CCCCN1CC(O)C(C(C1CO)O)O
Clc1ccc(c(c1)C)N=C1SCS1
CSc1ccccc1OCC(CNC(C)C)O
NC(=S)C1CCCc2c1ncc(c2)C
Brc1cc(Br)c2c(c1O)nccc2
Cc1cc(=O)[nH]c(=S)[nH]1
O=C(c1nccnc1)NCN1CCOCC1
CC(=NNC(=N)N)C=NNC(=N)N
COc1ccccc1OCC1CNC(=O)O1
O=C(C(C)(C)C)NCCCC(=O)O
CC(c1ccccc1OCC1=NCCN1)C
CN(CCOC(=O)CCC1CCCCC1)C
[O-][N+](=O)c1cnc(n1C)C
OCC(NCC(c1ccccc1)O)(C)C
N=C(NC(=N)N)NCCc1ccccc1
C=CCN1CCc2c(CC1)nc(s2)N
[O-]C(=O)CCN[N+](C)(C)C
O=c1[nH]cc(cc1N)c1ccncc1
NCC(c1ccc(cc1)Cl)CC(=O)O
SCC(C(=O)N1CCCC1C(=O)O)C
CN(N=Nc1[nH]cnc1C(=O)N)C
ClCCN(C(=O)NC1CCCCC1)N=O
COc1ccccc1OCC(COC(=O)N)O
CN1C(=O)CC(C1=O)c1ccccc1
NS(=O)(=O)Cc1noc2c1cccc2
[O-]C(=O)P(=O)([O-])[O-]
NC(=N)NN=Cc1c(Cl)cccc1Cl
OCC(C(C(C(C(=O)O)O)O)O)O
Cc1ccc(cc1)S(=O)(=O)[O-]
CNC1(CCCCC1=O)c1ccccc1Cl
C1CN=C(N1)C1CCCc2c1cccc2
Oc1cccc(c1)C=CC1=NCCCN1C
C=CCOc1ccc(cc1Cl)CC(=O)O
Oc1ccc(c(c1)S(=O)(=O)O)O
COc1cc(N)cn[n+]1c1ccccc1
OC(c1ccccc1Cl)CNC(C)(C)C
OC(=O)Cc1ccc(cc1)NC(=O)C
O=CC1(C)C2CCC1(C)C(=O)C2
CCC1(CC)C(=O)NC(=O)NC1=O
OC1OC(C(=O)N)C(C(C1O)O)O
CCOC(=O)NNc1nncc2c1cccc2
COc1c2CN(C)CCc2cc2c1OCO2
CCCCCCCCC=CCCCCCCCC(=O)O
O=C1C=C(C)C(=O)c2c1cccc2
c1ccc2c(c1)Sc1c(N2)cccc1
C1NCc2n(CC1)c1c(c2)cccc1
CN1C(=O)CN(C1=N)c1ccccc1
CNC(=O)ON(C(=O)NC)C(=O)C
CCOC(=O)NC(C(Cl)(Cl)Cl)O
Clc1ccc(c(c1)Cc1ccccc1)O
NC(C(=O)O)CSSCC(C(=O)O)N
CCN(C(C(=O)c1ccccc1)C)CC
CCOC(=O)c1ccccc1C(=O)OCC
CCC(Cc1cc(OC)c(cc1OC)C)N
CN(C(c1cccnc1)C1COCOC1)C
C=CCC(C(=O)NC(=O)N)C(C)C
Clc1ccc(cc1Cl)OCC1=NCCN1
OCC(CCCCCCC(CO)(C)C)(C)C
CCC(c1ccccc1)(COC(=O)N)O
OCC(COC(=O)c1ccc(cc1)N)O
O=c1c2ccccc2nnn1N1CCOCC1
CC1(C)CC(C)(C)CC(C1)(C)N
CC(=O)Oc1ccccc1C(=O)[O-]
OC(=O)CCCCCc1cccc2n1cnc2
OC(COc1ccc(cc1)O)CNC(C)C
CCN(CCNC(=O)c1ccccc1O)CC
CCCCC(COS(=O)(=O)[O-])CC
NC(=O)NC1CCC(=O)c2c1ccs2
NC(=O)c1ncn2c1nnn(c2=O)C
O=C(c1ccccc1C)NP(=O)(N)N
Cc1cc(nn2c1nnc2)N1CCCCC1
O=C(c1ccc(cc1)N)NCC(=O)O
NC(=O)CCC(C(=O)O)NC(=O)C
OC(=O)CCC(C(=O)O)NC(=O)N
Cc1ccc(c(c1)Cl)NC1=NCCN1
[O-][n+]1cc(ncc1C)C(=O)O
OC1CC(N(C1)C(=O)C)C(=O)O
O(CCOCC1OC1)CCOCCOCC1OC1
CCNC1C2CCC(C1c1ccccc1)C2

Continued on next page

O=C(NC1CCSC1=O)CSCC(=O)O
CNCCCCOc1ccccc1Cc1ccccc1
CC(=O)SCC(C(=O)O)NC(=O)C
OC(=O)CCC(C(=O)O)NC(=O)C
OC(=O)c1ccccc1OP(=O)(O)O
NC(=N)NCC1COC2(O1)CCCCC2
C1CN=C(N1)Nc1cccc2c1CCC2
CCCCCCCCCCCCCCCC(=O)NCCO
C1CN=C(O1)NC(C1CC1)C1CC1
C1CN=C(N1)C1CCCc2c1cccc2
CCOC(=O)c1cccc(c1)[NH3+]
NCC1(CC(=O)O)CC(C(C1)C)C
CCOC(=O)C(Cc1ccc(cc1)O)N
O=C1CCC(N1)C(=O)N1CCCCC1
Clc1ccc(cc1Cl)C12CNCC2C1
CC(=N)NCCSCC(C(=O)O)(N)C
CCOC(=O)CCCCCCCCC(=O)OCC
OC(=O)C(Cc1ccc(c(c1)O)O)N
COc1c2oc(=O)ccc2cc2c1occ2
CCCC(COC(=O)N)(COC(=O)N)C
ClC(COP(=O)(O)[O-])(Cl)Cl
OCCn1c(C)ncc1[N+](=O)[O-]
CNNCc1ccc(cc1)C(=O)NC(C)C
C[n+]1cccc(c1)OC(=O)N(C)C
CCCc1cc(=O)[nH]c(=S)[nH]1
CCN1C(=O)NC(C1=O)c1ccccc1
C1CN=C(N1)Cc1cccc2c1cccc2
C1CN2C(=NC(C2)c2ccccc2)S1
Oc1ccc(cc1)CC(C(=O)O)(N)C
CC(C12CC3CC(C2)CC(C1)C3)N
COc1ccc(c(c1)C(C(N)C)O)OC
CCOc1ccc(cc1)NC(=O)CC(O)C
C=CCc1ccccc1OCC(CNC(C)C)O
CCC(c1ccccc1)C(=O)NC(=O)N
C1CN=C(N1)Nc1cccc2c1CCCC2
CC(NCC(c1ccc(c(c1)O)O)O)C
OC(=O)Cc1ccc(cc1)c1ccccc1
CCOCCCNC(=O)C(C(CO)(C)C)O
SCC(C(=O)O)NC(=O)C(S)(C)C
CCCCCCCCCCCCCC[N+](C)(C)C
CC(P(=O)(O)O)(P(=O)(O)O)O
OC(=O)C1N2C(OC1=CCO)CC2=O
[O-][N+](=O)c1ccc(o1)C=NO
CNCCC(c1ccccc1)Oc1ccccc1C
COc1ccc(c2c1CCCC2N(C)C)Cl
[O-]C1=NC(C(=O)N1)NC(=O)N
C=CCN1C(=S)NC(C1=O)CC(C)C
N#Cc1cc(ccc1N)C(CNC(C)C)O
O=C(NC1CC1)C=Cc1cccc(c1)F
OCC1OC(CC1O)n1cnc(nc1=O)N
COc1cccc(c1)C[N+](CC)(C)C
CCOC(=O)N(C1CC1)Cc1ccccc1
ONC(=N)NN=Cc1c(Cl)cccc1Cl
[O-]P(=O)(CP(=O)(O)[O-])O
N#CCc1[nH]c(nc1C)c1ccccc1
OCc1n(=O)c2ccccc2n(=O)c1C
O=CC(C(C(C(C(=O)O)O)O)O)O
Cc1sc2c(c1CC1=NCCN1)cccc2
CNC(=NC)Cc1ccc2c(c1)cccc2
CCCCC(COC(=O)c1ccccc1O)CC
ClCCN(C(=O)NCC(C)(C)C)N=O
Nc1ccc(c(n1)N)N=Nc1ccccc1
CCCN1CCCC2C1Cc1cn[nH]c1C2
CC1C=CC(N1CC(=O)NC(=N)N)C
CCCCCCCCCCCCCCCC(C(CO)N)O
OCC(C1OC(=O)C(=C1[O-])O)O
Clc1ccc(cc1)S(=O)(=O)[O-]
CNCCOC(c1ccccc1C)c1ccccc1
OCC1OC(CC1O)n1ccc(nc1=O)N
CC(Cc1ccccc1)NCc1ccccc1Cl
[NH-]c1on[n+](c1)N1CCOCC1
O=C1NC(=NP(=O)(O)O)N(C1)C
CCCCNCC(=O)Nc1c(C)cccc1Cl
CCNC1=NC(=O)C(O1)c1ccccc1
OCC(CN1CCN(CC1)c1ccccc1)O
COc1c2ccc(=O)oc2cc2c1cco2
[O-]C(=O)COc1ccccc1C(=O)N
CSc1ccc(cc1C)OP(=S)(OC)OC
OC(c1cc(O)cc(c1)O)CNC(C)C
O=c1ccc2c(o1)cc1c(c2)cco1
CCN(C(=S)SC(=S)N(CC)CC)CC

Continued on next page

Table S8 – *Continued from previous page*

O=C1C=C(C)OS(=O)(=O)[N-]1
CN(CCOc1ccccc1Cc1ccccc1)C
CN(CC1CCCCC1c1cccc(c1)O)C
OC(=O)CCCCC1SCC2C1NC(=O)N2
OC(=O)C(CC(=O)O)(CC(=O)O)O
CC(Cc1ccc(cc1)C(C(=O)O)C)C
CCN(C(=S)SSC(=S)N(CC)CC)CC
CC(=O)Nc1nnc(s1)S(=O)(=O)N
OCCOCn1cnc2c1nc(N)[nH]c2=O
CS(=O)(=O)OCCCCOS(=O)(=O)C
N#CN=C(NCCSCc1nc[nH]c1C)NC
Nc1ccn(c(=O)n1)C1CSC(O1)CO
CCN(CC(=O)Nc1c(C)cccc1C)CC
CC(=O)OC(COC(=O)C)COC(=O)C
Nc1ccn(c(=O)n1)C1CCC(O1)CO
CCC1(C(=O)NCNC1=O)c1ccccc1
CCN(CCNC(=O)c1ccc(cc1)N)CC
Cc1cccc(c1C)C(c1c[nH]cn1)C
Cn1cnc2c1c(=O)n(C)c(=O)n2C
CCC1(CCC(=O)NC1=O)c1ccccc1
[O-][N+](=O)OC1COC2C1OCC2O
CCN(CCOC(=O)c1ccc(cc1)N)CC
OC(COC(=O)N)COc1ccc(cc1)Cl
Clc1ccc2c(c1NC1=NCCN1)nsn2
COP(=O)(C(C(Cl)(Cl)Cl)O)OC
CNCCCN1c2ccccc2CCc2c1cccc2
Clc1ccccc1CN1CCc2c(C1)ccs2
CN(CCC(c1ccccn1)c1ccccc1)C
COC(=O)C(c1ccccc1)C1CCCCN1
CCOc1c(cnn(c1=O)C)N1CCOCC1
Cc1cc(C2CCCCC2)n(c(=O)c1)O
CCCNC(COC(=O)c1ccccc1)(C)C
Oc1ccc(cc1)NC(=O)c1ccccc1O
C=CCOc1ccccc1OCC(CNC(C)C)O
COc1ccc(cc1)C(=O)N1CCCC1=O
Cn1c(=O)c2[n-]cnc2n(c1=O)C
C1COC(CN1)COc1cccc2c1CC=C2
OC(C[N+](C)(C)C)CC(=O)[O-]
CCCOC(=O)c1cc(O)c(c(c1)O)O
CNC(=N)NC(=O)Nc1c(C)cccc1C
OC(=O)C1NCCC(C1)CP(=O)(O)O
Cl[N-]S(=O)(=O)c1ccc(cc1)C
N#Cc1ccc(cc1)C1CCCc2n1cnc2
CC1CCCCN1CCCOC(=O)c1ccccc1
CC(Cn1cnc2c1c1ccccc1nc2N)C
OCC1OC(=O)N(C1)c1cccc(c1)C
Cc1onc(c1)C(=O)NNCc1ccccc1
CCCCC1(CC)C(=O)NC(=O)NC1=O
O=C1Nc2ccc(cc2C(O1)(C)C)Br
CNC1CCC(c2c1cccc2)c1ccccc1
OCCOCn1cnc2c1nc(N)[n-]c2=O
CC(=O)OC1C=CC(=O)OC1C1OC1C
ONC(=O)CNC(=O)c1ccc(cc1)Cl
CC(=O)Nc1ccc(cc1)OC(C)(C)C
C#CCN(CCCOc1ccc(cc1Cl)Cl)C
CCOCCOC(=O)C=Cc1ccc(cc1)OC
OC(=O)c1ccc(cc1)OCCn1cncc1
CCNC(Cc1cccc(c1)C(F)(F)F)C
Nc1[nH]c(=O)c2c(c1)[nH]cn2
[O-]C(=O)C1(CCC1)C(=O)[O-]
CCS(=O)CCCN1CCc2c(C1)cccc2
CCCCCCCCC=CCCCCCCCC(=O)OCC
CC(C(F)(F)F)OC(=O)C(=C)C#N
CNCC(c1cccc(c1)C(F)(F)F)OC
OC(=O)C(Cc1cc(O)c(cc1F)O)N
O=C(c1ccc(cc1)F)NP(=O)(N)N
CN(CCC(c1ccccc1)C1CCCCC1)C
CCn1ccnc1CC1COc2c(O1)cccc2
CCCN1CCCC2C1COc1c2cc(cc1)O
CCCCCCCCCCCCCCC(=O)OC(C)C
OS(=O)(=O)c1ccc2c(c1)cccc2
Fc1ccc(c2c1CCC2)OCC1CNCCO1
COc1cc(CCN=C2CCCN2C)ccc1OC
COc1nc(C)nc(c1NC1=NCCN1)Cl
OCC(c1ccc2c(c1)ccc(c2)OC)C
CCCN1CCOC2C1CCc1c2cc(cc1)O
Nc1c(Cl)cc(cc1Cl)CC1=NCCN1
CNCCC(c1ccccc1)Oc1ccccc1OC
CCCCCOC(=O)c1ccc(cc1)N(C)C
CC(C(OC(=O)N)C)C(OC(=O)N)C
CCCC1(CCN(C1)C)c1cccc(c1)O

Continued on next page

Table S8 – *Continued from previous page*

O=C(Nc1c(C)cccc1C)CN1CCCC1
N1CCN(CC1)c1ccc2c(n1)cccc2
OC(c1ccc(c(c1)O)O)C1CCCCN1
CCC1(CCC(=O)NC1=O)c1ccncc1
CCCCCCCCCCCCCCCC(=O)[O-]
Fc1cccc(c1)c1nn(c(=S)n1C)C
CCC(c1cccc(c1)O)C(CN(C)C)C
CCCCCCSc1nsnc1C1=CCCN(C1)C
OC1=C(O)C(=O)C(=C(C1=O)O)O
[O-]c1nc(=O)n(c(=O)n1Cl)Cl
Nc1ccn(c(=O)n1)C1COC(O1)CO
CCOC(=O)NP(=O)(N1CC1)N1CC1
OC1CCCc2c1c(N)c1c(n2)cccc1
CCCCCCOc1nsnc1C1=CCCN(C1)C
CNCC(c1cc(OC)c(c(c1)OC)O)O
Cc1ccc2c(n1)c(O)c(cc2Cl)Cl
CCCCCC=CCC=CCC=CCCCCC(=O)O
COC(=O)C(Cc1ccc(c(c1)O)O)N
CC(NNC(=O)COc1ccc(cc1)Cl)C
O=C1c2ncccc2c2c(C1=O)nccc2
CCN(CCc1onc(n1)c1ccccc1)CC
CCN(C(=O)CSc1ccc(nn1)Cl)CC
CN(C(Cc1ccccc1)C)Cc1ccccc1
Oc1ccc(cc1)C(=O)OCc1ccccc1
OC(=O)c1cc(=O)c2c(o1)cccc2
OC(=O)C(Oc1ccc(cc1)Cl)(C)C
COc1cc(OC)ccc1C(=CC(=O)O)C
O=C(c1ccncc1)NNCS(=O)(=O)O
COC(=O)c1cncn1C(c1ccccc1)C
O=C1[N-]S(=O)(=O)c2c1cccc2
Nc1[nH]c(=S)c2c(n1)[nH]cn2
CC(Cn1cnc2c1c1ncccc1nc2N)C
CNC(CC=Cc1cncc(c1)OC(C)C)C
OC(=O)c1ccc(cc1)S(=O)(=O)N
Cc1cccc(c1C)C(c1ncc[nH]1)C
Cn1cnc2c1c(=O)[nH]c(=O)n2C
OC(=O)C(Cc1c[nH]c2c1cccc2)N
OC(=O)c1ccccc1Nc1cccc(c1C)C
CN(CCOC(c1ccccc1)c1ccccc1)C
c1scc(n1)c1nc2c([nH]1)cccc2
CN1C(=O)CC(C1=O)(C)c1ccccc1
OC(COc1cccc2c1cccc2)CNC(C)C
CNCCCC1c2ccccc2C=Cc2c1cccc2
NC(=O)OCC(c1ccccc1)COC(=O)N
CCOC(=O)c1cncn1C(c1ccccc1)C
O=C(Cc1c(Cl)cccc1Cl)NC(=N)N
OCc1nc(ccc1O)C(CNC(C)(C)C)O
CN1CCCCC1C(=O)Nc1c(C)cccc1C
CCCCOc1ccc(cc1)OCCCN1CCOCC1
O=C1NCC(O1)COc1cc(C)cc(c1)C
Nc1nc2c(s1)cc(cc2)OC(F)(F)F
CNCCC=C1c2ccccc2CCc2c1cccc2
CN(CCOC(=O)COc1ccc(cc1)Cl)C
CC(c1ccc(c2c(c1)c(C)cc2)C)C
C1CCC2N(C1)CC1CC2CN2C1CCCC2
CCCCCCCCCCCCCCCC[n+]1ccccc1
NC(=S)NS(=O)(=O)c1ccc(cc1)N
Nc1nc(=O)n(cc1F)C1CSC(O1)CO
O=c1[nH]c(=O)n(cc1F)C1CCCO1
OCC(C(C(C(C(=O)[O-])O)O)O)O
COc1ccc(cc1C(CNC(=O)CN)O)OC
CCC1(CC)C(=O)NC(=O)N(C1=O)C
COc1cc(C)nc(n1)n1nc(cc1OC)C
O=C(c1ccc(cc1)N)NCC(=O)[O-]
N#Cc1ccccc1OCC(CNC(C)(C)C)O
CCCCCC=CCC=CCCCCCCCC(=O)OCC
OCC1OC2C(C1O)Oc1n2ccc(=N)n1
OC(=O)C=Cc1ccc(cc1)Cn1ccnc1
CCC(=C(c1ccccc1)c1ccccc1)CN
CCOC(=O)Cc1ccc(cc1)c1ccccc1
OC(COc1cccc2c1C=CC2)CNC(C)C
Brc1c(ccc2c1nccn2)NC1=NCCN1
CN(CCN(c1ccccn1)Cc1ccccc1)C
OCc1cc(ccc1O)C(CNC(C)(C)C)O
CCOP(=O)(SCC[N+](C)(C)C)OCC
COCCc1ccc(cc1)OCC(CNC(C)C)O
Nc1ccc(cc1)S(=O)(=O)N=C(N)N
OC(=O)c1cc(ccc1O)S(=O)(=O)O
NC(=S)NN=C1c2ccccc2N(C1=O)C
CN(CCN1c2ccccc2Sc2c1cccc2)C
COc1cc(C=CC(=O)N)cc(c1OC)OC

Continued on next page

Nc1ncnc2c1ncn2CCOCP(=O)(O)O
C=CCc1ccccc1OCC(=NO)CNC(C)C
CN(c1nc(nc(n1)N(C)C)N(C)C)C
CCN(CCCc1ccccc1)CCCc1ccccc1
CC(Cc1ccccc1)N=CC(Cl)(Cl)Cl
CCCN(n1ccc2c1cccc2)c1ccncc1
N=C(Nc1c(Cl)cccc1Cl)NC(=O)N
CCCCCCCCCCCCCCCC[N+](C)(C)C
CCC(CCCCC1CCC2C1CC(=O)C2)OC
ClC(P(=O)(O)O)(P(=O)(O)O)Cl
NC(=O)C1c2ccccc2CCc2c1cccc2
COc1cc(ccc1OC)Cc1cnc(nc1N)N
CC12NC(c3c2cccc3)Cc2c1cccc2
OC(=O)C1C2C1C(CC2)(N)C(=O)O
CCN(C(=O)c1ccc(c(c1)OC)O)CC
CCOC(=O)C(Cc1ccc(c(c1)O)O)N
Oc1ccc(cc1Sc1cc(Cl)ccc1O)Cl
COc1ccccc1OCCOCCOCCN1CCCCC1
CC(c1ncc(n1C)[N+](=O)[O-])C
CCCCN(C(=O)C(C)C)c1occ(n1)C
CCOC(=O)Nc1nc2c([nH]1)cccc2
CC(C1=NCCN1)Oc1c(Cl)cccc1Cl
CC12CC3CC(C1)(C)CC(C2)(C3)N
CCC1(CCCCN(C1)C)c1cccc(c1)O
[O-][N+](=O)c1nccn1CC(COC)O
CC1OCC(CO1)(Br)[N+](=O)[O-]
O=C(c1ccccc1)N1CC2C(C1)COC2
ClCCN(P1(=O)OCCC(N1)OO)CCCl
ClCCN(C(=O)NC1CCC(CC1)C)N=O
CN(c1ccccc1)N=NN(c1ccccc1)C
CC(=O)NS(=O)(=O)c1ccc(cc1)N
CN(C1=NC(=O)C(O1)c1ccccc1)C
Cc1cccc(c1)Oc1cnc(=O)[nH]c1
c1ccc(cc1)Cc1nnc2c(n1)cncc2
ONC(=O)CCCCCCC(=O)Nc1ccccc1
C1NCc2c3N(C1)CC1CCCC1c3ccc2
C=CCN1Cc2ccccc2c2c(C1)cccc2
ClCCN(P1(=O)OCCCN1CCCl)CCCl
CCCCC1C(=O)NN(C1=O)c1ccccc1
ONC(=O)C(c1ccc(cc1)CC(C)C)C
NCCC(P(=O)(O)O)(P(=O)(O)O)O
CN(CC(Oc1ccccc1)Oc1ccccc1)C
OC(=O)CSC(SCC(=O)O)SCC(=O)O
CCOC(=O)CSCCC1NC(CS1)C(=O)O
OCC(C(C(=O)NCCC(=O)O)O)(C)C
Oc1ccc(c(c1)S(=O)(=O)[O-])O
Oc1ccc(c(c1)S(=O)(=O)[O-])O
ClC(C(=O)N(c1ccc(cc1)O)C)Cl
OC(=O)c1cc(ccc1O)S(=O)(=O)O
Cc1ccc2c(c1)c1CCCC3c1n2CCN3
NCCCC(Nc1cc(OC)cc2c1nccc2)C
OC(COc1cccc2c1cccc2)CNC(C)C
CC(=O)Nc1ccc(cc1)C=NNC(=S)N
COc1c(ccc(c1OC)OC)CN1CCNCC1
O=c1n(Cl)c(=O)[nH]c(=O)n1Cl
CCCC1CN(C(=O)C1)C(C(=O)N)CC
CCOc1cc(ccc1C(=O)OC)NC(=O)C
Clc1cc(cnc1Cl)N1CC2C(C1)CN2
CCC(=O)NCC1CC1c1cccc2c1CCO2
CC(=O)OC1CC2C(C1(C)CC2)(C)C
NCCC(c1cccc(c1)OCC1CCCCC1)O
OC(=O)C(Cc1cn(c2c1cccc2)C)N
CCCCCCCCCCCCCCCCCC(=O)OCCCC
Clc1ccc(cc1)CCCOCCCN1CCCCC1
CCC1=CC2C(C1)CC2(CN)CC(=O)O
O=Cc1c(COP(=O)(O)O)cnc(c1O)C
O=c1[nH]c(=O)[nH]c(c1)C(=O)O
OCC1OC(CC1O)n1cnc2c1N=CNCC2O
OCC1OC(C(C1O)O)n1ccc(nc1=O)N
[O-]C(=O)C1N2C(OC1=CCO)CC2=O
Oc1cccc2c1C(=O)c1c(C2)cccc1O
NC(=O)N1c2ccccc2C=Cc2c1cccc2
CCOC(=O)C(Oc1ccc(cc1)Cl)(C)C
Nc1nnc(c(n1)N)c1cccc(c1Cl)Cl
ClC1C(Cl)C(Cl)C(C(C1Cl)Cl)Cl
Cn1c(=O)n(C)c2c(c1=O)[nH]cn2
CCC1(NC(=O)N(C1=O)C)c1ccccc1
O=C(C(c1cccnc1)(C)C)c1cccnc1
COc1ccc2c(c1)ccc(c2)CCC(=O)C
BrCCC(=O)N1CCN(CC1)C(=O)CCBr
CCc1nc(N)nc(c1c1ccc(cc1)Cl)N

Continued on next page

CCCCNc1ccc(cc1)C(=O)OCCN(C)C
Cc1nc2ccccc2c(=O)n1c1ccccc1C
CC(=O)N=c1sc(nn1C)S(=O)(=O)N
CCN(CCOC(=O)c1ccc(cc1Cl)N)CC
CN(CCOC(c1ccccc1C)c1ccccc1)C
CCCN(CCC)CCc1cccc2c1CC(=O)N2
CN(CCCN1c2ccccc2Sc2c1cccc2)C
Nc1cc(Cl)c(c(c1)Cl)NC1=NCCN1
Nc1ccc(cc1)S(=O)(=O)Nc1nccs1
OC(=O)c1ccccc1Nc1cccc(c1C)Cl
OC(=O)C(C(c1ccc(c(c1)O)O)O)N
CNC(=O)OCc1cccc(n1)COC(=O)NC
CN(C(=O)CN(c1ccccc1)C(=O)C)C
CC(=C)CNc1ccc(cc1)C(C(=O)O)C
CCCCCOc1ccccc1C(=CSC)n1cncc1
O=CC(C(C(C(C(=O)[O-])O)O)O)O
O=c1nc(N)[nH]c(c1Br)c1ccccc1
NC1=NCC2N1c1ccccc1Cc1c2cccc1
CCC(C(COC(=O)N)(COC(=O)N)C)C
CCCCCC=CCC=CCCCCCCCC(=O)[O-]
c1ccc(cc1)c1[nH]cnc1c1ccccc1
C[N+](CCCCCC[N+](C)(C)C)(C)C
CC(OC(=O)c1ccccc1)CNC1CCCCC1
OCC(C(C(C(C(C(=O)O)O)O)O)O)O
OC(=O)CC1C(CNC1C(=O)O)C(=C)C
O=C(c1ccc(cc1)Cl)NCCN1CCOCC1
CCC1C(=O)NC(=O)C1(C)c1ccccc1
CCC(=O)NCCC1CCc2c1c1CCOc1cc2
CCCCCCC1CCC(C1CCCCCCC(=O)O)O
C=CCC1(CC=C)C(=O)NC(=O)NC1=O
OCC1OCC(O1)n1cnc2c1nc(N)nc2N
CNCC(c1cccc(c1)NS(=O)(=O)C)O
COc1ccc(cc1)C=CC(=O)OCCC(C)C
Fc1cccc(c1Cn1cnc2c1ncnc2N)Cl
OCC1OC(C(C1O)O)n1cnc(nc1=O)N
[N-]=[N+]=CC(=O)OCC(C(=O)O)N
O=C(c1ccccc1)Oc1cccc2c1nccc2
O=C(c1ccccc1)OOC(=O)c1ccccc1
CC(=O)C=CC=C1C2CCC(C1(C)C)C2
O=c1[nH]c2c(o1)cc(c(c2)Cl)Br
CCC(C1(CC)C(=O)NC(=O)NC1=O)C
O=C(c1ccccc1)C[n+]1csc(c1C)C
CCCCC(=N)NC(=O)Nc1ccc(cc1)Cl
Oc1cccc(c1)C1CNC2(O1)CCCCCC2
CC(CCC#CC1CC1c1[nH]cnc1)(C)C
CN(C(C1CCCCC1O)c1cccc(c1)O)C
NC(=O)C1c2ccccc2C=Cc2c1cccc2
CN1C(=O)CN(C1=N)c1ccc(cc1)Cl
OC(=O)c1cccnc1Nc1cccc(c1C)Cl
OC(c1ccc(c(c1)O)O)CNC(C)(C)C
CN(C1CC(c2c1cccc2)c1ccccc1)C
Nc1nc(=O)n(cc1F)C1OC(C=C1)CO
CCOc1ccc(cc1)N=Nc1ccc(cc1N)N
O=C(c1ccc2c(c1)non2)N1CCCCC1
OCC1CC(C(O1)n1cnc2c1ncnc2N)F
CCCCCCCCCCCCCCCC[N+](CC)(C)C
[O-]C(=O)CC(C(=O)[O-])[NH3+]
CCC1(C(=O)NC(=O)N1C)c1ccccc1
CCCCCCCCCCCCCCC1(CO1)C(=O)OC
C1CN=C(N1)CC1=Cc2c(CC1)cccc2
CC(=O)Nc1ncc(s1)[N+](=O)[O-]
COc1ccc(c(c1)O)C(=O)c1ccccc1
C1CCC(CC1)(N1CCCCC1)c1ccccc1
Nc1ccc(c(c1)O)C(=O)Oc1ccccc1
CCCC1(CCN(CC1C)C)c1cccc(c1)O
OCc1nn2c(c1)c1ccccc1[nH]c2=O
CCCCCCCCN=c1ccn(cc1)CCCCCCCC
CC(NCCCNC(=O)Nc1c(C)cccc1C)C
Fc1cccc(c1Cn1nnc(c1)C(=O)N)F
O=c1n(Cl)c(=O)n(c(=O)n1Cl)Cl
C1CCC(=Cc2ccccc2)C(C1)N1CCC1
CCOCCP(CCOCC)CCP(CCOCC)CCOCC
N1CC2CC(C1)c1c2cc2c(c1)nccn2
O=C(Nc1c(C)cccc1C)N=C1CCCN1C
CCCCc1cc2ccccc2c(n1)OCCN(C)C
NCCCC(P(=O)(O)O)(P(=O)(O)O)O
CCN(CCn1c(=N)onc1c1ccccc1)CC
Cc1ccc2c(c1)Sc1c(S2)cc(cc1)C
COCC(C(=O)NCc1ccccc1)NC(=O)C
CN(CCCN1c2ccccc2Sc2c1nccc2)C
CC(Cn1c(C)ncc1[N+](=O)[O-])O

Continued on next page

Table S8 – *Continued from previous page*

Cc1ccc(c(c1)OCC1=NCCN1)C(C)C
N#CC(c1ccccc1)NC(Cc1ccccc1)C
NC(=N)NC(=O)c1nc(Cl)c(nc1N)N
CCC(OC(=O)c1ccccc1)(CN(C)C)C
CCC(c1ccccc1)C(=O)OCCN(CC)CC
[NH3+]CCCCC(C(=O)[O-])[NH3+]
O=C1N=CN=C2C1(CC1CC=CC=C1)S2
CN(CCCC1c2ccsc2SCc2c1cccc2)C
OCCn1cnc2c1c(=O)n(C)c(=O)n2C
OCC(C(C(=O)NCCCC(=O)O)O)(C)C
COC1=CC(=O)OC(C1)C=Cc1ccccc1
NCCCCC(C(=O)NC(Cc1ccccc1)C)N
OC(=O)C(Cc1ccc(c(c1)O)O)(N)C
NC(=O)NN=C1C=Cc2c(C1=O)cccc2
CCCCOCCOCCOCc1cc2OCOc2cc1CCC
OC(c1cc(O)cc(c1)O)CNC(C)(C)C
NCC(c1cc(ccc1F)NS(=O)(=O)C)O
Cc1ccc(cc1)Sc1ccccc1C1CCNCC1
CCCCCCCCCCCCC[n+]1ccc(cc1)C
CCC(n1c(O)nc2c1nc(C#C)cn2)CC
CN1C(=O)N(C2C1N(C)C(=O)N2C)C
OCC1OC(C(C1O)O)n1cnc2c1ncnc2N
OCC1CCC(O1)n1cnc2c1nc[nH]c2=O
ON(C(c1cc2c(s1)cccc2)C)C(=O)N
N#Cc1cc(c2ccncc2)c([nH]c1=O)C
Nc1cc(nc([n+]1[O-])N)N1CCCCC1
Oc1ccccc1C(=O)Oc1ccccc1C(=O)O
CCCC(C1(CC)C(=O)NC(=O)NC1=O)C
OC(COc1cccc2c1cc[nH]2)CNC(C)C
CCC1(CCC(C)C)C(=O)NC(=NC1=O)O
NNC(C(=O)O)(Cc1ccc(c(c1)O)O)C
CCC1(CCC(=O)NC1=O)c1ccc(cc1)N
Nc1ccc(cc1)S(=O)(=O)Nc1ncccn1
C=CCC1(C(C)C)C(=O)NC(=O)NC1=O
CN(CCCN1c2ccccc2CCc2c1cccc2)C
ClCCN(C(=O)NCc1cnc(nc1N)C)N=O
Cc1cccn2c1ncc(c2=O)c1[n-]nnn1
NCC1CC1(c1ccccc1)C(=O)N(CC)CC
CNCCC(c1cccs1)Oc1cccc2c1cccc2
CN(CCCOC1(CCCCCC1)Cc1ccccc1)C
COc1cccc(c1)C1(O)CCCCC1CN(C)C
CCOC(=O)C1(CCN(CC1)C)c1ccccc1
C[NH+](C(c1ccccc1)Cc1ccccc1)C
OC(COc1cc(C)ccc1Cl)CNC(C)(C)C
CN1CCCC(=C(c2cccs2)c2cccs2)C1
CS(=O)(=O)OCCCNCCCOS(=O)(=O)C
CC(C(=O)c1ccc(cc1)C)CN1CCCCC1
OCC(C(=O)NNCc1ccc(c(c1O)O)O)N
OCC1OC(C(C1O)O)n1cnc2c1ncnc2S
C[NH2+]CCCCOc1ccccc1Cc1ccccc1
[O-][N+](=O)OCCNC(=O)c1cccnc1
Oc1ccc(c(c1)Cl)C(CNC(C)(C)C)O
CN1CCC2(CC(C1)Cc1c2cc(cc1)O)C
O=C(c1ccncc1)NNCS(=O)(=O)[O-]
CCC(C(c1ccc(c(c1)O)O)O)NC(C)C
OCCc1sc[n+](c1C)Cc1cnc(nc1N)C
OC(=O)C(c1cccc(c1)Oc1ccccc1)C
Nc1ccc(cc1)S(=O)(=O)Nc1ccccn1
Clc1ccc(cc1)Cn1c(C)nc2c1cccc2
COc1cc2cc(oc2c(c1)Br)C1CCNCC1
COc1ccc2c(c1)c(CCNC(=O)C)ccc2
CCN(CCN1c2ccccc2Sc2c1cccc2)CC
O=C(c1ccc(cc1)N)Nc1c(C)cccc1C
CCCc1ncc(c(n1)N)C[n+]1ccccc1C
CN1CCN2C(C1)c1cccn1Cc1c2cccc1
CCC1(Cc2c(C1)cccc2)c1cnc[nH]1
Cc1ccc(cc1)C(OC(=O)c1cccnc1)C
CCN(P(=O)(N1CC1)N1CC1)c1scnn1
CNCC12CCC(c3c1cccc3)c1c2cccc1
CN1CC(OC1C(C)(C)C)c1cccc(c1)O
Clc1ccc2c(c1)CCC1C2CCC(=O)N1C
CCC(c1ccc(cc1)c1ccccc1)C(=O)O
CCCC(=O)OC1CN2CCC(C2C(C1O)O)O
Clc1ccc(cc1)C1OCc2c1cnc(c2O)C
CN(C1CCc2c(C1)c1ccccc1[nH]2)C
CNCCC(c1cccc(c1)F)c1cccc(c1)F
COc1ccc(c(c1)O)C(=O)c1ccccc1O
COc1cc(C)c(c(c1CC1=NCCN1)C)OC
Cc1ccc(c(c1)n1nc2c(n1)cccc2)O
[O-]S(=O)(=O)CCCS(=O)(=O)[O-]
CCCN1CCCCC1C(=O)Nc1c(C)cccc1C

Continued on next page

Table S8 – *Continued from previous page*

[O-][N+](=O)c1nccn1CC(=O)NCCO
CCOC(=O)c1cncn1C1CCCc2c1cccc2
CCC1(OCC(O1)C1CCCCN1)c1ccccc1
OC(COc1ccccc1C1CCCCC1)CNC(C)C
Oc1ccc2c(c1)C(CC2)Cc1c[nH]cn1
Clc1ccc(cc1)OCC1=NCCc2c1cccc2
NCC1Cc2ccc(cc2C(O1)c1ccccc1)F
FC(c1ccc(cc1)C1CN=C(O1)N)(F)F
OCC(OCn1cnc2c1nc(N)nc2[O-])CO
CCOC(C(=NNC(=S)N)C=NNC(=S)N)C
CN(CCCn1c2CCCCCCc2c2c1cccc2)C
O=C1NC2C(N1)C(SC2)CCC=CC(=O)O
Cc1nc2ccccc2c(=O)n1c1ccccc1Cl
CCN(CCNC(=O)COc1ccc(cc1)OC)CC
COc1ccc(cc1)OCC1=NCCc2c1cccc2
O=C(c1ccc2c(c1)cccc2)Cn1cncc1
CN1CCOC(c2c(C1)cccc2)c1ccccc1
[O-][N+](=O)c1ccc(o1)C(=O)CCl
CCC(=O)OC1(CCN(C1C)C)c1ccccc1
CCCC(C(=O)c1ccc(cc1)C)N1CCCC1
O=C1NC2=Nc3c(CN2C1C)c(Cl)ccc3
Clc1ccc2c(c1)C(=O)N1C(O2)CCO1
CCC(N1CC(CC1=O)C=C(F)F)C(=O)N
CC1=CC(=O)[C-](C(=O)O1)C(=O)C
CC(CC12CC3CC(C2)CC(C1)C3)(N)C
Fc1cc2C3(CCOc2cc1)NC(=O)NC3=O
CC(Cn1c(C)nc2c1c1ncccc1nc2N)C
Nc1ccc(cc1)S(=O)(=O)Nc1ccccc1
CCN(CCOc1ccc(cc1)Cc1ccccc1)CC
C[N+](Cc1cccs1)(CCOc1ccccc1)C
CCCOc1ccc2c(c1)sc(n2)NC(=O)OC
CCCC(COC(=O)N)(COC(=O)NCCCC)C
Clc1ccccc1C1=NCCNc2c1c(C)nn2C
CCC(C(=O)NC(=O)NC(=O)C)(CC)Br
CN(CCC(c1ccccc1)c1ccc(cc1)C)C
[O-][N+](=O)c1ccc(c2c1cccn2)O
O=C1CCC(N1)C(=O)N1CSCC1C(=O)O
CCN(CCNC(=O)COc1ccc(cc1)Cl)CC
CC(CC(O)(C)C)OC(C(Cl)(Cl)Cl)O
[O-][N+](=O)c1cncn1CCN1CCOCC1
Cc1cccn2c1ncc(c2=O)c1nnn[nH]1
c1ccc(cc1)Cc1nc2c([nH]1)cccc2
[O-]C(=O)CCCCC1SCC2C1NC(=O)N2
[O-]C(=O)C(CC(=O)O)(CC(=O)O)O
CC=CC(=O)N(C(C(=O)N(C)C)CC)CC
OC(=O)Cc1cscc1Nc1c(Cl)cccc1Cl
CC(Cc1ccc(cc1)C(C(=O)[O-])C)C
OCCNC(=O)C(c1ccc(cc1)CC(C)C)C
O=C1C(c2ccccc2)C(=O)c2c1cccc2
CCN(CC(=O)Nc1c(C)cc(cc1C)C)CC
COc1ccc2c(c1)ccc(c2)C(C(=O)O)C
OC(COc1ccc(cc1)CC(=O)N)CNC(C)C
ClCCN(c1ccc(cc1)CCCC(=O)O)CCCl
Clc1ccc2c(c1)S(=O)(=O)NC(=N2)C
OCC(OCn1cnc2c1nc(N)[nH]c2=O)CO
OCC1OC(C(C1O)O)n1cnc(n1)C(=O)N
CN(C(CN1c2ccccc2Sc2c1cccc2)C)C
CCC1(C(=O)NC(=O)NC1=O)c1ccccc1
ClCCN(C(COc1ccccc1)C)Cc1ccccc1
CN(c1c(C)n(n(c1=O)c1ccccc1)C)C
CN1CCN2C(C1)c1ccccc1Cc1c2nccc1
CN(CCC(c1ccccn1)c1ccc(cc1)Br)C
CN(CCC(c1ccccn1)c1ccc(cc1)Cl)C
CCCCOc1ccc(cc1)C(=O)CCN1CCCCC1
CCCC(COC(=O)N)(COC(=O)NC(C)C)C
CN(CCC=C1c2ccccc2CCc2c1cccc2)C
CN(CCC=C1c2ccccc2OCc2c1cccc2)C
Clc1cccc(c1)C(=O)C(NC(C)(C)C)C
Oc1ccc2c(c1)C1(C)CCCCCC(C2)C1N
CC(=O)[N-]S(=O)(=O)c1ccc(cc1)N
CN(C(CN1c2ccccc2Sc2c1nccc2)C)C
CC(=O)Nc1nnc(s1)S(=O)(=O)[NH-]
CCCCN1CCCCC1C(=O)Nc1c(C)cccc1C
S=C1SCN(CN1Cc1ccccc1)Cc1ccccc1
CN1CCN2C(C1)c1ccccc1Cc1c2cccc1
OCC1OC(CC1O)n1cnc2c1nc(Cl)nc2N
CC(c1c(C)n(n(c1=O)c1ccccc1)C)C
CC(C(=O)c1c(nn2c1cccc2)C(C)C)C
CCOC(=O)C1(CCCN(CC1)C)c1ccccc1
OC1CCC(CC1)NCc1cc(Br)cc(c1N)Br
Oc1ccc(cc1)C(=O)c1ccccc1C(=O)O

Continued on next page

Table S8 – *Continued from previous page*

CN(CCC=C1c2ccccc2SCc2c1cccc2)C
CC(C(Cc1ccc(cc1)Cl)(O)C)CN(C)C
Clc1ccc(c(c1)c1nc(N)nc(n1)N)Cl
OCC(NC1CC(O)(CO)C(C(C1O)O)O)CO
CCc1ccc(cc1)C(=O)C(CN1CCCCC1)C
[O-]C(=O)C=Cc1ccc(cc1)Cn1ccnc1
CCOC(=O)C=C(C=CCC(CCCC(C)C)C)C
OC(c1ccccc1)(c1ccccc1)C1CCCCN1
CCCCCCCCCCCCCCCCCC(=O)OCC(CO)O
CN1CCN(CC1)C(c1ccccc1)c1ccccc1
CCCOc1ccc(cc1N)C(=O)OCCN(CC)CC
CCCOc1cc(N)ccc1C(=O)OCCN(CC)CC
CN(CCOC(c1ccccn1)(c1ccccc1)C)C
CCOc1ccc2c(c1)sc(n2)S(=O)(=O)N
CC(N=C(N=C(Nc1ccc(cc1)Cl)N)N)C
CC(NC(=N)NC(=N)Nc1ccc(cc1)Cl)C
Nc1ccc(cc1)S(=O)(=O)c1cnc(s1)N
O=C1NC(=O)C2C1(C2)c1ccc(cc1)Cl
CN(CCN1CCN(C1=O)c1cccc(c1)Cl)C
NC(=O)c1ccc(cc1Cl)[N+](=O)[O-]
CC(=O)N(OCc1ccccc1)CCCc1ccccc1
COc1c(OC)ccc2c1c(C)nc(=O)[nH]2
C=CCC1(CC(C)C)C(=O)NC(=O)NC1=O
CCCCNc1c(cnc2c1cnn2CC)C(=O)OCC
CN1C(=Nc2c(CC1c1ccccc1)cccc2)C
O=C1c2ccccc2CN2N1Cc1ccccc1C2=O
CCOCCOC(=O)c1nc2c(o1)ccc(c2)Cl
Cc1nc(NCCCCCCc2ccccc2)nc(c1O)C
C=CCOC(c1ccc(cc1Cl)Cl)Cn1cncc1
CCCn1c(=O)[nH]c(=O)c2c1nc[nH]2
c1ccc(cc1)CCNc1scc(n1)c1ccccc1
OC(=O)Cc1ccccc1Oc1ccc(cc1Cl)Cl
CCC1C(C)C(CC=C1c1ccccc1)C(=O)O
O=C1NCC2(O1)CCN(CC2)CCc1ccccc1
CCCCCCCCc1ccc(cc1)CCC(CO)(CO)N
Fc1ccc2c(c1)n(C)cc(c2=O)S(=O)C
FC(c1ccc(cc1)C1OCC(=O)NC1)(F)F
CNCCSC1=Cc2ccccc2Oc2c1cc(F)cc2
OCC1OC2C(C1O)Oc1n2cc(F)c(=N)n1
COP(=O)(Oc1nc(Cl)c(cc1Cl)Cl)OC
O=C1CCC(=NN1)c1ccc(cc1)n1cncc1
CCC(=O)OC(C[N+](C)(C)C)CC(=O)O
CCCC(COC(=O)N)(COC(=O)NC1CC1)C
Clc1ccc(c(c1)Cl)C(=Cn1cncn1)Cl
COCCNC(=O)c1ccnc(c1)C(=O)NCCOC
CCOC(=O)C(Cc1ccc(c(c1)O)O)(N)C
Cc1cc(nnc1NCCN1CCOCC1)c1ccccc1
O=C1NCCN1c1ncc(s1)[N+](=O)[O-]
NCCSC(c1ccccc1Cl)C[N+](=O)[O-]
CN1CC(c2ccccc2)c2c(C1)c(N)ccc2
O=C(c1ccccc1Nc1ccncc1)N1CCCCC1
OCCC(Cn1cnc2c1[nH]c(N)nc2=O)CO
COc1cc(Cc2cnc(nc2N)N)c(cc1OC)C
ClCC(Cn1c(C)ncc1[N+](=O)[O-])O
COC1(OC)CCCN2C1=NC(C2)c1ccccc1
OC(=O)C=C1SC(C(=O)N1C)N1CCCCC1
OCC(CCn1cnc2c1[nH]c(N)nc2=O)CO
FC(C(C(F)(F)F)(F)F)(C(F)(F)F)F
C1CCC(NC1)CC(C1CCCCC1)C1CCCCC1
N#CNC(=Nc1ccncc1)NC(C(C)(C)C)C
CN(CCC1(C)OCCC2=C1Cc1c2cccc1)C
CC(CC(C)(C)C)Cc1cc(C)cc(=O)n1O
CN1CCCC1=NC(=Nc1ccccc1)N1CCCC1
OCC(C(C(C(C(=O)[O-])[O-])O)O)O
OC(COc1ccc(cc1)NC(=O)C)CNC(C)C
Clc1cccc(c1)C1(O)OCC(NC1C)(C)C
O=C(C1CCC(=O)N1)NC1CC1c1ccccc1
NC(=O)OCc1ncc(n1C)[N+](=O)[O-]
COP(=S)(Oc1cc(Cl)c(cc1Cl)Cl)OC
OC(C(C)(C)C)C=Cc1ccc2c(c1)OCO2
COc1cccc(c1)C(NCCCc1ccccc1Cl)C
CC(Cn1cnc2c1ncnc2N)OCP(=O)(O)O
OCC1OC(C(=CF)C1O)n1ccc(nc1=O)N
OCC1OC(C(C1O)O)c1scc(n1)C(=O)N
CCOc1cc2sc(cc2cc1OCC)C(=O)[O-]
Clc1ccc(c(c1)O)Oc1ccc(cc1Cl)Cl
C=CCC(Cc1cc(OC)c(c(c1)OC)OC)NC
COc1ccc(cc1)CN(c1nccs1)CCN(C)C
Clc1ccc2c(c1)c(no2)C(n1cncc1)C
OCC1OC(C(C1O)O)n1cnc(n1)C(=N)N
CCC(C1(CC=C)C(=O)NC(=O)NC1=O)C

Continued on next page

Oc1cccc2c1C(=O)c1c(C2=O)cccc1O
OC(COc1cccc2c1SCCC2)CNC(C)(C)C
OC(COc1cc(Cl)ccc1Cl)CNC(C)(C)C
CN1CCC(CC1)N(c1ccccc1)Cc1cccs1
OC(=O)c1cc(nc2c1cccc2)c1ccccc1
CC(=O)Oc1c(cccc1C(=O)O)OC(=O)C
CC1CCN(CC1)CCCC(=O)c1ccc(cc1)F
CCCC(C1(C=C)C(=O)NC(=O)NC1=O)C
CCC=C(C1(CC)C(=O)NC(=O)NC1=O)C
C=CCC1(CC(O)C)C(=O)NC(=O)NC1=O
OC(=O)C(Cc1c[nH]c2c1cc(O)cc2)N
NC(=N)NN=C1C=CC(=NNC(=S)N)C=C1
OC(=O)CNC(=O)C(SC(=O)c1cccs1)C
CN(CCC(c1ccccn1)c1ccc(cc1)Cl)C
CCCN(C(C(=O)N(C)C)CC)C(=O)C=CC
OC1CCC(CC1)NCc1cc(Br)cc(c1O)Br
OC(=O)Cc1ccccc1Nc1c(Cl)cccc1Cl
CCCCCCCCCC[N+](CCCCCCCCCC)(C)C
Clc1cccc(c1)C(=O)C(NC(C)(C)C)C
CCC=CCC=CCC=CCC=CCC=CCCCC(=O)O
OC(=O)C(c1ccc(cc1)CC1CCCC1=O)C
CCCCCCCCC(=O)NCc1ccc(c(c1)OC)O
[NH3+]C(c1ccccc1)C(=O)OCCC(C)C
CCOP(=S)(ON=C(c1ccccc1)C#N)OCC
CCNP(=S)(OC(=CC(=O)OC(C)C)C)OC
COc1nsnc1NS(=O)(=O)c1ccc(cc1)N
OC(=O)C(Cc1cc(Br)c(c(c1)Br)O)N
CCCc1ccc(c(c1)c1cc(CCC)ccc1O)O
OC1Cc2ccccc2N(c2c1cccc2)C(=O)N
Oc1ccc(cc1O)C1CNCc2c1ccc(c2O)O
N1CC2C(C1)(C2)c1ccc2c(c1)cccc2
COc1cc(Cc2cnc(nc2N)N)cc(c1OC)OC
CCn1cc(C(=O)O)c(=O)c2c1nc(C)cc2
CCCNC(=O)NS(=O)(=O)c1ccc(cc1)Cl
Nc1ccc(cc1)S(=O)(=O)Nc1noc(c1)C
CC(P(=O)(O)[O-])(P(=O)(O)[O-])O
OC(=O)C(c1ccc(c(c1)F)c1ccccc1)C
Cc1ccc(c(c1)OCCCC(C(=O)O)(C)C)C
CCCCNC(=O)NS(=O)(=O)c1ccc(cc1)C
CCCC(C1(CC=C)C(=O)NC(=O)NC1=O)C
O=C1N=C(NC1(c1ccccc1)c1ccccc1)O
O=C1Cc2ccccc2N(c2c1cccc2)C(=O)N
Nc1ccc(cc1)S(=O)(=O)c1ccc(cc1)N
OC(COc1nsnc1N1CCOCC1)CNC(C)(C)C
CCC(C1(CC)C(=O)NC(=NC1=O)[O-])C
CCCN(C(C(=O)Nc1c(C)cccc1C)CC)CC
Cc1cc(cc(c1CC1=NCCN1)C)C(C)(C)C
CN(CCC=C1c2ccccc2C=Cc2c1cccc2)C
OC(c1ccccc1)(C1CCCCC1)CCN1CCCC1
OC(=O)C1CCn2c1ccc2C(=O)c1ccccc1
CNCCCC12CCC(c3c1cccc3)c1c2cccc1
NC(=O)NN=Cc1ccc(o1)[N+](=O)[O-]
Nc1ccc(cc1)S(=O)(=O)Nc1nnc(s1)C
CN(Cc1cccc2c1cccc2)CC=Cc1ccccc1
OCC1OC(C(C1O)(F)F)n1ccc(nc1=O)N
CN1CCC(CC1)OC(c1ccccc1)c1ccccc1
COc1nccnc1NS(=O)(=O)c1ccc(cc1)N
Clc1ccc2c(c1)C(=NCCN2C)c1ccccc1
CN(CCOC(c1ccccn1)c1ccc(cc1)Cl)C
O=C(c1ccc(cc1)c1ccccc1)CCC(=O)O
CN(CCC(c1ccccc1Cl)(c1ccccc1)O)C
OCC1OC(C(C1O)O)n1cnc(c1O)C(=O)N
CCC(c1ccccc1)C(=O)NC(=O)NC(=O)C
CC(N1CCCCC1)COc1ccccc1Cc1ccccc1
CC(C(c1ccccc1)(C#N)CC(N(C)C)C)C
CCC(c1ccccc1)c1noc(n1)CCN(CC)CC
CCCCOc1cc(ccc1N)C(=O)OCCN(CC)CC
CC(Cn1cnc2c1c(=O)n(C)c(=O)n2C)O
COc1ccc(cc1OC1CCCC1)C1CNC(=O)C1
COc1ccc(cc1)CN(c1ncccn1)CCN(C)C
CCOC(=O)NNc1ccc(nn1)N(CC(O)C)CC
NC(=O)CS(=O)C(c1ccccc1)c1ccccc1
CN1CCC2=C(C1)c1ccccc1Cc1c2cccc1
CCCCNC(=O)NS(=O)(=O)c1ccc(cc1)N
CC(=O)NN=Cc1ccc(o1)[N+](=O)[O-]
[O-][N+](=O)c1ccc(o1)C=NNC(=N)N
CCN(C(=O)Oc1cccc(c1)C(N(C)C)C)C
CN(CC(C1(O)CCCCC1)c1ccc(cc1)O)C
CCOc1ccccc1OC(c1ccccc1)C1OCCNC1
OCC1OC(C(C1O)O)n1cnc(c1N)C(=O)N
OC(=O)C1=CC(=O)C(O1)(C)c1ccccc1

Continued on next page

CC(C(=O)OC(Cc1ccc(cc1)Cl)(C)C)N
c1ccc(cc1)CN(c1ccccc1)CC1=NCCN1
CCCNC(C(=O)Nc1c(C)csc1C(=O)OC)C
NC(=O)OCCn1c(C)ncc1[N+](=O)[O-]
CCOCCc1c(nc(n2c1ncn2)N)c1ccccc1
CN(C1CCCCC1NC(=O)c1ccc(cc1)Br)C
CCCCc1cc(OC)c2c(c1OC(=O)C)cccc2
CCC(c1ccccc1)C(=O)OCCOCCN(CC)CC
COc1ccc(cc1C(C(NC(C)(C)C)C)O)OC
OC(=O)C(c1ccc2c(c1)Cc1c2cccc1)C
O=C(OCC(Cl)(Cl)Cl)OCC(Cl)(Cl)Cl
CNCCCC1(C)CN(c2c1cccc2)c1ccccc1
Brc1ccc(cc1)NC(=O)c1cc(Br)ccc1O
OC(CC(c1ccccc1)c1ccccc1)CNC(C)C
Fc1ccc(cc1)C=Cc1cc(C)c(c(c1)C)O
Cc1c(CSCCNC(=NC#N)NCC#C)nc[nH]1
OC(=O)C(c1cccc2c1occ2c1ccccc1)C
OCC(C(C(C(C(C(=O)[O-])O)O)O)O)O
Oc1ccc(cc1)C1=Cc2c(OC1)cc(cc2)O
O=C(N1CCCCC1)C=Cc1ccc2c(c1)OCO2
CN(CCC1(C=Cc2c1cccc2)c1ccccc1)C
CCCCCCCCCCCC[n+]1ccc2c(c1)cccc2
CCN(CCn1c(=O)oc2c(c1=O)cccc2)CC
CN1CCCC1=NC(=Nc1ccccc1)N1CCOCC1
CNC(C(c1ccc(cc1)NS(=O)(=O)C)O)C
CN1CCC(C1)CN1c2ccccc2Sc2c1cccc2
COc1cc(Cc2cnc(nc2N)N)cc(c1SC)OC
NC(=O)Cc1cccc(c1N)C(=O)c1ccccc1
N#CCN1CC(=N)N(C1=O)c1ccc(cc1)Cl
CCCCC(COC(=O)C=Cc1ccc(cc1)OC)CC
CCCCC(COC(=O)c1ccc(cc1)N(C)C)CC
N#Cc1c(NCc2cccnc2)[nH]c(nc1=O)C
O=C1C(=O)C2=C1NCCCN2CCP(=O)(O)O
CCC(OC(=O)c1ccccc1C(=O)O)(C#C)C
O=C(CN1CCCC1=O)NCCN(C(C)C)C(C)C
CCN1CCC(=C(c2ccccc2)c2ccccc2)C1
CCC(=O)N(c1ccccn1)C(CN1CCCCC1)C
COc1ccc(cc1)CN(c1ccccn1)CCN(C)C
NCC(=O)NC(c1ccccc1)(Cc1ccccc1)C
CN(CCC1CN(C)C(=S)c2c(O1)nccc2)C
Cc1nc(NCc2ccccc2)c2c(n1)[nH]cc2
[O-]C(=O)CCCCCCCNC(=O)c1ccccc1O
[O-]P(=O)(OP(=O)([O-])[O-])[O-]
Nc1ccc(cc1)S(=O)(=O)Nc1snc(c1)C
n1nnc([nH]1)c1cc2ccccc2n2c1nnn2
CCCC(C1(CC=C)C(=O)NC(=S)NC1=O)C
N#CNC(=NCCSCc1csc(n1)NC(=N)N)NC
COc1cc(cc(c1OC)OC)C(=O)N1CCOCC1
ClCCN(c1c[nH]c(=O)[nH]c1=O)CCCl
CC1C2Cc3c(C1(C)CCN2CC1CC1)cccc3
C1CCN(CC1)CCC(c1ccccc1)c1ccccc1
CN(CC(CN1c2ccccc2Sc2c1cccc2)C)C
CC(=O)Oc1cc(ccc1C(=O)O)C(F)(F)F
Clc1ccc(cc1)C(c1cc2c(o1)cccc2)O
CC1CC(OC(=O)c2cccnc2)CC(C1)(C)C
CCCCCCn1c(=O)c2n(C)cnc2n(c1=O)C
CCCCN(CCCC)CCNc1onc(n1)c1ccccc1
CN(CCN(c1ccccn1)Cc1ccc(cc1)Cl)C
CN1CCC(CC1)N(c1ccccc1)Cc1ccccc1
CCCCCCCCCCCC[N+](Cc1ccccc1)(C)C
CCCOC(=O)c1sc(c(c1O)O)C(=O)OCCC
COc1cc(Cc2cnc(nc2N)N)cc(c1Br)OC
COc1ccccc1OC(=O)c1ccccc1OC(=O)C
CCN(CC(=O)Oc1ccc(cc1)NC(=O)C)CC
CCC1(C2=CCCCC2)C(=O)NC(=O)NC1=O
O=C1CCc2c(C1(C)CCN1CCCCC1)cccc2
c1ccc(cc1)N(Cc1ccccc1)CCN1CCCC1
OC(=O)Cc1cccc(c1N)C(=O)c1ccccc1
CC(N1CCCCC1)COc1ccccc1Cc1ccccc1
CN(CCN1c2ccccc2Sc2c1cc(Cl)cc2)C
O=c1[nH]c(=O)[nH]c(c1)C(=O)[O-]
OP(=O)(C(P(=O)(O)O)NC1CCCCCC1)O
OC(COc1ccccc1C1CCCC1)CNC(C)(C)C
O=C(c1ccc[nH]c1=O)Nc1c(C)cccc1C
CCC(=O)C1(CCN(CC1)C)c1cccc(c1)O
CN1CCc2c(C1)c1ccccc1n2Cc1ccccc1
COc1ccc2c(c1)c(CCNC(=O)C)c[nH]2
COc1ccc(c2c1cccc2)C(=O)CCC(=O)O
O=C(CC12CCCN2CCC1)Nc1c(C)cccc1C
OCC(=O)NCCCOc1cccc(c1)CN1CCCCC1
[O-]C(=O)CCCNC(=O)c1ccc(cc1O)Cl

Continued on next page

Table S8 – *Continued from previous page*

CCOc1ccccc1OC(c1ccccc1)C1OCCNC1
CN1CCN(CC1)c1cccc2c1oc(=O)[nH]2
Cc1ccc(c(c1)C)Sc1ccccc1N1CCNCC1
CNc1ccc(cc1F)c1nc2c(s1)cc(cc2)O
OC(=O)Cn1c2ccccc2c(=O)c2c1cccc2
Oc1ccc2c(c1)CCC1C2CCC2(C1CCC2O)C
CCCSc1ccc2c(c1)nc([nH]2)NC(=O)OC
COc1cc(CNC(=O)CCCCC=CC(C)C)ccc1O
CN(CCCN1c2ccccc2Sc2c1cc(Cl)cc2)C
ClCCN(c1ccc(cc1)CC(C(=O)O)N)CCCl
CN(CC(CN1c2ccccc2CCc2c1cccc2)C)C
CCN(C(=O)C(c1ccccc1)CO)Cc1ccncc1
ClC(C(c1ccccc1Cl)c1ccc(cc1)Cl)Cl
Nc1ccc(cc1)S(=O)(=O)[N-]c1ncccn1
Cc1cn(C2OC(C=C2)CO)c(=O)[nH]c1=O
Nc1ccc(cc1)S(=O)(=O)Nc1onc(c1C)C
OC(=O)C(c1ccc(cc1)C(=O)c1cccs1)C
CCCC(C1(CC)C(=O)NC(=NC1=O)[O-])C
Clc1[nH]c2c(n1)n(C)c(=O)n(c2=O)C
CCOC(=O)CC(C(=O)OCC)SP(=S)(OC)OC
OC(COc1ccc(cc1)CCOCC1CC1)CNC(C)C
COC(=O)CCc1ccc(cc1)OCC(CNC(C)C)O
OCC(Cn1cnc2c1c(=O)n(C)c(=O)n2C)O
CCCC(C1(CC)C(=O)NC(=NC1=O)[S-])C
CCN(CCNC(=O)c1cc(Cl)c(cc1OC)N)CC
O=C(c1cnoc1C)Nc1ccc(cc1)C(F)(F)F
OC(c1ccccc1)(C1CCCCC1)CCN1CCCCC1
COc1ccc(cc1)C(C1(O)CCCCC1)CN(C)C
O=C(N(C)C)Oc1cccc(c1)[N+](C)(C)C
OC(COc1cccc2c1CCCC2=O)CNC(C)(C)C
CC(NCC(c1ccc(cc1)NS(=O)(=O)C)O)C
CCN(C(CN1c2ccccc2Sc2c1cccc2)C)CC
COc1cc(cc(c1OC)OC)C(=O)NC1CCCNC1
CCOC(=O)[N-]c1on[n+](c1)N1CCOCC1
OC(=O)C(c1ccc(s1)C(=O)c1ccccc1)C
C=CCOc1cc(Cl)ccc1C(=O)NCCN(CC)CC
OC(=O)COc1nn(c2c1cccc2)Cc1ccccc1
FCc1nc2ccc(cc2c(=O)n1c1ccccc1C)N
CCC(C(c1ccc(cc1)O)CC)c1ccc(cc1)O
CCCCC1C(=O)NC(=O)N(C1=O)C1CCCCC1
CCNC(=O)NCCCOc1cccc(c1)CN1CCCCC1
CCCCCOc1ccc(cc1OCC)C1SC(=O)NC1=O
Brc1cc(CN(C2CCCCC2)C)c(c(c1)Br)N
Cc1ccc(cc1)C(=CCN1CCCC1)c1ccccn1
CCCCCCNC(=O)n1cc(F)c(=O)[nH]c1=O
Clc1ccc(cc1S(=O)(=O)N)S(=O)(=O)N
OCC(C(CO)O)OCn1ccnc1[N+](=O)[O-]
O=S(=O)(c1cccc2c1ccnc2)N1CCNCCC1
NC(=O)NN=C1C=C2C(O)CN(C2=CC1=O)C
OC(=O)C(c1ccc(cc1)C=C1CCCCC1=O)C
CN1CCCC(C1)CC1c2ccccc2Sc2c1cccc2
CCN(CC(=O)Nc1c(C)cccc1C(=O)OC)CC
CCC=CCC=CCC=CCC=CCC=CCCCC(=O)OCC
OP(=O)(C(P(=O)(O)O)(Cn1cncc1)O)O
CC(=CC(=NN=c1[nH]ncc2c1cccc2)C)C
OC(c1ccccc1)(c1ccccc1)CCN1CCCCC1
CCC1(CCC(C)C)C(=O)NC(=NC1=O)[O-]
OC(=O)c1ccccc1Nc1c(Cl)ccc(c1Cl)C
Cc1cc(c(c(c1)C(C)(C)C)O)C(C)(C)C
Nc1ccc(cc1)S(=O)(=O)Nc1nccc(n1)C
ClN(S(=O)(=O)c1ccc(cc1)C(=O)O)Cl
CCc1nnc(s1)NS(=O)(=O)c1ccc(cc1)N
CN1CCCC(C1)CN1c2ccccc2Sc2c1cccc2
CN(CCCC1c2ccccc2Nc2c1cc(Cl)cc2)C
CN1CCN(CC1)C1=Nc2ccccc2Sc2n1ccn2
CCN(CCNC(=O)c1ccc(cc1)NC(=O)C)CC
CCOc1ccc(c2c1nccc2)S(=O)(=O)[O-]
OCC1OC(CC1F)n1cc(C)c(=O)[nH]c1=O
O=C1NC2=Nc3c(CN2C1)c(Cl)c(cc3)Cl
CN1CCC(=C2c3ccccc3Oc3c2cccn3)CC1
c1ccn(c1)N=C1C=CC(=Nn2cccc2)C=C1
CN(CC(CC1c2ccccc2CCc2c1cccc2)C)C
COc1cccc(c1)C1(O)CC(O)CCC1CN(C)C
OC(=O)C(=O)Nc1cccc(c1)c1nnn[nH]1
NC(=N)NN=C1C=C2C(O)CN(C2=CC1=O)C
CCN(C(CN1c2ccccc2Sc2c1cccc2)C)CC
NC(=O)Nc1cc(ccc1O)C(CNC(C)(C)C)O
OC(=O)C1CCN1C12CC3CC(C2)CC(C1)C3
COC(=S)NCCn1c(C)ncc1[N+](=O)[O-]
CN(C(C(c1ccccc1)O)C)CC=Cc1ccccc1
OC(=O)Cc1cc(n(c1C)c1ccc(cc1)Cl)C

Continued on next page

CN(CCC1c2ccc(cc2CCc2c1nccc2)Cl)C
CNP(=O)(Oc1ccc(cc1Cl)C(C)(C)C)OC
OC(=O)CCn1c2ccccc2c(c1C)Cn1cncc1
OC(=O)Cc1cc(C)ccc1Nc1c(F)cccc1Cl
CNC(=O)c1ccc(c(c1)S(=O)(=O)NC)Cl
[O-][n+]1ccccc1SSc1cccc[n+]1[O-]
Oc1cc(CCNCCCCCCNCCc2ccccc2)ccc1O
CC(CN(CC(O)C)CCN(CC(O)C)CC(O)C)O
OCCCC(=O)NCC(c1cccc(c1)OC)(CC)CC
C1CN(CCC1C1CCN(CC1)CC1OC1)CC1OC1
CCc1nc(N)nc(c1c1ccc(c(c1)Cl)Cl)N
CCOC(=O)C=C1SC(C(=O)N1C)N1CCCCC1
CN(CCCn1c(=O)c2ccccc2c2c1cccc2)C
CCC(C1(O)CCC(CC1)c1ccccc1)C(=O)O
CCCCN=C(c1ccccc1Cl)c1cc(Cl)ccc1O
O=C(NC1=NC(=O)CN1C)Nc1cccc(c1)Cl
OCC1OC(CC1O)n1cc(F)c(=O)[nH]c1=O
OC(c1ccccc1)C1CCN(CC1)CCc1ccccc1
CC1CC(OC(=O)c2ccccc2O)CC(C1)(C)C
COC(=O)C(c1c[nH]c2c1cc(OC)cc2)CN
CCN(CCOC(=O)C1(CCCCC1)CCC(C)C)CC
CC1CC2N(O1)C(=O)c1c(O2)ccc(c1)Cl
Cn1c2ccccc2c2c1C1CC3CCCCC3CN1CC2
CNCCC=C1c2ccccc2C2C(c3c1cccc3)C2
OC(=O)CC(c1ccc(o1)c1ccc(cc1)Cl)O
CCCOc1ccc2c(c1)nc([nH]2)NC(=O)OC
CCCCc1ccc2c(c1)nc([nH]2)NC(=O)OC
CCc1[nH]c(=O)[nH]c1C(=O)c1ccncc1
CC(c1ccc(c(c1)Cl)N1CC=CC1)C(=O)O
CN1CCC(=C2c3ccsc3CCc3c2cccc3)CC1
Brc1[nH]c2c(n1)n(C)c(=O)n(c2=O)C
COC(=O)c1nc2c(o1)c1ncccc1c(c2)Cl
Oc1ccc2c(c1)CC[N+]1=C2CCC2C1CCC2
CCCN(C1CCc2c(C1)cccc2O)CCc1cccs1
CC(=O)NC(C(=O)O)CSC(=O)c1ccccc1O
O=S(c1nc2c([nH]1)cccc2)Cc1ccccn1
OCC1OC(O)C(C(C1O)O)NC(=O)N(N=O)C
CC(=O)Nc1ccc(cc1)C(=O)Nc1ccccc1N
CCOC(=O)C1(CCC=CC1N(C)C)c1ccccc1
COc1cc2c(cc1OC)CCN(CC2c1ccccc1)C
CCOC(=O)C(CC(=O)OCC)(CC(=O)OCC)O
OC(=O)c1ccccc1c1ccc(cc1)C(F)(F)F
c1ccc(cc1)N1CCN(CC1)CCc1nnn[nH]1
OCC1OC(CC1O)n1cc(C)c(=O)[nH]c1=O
CCN(CCNC(=O)c1cc(Br)c(cc1OC)N)CC
Oc1ccc2c(c1)CCC1C2CCC2(C1CCC2O)C
OC(COc1cccc2c1cc([nH]2)C)CNC(C)C
CCN(c1[nH]c(nc(=NN)n1)N(CC)CC)CC
Cc1cnc(nc1)NS(=O)(=O)c1ccc(cc1)N
OC(C(COS(=O)(=O)C)O)COS(=O)(=O)C
O=C(NCc1ccccc1)CCNNC(=O)c1ccncc1
ONC(=O)CS(=O)C(c1ccccc1)c1ccccc1
CN1CCC(=C2c3ccccc3Sc3c2cccc3)CC1
CN1CCc2c3C1Cc1ccc(c(c1c3ccc2)O)O
CCCCOCCOC(=O)C(c1ccccc1)N1CCCCC1
CCOP(=S)(Oc1nc(Cl)c(cc1Cl)Cl)OCC
COP(=S)(Oc1ccc(cc1)S(=O)(=O)N)OC
CCOP(=S)(Oc1cc(C)nc(n1)C(C)C)OCC
CCOCCn1c(nc2c1cccc2)N1CCCN(CC1)C
OCC1OC(C(C1O)O)n1cnc2c1nc(F)nc2N
O=C1C(c2ccc(cc2)F)C(=O)c2c1cccc2
OC(=O)Cn1cnc2c1c(=O)n(C)c(=O)n2C
O=C(C(Oc1ccccc1)(C)C)OCCN1CCOCC1
CCCCCC(C=CC=CCC=CCC=CCCCC(=O)O)O
Nc1cnc(c(n1)N)c1cc(Cl)cc(c1Cl)Cl
COc1ccc2c(c1)CN(CCS2)C(=O)C(=O)O
COc1nccc(c1)NCCNC(=O)C=CC(F)(F)C
O=C1CC2C(C1(CC2)CS(=O)(=O)O)(C)C
OCC1OC(C(C1O)O)n1cnc2c1nc[nH]c2=O
Oc1ccc2c(c1)CCC1C2CCC2(C1CCC2=O)C
Fc1ccc(c(c1)F)c1ccc(c(c1)C(=O)O)O
OC(=O)C(c1cccc(c1)C(=O)c1ccccc1)C
CN1C(=O)CCS(=O)(=O)C1c1ccc(cc1)Cl
Nc1nc(C)c(c(n1)N)c1ccc(c(c1)Cl)Cl
Nc1nc(N)c2c(n1)nc(c(n2)c1ccccc1)N
O=C1C(CCc2c1ccccc1n2C)Cn1ccnc1C
O=C1Nc2ccc(cc2C(=NC1O)c1ccccc1)Cl
CCC(=C(c1ccc(cc1)O)CC)c1ccc(cc1)O
CN(CCc1c[nH]c2c1cc(cc2)Cn1cncn1)C
CCC1(C(=O)NC(=O)N(C1=O)C)c1ccccc1
CCC1(C(=O)NC(=NC1=O)[O-])c1ccccc1

Continued on next page

CCN(CCOC(=O)C1(CCCCC1)C1CCCCC1)CC
CN(CCC=C1c2ccccc2Sc2c1cc(Cl)cc2)C
CN(CCCN1c2ccccc2CCc2c1cc(Cl)cc2)C
NCCON=C(c1ccc(cc1)C(F)(F)F)CCCCOC
O=C1OCCN1N=Cc1ccc(o1)[N+](=O)[O-]
CCn1nc(C(=O)O)c(=O)c2c1cc1OCOc1c2
[O-]C(=O)Cc1ccccc1Nc1c(Cl)cccc1Cl
COc1ccc2c(c1)ccc(c2)C(C(=O)[O-])C
OC(c1ccccc1)C(=O)OC1CC2CCC(C1)N2C
O=C1NC(=O)C(C(=O)N1C)(C)C1=CCCCC1
N#CC(=C1SCC(S1)c1ccccc1Cl)n1cncc1
CC(C(=NO)C)NCC(CNC(C(=NO)C)C)(C)C
COc1ncnc(c1)NS(=O)(=O)c1ccc(cc1)N
Cc1ccc(cc1)C=Nn1c(=S)scc1c1ccccc1
CCOc1ccc2c(c1)c(N)c1c(n2)cc(cc1)N
Oc1ccc(cc1)CCNC(C(c1ccc(cc1)O)O)C
OC(c1ccccc1)(c1ccccc1)CCCN1CCCCC1
OC(=O)C1CCc2c1cc(Cl)c(c2)C1CCCCC1
OC(c1cc(Cl)c(c(c1)Cl)N)CNC(C)(C)C
CN(CCCOc1nn(c2c1cccc2)Cc1ccccc1)C
CN1CCN(CC1)C1=Nc2ccccc2Cc2c1cccc2
CCN(CCOc1ccccc1C(=O)CCc1ccccc1)CC
c1ccc(cc1)C1(CC1C1=NCCN1)c1ccccc1
OC(COc1ccccc1OCC1CCCO1)CNC(C)(C)C
Cc1ccc2c(c1)C13CCCCC3C(C2)N(CC1)C
CCOC(=O)c1ccc(cc1)NC(=O)CN1CCCCC1
CN(CCOc1cc(C)c(cc1C(C)C)OC(=O)C)C
OC(=O)c1ccccc1Nc1cccc(c1)C(F)(F)F
Cc1cc2CCCS(=O)(=O)c2cc1S(=O)(=O)N
CC(=O)Oc1cc(C)c(c2c1cccc2)OC(=O)C
OCC1OC(CC1O)n1cc(Br)c(=O)[nH]c1=O
CC(NC(C)(C)C)CC(c1ccccc1)c1ccccc1
[O-]CC(CCn1cnc2c1[nH]c(N)nc2=O)CO
CN1CCC2=C(C1)C(c1ccccc1)c1c2cccc1
CCn1cc(C(=O)O)c(=O)c2c1cc1OCOc1c2
COn1cc(C(=O)O)c(=O)c2c1cc1OCOc1c2
OC(COc1ccc(cc1)COCCOC(C)C)CNC(C)C
OC(=O)Cc1ccc(n1C)C(=O)c1ccc(cc1)C
CCN(CCCC(Nc1ccnc2c1ccc(c2)Cl)C)CC
COc1ccc(nn1)NS(=O)(=O)c1ccc(cc1)N
O=C1NCCN1N=Cc1ccc(o1)[N+](=O)[O-]
COc1cnc(nc1)NS(=O)(=O)c1ccc(cc1)N
CCN(CCCC(Nc1cc(OC)cc2c1nccc2)C)CC
COc1cc(CCNCC(c2ccc(cc2)O)O)ccc1OC
CCCN(C1Cc2cc(O)cc3c2c(C1)ccc3)CCC
CCOC(=O)c1cnc2c(c1NN=C(C)C)cnn2CC
O=C1CCC2(C(=O)N1)CCC(=O)c1c2cccc1
CNCCCC1(C)c2ccccc2N(C1=O)c1ccccc1
Clc1ccc(c(c1)Cl)C1(OCCO1)Cn1cncn1
O=C(NP(=O)(N1CC1)N1CC1)OCc1ccccc1
O=C1C(c2ccc(cc2)Br)C(=O)c2c1cccc2
COC(=O)NN=Cc1cn(=O)c2c(n1=O)cccc2
CNC(=O)COc1ccccc1OCC(CNC(C)(C)C)O
OC(COc1ccc(cc1)OCCOCC1CC1)CNC(C)C
Oc1cccc(c1)C12CCCCC2CN(CC1)CC1CC1
OCC1OC(C(C1O)F)n1cnc2c1nc(Cl)nc2N
ClCC(O[N+](=O)[O-])CO[N+](=O)[O-]
CN(C(c1ccccc1)CCOc1cccc2c1cccc2)C
Oc1ccc2c(c1)C13CCCCC3C(C2)N(CC1)C
NC(=O)N1CC(C1)Oc1cccc(c1)C(F)(F)F
OCC1OC(CC1O)n1cc(CC)c(=O)[nH]c1=O
CN(CCC=C1c2ccccc2CN(c2c1cccc2)C)C
CCN(CC(c1ccccc1)OC(=O)c1ccccc1)CC
OC(=O)C(c1ccc(c(c1)Cl)C1CCCCC1)Cl
O=C1OC(CN1C)CCN1CCC(=CC1)c1ccccc1
COc1ccc(cc1OC1CCCC1)C(=NOC(=O)N)C
CCOC(=O)Nc1ccc(nc1N)NCc1ccc(cc1)F
Brc1cc(Br)c(c(c1)C(=O)NCC1CCCO1)O
CN(CC#CC=C1c2ccccc2C=Cc2c1cccc2)C
CN(CCCN1c2ccccc2CC(=O)c2c1cccc2)C
OCC1C(CO)CC1n1cnc2c1nc(N)[nH]c2=O
CC1CCC(C(C1)OC(=O)c1ccccc1N)C(C)C
Brc1cc(Br)c(c(c1)C(=O)Nc1ccccc1)O
OCC1OC(C(C1O)O)n1cnc2c1nc(N)nc2OC
CN(CCC=C1c2cc(Cl)ccc2CCn2c1ccc2)C
CCC(C(COC(=O)N)(COC(=O)NC(C)C)C)C
CCN1C(=CC(=O)OCC)SC(C1=O)N1CCCCC1
[O-]P(=O)(C(P(=O)(O)O)Cc1ccccn1)O
N#CC(C(=O)c1cccn1C)C(=O)Nc1ccccc1
O=C(Nc1c(C)cccc1Cl)C=C1SCC(=O)N1C
OCC1OC(CC1F)n1cc(Cl)c(=O)[nH]c1=O

Continued on next page

Table S8 – *Continued from previous page*

CCOC(=O)c1ccc(cc1)N(CC(O)C)CC(O)C
O=C1Nc2c(C1=Cc1[nH]c(cc1C)C)cccc2
NCCC(c1ccccc1)Oc1ccc(cc1)C(F)(F)F
CCCCCCCCCCCC(=O)OCC(C1OCC(C1O)O)O
Nc1ccc(cc1)S(=O)(=O)Nc1ccc(nn1)Cl
COC(=S)NCCn1c(CC)ncc1[N+](=O)[O-]
CCCCCCCCNC(C(c1ccc(cc1)SC(C)C)O)C
CCn1c2ccccc2c2c1C(C)(SCC2)CCN(C)C
O=C1NC(=O)C2=C(C1=Cc1ccccc1)CCCC2
CSc1ccc2c(c1)c(=O)n(cn2)C=CC(=O)O
Brc1ccc(cc1)NC(=O)c1sc(c(c1)Br)Br
Nc1n[n+]([O-])c2c([n+]1[O-])cccc2
O=C(N1CCCC1)NS(=O)(=O)c1ccc(cc1)C
CCCCNc1c(C(=O)OCC)c(C)nc2c1cnn2CC
OC(=O)c1cnccc1Nc1cccc(c1)C(F)(F)F
CCC(=O)OC1(CC(C)N(CC1C)C)c1ccccc1
O=C1C(c2ccc(cc2)Cl)C(=O)c2c1cccc2
O=S1(=O)CCN(CN1)CN1CCS(=O)(=O)NC1
COc1cc(OC)cc(c1C(=O)CCCN1CCCC1)OC
ClCCN(C(=O)NC(P(=O)(OCC)OCC)C)N=O
OC(=O)C(c1cccc(c1)C(=O)c1ccccc1)C
OC(=O)c1c(O)c(nc2c1cccc2)c1ccccc1
Brc1ccc2c(c1)C(=O)C(C2=O)c1ccccc1
CCN(CCC1(CCC(=O)NC1=O)c1ccccc1)CC
CC(N1CCOC(C1)c1cccc(c1)C(F)(F)F)C
OCC(COP(=O)(OCC[N+](C)(C)C)[O-])O
OCCOCCN1CCC(CC1)(C(=O)CC)c1ccccc1
O=C(NS(=O)(=O)c1ccc(cc1)N)C=C(C)C
COc1ccc(cc1)C1C(=O)c2c(C1=O)cccc2
CN1CCC(=C2c3ccccc3CCc3c2nccc3)CC1
CCCOc1ccc(cc1NC(=O)C)[N+](=O)[O-]
ClC(P(=O)(O)[O-])(P(=O)(O)[O-])Cl
CN(=O)(CCCN1c2ccccc2CCc2c1cccc2)C
CCCCCCCCCCCCCC[N+](Cc1ccccc1)(C)C
OC(=O)c1cccnc1Nc1cccc(c1)C(F)(F)F
OP(=O)(C(P(=O)(O)O)(Cc1cccnc1)O)O
OC(=O)C1N2C(=O)CC2S(=O)(=O)C1(C)C
Nc1ccc(cc1)S(=O)(=O)Nc1cncc(n1)Cl
COc1cc(ccc1OC)C(N(C)C)CCCc1ccccc1
COc1cc(ccc1OC)N(C(C)C)CCCc1ccccc1
Oc1ccc(c(c1)O)C1=NC(CS1)(C)C(=O)O
CCOC(=O)Nc1ccc(cc1N)NCc1ccc(cc1)F
N#Cc1nccc(c1)c1n[nH]c(n1)c1ccncc1
Oc1ccc(cc1)C1Oc2ccc(cc2C2C1CCC2)O
CCCN1CCC(CC1)c1cccc(c1)S(=O)(=O)C
CC(c1cccc(c1C)C)c1c[nH]c(=S)[nH]1
COc1ccc(cc1)N(c1nc(C)nc2c1cccc2)C
NC1CCC(c2c1cccc2)c1ccc(c(c1)Cl)Cl
CNc1ccc(c(n1)F)c1cc2c(o1)ccc(c2)O
OCC(c1ccccc1)C(=O)OC1CC2CCC(C1)N2C
COc1ccc2c(c1)sc(n2)NC(=O)Nc1ccccc1
CNCCC(c1ccccc1)Oc1ccc(cc1)C(F)(F)F
Clc1cc2NCNS(=O)(=O)c2cc1S(=O)(=O)N
Clc1ccc(cc1)C1(O)c2ccccc2C2=NCCN12
CCCN(S(=O)(=O)c1ccc(cc1)C(=O)O)CCC
Cc1ccc(cc1)N(c1cccc(c1)O)CC1=NCCN1
OC(COc1cccc2c1CCC(=O)N2)CNC(C)(C)C
Oc1ccc(cc1)C1CNCCc2c1cc(O)c(c2Cl)O
CC(CCc1ccc(cc1)O)NCCc1ccc(c(c1)O)O
CCCNCC(COc1ccccc1C(=O)CCc1ccccc1)O
CCCC(C1(CC=C)C(=O)NC(=NC1=O)[S-])C
CNC1CCC(c2c1cccc2)c1ccc(c(c1)Cl)Cl
COc1ccc2c(c1)C13CCCCC3C(C2)N(CC1)C
CC=C(C(=CC)c1ccc(cc1)O)c1ccc(cc1)O
CN(CCOC(=O)C(C1(O)CCCC1)c1ccccc1)C
Cc1cc(c(c(c1CC1=NCCN1)C)O)C(C)(C)C
Cc1cc2c(o1)c(C)c1c(c2)c(C)cc(=O)o1
CC(=O)SCC(C(=O)O)CC(=O)c1ccc(cc1)C
OC(=O)Cc1ccc2c(c1)N(C)c1c(S2)cccc1
CCN1CCC(=C2c3ccccc3CCc3c2cccc3)C1C
O=C1CN=C(c2c(N1)ccc(c2)Br)c1ccccn1
CC1OC(C(C1O)O)n1cc(F)c(=O)[nH]c1=O
CCCC(C1(CC=C)C(=O)[N-]C(=O)NC1=O)C
CN(CCOC1=Cc2ccccc2Sc2c1cc(Cl)cc2)C
CC(Oc1ccc2c(c1)occ(c2=O)c1ccccc1)C
CCOc1cc(OCC)c(cc1C(=O)CCC(=O)O)OCC
CCC1(O)CCC2C1(C)CCC1C2CCC2=CCCCC12
CCCCC(C=C(C=CC=CC(=O)N1CCCC1=O)C)O
CC(NC(C(c1ccc(cc1)O)O)C)CCc1ccccc1
Clc1ccc(cc1)C(c1ccccc1)OCCN1CCCCC1
OC(=O)C(c1ccc2c(c1)Cc1c(O2)nccc1)C

Continued on next page

Table S8 – *Continued from previous page*

CC(NC(C(c1ccc(cc1)O)O)C)COc1ccccc1
CC(OC(=O)C(=C1SC=CS1)C(=O)OC(C)C)C
O=S1(=O)CCCCN1c1ccc(cc1)S(=O)(=O)N
[O-]C(=O)Cc1cccc(c1N)C(=O)c1ccccc1
OCC=C(CCC=C(CCC=C(CCC=C(C)C)C)CO)C
CC(=O)OC1C(=O)OC2C1OC(=O)C2OC(=O)C
OC(=O)C(c1ccc(c2c1cccc2)C1CCCCC1)C
CCCCNc1ccc(cc1)C(=O)OCC[NH+](CC)CC
O=C1N=C(NC1(c1ccccc1)c1ccccc1)[O-]
OCCN(CCCC(Nc1ccnc2c1ccc(c2)Cl)C)CC
Clc1cccc(c1)COc1ccccc1C(=C)n1cncc1
CN(Cc1cccc2c1cccc2)CC=CC#CC(C)(C)C
C=CCN1CCC23C(C1Cc1c3cc(cc1)O)CCCC2
CN(C=Nc1ccc(cc1C)C)C=Nc1ccc(cc1C)C
Nc1ccc(cc1)S(=O)(=O)Nc1oc(c(n1)C)C
CN(CCCN1c2ccccc2C(c2c1cccc2)(C)C)C
CC(CC(C1(CCC1)c1ccc(cc1)Cl)N(C)C)C
O=C(c1cnn2c1nccc2c1ccncc1)c1ccccn1
CCN1CCCC1CNC(=O)c1c(OC)ccc(c1OC)Br
C1CCC2N(C1)CC1c3c2cccc3CCc2c1cccc2
OC(=O)Cc1ccc(cc1)OCCNCC(c1ccccc1)O
OCC1C=CC(C1)n1cnc2c1nc(N)nc2NC1CC1
CCN(CCOC(=O)C(c1ccccc1)c1ccccc1)CC
OC(=O)c1snc(c1N)c1cccc(c1)C(F)(F)F
CCN(CCOC(=O)c1cccc(c1O)c1ccccc1)CC
CCC1(O)CCC2C1(C)CCC1C2CC=C2C1CCCC2
OC(=O)COc1ccc2c(c1Br)onc2c1ccccc1F
CCCC(=O)OC(P(=O)(OC)OC)C(Cl)(Cl)Cl
CC(NCC(c1ccc(cc1)O)O)CCc1ccc(cc1)O
CN(CCCSc1ccccc1NC(=O)C=Cc1ccccc1)C
CC1CC(OC(=O)C2CCC(=O)N2)CC(C1)(C)C
COc1cccc2c1n(=O)c1cccc(c1n2=O)[O-]
Cc1ccccc1N1CCN(CC1)CCc1nnc2n1CCCC2
Clc1ccc(cc1)C(c1ccccn1)(C1=NCCN1)O
O=C(C1(CCCCC1)C1CCCCC1)OCCN1CCCCC1
[N-]=[N+]=CC(=O)CCC(C(=O)O)NC(=O)C
Oc1ccc2c(c1)CC=C1C2CCC2(C1CCC2=O)C
CCOC(=O)C(=C(c1ccccc1)c1ccccc1)C#N
CC(=O)Oc1ccc(cc1C(=O)O)c1ccc(cc1)F
OC(=O)c1cccnc1Nc1cccc(c1C)C(F)(F)F
CNC(=O)N1CC(C1)Oc1cccc(c1)C(F)(F)F
CCOP(=O)(OCC)CCCCCCOc1ccc(cc1Cl)OC
CNC1Cc2c(CC1)[nH]c1c2cc(cc1)C(=O)N
[O-][N+](=O)c1ccc(o1)c1csc2=NCCn12
OC(=O)CCC(=O)c1ccc2c(c1)c1ccccc1o2
OC(=O)Cc1ccc2c(c1)C(=O)c1ccccc1CO2
O=C1CCC(C(=O)N1)N1Cc2c(C1=O)cccc2N
CC(CCCCn1c(=O)c2n(C)cnc2n(c1=O)C)O
Clc1ccc(nn1)N1CCN(CC1)CC=Cc1ccccc1
[O-][N+](=O)c1cnc(n1CCOP(=O)(O)O)C
CCOC(=O)NP(=O)(N1CC1(C)C)N1CC1(C)C
CCn1cc(C(=O)[O-])c(=O)c2c1nc(C)cc2
Clc1ccc(cc1Cl)Nc1cc[n+]2c(c1)cccc2
CNCC(CC12CCC(c3c1cccc3)c1c2cccc1)O
c1ccc(cc1)OCc1[nH][nH]c2c(n1)cncc2
CCN(CCSC(=O)N(c1ccccc1)c1ccccc1)CC
CCC(c1c(=O)oc2c(c1O)cccc2)c1ccccc1
CCCC1(OCCc2c1[nH]c1c2cccc1)CC(=O)O
OCC1OC(C(C1O)O)c1n[nH]c(c1O)C(=O)N
CN1CCC(C1)OC(c1ccc(cc1)Cl)c1ccccc1
CCn1nc(c2c1C(=NCC(=O)N2)c1ccccc1)C
O=C(Nc1c(C)cccc1Cl)CCN1CCCC2C1CCC2
COc1cc(cc(c1OC)OC)C(=O)C=CN1CC=CC1
CCCCCCCOC1C(OC2C1OC(O2)(C)C)C(CO)O
Nc1ccc(cc1)S(=O)(=O)NC(=O)c1ccccc1
CCN(CCN=S(=O)(c1ccccc1)c1ccccc1)CC
CNCCCC1(OC(c2c1cccc2)(C)C)c1ccccc1
COCCOc1c(OC)cc(cc1OC)Cc1cnc(nc1N)N
CCN(CCSC(=O)C(c1ccccc1)c1ccccc1)CC
OC(=O)Cc1ccc2c(c1)SCc1c(C2=O)cccc1
CCCCNC(=O)[N-]S(=O)(=O)c1ccc(cc1)C
CCCCC(CCC(OS(=O)(=O)[O-])CC(C)C)CC
CCN(CCNC(c1ccccc1)C(=O)OCCC(C)C)CC
CC(N(C(C)C)CCC(c1ccccc1)c1ccccc1)C
CC(c1ccccc1)NCCC(c1ccccc1)c1ccccc1
CN(CCOC(c1ccc(cc1)Cl)(c1ccccc1)C)C
O=C1NOCC1N=Cc1ccc(cc1)C=NC1CONC1=O
CCN(CCCN1c2ccccc2Sc2c1cc(Cl)cc2)CC
CCCCSc1ccc(cc1)C(c1ccccc1)SCCN(C)C
OC(=O)CCCCCCNC1c2ccccc2CCc2c1cccc2
O=C(c1cccs1)Nc1ncc(s1)[N+](=O)[O-]

Continued on next page

OC1OC2OC3(C)CCC4C2(C(C1C)CCC4C)OO3
CCN(CCn1cnc2c1c(=O)n(C)c(=O)n2C)CC
C[N+](CCOC(c1ccccc1)Cc1ccccc1)(C)C
Clc1ccc(cc1)CC(=CCN1CCCC1)c1ccccc1
COP(=O)(SCn1c(=O)oc2c1ncc(c2)Cl)OC
OC(COc1cccc2c1oc(c2)C(=O)C)CNC(C)C
CN1CCC(=C2c3ccccc3C=Cc3c2cccc3)CC1
CN(CCOC(c1ccc(cc1)Br)(c1ccccc1)C)C
OCCN(CCCC(Nc1ccnc2c1ccc(c2)Cl)C)CC
O=C1CCC2C(=C1)CCC1C2CCC2(C1CCC2O)C
O=C1CN=C(c2c(N1)ccc(c2)Cl)c1ccccc1
COP(=S)(SCN1C(=O)c2c(C1=O)cccc2)OC
CC(NC1CCC(CC1)(c1ccccc1)c1ccccc1)C
CC(Oc1ccccn1)COc1ccc(cc1)Oc1ccccc1
Clc1ccc(cc1)SC(P(=O)(O)O)P(=O)(O)O
Fc1ccccc1c1onc(n1)c1cccc(c1)C(=O)O
NC(=O)c1cccc2c1nc([nH]2)C1(C)CCCN1
OC(c1ccccc1)(c1ccccc1)C1CN2CCC1CC2
OCC1CN(CC1O)Cc1c[nH]c2c1nc[nH]c2=O
CCOc1ccc2c(c1)[nH]c(n2)SCCN1CCOCC1
OCC1(CO)CC1=Cn1cnc2c1nc(N)[nH]c2=O
[O-]C(=O)c1ccccc1Nc1c(Cl)ccc(c1Cl)C
Oc1ccc2c(c1)CCC1C2CCC2(C1CC(C2O)O)C
CNC(=O)Oc1ccc2c(c1)C1(C)CCN(C1N2C)C
CC1CC(OC(=O)C(c2ccccc2)O)CC(C1)(C)C
Clc1ccc2c(c1)C(=NCC(=O)N2C)c1ccccc1
Clc1ccc2c(c1)C(=NCc1n2cnn1)c1ccccc1
CCC1(OCCc2c1[nH]c1c2cccc1CC)CC(=O)O
CCCCCCCCOc1ccc(c(c1)O)C(=O)c1ccccc1
CN1CCN(CC1)C1=Nc2ccccc2Nc2c1cc(s2)C
OC(=O)CCc1nc(c(o1)c1ccccc1)c1ccccc1
CC(=O)CCCCn1c(=O)c2n(C)cnc2n(c1=O)C
CCCN1CC(CSC)CC2C1Cc1c[nH]c3c1c2ccc3
Clc1cc2N=CNS(=O)(=O)c2cc1S(=O)(=O)N
COc1c(OC)ncnc1NS(=O)(=O)c1ccc(cc1)N
O=C1CC2C(C1(CC2)CS(=O)(=O)[O-])(C)C
COC(=O)C(c1ccccc1Cl)N1CCc2c(C1)ccs2
CN1C2CCC1CC(C2)OC(c1ccccc1)c1ccccc1
CN(CCC=C1c2ccccc2C(c2c1cccc2)(C)C)C
CC(Cc1ccc(cc1)C(C(=O)OCc1ccccn1)C)C
CC(c1nnc(s1)NS(=O)(=O)c1ccccc1)(C)C
CCN(CCOCCOC(=O)C1(CCCC1)c1ccccc1)CC
OC1CCC2C1(C)CCC1C2CCC2C1(C)CC1SC1C2
CN(CCn1c2ccccc2n(c2c(c1=O)cccc2)C)C
COc1cc(CCNCC(COc2cccc(c2)C)O)ccc1OC
CCS(=O)(=O)CCn1c(C)ncc1[N+](=O)[O-]
CCOC(=O)c1c(N)sc2c1CCN(C2)Cc1ccccc1
CCCCC(CNC(=O)CC(OC(=O)CCC(=O)O)C)CC
Nc1ccc(cc1)S(=O)(=O)Nc1cc(C)nc(n1)C
CCN(CCOCCOC(=O)C(c1ccccc1)(CC)CC)CC
C#CC1(O)CCC2C1(C)CCC1C2CCC2=CCCCC12
CCP(=O)(Oc1ccc(cc1)[N+](=O)[O-])OCC
NCCC(=O)[N-]C(C(=O)[O-])Cc1[nH]cnc1
O=C(NS(=O)(=O)c1ccc(cc1)Cl)NN1CCCC1
CC(C(=O)c1ccc(cc1)C(F)(F)F)CN1CCCC1
[O-]C(=O)C(CC(=O)[O-])(CC(=O)[O-])O
CC(Cc1ccccc1)NCCC(c1ccccc1)c1ccccc1
CNC(=O)Oc1ccc2c(c1)C1(C)CCN(C1N2C)C
Nc1ccc(cc1)S(=O)(=O)Nc1nnc(s1)C(C)C
O=C(NS(=O)(=O)c1ccc(cc1)C)NC1CCCCC1
Nc1ccc(cc1)S(=O)(=O)Nc1nc(C)cc(n1)C
Cc1cc(Cl)c(cc1S(=O)(=O)N)S(=O)(=O)N
O=C1OC2OC3(C)CCC4C2(C(C1C)CCC4C)OO3
COC1OC2OC3(C)CCC4C2(C(C1C)CCC4C)OO3
O=C(Cn1ccnc1[N+](=O)[O-])NCc1ccccc1
Nc1ncc(nn1)C=Cc1ccc(o1)[N+](=O)[O-]
CCn1ccc(nc1=O)NS(=O)(=O)c1ccc(cc1)N
Fc1cc(F)c2c(c1)c1CC(CCc1[nH]2)N(C)C
CN1CC2Cc3c(OC2(C(C1)C)O)ccc1c3cccc1
CCCCCNC(=N)NN=Cc1c[nH]c2c1cc(OC)cc2
CN(NC(=O)c1ccc(c(c1)S(=O)(=O)N)Cl)C
OCC(C1OC2C(C1OCCCN(C)C)OC(O2)(C)C)O
NCCOCc1c(C)oc2c1cc1c(C)cc(=O)oc1c2C
COc1ccc(cc1)C(=O)c1ccc2n1CCC2C(=O)O
COc1ccc(cc1)c1nc(C)nnc1c1ccc(cc1)OC
Oc1ccc(cc1)CCCCNCC(c1ccc(c(c1)O)O)O
Clc1ccc2c(c1)C1CN(CC1c1c(O2)cccc1)C
CCN(c1ccc(c(c1)C)NC(=O)c1ccccc1N)CC
Nc1ccc(cc1)S(=O)(=O)N1CC2CCC(C1)CC2
O=C1CN=C(c2c(N1)sc1c2CCCC1)c1ccccc1
C[NH+](CCOC(c1ccc(cc1)Br)c1ccccc1)C

Continued on next page

Table S8 – *Continued from previous page*

OCCC(=C(N(Cc1cnc(nc1N)C)C=O)C)SSCCC
COc1cc(cc(c1OC)OC)C(=O)NCCCCCC(=O)O
CCCCCCCCCCCCCCCC[N+](Cc1ccccc1)(C)C
O=C(C(c1ccsc1)C1CCCCC1)OCCN1CCCCCC1
Clc1cccc(c1)C1(O)C2=NCCCN2c2c1cccc2
C#CC1(O)CCC2C1(C)CCC1C2CC=C2C1CCCC2
OC(=O)C(Oc1ccc(cc1)C1CC1(Cl)Cl)(C)C
OCC1OC(C(C1O)F)n1cc(C)c(=O)[nH]c1=O
CCCCCCC[N+](CCCCc1ccc(cc1)Cl)(CC)CC
Cc1c(Nc2ncccc2C(=O)OCC(CO)O)cccc1Cl
CN1CCC(=C(c2ccccn2)c2ccc(cc2)Cl)CC1
OC(C1CC1c1ccncc1)(c1ccccc1)c1ccccc1
SCC(C(=O)N1CC(CC1C(=O)O)Sc1ccccc1)C
Cn1c(=O)n(C)c2c(c1=O)n(cn2)CC1OCCO1
CC(CC1CCCCC1)NCCC(c1ccccc1)c1ccccc1
CSc1ccc(cc1)C(=O)c1[nH]c(=O)[nH]c1C
CN(CCCn1cc(c(n1)c1ccccc1)c1ccccc1)C
OCCn1c(ncc1[N+](=O)[O-])c1ccc(cc1)F
[O-]C(=O)c1oc2c(c1)cc(cc2)Cc1cccnc1
COC1C2Cc3c(C1(C)CCN2CC1CC1)cc(cc3)O
NC1=NN(C(=O)C1)C(c1ccc(c(c1)Cl)Cl)C
CC(C(C)(C)C)N=C(Nc1ccc(cc1)C#N)NC#N
COC(=O)C1(CCCCC1c1ccccc1)C(CN(C)C)C
CCN(C(=O)N)N=Cc1ccc(o1)[N+](=O)[O-]
O=CNNc1scc(n1)c1ccc(o1)[N+](=O)[O-]
NC(=N)c1ccc(o1)c1ccccc1[N+](=O)[O-]
Cc1cc2c(cc1C)c(=O)c(=N(=O)[O-])c2=O
Cc1ccc(o1)Cn1c(NC2CCNCC2)nc2c1nccc2
CN(CCC=C1c2ccccc2OCc2c1oc1c2cccc1)C
O=C1N(CC2c3c1cccc3CCC2)C1CN2CCC1CC2
Nc1nc2c(c[nH]c2c(=O)[nH]1)Cc1cccnc1
OC(c1ccc(cc1)Cl)(c1ccc(cc1)Cl)C1CC1
CC(NCC(c1ccc(c(c1)NS(=O)(=O)C)O)O)C
ClCCN(CCN1C(=O)NC2(C1=O)CCCCC2)CCCl
Clc1ccc2c(c1)C(=NCC(=S)N2C)c1ccccc1
OC1CCCc2c1c(NCc1ccccc1)c1c(n2)cccc1
Brc1ccc(cc1)NC(=O)c1cc(Br)cc(c1S)Br
C#CC1(O)CCC2C1(C)CCC1C2CCC2=C1CCCC2
Brc1ccc(cc1)NC(=O)c1cc(Br)cc(c1O)Br
O=C(Nc1ccc(c(c1)Cl)Cl)Nc1ccc(cc1)Cl
CN1C2CCC1CC(C2)OC(=O)c1cc(C)cc(c1)C
OC(COc1ccc(cc1)O)CNCCNC(=O)N1CCOCC1
COc1cc(N)c(cc1C(=O)NC1CN2CCC1CC2)Cl
O=C1CN=C(c2c(N1C)n(C)nc2C)c1ccccc1F
C=Cc1cc(O)cc2c1oc(n2)c1ccc(c(c1)F)O
COc1cc(CCOC2CCCCC2N2CCC(C2)O)ccc1OC
CCCCCCCCCCCC[N+](Cc1ccccc1)(CCO)CCO
CS(=O)(=O)c1ccc(cc1)c1nc2n(c1)cccc2
CCc1cc2cc(OC)c(cc2c(n1)Cc1ccccc1)OC
CN(CCOCC(=O)NC12CC3CC(C2)CC(C1)C3)C
O=C1C(=C(N2CC2)C(=O)C=C1N1CC1)N1CC1
CCNC1=Nc2ccc(cc2C(O1)(C)c1ccccc1)Cl
CCC(=O)C(c1ccccc1)(c1ccccc1)CCN(C)C
CCCCN(C(=O)c1cccc2c1cccc2)CCN(CC)CC
Oc1cc(C)c(cc1C1CC2C(C1(C)CC2)(C)C)C
[O-]C(=O)COc1nn(c2c1cccc2)Cc1ccccc1
OC(=O)Cc1cccc(c1N)C(=O)c1ccc(cc1)Br
OC(COc1cccc2c1c1ccccc1[nH]2)CNC(C)C
CCOP(=O)(OC(=CCl)c1ccc(cc1Cl)Cl)OCC
CCCCCCCCCCCCCCCC[N+](C1CCCCC1O)(C)C
CN1CCCN(CC1)C(c1ccc(cc1)Cl)c1ccccc1
CCCCCN(CCC(P(=O)(O)O)(P(=O)(O)O)O)C
CC(C(C1CNc2c(N1)c(=O)nc([nH]2)N)O)O
O=C(C(N)C)NC1(CCC2C1C2C(=O)O)C(=O)O
FCCOCCOCCOc1ccc(cn1)C=Cc1ccc(cc1)NC
OC(=O)C(c1ccccc1)(c1ccccc1)c1ccccc1
[O-]C(=O)C1=Cc2c(CC1)cc(s2)Cn1cncc1
CC(N1CCCCC1)CC(c1ccccc1)(c1ccccc1)O
FCCOCCOCCOc1ccc(cc1)C=Cc1ccc(cc1)NC
NC(=O)C(NCc1ccc(cc1)OCc1cccc(c1)F)C
COc1ccc2c(c1)CN(CCS2)C(=O)C(=O)[O-]
CCOC(=O)NC(=N)NC(=O)Cc1c(Cl)cccc1Cl
CC(=O)c1cc2c(o1)C(=O)c1c(C2=O)cccc1
SCC(C(=O)NCC(=O)O)NC(=O)CCC(C(=O)O)N
OC(=O)CN(CC(=O)O)CCN(CC(=O)O)CC(=O)O
CC1C(=O)OC2C1CCC1(C2=C(C)C(=O)C=C1)C
OC1CCC2(C(=CCC3C2CCC2(C3CCC2O)C)C1)C
CC(C1C2CC(=C(N2C1=O)C(=O)O)SCCNC=N)O
CCCC(C(=O)OC1CC2CCC(C1)[N+]2(C)C)CCC
CCC(=C)C(=O)c1ccc(c(c1Cl)Cl)OCC(=O)O
NC(=Nc1scc(n1)CSCCC(=NS(=O)(=O)N)N)N

Continued on next page

Fc1ccc(c(c1)F)C(Cn1cncn1)(Cn1cncn1)O
O=C(NS(=O)(=O)c1ccc(cc1)C)NN1CCCCCC1
OC(COc1cccc2c1CC(O)C(C2)O)CNC(C)(C)C
Cc1ccnc2c1[nH]c(=O)c1c(n2C2CC2)nccc1
CC(=CCN1CCC2(C(C1Cc1c2cc(cc1)O)C)C)C
Clc1cc(cc(c1Cl)S(=O)(=O)N)S(=O)(=O)N
CCCCOc1nc2ccccc2c(c1)C(=O)NCCN(CC)CC
OC(C1CC2CC1C=C2)(c1ccccc1)CCN1CCCCC1
CCc1c(C)[nH]c2c1C(=O)C(CC2)CN1CCOCC1
O=C1CCC2C34C1Oc1c4c(CC2N(CC3)C)ccc1O
OC(P(=O)(O)O)(P(=O)(O)[O-])Cc1cccnc1
N#Cc1ccc(cc1)C(n1cncn1)c1ccc(cc1)C#N
Clc1c[nH]cc1c1cccc(c1[N+](=O)[O-])Cl
COc1cccc(c1)C(=O)CCNC(C(c1ccccc1)O)C
CCN(CCCN(C1Cc2c(C1)cccc2)c1ccccc1)CC
CCc1sc2c(c1)C(=NCC(=O)N2C)c1ccccc1Cl
CN1CCC(=C2c3ccccc3CC(=O)c3c2ccs3)CC1
Clc1ccc2c(c1)C(=NCC(=O)N2C)c1ccccc1F
COCCOc1cnc(nc1)[N-]S(=O)(=O)c1ccccc1
C=CCC1(O)CCC2C1(C)CCC1C2CCC2=CCCCC12
CCOCC(COc1ccc(cc1)NC(=O)CC[S+](C)C)O
CC(Cc1ccc(cc1)O)NCC(c1cc(O)cc(c1)O)O
O=CN(C(=C1CCOC(=O)S1)C)Cc1cnc(nc1N)C
COc1ccc2c3c1OC1C43CCN(C(C2)C4CCC1O)C
CCCCOC(=O)C(=O)Nc1cccc(c1)c1nnn[nH]1
CCOC(=O)c1ccc(cc1)OC(=O)CCCCCNC(=N)N
Clc1ccc(cc1)Cn1c(CN2CCCC2)nc2c1cccc2
ClCCN(C(=O)NCC1OC(OC)C(C(C1O)O)O)N=O
Cc1nc[nH]c1CC1CCc2n(C1=O)c1ccccc1c2C
O=C(N(c1c(C)cccc1C)CCCc1cccnc1)C(N)C
Nc1ccc(cc1)S(=O)(=O)Nc1ccnn1c1ccccc1
OC(=O)Cc1sc(nc1c1ccc(cc1)Cl)c1ccccc1
COc1ncnc(c1)[N-]S(=O)(=O)c1ccc(cc1)N
N#Cc1cc(c([nH]c1=O)C)c1ccc2n(c1)ccn2
OCc1c(CSSCc2cnc(c(c2CO)O)C)cnc(c1O)C
COc1ccc2c3c1OC1C3(CCN(C2)C)C=CC(C1)O
[O-]C(=O)C1N2C(=O)CC2S(=O)(=O)C1(C)C
NCC1CCC(CC1)C(=O)Oc1ccc(cc1)CCC(=O)O
Fc1ccc(cc1)C1CCNCC1COc1ccc2c(c1)OCO2
COC(=O)C(NC(=O)C(CC(=O)O)N)Cc1ccccc1
OC(=O)COc1ccc(c(c1Cl)Cl)C(=O)c1cccs1
[O-]P1(=O)OP(=O)([O-])OP(=O)(O1)[O-]
COc1cc(nc(n1)C)NS(=O)(=O)c1ccc(cc1)N
OCc1cccc(n1)C=Cc1ccc(o1)[N+](=O)[O-]
OC(C(C)(C)C)(Cn1ncnc1)COc1ccc(cc1)Cl
CN(CCCN1c2ccccc2Sc2c1cc(cc2)C(=O)C)C
COc1ccc2c(c1)N(CCCN(C)C)c1c(S2)cccc1
CN1CCC(=C2c3ccccc3Oc3c2cc(Cl)cc3)CC1
CN1CCN(CC1)C1=Nc2ccccc2Sc2c1cc(C)cc2
CN(CCN1C(=O)CC(Sc2c1cccc2)c1ccccc1)C
O=C1N(CCc2c1c1ccccc1n2C)Cc1nc[nH]c1C
CCCc1cc2c(cc1N(CC)CC)ncc(c2O)C(=O)OC
Nc1nccc(n1)C=Cc1ncc(n1C)[N+](=O)[O-]
CN1CCC2(C(C1)c1cccc(c1Oc1c2cccc1)C)O
O=C1NN=C(C(C1)C)c1ccc2c(c1)OCC(=O)N2
Brc1ccc(cc1)CNC(=O)c1cc(Br)cc(c1O)Br
CON=CC1C2CCC(N2C)CC1c1ccc(c(c1)Cl)Cl
Brc1cccc(c1)c1nnn(n1)CCC(=O)N1CCCCC1
O=C(C(C)C)Nc1cccc(c1)C1N=C2N(C1)CCS2
O=C1Cc2c(N1)ccc1c2OC(CNCc2ccccc2)CO1
CCCCC(C1NC(=O)N(C1=O)SC(Cl)(Cl)Cl)CC
OC(=O)C(c1ccc(c(c1)Cl)C(=O)c1cccs1)C
Oc1ccc2c(c1)C1(C)CCN(C(C2)C1C)CC1CC1
CCN1CC(CN1CC)NC(=O)c1cc(Cl)c(cc1OC)N
Clc1ccc2c(c1)CCc1c(C2=C2CCNCC2)nccc1
OC(COc1ccc(cc1C(=O)C)NC(=O)C)CNC(C)C
NCc1c[nH]c(=S)n1C1CCc2c(C1)cc(cc2F)F
Cc1noc(c1)CCCCCCCOc1ccc(cc1)C1=NCCO1
COc1ccc2c(c1)CCC1C2CCC2(C1CC(C2O)O)C
CCN1C2CCC1CC(C2)OC(c1ccccc1)c1ccccc1
CCOC(=O)N(c1cccc(c1)C(F)(F)F)CCN(C)C
OC(=O)c1cccc2c1Nc1cc(ccc1S2)C(F)(F)F
OCC1NC(C(C1O)O)c1c[nH]c2c1nc[nH]c2=O
O=C1C(CCc2c1c1ccccc1n2C)Cc1nc[nH]c1C
OC(=O)c1cn2C(C)CCc3c2c(c1=O)cc(c3C)F
CC(NCCCC1(C(=O)N)c2ccccc2c2c1cccc2)C
CCC1=C2CCC3(C(C2CCC1=O)CCC3OC(=O)C)C
COc1cc(ccc1c1nc2c([nH]1)ccnc2)S(=O)C
N#CC(=NNc1ccc(cc1)C1=NNC(=O)CC1C)C#N
O=C(Nc1c(C)cccc1C)CN(CC(=O)O)CC(=O)O
CN1CC2C3CC(=O)NC2(CC1)Oc1c3cc(Cl)cc1

Continued on next page

CCN(CCNC(=O)c1ccc(cc1)NS(=O)(=O)C)CC
CCC#CC(C1(CC=C)C(=O)NC(=O)N(C1=O)C)C
NC(=O)C(=O)NN=Cc1ccc(o1)[N+](=O)[O-]
[O-][N+](=Cc1ccc(o1)[N+](=O)[O-])CCO
[O-][N+](=O)c1ccc(o1)C=NN1CC(NC1=O)C
CCCCC(COP(=O)(Oc1ccccc1)Oc1ccccc1)CC
NCCNc1ccc(c2c1C(=O)c1cnccc1C2=O)NCCN
OC(C[N+](C(C)C)(C)C)COc1cccc2c1cccc2
O=C1CCC(=NN1)c1ccc2c(c1)CN(C(=O)N2)C
Cc1ccc2c(c1)n(C(C)C)c(=O)nc2c1ccccc1
CCCc1c2CCCCc2c(c2c1oc(cc2=O)C(=O)O)O
CCCCCCCCCCCCCCCCCOC(=O)C=CC(=O)[O-]
OC1CCCCCc2cc(O)cc(c2C(=O)OC(CCC1)C)O
OCC1OC(C(C1O)O)n1cnc2c1ncnc2NC1COCC1
Fc1ccc(cc1)Cn1c(NC2CCNCC2)nc2c1cccc2
CC([N+](C)(C)C)CN1c2ccccc2Sc2c1cccc2
CSc1ccc2c(c1)c(=O)n(cn2)C=CC(=O)[O-]
CC1CCCC(N1C(=S)Nc1cccc(c1)C(F)(F)F)C
CSCc1cc(=O)c2c(o1)c(OC)c1c(c2OC)cco1
OC(COc1ccccc1C)CNCCOc1ccc(cc1)C(=O)N
Cc1cccc(c1)N(C(=S)Oc1ccc2c(c1)CCC2)C
Clc1ccc(c(c1NS(=O)(=O)C)C)OCC1=NCCN1
COc1cc(N)c(cc1C(=O)NC1CCN2CC1CCC2)Cl
OCC=C(C=CC=C(C=CC1=C(C)CCCC1(C)C)C)C
COc1ccc(cc1OCC1CC1)C(Cn1cc[nH]c1=O)C
OC(=O)C1NCC2C(C1)CC(CC2)CCc1nnn[nH]1
CCCCC(CN1CN(CC(CCCC)CC)CC(C1)(C)N)CC
C=CCN1CCCC1CNC(=O)c1cc2nn[nH]c2cc1OC
CCCS(=O)c1ccc2c(c1)nc([nH]2)NC(=O)OC
OCCN(c1nc(c(o1)c1ccccc1)c1ccccc1)CCO
CCCOc1ccc2c(c1)CCC1C2CCC2(C1CCC2OC)C
CC[N+](C(CC(c1ccccc1)c1ccccc1)C)(C)C
OC(=O)Cc1cn(nc1c1ccc(cc1)Cl)c1ccccc1
Clc1ccc2c(c1)C(=NCC(=O)N2C)C1=CCCCC1
NC(=O)NN=C1C=C2C(O)CN(C2=CC1=O)C(C)C
c1cnc(nc1)N1CCN(CC1)Cc1ccc2c(c1)OCO2
CC(N1CCC(CC1)(c1ccccc1)c1ccccc1)(C)C
CCOC1OC2OC3(C)CCC4C2(C(C1C)CCC4C)OO3
CN(CCOCCOC(=O)N1c2ccccc2Sc2c1cccc2)C
CN(CCn1nc(Cc2ccccc2)c2c(c1=O)cccc2)C
O=C1CCC2(C(C1)CCC1C2CCC2(C1CCC2O)C)C
CCCCCCCCCCCCCCC([N+](C)(C)C)C(=O)OCC
CCCCCCCCCCCCCCCCCC(=O)OCC[N+](C)(C)C
O=C1CN=C(c2c(N1)ccc(c2)Cl)c1ccccc1Cl
O=C(NC(=O)c1c(F)cccc1F)Nc1ccc(cc1)Cl
OCC1OC(CC1O)n1cc(C(C)C)c(=O)[nH]c1=O
CNC(=O)Oc1ccc2c(c1)C1(C)CCN(OC1N2C)C
CCC1CN2CCC1CC2C(c1ccnc2c1cc(OC)cc2)O
[O-]C(=O)C(c1cccc(c1)C(=O)c1ccccc1)C
COc1ccccc1OC(=O)C(c1ccc(cc1)CC(C)C)C
OC1C=CC2C34C1Oc1c4c(CC2N(CC3)C)ccc1O
O=C1CCC2=C3C=CC4(C(C3CCC2=C1)CCC4O)C
COc1ccc(cc1OC1CC2CC1CC2)C1CNC(=O)NC1
CC(Cc1ccc(cc1)C(C(=O)NS(=O)(=O)C)C)C
CCc1nn(c2c1CCn1c2nnc1c1cccs1)C1CCCC1
NCC(=O)N1CC(CC1C(=O)O)NC(=O)c1ccccc1
CC(=O)OC1Cc2ccccc2N(c2c1cccc2)C(=O)N
COc1ccc(cc1CC(C1CNCCO1)(C1CCOCC1)O)F
O=c1oc2cc(ccc2c2c1CCCCC2)OS(=O)(=O)N
CN(CCON=C(c1ccccc1F)C=Cc1ccc(cc1)O)C
N#CC(=C(O)C)C(=O)Nc1ccc(cc1)C(F)(F)F
NC(=O)C1CCC2CN1C(=O)N2OS(=O)(=O)[O-]
CC1CNCCCN1S(=O)(=O)c1cccc2c1c(F)cnc2
O=c1[nH]c(CN2CCCC2=N)c(c(=O)[nH]1)Cl
Cc1ccccc1C(c1ccccc1C)(C1CN2CCC1CC2)O
CCOP(=O)(Oc1ccc(cc1)[N+](=O)[O-])OCC
ONC(=O)CCCCCCC(=O)Oc1ccc(cc1)C(=O)OC
O=C1CCC2(C(=C1)CCC1C2CCC2(C1CCC2O)C)C
COc1ccc2c3c1OC1C43CCN(C(C2)C4C=CC1O)C
Oc1cc2OC(c3ccc(c(c3)O)O)C(Cc2c(c1)O)O
CN1CCN(CC1)C1=Nc2cc(Cl)ccc2Nc2c1cccc2
CC(=O)OCC(CCn1cnc2c1nc(N)nc2)COC(=O)C
Cc1cccc(c1)N(C(=S)Oc1ccc2c(c1)cccc2)C
CN(CCc1c[nH]c2c1cc(CC1COC(=O)N1)cc2)C
OCc1cc2CCC(Nc2cc1[N+](=O)[O-])CNC(C)C
CCCCC1C(=O)N(N(C1=O)c1ccccc1)c1ccccc1
CCN(c1cccc(c1)c1ccnc2n1ncc2C#N)C(=O)C
C#CC1(O)CCC2C1(C)CCC1C2CCc2c1ccc(c2)O
C=CC1CN2CCC1CC2C(c1ccnc2c1cc(OC)cc2)O
Clc1ccc(cc1)C(c1ccccc1)(OCCC1CCCN1C)C
O=C1CCC(C(=O)N1)N1C(=O)c2c(C1=O)cccc2

Continued on next page

Table S8 – *Continued from previous page*

CN1CCN(CC1)C1=Nc2ccccc2Oc2c1cc(Cl)cc2
Clc1cc(Sc2cc(Cl)cc(c2O)Cl)c(c(c1)Cl)O
COc1ccc2c3c1OC1C43CCN(C(C2)C4CCC1=O)C
COc1nc(OC)nc(c1)NS(=O)(=O)c1ccc(cc1)N
CCN1CCCC(C1)OC(=O)C(c1ccccc1)c1ccccc1
C[N+]1(CCOCC1)CCC(c1cccs1)(c1ccccc1)O
CN(CCC1=C(C(c2ccccn2)C)c2c(C1)cccc2)C
CCn1cc(C(=O)O)c(=O)c2c1nc(nc2)N1CCCC1
O=C(COC(=O)C)NCCCOc1cccc(c1)CN1CCCCC1
OC(COc1ccc(c2c1oc(=O)cc2)C)CNC(C)(C)C
CCc1cc2c(s1)n1c(CN=C2c2ccccc2Cl)nnc1C
CCN(CCNC(=O)c1cc(ccc1OC)S(=O)(=O)C)CC
OC(=O)COC(=O)Cc1ccccc1Nc1c(Cl)cccc1Cl
CN1CCN(CC1)C1=Nc2ccccc2Sc2c1cc(Cl)cc2
CC(=O)Nc1ccc(cc1)S(=O)(=O)Nc1noc(c1)C
CCCn1cnc2c1c(=O)n(CCCCC(=O)C)c(=O)n2C
Brc1cc2c(s1)n1c(CN=C2c2ccccc2Cl)nnc1C
CC1CCCC(N1CCCC(c1ccccn1)(c1ccccc1)O)C
OC(COc1cn(C)c(=O)c2c1cccc2)CNC(C)(C)C
CCCCOC(=O)c1ccccc1Nc1cccc(c1)C(F)(F)F
N#CC(=C1SCC(S1)c1ccc(cc1Cl)Cl)n1cncc1
COc1cc(C=CC(=O)Nc2ccccc2C(=O)O)ccc1OC
CN1C2CCC1CC(C2)OC(=O)c1c[nH]c2c1cccc2
OC(COc1cc(C)c(c(c1C)C)OC(=O)C)CNC(C)C
COc1ccc(cc1OC)Cc1nccc2c1cc(OC)c(c2)OC
Fc1cc2CCC(n3c2c(c1)c(=O)c(c3)C(=O)O)C
Fc1ccc(cc1)C(=O)CCCN1CCN(CC1)c1ccccn1
Fc1ccc2c(c1)c1CN(CCc3ccncc3)CCc1[nH]2
[O-]C(=O)Cn1c(=O)c2cccc3c2c(c1=O)ccc3
CCCc1c(OCCCCc2nnn[nH]2)ccc(c1O)C(=O)C
NC(=O)Nc1ccc(cc1)S(=O)(=O)c1ccc(cc1)N
O=C1NCCN1C1CCN(CC1)CCC1COc2c(O1)cccc2
CN1C2CCC1CC(C2)OC(=O)c1cc(Cl)cc(c1)Cl
O=C1C(CCc2c1c1cccc3c1n2CCC3)Cn1ccnc1C
CCCCCC(CCC1CCC(=O)C1CCCCCCC(=O)O)(O)C
C1CCC(NC1)C1COC(O1)(c1ccccc1)c1ccccc1
O=C1NC(=O)CN(C1)CC(N1CC(=O)NC(=O)C1)C
OC(C(Cl)(Cl)Cl)NC(=O)NC(C(Cl)(Cl)Cl)O
COC(=O)Nc1[nH]c2c(n1)cc(cc2)SC1CCCCC1
CN1CCc2c(C3C1CCc1c3cccc1)cc(c(c2)Cl)O
COc1ccc(cc1)C(=O)Nc1ccccc1CCC1CCCCN1C
NN=c1[nH]nc2c(c1)CN(CC2)C(=O)c1ccccc1
OCC1C(O)CC(C1=C)n1cnc2c1nc(N)[nH]c2=O
Cc1ccc(cc1)C=C1C(=O)C2(C(C1CC2)(C)C)C
COC(=O)Nc1[nH]c2c(n1)cc(cc2)Sc1ccccc1
OC(CNC(CNC(=O)N)(C)C)COC(=O)c1ccccc1F
Cc1cc(O)c2c(n1)ccc1c2[nH]c(n1)c1ccco1
O=C(NS(=O)(=O)c1ccc(cc1)C)NC1CCCCCCC1
CCC(c1ccccc1)(N(CC1CC1)C)CC=Cc1ccccc1
OC(=O)C(c1ccc(cc1)N1Cc2c(C1=O)cccc2)C
CCCCCCCCCCCC(=O)OCCNC(=O)C[n+]1ccccc1
COc1cc2c(cc1OC)CCN(C2CCc1ccc(cc1)Cl)C
[O-][N+](=O)c1cccc(c1)C1N=C2N(C1)CCS2
ON=Cc1cc[n+](cc1)COC[n+]1ccc(cc1)C=NO
CCOC(=O)c1c(C)cc2c(c1C)c(=O)[nH]nc2CO
NC(=N)c1ccc(cc1)C=C1c2ccccc2c2c1cccc2
Clc1cc2NC(=NS(=O)(=O)c2cc1Cl)C1CC=CC1
FC(C(C(F)(F)F)(F)F)(C(C(F)(F)F)(F)F)F
FC(C(C(C(F)(F)F)(F)F)(C(F)(F)F)F)(F)F
CCN1CCCC1CNC(=O)c1c(O)c(Cl)cc(c1OC)Cl
OCC1OC(C(C1O)O)n1cnc2c1ncnc2NCC=C(C)C
CCCCC(CC=CC1C(O)CC(=O)C1CCCCCCCO)(O)C
N=C(NN=Cc1ccc(cc1)Cl)NN=Cc1ccc(cc1)Cl
CCCCCCCCCCCCCCCC(=O)OCC(C1OCC(C1O)O)O
ClC=C(c1cc(Cl)c(cc1Cl)Cl)OP(=O)(OC)OC
Nc1ccc(cc1)S(=O)(=O)Nc1cnc2c(n1)cccc2
CC(Cc1sc2c(c1C)c(=O)nc([nH]2)C(=O)O)C
NC(=O)C(c1ccccc1)(c1ccccc1)CCn1ccnc1C
CCC(Cn1c(=O)n(C)c(=O)c2c1nc([nH]2)C)C
COC(=O)c1cc(Cl)ccc1NS(=O)(=O)C(F)(F)F
O=Cc1cnc2n1CCc1c(C2=C2CCN(CC2)C)cccc1
C1CCC2(C1)C1CN(CC2CN(C1)CC1CC1)CC1CC1
C[N+]1(C)CCC(=C(c2ccccc2)c2ccccc2)CC1
CCN(CC(OC(=O)C1CCCCC1(O)C1CCCCC1)C)CC
[O-]P(=O)(C(P(=O)(O)[O-])NC1CCCCCC1)O
NC(=N)c1ccc(cc1)OCCCOc1ccc(cc1)C(=N)N
CCN(CCCOC(=O)C1(CC2CC1CC2)c1ccccc1)CC
CN1C2CCC1CC(=C1c3ccccc3SCc3c1cccc3)C2
c1ccc2c(c1)N(C1CN3CCC1CC3)c1ccccc1CC2
CN1C2CCC1CC(C2)OC1c2ccccc2CCc2c1cccc2
COc1ccc2c(c1CN1CCCCC1)oc1c(c2=O)cccc1

Continued on next page

CCN(CCOC(=O)C(c1ccccc1)(c1ccccc1)O)CC
COc1cc(OC)cc(c1C(=O)CCC[NH+]1CCCC1)OC
COC(CCCC(CC=CC(=CC(=O)OC(C)C)C)C)(C)C
OC1CCC2(C(=CCC3C2CCC2(C3CCC2=O)C)C1)C
C=CC1CN2CCC1CC2C(c1ccnc2c1cc(OC)cc2)O
Nc1ccc(c(c1)N)N=Nc1ccc(cc1)S(=O)(=O)N
CCCCCCCCCCCCCCCCOC(=O)CCCCCCCCCCCCCCC
ClCCN(S(=O)(=O)C)N(S(=O)(=O)C)C(=O)NC
OCCn1nc(c(c1CC)Oc1cc(C#N)cc(c1)C#N)CC
CC(N(C)C)CC(c1ccccc1)(c1ccccc1)C(=O)N
CN(CCn1c(=O)c2cc(N)cc3c2c(c1=O)ccc3)C
CC1CCCN1CCCOc1ccc(cc1)c1ccc(=O)[nH]n1
Cc1cn(C2OC(C=C2)(CO)C#C)c(=O)[nH]c1=O
O=C(c1cc2c(s1)c(Cl)ccc2)NC1CN2CCC1CC2
Oc1ccc2c(c1)ccc(n2)c1ccc(cc1Cl)C(=O)O
O=C1CCC2(C(=C1)CCC1C2CCC2(C1CCC2=O)C)C
CCCCCC(C=CC1C(O)CC(C1CC=CCCCC(=O)O)O)O
OC(C(=O)NC(C(=O)O)CC(C)C)C(Cc1ccccc1)N
COC(=O)C1C(CC2N(C1CC2)C)OC(=O)c1ccccc1
CCCCCC(C=CC1C(O)CC(=O)C1CCCCCCC(=O)O)O
Clc1ccc2c(c1)C(=NCc1n2c(C)nn1)c1ccccc1
Clc1ccc2c(c1)C(=Nc1c(O2)cccc1)N1CCNCC1
Clc1ccccc1C(n1cncc1)(c1ccccc1)c1ccccc1
CCC(=O)N(c1ccccc1)C1CCN(CC1)CCc1ccccc1
Clc1ccc2c(c1)C(=NC(C(=O)N2C)O)c1ccccc1
CN(CCCN1c2ccccc2Sc2c1cc(cc2)C(F)(F)F)C
CCC1NC(=O)c2c(N1)cc(c(c2)S(=O)(=O)N)Cl
CN1CCN(CC1)CCCN1c2ccccc2Sc2c1cc(Cl)cc2
[O-][N+](=O)OC1COC2C1OCC2O[N+](=O)[O-]
C#CC1(O)CCC2C1(C)CCC1C2CCc2c1ccc(c2)OC
CN1C2CCCC1CC(C2)NC(=O)c1nn(c2c1cccc2)C
OCc1cc(ccc1O)C(CNCCCCCCOCCCCc1ccccc1)O
CCOC(=O)c1ncn2c1CN(C)C(=O)c1c2ccc(c1)F
CC[N+](CCC(c1ccccc1)(C1CCCCC1)O)(CC)CC
Clc1ccc(c(c1)Cl)C(Cn1ccnc1)OCc1ccsc1Cl
[O-][N+](=O)c1ccc(o1)C=NN1CC(OC1=O)CSC
CN(Cc1cccc2c1cccc2)Cc1ccc(cc1)C(C)(C)C
CCOC(=O)COc1ccc2c(c1)oc(cc2=O)c1ccccc1
CC12Cc3nonc3CC1CCC1C2CCC2(C1CCC2(C)O)C
N#Cc1cc(ccc1OCC(C)C)c1nc(c(s1)C(=O)O)C
CCN1CCCC1CNC(=O)c1cc(ccc1OC)S(=O)(=O)N
Clc1ccc2c(c1)N(C(=O)CC(=O)N2C)c1ccccc1
CCC1CC2C(C1O)(C)CCC1C2CCC2=CC(=O)CCC12
O=C(C(c1ccccc1)(c1ccccc1)O)OCCN1CCCCC1
CCC(C(c1ccccc1)C(=O)OCC[N+](CC)(CC)C)C
COc1cccc(n1)N(C(=S)Oc1ccc2c(c1)CCCC2)C
Oc1ccc(cc1c1ccccc1)C(=O)c1ccccc1C(=O)O
CCN(CCOC(=O)CC(c1ccccc1)(c1ccccc1)O)CC
CC(=Cc1ccccc1)C=C1SC(=S)N(C1=O)CC(=O)O
c1ccc(cc1)c1ccc(cc1)C(n1ccnc1)c1ccccc1
C[N+]12CCCCC2CCC(=C(c2cccs2)c2cccs2)C1
CCC(=O)C(c1ccccc1)(c1ccccc1)CC(N(C)C)C
OC(c1ccccc1)CC1CCCC(N1C)CC(=O)c1ccccc1
CCn1cc(C(=O)O)c(=O)c2c1cc(cc2)c1ccncc1
Nc1ccc(cc1)S(=O)(=O)Nc1nnc(s1)C(C)(C)C
CCn1cc(C(=O)O)c(=O)c2c1ccc1c2sc(=O)n1C
CN1CCN(CC1)C1=Nc2cc(F)ccc2Nc2c1cc(s2)C
Fc1ccc2c(c1)c1CN(CCCc3cccnc3)CCc1[nH]2
Cc1onc(c1c1ccc(cc1)S(=O)(=O)N)c1ccccc1
OC1C(O)C(OC1n1cnc2c1ncnc2N)COP(=O)(O)O
CCN(Cc1cc(ccc1O)Nc1ccnc2c1ccc(c2)Cl)CC
CCCC1C(=O)n2n(C1=O)c1cc(C)ccc1nc2N(C)C
CCc1cccc(c1)N(C(=N)Nc1ccc2c(c1)cccc2)C
O=CNc1cc(ccc1O)C(CNC(Cc1ccc(cc1)OC)C)O
CCC(=O)C(C(=O)CC)CCCCCCOc1ccc(cc1Cl)OC
Fc1ccc(cc1)Cc1ccc(s1)C#CC(N(C(=O)N)O)C
OCC(c1ccccc1)C(=O)OC1CC2CCC(C1)N2(=O)C
CCCN(CCOC(=O)C(c1ccccc1)(c1ccccc1)O)CC
FC(c1ccc(cc1)c1ccc(cc1)OCCN1CCCC1)(F)F
CCCCc1ccc(cc1)NC(=O)CN(CC(=O)O)CC(=O)O
COc1cc(cc(c1OC)OC)C(=O)NCCCCCC(=O)[O-]
OC(=O)C(c1ccc2c(c1)[nH]c1c2cc(cc1)Cl)C
OCC(C(c1ccc(cc1)C(=O)C)O)NC(=O)C(Cl)Cl
Clc1cc2N=C[N-]S(=O)(=O)c2cc1S(=O)(=O)N
Clc1ccc2c(c1)C1N(CCc3c1cccc3)CC(=O)N2C
OCC(NCc1cc2c3ccccc3ccc2c2c1cccc2)(CO)C
COC(=O)Nc1[nH]c2c(n1)cc(cc2)C(=O)C1CC1
CCCCCC(C=CC1CCC(=O)C1CCCCCCC(=O)O)(O)C
CCC(=O)N(C1CCCC1N(C)C)c1ccc(c(c1)Cl)Cl
CC(=O)Oc1ccc2c(c1)CCC1C2CCC2(C1CCC2O)C
C#CC1(O)CCC2C1(C)CCC1N2CCc2c1ccc(c2)OC

Continued on next page

CCc1cccc(c1NC(=O)CN(CC(=O)O)CC(=O)O)CC
O=C(C(c1ccccc1)O)OC1CC(C)N(C(C1)(C)C)C
CCOC(=O)C(c1ccccc1)(C(=O)NCCN(CC)CC)CC
CC(c1cccc(c1OCOP(=O)([O-])[O-])C(C)C)C
CCCCCN(CCC(P(=O)(O)O)(P(=O)(O)[O-])O)C
CNCCc1ccc(c(c1)OC(=O)C(C)C)OC(=O)C(C)C
O=C1CCC(=NN1)c1ccc2c(c1)C(C)(C)C(=O)N2
CN(CCOc1cc(C)c(cc1C(C)C)OC(=O)OC(C)C)C
OCC1=C(C)C=C2C1=C(C)C1(C(C2=O)(C)O)CC1
Oc1ccc2c(c1)C1(C)CCN(C(C2=O)C1C)CC1CC1
CC(C(Cc1ccc(c(c1)O)O)C)Cc1ccc(c(c1)O)O
COP(=O)(C(c1ccc(cc1)Cl)OP(=O)(OC)OC)OC
COc1cc(ccc1O)C(=O)Nc1ccccc1CCC1CCCCN1C
OC(=O)C(Oc1ccc(cc1)C1CCCc2c1cccc2)(C)C
[O-][N+](=O)c1ccc(o1)C=NN1CCN(C1=O)CCO
S=C=Nc1ccc(cc1)Oc1ccc(cc1)[N+](=O)[O-]
COc1ccc(cc1Cc1cnc2c(c1C)c(N)nc(n2)N)OC
NC(=O)CCCN=C(c1cc(F)ccc1O)c1ccc(cc1)Cl
CCCOC(=O)Cc1ccc(c(c1)OC)OCC(=O)N(CC)CC
O=C(CP(=O)([O-])[O-])NC(C(=O)O)CC(=O)O
CC[N+](CCOc1ccc(cc1)C=Cc1ccccc1)(CC)CC
OC(c1ccc(c(c1)CS(=O)(=O)C)O)CNC(C)(C)C
COc1cc(O)c(cc1S(=O)(=O)O)C(=O)c1ccccc1
OC(=O)COc1ccc(cc1)CCNS(=O)(=O)c1ccccc1
CC(=O)Oc1ccccc1C(=O)OC1OC(=O)c2c1cccc2
Clc1ccc(cc1)Nc1nnc(c2c1cccc2)Cc1ccncc1
OCCN(CC(Cn1cnc2c1c(=O)n(C)c(=O)n2C)O)C
CC(=CC=CC(=CC(=O)O)C)CCC=C(CCC=C(C)C)C
C1CNC2CCCCC2NCc2nc(CNC3C(N1)CCCC3)ccc2
ClCCN(c1ccc2c(c1)nc(n2C)CCCC(=O)O)CCCl
O=C(NS(=O)(=O)c1ccc(c(c1)N)C)NC1CCCCC1
CCC1(O)CCC2C1(C)CCC1C2CCC2=CC(=O)CCC12
CCC(c1ccc(cc1)N1Cc2c(C1=O)cccc2)C(=O)O
OC(=O)C(c1ccc2c(c1)nc(o2)c1ccc(cc1)F)C
BrC=Cc1cn(C2CC(C(O2)CO)O)c(=O)[nH]c1=O
Clc1ccc(c(c1)Cl)Cn1nc(c2c1cccc2)C(=O)O
CCCCC(C(=O)N(c1ccccc1)Nc1ccccc1)C(=O)O
CC(=O)Nc1ccc(cc1)OC(=O)c1ccccc1OC(=O)C
CCC(N(C(CC)C)CC(c1cccn1Cc1ccccc1Cl)O)C
C#CCN1C(=O)CN=C(c2c1ccc(c2)Cl)c1ccccc1
[O-][N+](CCCN1c2ccccc2CCc2c1cccc2)(C)C
OCc1c(COC(=O)CCC(=O)OCCN(C)C)cnc(c1O)C
ClC(C(c1ccc(cc1)Cl)c1ccc(cc1)Cl)(Cl)Cl
COC(c1ccccc1)CN1CCN(CC1)CCC(c1ccccc1)O
CC(OC(=O)C1(CCOCC1)c1ccccc1)CCN1CCOCC1
O=C1C=CC2(C(=C1)CCC1C2CCC2(C1CCC2O)C)C
Clc1cc2CCc3c(C(c2c(c1)Cl)n1cncc1)cccc3
CCOc1ccc2c3c1OC1C43CCN(C(C2)C4C=CC1O)C
C#CC1(O)CCC2C1(C)CCC1C2CCC2=CC(CCC12)O
[O-]C(=O)C(CC(=O)[O-])(CC(=O)[O-])[O-]
NCCCNCCCCNC(=O)C(NC(=O)CCCCCCNC(=N)N)O
Oc1ccc(cc1)C(C(N1CCC(CC1)Cc1ccccc1)C)O
O=C1CC(C)(C(=O)c2c1cccc2)S(=O)(=O)[O-]
O=C(c1ccccc1)OCCn1c(C)ncc1[N+](=O)[O-]
OCCN1CCN(CC1)CCCN1c2ccccc2C=Cc2c1cccc2
CCn1cc(C(=O)O)c(=O)c2c1nc(nc2)N1CCNCC1
OCCOCCN1CCN(CC1)C1=Nc2ccccc2Sc2c1cccc2
Nc1ccc(cc1)S(=O)(=O)[N-]c1nc(C)cc(n1)C
COc1cc2c(cc1OC)CCN1C2CC(=O)C(C1)CC(C)C
CCCc1c(ncn2c1nc(n2)C)Cn1ccnc1c1ncccc1F
ONC(=O)C=Cc1cccc(c1)S(=O)(=O)Nc1ccccc1
OP(=O)(C(P(=O)(O)O)(Cc1cnc2n1cccc2)O)O
CCCCCCCCCCCCCCCCOc1nc2ccc(cc2c(=O)o1)C
CN1CCc2c(C1)c1cc(C)ccc1n2CCc1ccc(nc1)C
OS(=O)(=O)c1ccc2c(c1)nc([nH]2)c1ccccc1
CCCCCCCCNC(=N)NC(=N)NCc1ccc(c(c1)Cl)Cl
CCCCCCCOc1ccc(cc1C(F)(F)F)CCC(CO)(CO)N
OCC1OC(C(C1O)O)(CO)OC1OC(CO)C(C(C1O)O)O
OCC1OC(O)C(C(C1OC1OC(CO)C(C(C1O)O)O)O)O
CCCCCC(C=CC1C(O)CC(=O)C1CC=CCCCC(=O)O)O
OCC(c1ccccc1)C(=O)OC1CC2N(C(C1)C1C2O1)C
C#CC1(O)CCC2C1(C)CCC1C2CCC2=CC(=O)CCC12
OC1CCC2(C(C1)CCC1C2CCC2(C1CCC2C(O)C)C)C
Cn1cnc(c1Sc1ncnc2c1[nH]cn2)[N+](=O)[O-]
OC(=O)c1cc(c(cc1NCc1ccco1)Cl)S(=O)(=O)N
OCC1OC(C(C1OC1OC(CO)C(C(C1O)O)O)O)(O)CO
Clc1ccc2c(c1)C(=NC(C(=O)N2)O)c1ccccc1Cl
CSc1ccc2c(c1)N(CCC1CCCCN1C)c1c(S2)cccc1
OCC1OC(CC1O)n1cc(c(=O)[nH]c1=O)C(F)(F)F
COc1ccc2c(c1)N(CC(CN(C)C)C)c1c(S2)cccc1
CNC(=C[N+](=O)[O-])NCCSCc1ccc(o1)CN(C)C

Continued on next page

Table S8 – *Continued from previous page*

O=C1NC(=O)N(C1)N=Cc1ccc(o1)[N+](=O)[O-]
CNC(=C[N+](=O)[O-])NCCSCc1csc(n1)CN(C)C
CNS(=O)(=O)Cc1ccc2c(c1)c(CCN(C)C)c[nH]2
O=C1OCC2(C(C1)CCC1C2CCC2(C1CCC2(C)O)C)C
O=C(N1CC(=O)N2C(C1)c1ccccc1CC2)C1CCCCC1
Clc1ccc2c(c1)C(=NCc1n2c(C)nc1)c1ccccc1F
CC(CCc1ccccc1)NCC(c1ccc(c(c1)C(=O)N)O)O
CC(COCC(N1CCCC1)CN(c1ccccc1)Cc1ccccc1)C
CCNC1CC(C)S(=O)(=O)c2c1cc(s2)S(=O)(=O)N
CCC#CC(C1(CC=C)C(=O)N=C(N(C1=O)C)[O-])C
CN1CCCCC1CCN1c2ccccc2Sc2c1cc(cc2)S(=O)C
N#Cc1ccc2c(c1)COC2(CCCN(C)C)c1ccc(cc1)F
O=C1CCC2(C34C1Oc1c4c(CC2N(CC3)C)ccc1O)O
Clc1ccc(cc1)COC(c1ccc(cc1Cl)Cl)Cn1cncc1
Clc1ccc(cc1)CSC(c1ccc(cc1Cl)Cl)Cn1cncc1
CCOC(=O)C1=CC(C(C(C1)N)NC(=O)C)OC(CC)CC
CCc1ccc(nc1)CCOc1ccc(cc1)CC1SC(=O)NC1=O
CN1CCCN=C1COC(=O)C(c1ccccc1)(C1CCCCC1)O
CCOCn1c(=O)[nH]c(=O)c(c1Cc1ccccc1)C(C)C
OCCOCCN1CCN(CC1)C(c1ccc(cc1)Cl)c1ccccc1
CC(C1CCC(CC1)C(=O)NC(C(=O)O)Cc1ccccc1)C
COc1ccc2c(c1)Sc1c(N2C)cc(cc1)C(C(=O)O)C
CN(CCC=C1c2cc(ccc2OCc2c1cccc2)CC(=O)O)C
COc1ccc2c(c1CN(C)C)oc(c(c2=O)C)c1ccccc1
c1ccc2c(c1)N(CC1CN3CCC1CC3)c1c(S2)cccc1
CC[N+]1(CC)CCC(=C(c2ccccc2)c2ccccc2)C1C
O=C(c1cccnc1)Nc1c(=O)n(n(c1C)C)c1ccccc1
CC[N+](CCOC(=O)C(C1CCCC1)C1CCCC1)(CC)CC
OC(COc1cccc2c1oc(c2)C(=O)C)C[NH2+]C(C)C
O=C1CCC2(C(C1)CCC1C2CCC2(C1CCC2(C)O)C)C
OC(=O)C(c1ccc2c(c1)CC(=O)c1c(S2)cccc1)C
OC(c1cc(Cl)c(c(c1)C(F)(F)F)N)CNC(C)(C)C
CCC(c1ccc(cc1)CC(CN1CC(C)OC(C1)C)C)(C)C
BrC=Cc1cn(c(=O)[nH]c1=O)C1OC(C(C1O)O)CO
O=C1Nc2ccc(cc2C2(N(C1)CCO2)c1ccccc1F)Br
CCCN(C(=O)C(NC(=O)c1ccccc1)CCC(=O)O)CCC
O=C1NC(=O)C2(N1)CC(Oc1c2cc(F)cc1)C(=O)N
COc1cc(CC2NCCc3c2cc(O)c(c3)O)cc(c1OC)OC
O=C(c1cn(c2c1cccc2)C)C1CCc2c(C1)[nH]cn2
OCC(C(C(C(CO)O)O)OC1OC(CO)C(C(C1O)O)O)O
OC(c1ccccc1)C(=O)OC1CC2CCC(C1)[N+]2(C)C
OC(=O)C1CCCN(C1)CCC=C(c1sccc1C)c1sccc1C
CCN1CCCC1CNC(=O)c1cc(ccc1OC)S(=O)(=O)CC
N#Cc1ccc2c(c1)C(N1CCCC1=O)C(C(O2)(C)C)O
OC(COc1ccccc1C)CNCCn1cc(C)c(=O)[nH]c1=O
CCC(C(c1ccc(c2c1ccc(=O)[nH]2)O)O)NC(C)C
CCOc1ccc2c(c1)c(ccn2)C(C1CC2CCN1CC2CC)O
O=C(N(c1ccc(cc1)OC(=O)c1ccco1)C)C(Cl)Cl
NC(=O)C(=Cc1ccc(o1)[N+](=O)[O-])c1ccco1
COC1CC(=C(c2cccs2)c2cccs2)C[N+](C1)(C)C
O=c1n(CCCCN2CCN(CC2)c2ncccn2)ncc(=O)n1C
Clc1ccc(cc1)C(=Cc1ccc(cc1Cl)Cl)Cn1cncc1
COc1ccccc1N1CCN(CC1)CCCC(=O)c1ccc(cc1)F
COc1cc(ccc1OC)C(=O)NCc1ccc(cc1)OCCN(C)C
Cc1ccc2c(c1)nc(o2)NC(c1ccccn1)CC1CCCCC1
OC(=O)C1N2C(=O)C(C2SC1(C)C)N=CN1CCCCCC1
CCn1cc(C(=O)O)c(=O)c2c1nc(cc2)Cc1ccccc1
OCC=Cc1n(C)c2c(c1CO)C(=O)C(=CC2=O)N1CC1
CCN1CCN(C2C1c1c(C)c3c(n1CC2)cccc3)C(C)C
O=C1CCC2(C(=C1)CC1C2CCC2(C1CCC2(C)O)C)C
OC(=O)C(c1ccc2c(c1)nc(o2)c1ccc(cc1)Cl)C
OC(=O)C(OCc1nn(c2c1cccc2)Cc1ccccc1)(C)C
CCCCCCOc1ccc(c2c1cccc2)C(=N)N(CCCC)CCCC
CC(OC(=O)Nc1ccc2c(c1)[nH]c(n2)c1cscn1)C
NC(=NC(=O)c1ccc(c(c1)S(=O)(=O)C)C(C)C)N
O=C1NC(=O)C(S1)Cc1ccc(cc1)OCC1(C)CCCCC1
CC(NC1C2CCC(C1(O)c1ccc(c(c1)Cl)Cl)CC2)C
CN(CCCn1c(=O)n(c2c1ccc(c2)Cl)c1ccccc1)C
CC1CC(C)C(=O)C(C1)C(CC1CC(=O)NC(=O)C1)O
C#CCC12CCC(=O)C=C1CCC1C2CCC2(C1CCC2=O)C
C#CC(c1ccccc1)(c1ccccc1)OC(=O)NC1CCCCC1
Oc1ccc(cc1)C(c1ccccc1C(=O)O)c1ccc(cc1)O
CCC(=C)C(=O)c1ccc(c(c1Cl)Cl)OCC(=O)[O-]
OCCOCCOC(=O)c1ccccc1Nc1cccc(c1)C(F)(F)F
Fc1ccc(cc1)Oc1cccc(c1)C#CC(N(C(=O)N)O)C
CCCNCC(COc1ccc2c(c1)oc(cc2=O)c1ccccc1)O
Fc1ccc(cc1)c1nc(=O)n(c2c1ccc(c2)C)C(C)C
CCOP(=O)(Cc1ccc(cc1)c1nc2c(s1)cccc2)OCC
O=C(NS(=O)(=O)c1ccc2c(c1)CCC2)NC1CCCCC1
CCCCC(CN1CN2C(C1)(C)CN(C2)CC(CCCC)CC)CC
CC(c1ccc(cc1)NC(=O)CN(CC(=O)O)CC(=O)O)C

Continued on next page

Cc1nc2c([nH]1)cc(cc2)C(n1cncc1)c1ccccc1
COc1ccc(cc1OC)C=Nc1ccnc2c1cc(OC)c(c2)OC
CCN(CCNc1ccc(c2c1c(=O)c1c(s2)cccc1)C)CC
O=C1N(CCc2c1c1cccc(c1n2C)F)Cc1nc[nH]c1C
O=C1CC(C)C2(C(C1)CCC1C2CCC2(C1CCC2O)C)C
O=C1NC(=O)C2C1C1C=CC2C2C1C1C2C(=O)NC1=O
c1ccc(cc1)C1CCN(C1)CC1CCCc2c1ccc1c2OCO1
CCC1(O)CCC2C1(CC)CCC1C2CCC2=CC(=O)CCC12
C#CC1(O)CCC2C1(C)CCC1C2CCC2=C1CCC(=O)C2
Clc1ccc(cc1)C(Cn1cncc1)OCc1c(Cl)cccc1Cl
C#CCOCC1COC(O1)(Cn1cncc1)c1ccc(cc1Cl)Cl
CCCCc1c([O-])n(n(c1=O)c1ccccc1)c1ccccc1
O=C(N1c2ccccc2Sc2c1nccc2)OCCOCCN1CCCCC1
O=C1OC(OC1(c1ccccc1)c1ccccc1)CCN1CCCCC1
OC(=O)Cc1nn(cc1c1ccc(cc1)Cl)c1ccc(cc1)F
CCCC(c1ccccc1)(c1ccccc1)C(=O)OCCN(CC)CC
CN1CCCC(C1)OC(=O)C(c1ccccc1)(C1CCCCC1)O
Clc1ccc(cc1)c1nc(c(o1)COC(C(=O)O)(C)C)C
CN(C(=O)c1c(O)c2ccccc2n(c1=O)C)c1ccccc1
CCCCCCCCCCCCCCCCC(=O)OCC(C1OCC(C1O)O)O
Nc1ccc(cc1)S(=O)(=O)Nc1cc(nn1c1ccccc1)C
N#Cc1ccc(n1C)c1ccc2c(c1)C(C)(C)OC(=S)N2
CC(c1cc(CC2SCNC2=O)cc(c1O)C(C)(C)C)(C)C
CCOC(=O)C([N-]CCNC(C(=O)OCC)C[S-])C[S-]
OC1C2OP(=O)(O)OCC2OC1n1c(Cl)nc2c1ncnc2N
COC(=O)C1Oc2ccc(cc2Cc2c(O1)ccc(c2)Cl)Cl
CCN(C(=O)CNC(=O)c1cc(OC)c(c(c1)OC)OC)CC
C=CCONC1=Nc2ccc(cc2C(=NC1)c1ccccc1Cl)Cl
Clc1ccc(s1)C(=NNc1c(Cl)cccc1Cl)Cn1cncc1
N#Cc1ccccc1OCC(CNCCNC(=O)Cc1ccc(cc1)O)O
CN(C(=O)CCCOC(=O)C(Oc1ccc(cc1)Cl)(C)C)C
O=C1CCC2(C(=C1O)CCC1C2CCC2(C1CCC2=O)C)C
COc1ccc2c(c1)C(O)(CCCN(C)C)c1c(S2)cccc1
CN(CC(CN1c2ccccc2S(=O)(=O)c2c1cccc2)C)C
[O-][N+](CCC=C1c2ccccc2CCc2c1cccc2)(C)C
C#CCOC(c1ccccc1)(c1ccccc1)C(=O)OCCN(C)C
O=C1CCC2(C(=C1Cl)CCC1C2CCC2(C1CCC2O)C)C
CCOP(=S)(Oc1ccc2c(c1)oc(=O)c(c2C)Cl)OCC
OC1CCC2(C(=CCC3C2CCC2(C3CCC2(C)O)C)C1)C
OC(=O)c1cc(N=Nc2ccc(c(c2)C(=O)O)O)ccc1O
NC(=N)c1ccc(cc1)OCCCCCOc1ccc(cc1)C(=N)N
CN1Cc2c(ncn2c2c(C1=O)c(Cl)ccc2)C(=O)OCC
CCC12CCCN3C2c2n(C(=O)C1)c1c(c2CC3)cccc1
CC(=O)CC(c1c(=O)oc2c(c1O)cccc2)c1ccccc1
CCCCCCCCCCCC(=O)OCC(OC(=O)CCCCCCCCCCC)C
CC(=CCSc1ccccc1C(=O)O)CCC=C(CCC=C(C)C)C
ClCCC1C(=O)NC2(C1(C)OC2=O)C(C1CCCC=C1)O
Clc1cc(Cl)cc(c1)c1nc2c(o1)cc(cc2)C(=O)O
CC(CC1C(OC(=O)N1CCCN1CCCCCC1)c1ccccc1)C
OCC(C(C1OC(O)(CC(C1NC(=O)C)O)C(=O)O)O)O
O=C(c1ccc(cc1)CN1CCOCC1)N1CCN(CC1)C1CC1
N#CCC(n1ncc(c1)c1ncnc2c1cc[nH]2)C1CCCC1
CCC(Nc1nc(C)nc2n1nc(c2c1ccc(nc1C)OC)C)C
NC(=O)c1cccc2c1nn(c2)c1ccc(cc1)C1CCCNC1
Fc1ccc(cc1)n1c(C)nc(c1C)C#Cc1ccnc(c1)Cl
OCC1OC(CC1OC(=O)C(C(C)C)N)n1ccc(nc1=O)N
CCCCCc1cc(O)c(c(c1)O)C1C=C(C)CCC1C(=C)C
CC(=CC=CC(=CC(=O)O)C)C=CC1=C(C)CCCC1(C)C
CCCCCC(C=CC1C(O)CC2C1CC(=CCCCC(=O)O)O2)O
O=C(NS(=O)(=O)c1ccc(cc1)C(=O)C)NC1CCCCC1
CCCCCc1cc(O)c2c(c1)OC(C1C2C=C(C)CC1)(C)C
Clc1ccc2c(c1)C(=NCc1n2c(C)nn1)c1ccccc1Cl
CC(C(C(=O)OCCOCn1cnc2c1nc(N)[nH]c2=O)N)C
O=C1CCC2(C(=C1)CCC1C2CCC2(C1CCC2(C)O)C)C
CCN(CC#CCOC(=O)C(c1ccccc1)(C1CCCCC1)O)CC
Clc1ccc2c(c1)C(=NCC(=O)N2CC1CC1)c1ccccc1
CCC(=O)OC(c1ccccc1)(C(CN(C)C)C)Cc1ccccc1
OCCN1CCN(CC1)CCCN1c2ccccc2Sc2c1cc(Cl)cc2
CCN(CCNc1ccc(c2c1c(=O)c1c(s2)cccc1)CO)CC
O=C1OCC(=C1c1ccccc1)c1ccc(cc1)S(=O)(=O)C
CCCC(=O)Nc1ccc(c(c1)C(=O)C)OCC(CNC(C)C)O
Cc1ccc(c(c1)C(c1ccccc1)CCN(C(C)C)C(C)C)O
FC(c1cc2NCNS(=O)(=O)c2cc1S(=O)(=O)N)(F)F
OC(=O)C1N2C(=O)CC2S(=O)(=O)C1(C)Cn1ccnn1
O=C(OCC[N+](C)(C)C)CCC(=O)OCC[N+](C)(C)C
COc1ccc2c3c1OC1C43CCN(C(C2)C4(O)CCC1=O)C
Clc1ccc(cc1)CCC(Sc1c(Cl)cccc1Cl)Cn1cncc1
FC(C1(C#CC2CC2)OC(=O)Nc2c1cc(Cl)cc2)(F)F
C#CC1(O)CCC2C1(CC)CCC1C2CCC2=CC(=O)CCC12
N#CC(c1cc(cc(c1)C(C#N)(C)C)Cn1ncnc1)(C)C
C[N+]1(C)CCN(CC1)CC(c1ccccc1)(C1CCCCC1)O

Continued on next page

OCC(c1ccccc1)C(=O)OC1CC2CCC(C1)[N+]2(C)C
CCC1Nc2cc(Cl)c(cc2S(=O)(=O)N1)S(=O)(=O)N
O=C1Nc2ccc(cc2C2(N(C1)CCO2)c1ccccc1Cl)Cl
OCCN1CCN(CC1)C(=O)Cn1c(=O)sc2c1cc(Cl)cc2
OCC(COC(=O)c1ccccc1Nc1ccnc2c1ccc(c2)Cl)O
Cc1cccc(c1)N(C(=S)Oc1ccc2c(c1)C1CCC2C1)C
Clc1ccc2c(c1)C(=NC(C(=O)N2C)O)c1ccccc1Cl
COc1ccc(cc1)c1c(onc1c1ccc(cc1)OC)CC(=O)O
O=C(C1=C(O)c2sccc2S(=O)(=O)N1C)Nc1ccccn1
OC(CSc1scc(n1)c1ccc(s1)C(=O)N)CNC(C)(C)C
O=C1CCc2c(N1)ccc(c2)OCCCCc1nnnn1C1CCCCC1
CC(c1ccc(c2c(c1)c(C)cc2S(=O)(=O)[O-])C)C
Clc1ccc(cc1)SC(P(=O)(O)[O-])P(=O)(O)[O-]
CC[N+](Cc1ccccc1)(CC(=O)Nc1c(C)cccc1C)CC
CCC12CC(=C)C3C(C1CCC2(O)C#C)CCC1=CCCCC31
Nc1ccc(cc1)S(=O)(=O)NC(=O)c1ccc(c(c1)C)C
O=C1CCC(=O)N1CC1CCC2N(C1)CCN(C2)c1ncccn1
CN(CCCN1c2ncccc2N=C(c2c1cccc2)c1ccccc1)C
CN(Cc1ccccc1)CCc1cc2c3n1c1ccccc1CCc3ccc2
OCCC(=C(N(Cc1cnc(nc1N)C)C=O)C)SSCC1CCCO1
COc1ccc(cc1OC)C(CN1CCN(CC1)c1cccccc1=O)O
CC(c1cccc2c1cccc2)NCCCc1cccc(c1)C(F)(F)F
Clc1cccc(c1)C(n1cncc1)c1ccc2c(c1)[nH]cn2
Clc1ccc(cc1)C(c1ccc2c(c1)n(C)nn2)n1ncnc1
CC(CNC1CCS(=O)(=O)c2c1cc(s2)S(=O)(=O)N)C
OCC#CCCCC#CCCCCC1=C(C)C(=O)C(=C(C1=O)C)C
CN(C(C(=O)Nc1c(C)n(n(c1=O)c1ccccc1)C)C)C
COCCOc1cc2c(ncnc2cc1OCCOC)Nc1ccc(cc1)C#C
CCC1(c2ccccc2)C(=O)N(COC)C(=O)N(C1=O)COC
Clc1ccc2c(c1)C(=NCCN2CC(F)(F)F)c1ccccc1F
CCC12C=CC3=C4CCC(=O)C=C4CCC3C1CCC2(O)C#C
OCC1OC(C(C1O)O)n1c(=O)sc2c1[nH]c(N)nc2=O
O=C1CCC2C3(C1)CCN(C2Cc1c3c(O)ccc1)CC1CC1
O=C1CCC2C(=C1)CCC1C2CCC2(C1CC(C2O)(C)C)C
O=C1CCC2C(=C1)CC(C1C2CCC2(C1CCC2(C)O)C)C
CCOC(=O)Nc1cc2NC(C)C(=Nc2c(n1)N)c1ccccc1
COc1c(CC=C(CCC(=O)O)C)c(O)c2c(c1C)COC2=O
COC(=O)Nc1[nH]c2c(n1)cc(cc2)C(=O)c1cccs1
CCC(C(c1ccccc1)(c1ccccc1)CC(NC)C)OC(=O)C
COCC(=O)N(c1ccccc1F)C1CCN(CC1)CCc1ccccc1
CCCCCCCCC=CCCCCCCCC(=O)NCc1ccc(c(c1)OC)O
CCCCC1C(=O)N(N(C1=O)c1ccc(cc1)O)c1ccccc1
O=C(c1cc(Cl)c(cc1OCC1CC1)N)NC1CN2CCC1CC2
CCC(C(c1ccccc1)C(=O)OC1CC[N+](CC1)(C)C)C
CCCCN1CCC(CC1)CNC(=O)c1c2OCCCn2c2c1cccc2
COc1ccc(cc1OC1CCCC1)C(=O)Nc1c(Cl)cncc1Cl
CC1CCC2=C(C1)c1c(OC(=O)C)cc(cc1OC2(C)C)C
OCCN1CCN(CC1)CCCN1c2ccccc2Sc2c1cc(Cl)cc2
COC(=O)c1cnc2c(c1O)cc(c(c2)OC(C)C)OC(C)C
O=C(c1ccc2c(c1)OCCO2)CCN1CCC(C1)c1ccccc1
COC1CCCC2C1=C(C(=O)[O-])N1C2C(C1=O)C(O)C
C[n+]1cc2c3OCOc3ccc2c2c1c1cc3OCOc3cc1cc2
Fc1ccc(cc1)c1cncc(c1)CNCC1CCc2c(O1)cccc2
CCCCCCCCC=CCCCCCCCC(=O)OCC(C1OCC(C1O)O)O
O=C(C1=C(O)c2ccccc2S(=O)(=O)N1C)Nc1nccs1
Nc1ccc(cc1)S(=O)(=O)N(c1onc(c1C)C)C(=O)C
O=c1n(CC2OC2)c(=O)n(c(=O)n1CC1OC1)CC1OC1
O=C(C1C(=O)NC(=O)c2c1cccc2)Nc1ccc(cc1)Cl
CCCC1(O)CCC2C1(C)CCC1C2CCC2=CC(=O)CCC12C
CC(Cc1ccccc1)NC(=O)NS(=O)(=O)c1ccc(cc1)C
N#CC1CCCN1C(=O)CNC12CC3CC(C1)CC(C2)(C3)O
CC(Cc1cccc(c1)C(F)(F)F)NCCOC(=O)c1ccccc1
Fc1ccc(cc1)C(c1ccccc1F)(n1cncc1)c1ccccc1
NC(=N)c1ccc(cc1)OCCCCCCOc1ccc(cc1)C(=N)N
N#Cc1ccc2c(c1)N(CC(CN(C)C)C)c1c(S2)cccc1
O=C(CC(C(=O)O)NC(=O)C)NC(C(=O)O)CCC(=O)O
Oc1ccc(cc1)C1(Oc2ccccc2NC1=O)c1ccc(cc1)O
Nc1ccc(cc1)C(=O)Oc1c(C)c(O)c2c(c1O)cccc2
Clc1ccc2c(c1)C(=NCc1n2c(C)nc1)c1ccccc1Cl
O=C(N1CC(=O)N2C(C1)c1ccccc1CCC2)C1CCCCC1
COc1cc2c(cc1OC)CC1c3c2c(OC)c(OC)cc3CCN1C
CCCCCCCN(CCCC(c1ccc(cc1)NS(=O)(=O)C)O)CC
Oc1cc2OC(c3ccc(c(c3)O)O)C(C(c2c(c1)O)O)O
CN(c1c(C)n(n(c1=O)c1ccccc1)C)CS(=O)(=O)O
O=C1CC2CCC3C(C2(C(=C1)C)C)CCC1(C3CCC1O)C
CCCC[C-]1C(=O)N(N(C1=O)c1ccccc1)c1ccccc1
CNC1C(O)C2OC3OC(C)CC(=O)C3(OC2C(C1O)NC)O
COc1nc(OC)nc(c1)[N-]S(=O)(=O)c1ccc(cc1)N
Cc1ncc(c(n1)N)C[n+]1csc(c1C)CCOP(=O)(O)O
CCCCCCCC(=O)CCC1C(O)CC(C1CC=CCCCC(=O)O)O
Cc1ccc(cc1)c1nc2n(c1CC(=O)N(C)C)cc(cc2)C

Continued on next page

O=C1CCC(C(=O)N1)N1C(=O)c2c(C1=O)c(N)ccc2
COc1cc(ccc1OC)CC(C(Cc1ccc(c(c1)OC)OC)C)C
SCCNCCN(CC1C2CCC(N2C)CC1c1ccc(cc1)Cl)CCS
NC(=N)NC(=O)c1cnn(c1C1CC1)c1cccc2c1cccn2
CC(OC(=O)C(C)C)OC(=O)NCC1(CCCCC1)CC(=O)O
COc1cc(ccc1OC)C1CCN(CC1)CCC1CCOc2c1cccc2
CCOC(=O)c1ccc(cc1)OCCN1CC(O)C(C(C1CO)O)O
FCCCOc1cc2CCN3C(c2cc1OC)CC(C(C3)CC(C)C)O
OC(=O)CSc1nnc(n1c1ccc(c2c1cccc2)C1CC1)Br
Fc1cc(CNCCC2CCOCC2)ccc1Oc1ccc(cn1)C(=O)N
CNC(=O)c1ccc2c(c1)ccc(c2)C1(O)CCn2c1cnc2
CNCc1cc(n(c1)S(=O)(=O)c1cccnc1)c1ccccc1F
OC(CCCCCC(C(=O)O)(C)C)CCCCCC(C(=O)O)(C)C
OCC(C(C(C(C(CC(=O)C(=O)O)O)NC(=O)C)O)O)O
O=C(C1=C(O)c2ccccc2S(=O)(=O)N1C)Nc1ccccn1
OC1CCC2(C(=CCC3C2CCC2(C3CCC2C(=O)C)C)C1)C
CCn1cc(C(=O)O)c(=O)c2c1cc(N1CCNCC1)c(c2)F
CCn1cc(C(=O)O)c(=O)c2c1nc(N1CCNCC1)c(c2)F
COC(=O)Nc1[nH]c2c(n1)cc(cc2)C(=O)c1ccccc1
CC(NC(=O)NS(=O)(=O)c1cnccc1Nc1cccc(c1)C)C
O=C1C=CC2(C(=C1)CCC1C2CCC2(C1CCC2(C)O)C)C
CC12Cc3cn[nH]c3CC1CCC1C2CCC2(C1CCC2(C)O)C
Fc1ccc(c(c1)F)C(C(c1ncncc1F)C)(Cn1cncn1)O
O=C1NC(=O)C(S1)Cc1ccc(cc1)OCCN(c1ccccn1)C
O=C(N1CCCc2c1ccc(c2)OC(=O)c1ccco1)C(Cl)Cl
CCCCCC(C=CC1C(O)CC(C1CC=CCCCC(=O)O)O)(O)C
CC[N+](CCOC(=O)C1c2ccccc2Oc2c1cccc2)(CC)C
COCCCOc1ccnc(c1C)CS(=O)c1[n-]c2c(n1)cccc2
CC1CS(=O)(=O)CCN1N=Cc1ccc(o1)[N+](=O)[O-]
CCc1oc2c(c1C(=O)c1cc(Br)c(c(c1)Br)O)cccc2
OC(=O)c1cc(O)c2c(n1)c(=O)cc1c2nc2ccccc2o1
CCN1CCN(CC1)c1nc2CCCCCCc2c(c1)c1ccc(cc1)F
CCCCCOC(=O)Nc1nc(=O)n(cc1F)C1OC(C(C1O)O)C
O=C1CN2CC(OC2(c2c(N1)ccc(c2)Cl)c1ccccc1)C
Clc1ccc2c(c1)C(=NCC(=O)N2CC1CC1)c1ccccc1F
CC(=CCC1C(=O)N(N(C1=O)c1ccccc1)c1ccccc1)C
CCCCC(=O)OC1CCC2C1(C)CCC1C2CCc2c1ccc(c2)O
O=C1N(Cc2c1cc(c(c2)Cl)S(=O)(=O)N)C1CCCCC1
CC(=O)CCC1C(=O)N(N(C1=O)c1ccccc1)c1ccccc1
O=C(NS(=O)(=O)c1ccc(cc1)C)NN1CC2C(C1)CCC2
COc1ccccc1N1CCN(CC1)CC(COc1cccc2c1cccc2)O
Clc1ccc(c(c1)Nc1ccccc1C(=O)[O-])C(=O)[O-]
OC1C(O)C(OC1n1cnc2c1nc(F)nc2N)COP(=O)(O)O
CCCCCC(C=CC1C(O)CC2C1CC(=C2)CCCCC(=O)OC)O
O=C(Nc1ccc(cc1)S(=O)(=O)Nc1nccs1)CCC(=O)O
FC(c1cc2N=CNS(=O)(=O)c2cc1S(=O)(=O)N)(F)F
OCN(c1ncc(nn1)C=Cc1ccc(o1)[N+](=O)[O-])CO
COCC1CN(C(=O)O1)c1ccc(cc1)OCCC(C(F)(F)F)O
COCC1OC(=O)N(C1)c1ccc(cc1)OCc1cccc(c1)C#N
Clc1ccc(cc1)C1(Cn2cncn2)OC1c1ccc(cc1Cl)Cl
OCCN1CCN(CC1)CCC=C1c2ccccc2Sc2c1cc(Cl)cc2
CN(CCCC1c2ccccc2C(c2c1cc(cc2)C(F)(F)F)C)C
O=c1[nH]c2c(n1C1CCN(CC1)CCOc1ccccc1)cccc2
CN(Cc1nnc2n1c1ccc(cc1C(=NC2)c1ccccc1)Cl)C
CCN(CCC(=NOC(=O)Nc1ccc(cc1)OC)c1ccccc1)CC
CCOC(=O)C1(CCN(CC1)CCc1ccc(cc1)N)c1ccccc1
CCCn1c(=O)n(c2ccc(cc2)Cl)c2c(c1=O)[nH]cn2
COc1ccc(cc1)C(=O)CC(=O)c1ccc(cc1)C(C)(C)C
CCN(CCNC(=O)c1cc(Cl)c(cc1OC(C(=O)C)C)N)CC
Clc1ccc2c(c1)nn(n2)c1cc(C)cc(c1O)C(C)(C)C
CCNC(=O)OC1CCN(CC1)c1nncc2c1cc(OC)c(c2)OC
Nc1[nH]c2c(n1)n(CC1CC1)c(=O)n(c2=O)CC1CC1
O=C(Nc1ccc(c(c1)C(F)(F)F)Cl)Nc1ccc(cc1)Cl
OC(=O)Cc1ccc(cc1)CCNS(=O)(=O)c1ccc(cc1)Cl
CC(C(=O)N(C1CCCC1)CC(=O)O)CSC(=O)C(C)(C)C
O=c1oc2ccccc2c(c1Cc1c(=O)oc2c(c1O)cccc2)O
N#CCC1(O)CCC2C1(C)CCC1=C3CCC(=O)C=C3CCC21
OC(=O)Cc1nn(Cc2ccc(cc2F)Br)c(=O)c2c1cccc2
O=C(c1ccccc1)c1cccc(c1)[N-]S(=O)(=O)C(F)F
CC(Cc1ccccc1)NCCn1cnc2c1c(=O)n(C)c(=O)n2C
Fc1ccc(cc1)C(=O)C1CN(C)CCC1(O)c1ccc(cc1)F
Clc1ccc2c(c1)c(nc(=O)n2CC(F)(F)F)c1ccccc1
[O-]C(=O)C(CCCCOCCCCC(C(=O)[O-])(C)C)(C)C
C#CC1(O)C=CC2C1(CC)CCC1C2CCC2=CC(=O)CCC12
CN1C2CCC1CC(C2)NC(=O)n1c(=O)[nH]c2c1cccc2
COc1ccc(cc1)c1sc(nc1c1ccc(cc1)OC)C(F)(F)F
COC(=O)c1ccc(c2c1nc1c(O)cc(c(c1n2)C=O)O)O
CCN(CCOC(=O)C(Cc1cccc2c1cccc2)CC1CCCO1)CC
CCOP(=O)(On1c(=O)c2cccc3c2c(c1=O)ccc3)OCC
CN1C(=S)SC(C1=O)N=Nc1ccc(cc1)[N+](=O)[O-]
ON=C1CCC2C(=C1)CCC1C2CCC2(C1CCC2(O)C#C)CC

Continued on next page

COC(=O)Nc1[nH]c2c(n1)cc(cc2)S(=O)c1ccccc1
Oc1ccc2c(c1)C13CCCCC3(C(C2)N(CC1)CC1CC1)O
Oc1ccc(cc1)C1(OC(=O)c2c1cccc2)c1ccc(cc1)O
CC(=O)OC(c1ccccc1)(C(CN1CCCC1)C)Cc1ccccc1
n1ccc(cc1)CC1(Cc2ccncc2)c2cccnc2c2c1cccn2
COC(=O)c1cc2[nH]c(nc2cc1C)S(=O)Cc1ncccc1C
Clc1ccc2c(c1)C(=C(c1cccs1)O)C(=O)N2C(=O)N
OC(=O)Cc1sc2n(c1c1ccc(cc1)Cl)c1c(n2)cccc1
OC1C(CCC1Oc1ccccc1)NCC1CCc2c(C1=O)cc(s2)C
Fc1ccc(cc1)C(c1ccc(cc1)F)(c1ccccc1)C(=O)N
[O-]C(=O)CNc1ccc(cc1)S(=O)(=O)c1ccc(cc1)N
CN(C(COC(=O)C(c1ccccc1)(c1ccccc1)O)(C)C)C
CC[N+](CCOC(=O)C(c1cccs1)(C1CCCC1)O)(CC)C
Oc1ccc(cc1)C(=O)NN=Cc1ccc(o1)[N+](=O)[O-]
OC(CNC(C)(C)C)COc1ccc(cc1)NC(=O)NC1CCCCC1
Brc1cc(ccc1OCCCOc1ccc(cc1Br)C(=N)N)C(=N)N
C#CC1(O)CCC2C1(C)C=CC1=C3CCC(=O)C=C3CCC21
[O-][N+](=O)c1ccc(o1)C=NN1CC(=O)N(C1=O)CO
OCCN1C(=O)C(O)N=C(c2c1ccc(c2)Cl)c1ccccc1F
CC(NC(=O)c1cccnc1)CCN1CCN(CC1)c1ccc(cc1)F
CSc1nc2c([nH]1)cc(c(c2)Cl)Oc1cccc(c1Cl)Cl
C1CCN(CC1)CCc1onc(n1)CC(c1ccccc1)c1ccccc1
CCOC(=O)C(=Cc1ncc(n1C)[N+](=O)[O-])C(=O)C
COc1ccc(cc1)N(C(=O)c1ccc(cc1)Cl)CCCC(=O)O
COc1ccc2c3c1OC1C43CC[NH+](C(C2)C4C=CC1O)C
OCC(=O)C1CCC2C1(C)CCC1C2CCC2=CC(=O)CCC12C
CCOc1ccc(cc1OCC)Cc1nccc2c1cc(OCC)c(c2)OCC
C=CCN1CCC23C4C1Cc1c3c(OC2C(C=C4)O)c(cc1)O
Oc1ccc(cc1)C1(c2ccc(cc2)O)C(=O)Nc2c1cccc2
COCCCOc1ccnc(c1C)CS(=O)c1nc2c([nH]1)cccc2
ClCCC(=C(c1ccccc1)c1ccc(cc1)OCCO)c1ccccc1
N#Cc1ccccc1c1cc(cn(c1=O)c1ccccc1)c1ccccn1
COCCCN1CCC(CC1)NC(=O)c1cc(Cl)c(c2c1OCC2)N
FCCOCc1ccc(cc1)COc1cnn(c(=O)c1Cl)C(C)(C)C
Clc1ccc(cc1)CS(=O)(=O)C=Cc1ccc(cc1)C(=O)O
COc1cc(CNCCC2CCOCC2)ccc1Oc1cnc(cn1)C(=O)N
CC1(CSC(=N1)c1cccc(c1O)OCCOCCOCCOC)C(=O)O
BrCCNP(=O)(NCCBr)OCc1cnc(n1C)[N+](=O)[O-]
O=C(C1CCC2CN1C(=O)N2OS(=O)(=O)O)NC1CCNCC1
OCc1ccc(c(c1)C(c1ccccc1)CCN(C(C)C)C(C)C)O
O=S(=O)(c1cnc2c(c1)cccc2N1CCNCC1)c1ccccc1
O=C1CCC2(C(=C1)CCC1C2CCC2(C1CCC2C(=O)C)C)C
O=C1C=CC2(C(=C1)CCC1C2CCC2(C1CCC(=O)O2)C)C
Fc1cc2c(cc1N1CCNCC1)n(cc(c2=O)C(=O)O)C1CC1
CCCCNc1cc(cc(c1Oc1ccccc1)S(=O)(=O)N)C(=O)O
CNC1=Nc2ccc(cc2C(=[N+](C1)[O-])c1ccccc1)Cl
CC(N(C(C)C)CCC(c1ccccn1)(c1ccccc1)C(=O)N)C
OCC1OC(CC1N=[N+]=[N-])n1cc(C)c(=O)[nH]c1=O
CCCCC(CC=CC1C(O)CC(=O)C1CCCCCCC(=O)OC)(O)C
OC=C1CC2(C)C(CC1=O)CCC1C2CCC2(C1CCC2(C)O)C
CC(=O)CC(c1c(=O)oc2c(c1[O-])cccc2)c1ccccc1
ClCC1Nc2cc(Cl)c(cc2S(=O)(=O)N1C)S(=O)(=O)N
Clc1cccc(c1)N1CCN(CC1)CCCn1nc2n(c1=O)cccc2
Oc1ccc2c(c1)C13CCCCC3(C(C2)N(CC1)CC1CCC1)O
COCC1(CCN(CC1)CCc1cccs1)N(c1ccccc1)C(=O)CC
CC(=O)NCC1OC(=O)N(C1)c1ccc(c(c1)F)N1CCOCC1
CCC(=O)OC1CCC2C1(C)CCC1C2CCC2=CC(=O)CCC12C
CCCOC(c1ccccc1)(c1ccccc1)C(=O)OC1CCN(CC1)C
OCC1OC2C(C(C1O)O)OC(=O)c1c2c(O)c(c(c1)O)OC
O=C(N1CCc2c(C1c1ccccc1)cccc2)OC1CN2CCC1CC2
CCOC(=O)C1=CC2(CC)CCCN3C2c2n1c1ccccc1c2CC3
OCC(C(c1ccc(cc1)S(=O)(=O)C)O)NC(=O)C(Cl)Cl
COc1ccccc1OCCNCC(c1ccc(c(c1)S(=O)(=O)N)C)O
CC(=O)OC(OC(=O)C(c1ccc(c(c1)F)c1ccccc1)C)C
COc1c(OC)ccc2c1C13CCN(C(C2)C3(O)CCC(C1)O)C
C[N+]1(CCCCC1)CC1COC(O1)(c1ccccc1)c1ccccc1
CCCCN(C(=O)N1CCOCC1)CCN(C(=O)N1CCOCC1)CCCC
CC(=O)OCC=C(C=CC=C(C=CC1=C(C)CCCC1(C)C)C)C
C#CC1(O)CCC2C1(C)CCC1C2C(C)CC2=C1CCC(=O)C2
OC(COc1cccc2c1OCC(C2)O[N+](=O)[O-])CNC(C)C
CC(C(c1ccccc1)O)N(CCOC(c1ccccc1)c1ccccc1)C
[O-]C(=O)CN(CC(=O)O)CCN(CC(=O)O)CC(=O)[O-]
CCOC(=O)COc1ccc2c(c1)oc(=O)c(c2C)CCN(CC)CC
C[N+]1(CCCCC1)CC1COC(O1)(C1CCCCC1)c1ccccc1
O=C1C(=C(C1=O)NCC=CCOc1nccc(c1)CN1CCCCC1)N
Fc1cc2c(cc1N1CCNCC1)n(cc(c2=O)C(=O)O)C1CC1
[O-]N=C(C([N-]CC(C[N-]C(C(=NO)C)C)(C)C)C)C
COc1ccc2c(c1C(F)(F)F)cccc2C(=S)N(CC(=O)O)C
O=C(Cc1ccccc1)NC1C(=O)N2C1SC(C2C(=O)O)(C)C
COc1cc2OC(C)(C)C=Cc2c2c1c(=O)c1c(n2C)cccc1
CCCCCCCCCCCCCCCC(=O)OCC(C1OC(=O)C(=C1O)O)O

Continued on next page

Table S8 – *Continued from previous page*

CC1CCCC(N1NC(=O)c1ccc(c(c1)S(=O)(=O)N)Cl)C
CC(=O)Oc1ccccc1C(=O)Nc1ncc(s1)[N+](=O)[O-]
CCOC(=O)C1(CCN(CC1)CCC(c1ccccc1)O)c1ccccc1
CN(CCc1c[nH]c2c1cc(cc2)CS(=O)(=O)N1CCCC1)C
C=CCC1(O)CCC2C1(C)C=CC1=C3CCC(=O)C=C3CCC21
CCCCCC(C=CC1C(O)CC(=O)C1CC=CCCCC(=O)O)(O)C
O=C(NCC(=O)NCC(=O)O)CNC(=O)CSC(=O)c1ccccc1
O=C1CC2(CCCC2)CC(=O)N1CCNCC1COc2c(O1)cccc2
CCCCCC(C=CC1C(O)CC(C1CC=CCCCC(=O)OC)O)(O)C
CCCCCC1C(=O)n2n(C1=O)c1ccccc1c(c2)c1ccccc1
Fc1ccc2c(c1)C(OCc1c(F)cccc1F)C(S2)Cn1ccnc1
COc1cc(N)c(cc1C(=O)NC1CCN(CC1)Cc1ccccc1)Cl
COc1ccc2c(c1)CCN1C2CC2C(C1)CCCN2S(=O)(=O)C
O=C(C12CC3CC(C2)CC(C1)C3)NCCc1ccc(c(c1)O)O
O=C(CNC(=O)C(Cc1ccccc1)CSC(=O)C)OCc1ccccc1
OC(=O)CN(CC(=O)O)CCOCCOCCN(CC(=O)O)CC(=O)O
ClC#CC1(O)CCC2C1(C)CCC1=C3CCC(=O)C=C3CCC21
CCn1c(CC)nn(c1=O)CCCN1CCN(CC1)c1cccc(c1)Cl
O=C1NC(Nc2c1cc(c(c2)Cl)S(=O)(=O)N)c1ccccc1
FCC(C(c1ccc(cc1)S(=O)(=O)C)O)NC(=O)C(Cl)Cl
O=C1CC(C)(C)CC(=O)N1CCCCN1CCN(CC1)c1ncccn1
O=C(Cn1cnc2c(c1=O)cc(c(c2)Br)Cl)CC1NCCCC1O
O=C(c1ccccc1)NC1CCN(CC1)CCc1c[nH]c2c1cccc2
COc1ccc(cc1)c1c([nH]c2c1cccc2)c1ccc(cc1)OC
CCN(CCCNC(=O)Cn1ncc(c1c1ccccc1)c1ccccc1)CC
N#CCC1CN(C)C2C(C1)c1cccc3c1c(C2)c([nH]3)Cl
OC(COc1ccc(cc1)C(=O)O)CCc1ccc(cc1)C(C)(C)C
COc1c(OC)c(OC(=O)C)c2c(c1OC(=O)C)ccc(c2)Cl
OC1=C(CCCCCCCC2CCCCC2)C(=O)c2c(C1=O)cccc2
NC(=O)c1cc(cc(c1)[N+](=O)[O-])[N+](=O)[O-]
CCCCC(COC(=O)C(=C(c1ccccc1)c1ccccc1)C#N)CC
Oc1ccc(cc1n1nc2c(n1)cccc2)C(CC(C)(C)C)(C)C
O=C(C(c1ccccc1)Oc1ccccc1)NCCCCN1C(C)CCCC1C
COc1cc2c(cc1OC)ncnc2N1CCN(CC1)C(=O)OCC(C)C
CCOC(=O)c1ccc(cc1)OCCC1CCN(CC1)c1ccc(nn1)C
OC(=O)CCCCON=C(c1cccc(c1)C(F)(F)F)c1cccnc1
CCNC(=O)C1OC(C(C1O)O)n1cnc2c1ncnc2NC1CCCC1
CC(CN(c1ccccc1CS(=O)c1nc2c([nH]1)cccc2)C)C
CC(CCCC(CCCC(C)C)C)CCCCC(CCCC(CCCC(C)C)C)C
CCN(CCNC(=O)N(C(C)C)CCS(=O)(=O)c1ccccc1)CC
C#CCCCC(=O)c1cc(c(c(c1)C(C)(C)C)O)C(C)(C)C
OC(=O)c1ccc2c(c1)c(=O)c1c(o2)ccc(c1)S(=O)C
NC(=O)CCCN=C(c1cc(Cl)cc(c1O)C)c1ccc(cc1)Cl
CC(=O)OC1CCC2C1(C)C=CC1=C3CCC(=O)C=C3CCC21
N#Cc1ccccc1Cn1c(cc(=O)n(c1=O)C)N1CCCC(C1)N
CCOc1cc(ccc1OCC)C=C1NCCc2c1cc(OCC)c(c2)OCC
CCN(CCn1c(=O)c(Cc2ccc(cc2)OC)nc2c1cccc2)CC
COc1ccc(cc1C(=O)NCc1cccnc1)C(=O)NCc1cccnc1
Clc1ccc(cc1)OC(C(=O)OCCOC(=O)c1cccnc1)(C)C
CCN1CCCC1CNC(=O)c1cc(c(cc1OC)N)S(=O)(=O)CC
OC1CCC(CC1)NCc1cc(Br)cc(c1NC(=O)c1cccs1)Br
COC(c1ccccc1)CN1CCN(CC1)CC(C(c1ccccc1)OC)O
COc1ccc2c3c1OC1C43CCN(C(C2)C4CCC1OC(=O)C)C
OC(=O)C(CSCCCCC=C(C(=O)O)NC(=O)C1CC1(C)C)N
Clc1ccc(cc1)NC(=O)c1c(C)nsc1NC(=O)c1ccccc1
OCCOCCN1CCN(CC1)CC(CN1c2ccccc2Sc2c1cccc2)C
O=C1NC(=O)N(C1)N=CC=Cc1ccc(o1)[N+](=O)[O-]
[O-]C(=O)c1cc(c(cc1NCc1ccco1)Cl)S(=O)(=O)N
O=C(c1ccc(c(c1)S(=O)(=O)N)Cl)NN(c1ccccc1)C
CC[N+](CCOC(=O)CCC(=O)OCC[N+](CC)(C)C)(C)C
OC(=O)CCCC=CCC1C(O)CC(C1C=CC(COc1cscc1)O)O
CCOc1ccc(cc1)C(COCc1cccc(c1)Oc1ccccc1)(C)C
O=C(OCc1cccnc1)NCc1ccc(cc1)C(=O)Nc1ccccc1N
COc1ccc2c(c1)ccc(c2)C(C(=O)OCCCCON(=O)=O)C
Nc1ccc(cc1)C(=O)N1CC2CN(CC(C1)C2)Cc1ccccc1
N#CCC(=O)N1CCC(C(C1)N(c1ncnc2c1cc[nH]2)C)C
[O-]C(=O)C(C1CCC2(C(=C(C)C(=O)C=C2)C1O)C)C
Nc1nc(c2ccco2)c2c(n1)n(nn2)Cc1ccc(c(c1)C)N
COc1ccc2c(c1)ccc(c2)C(C(=O)OCCS(=O)(=O)C)C
C=C1CCN(CC1)C(C(c1ccc(cc1F)F)(Cn1cncn1)O)C
CNCc1ccc(cc1)c1[nH]c2c3c1CCNC(=O)c3cc(c2)F
CNS(=O)(=O)CC1CCC(CC1)N(c1[nH]cnc2c1ccn2)C
CC(=O)NCC1OC(=O)N(C1)c1ccc(c(c1)F)N1CCSCC1
CN(C(c1cscc1)C(=O)Nc1ccc2c(c1)cc[nH]c2=O)C
COc1ccccc1OCCNCC(COc1cccc2c1c1ccccc1[nH]2)O
C#CC1(O)CCC2C1(C)CCC1C2CCC2=Cc3c(CC12C)cno3
Clc1ccc2c(c1)C(=NCC(=O)N2CC(F)(F)F)c1ccccc1
Clc1ccc(c(c1)Cl)COC(c1ccc(cc1Cl)Cl)Cn1cncc1
O=C(C(c1ccccc1)(C1CCCC1)O)OC1CC[N+](C1)(C)C
O=C(C(C)C)Nc1ccc(c(c1)C(F)(F)F)[N+](=O)[O-]
CCCC(C(=O)OCC)NC(C(=O)N1C2CCCCC2CC1C(=O)O)C

Continued on next page

```
COCCCN1CC(NCC)c2c(S1(=O)=O)sc(c2)S(=O)(=O)N
COc1cc2c(cc1OC)CC(C2=O)CC1CCN(CC1)Cc1ccccc1
[O-]C(=O)C1N=C(c2ccccc2)c2c(NC1=O)ccc(c2)Cl
O=C1CN2C3CC1CC2CC(C3)OC(=O)c1c[nH]c2c1cccc2
CCC(C(c1ccccc1)(c1ccccc1)CC(N(C)C)C)OC(=O)C
Clc1cc(Cl)c(c(c1O)Cc1c(O)c(Cl)cc(c1Cl)Cl)Cl
OCC(C(C1OC(=CC(C1NC(=O)C)NC(=N)N)C(=O)O)O)O
O=C1CCC2C1(C)CCC1C2CCc2c1ccc(c2)OS(=O)(=O)O
O=C1C=CC2(C(=C1)C(=C)CC1C2CCC2(C1CCC2=O)C)C
CCC(=C(c1ccccc1)c1ccc(cc1)OCCN(C)C)c1ccccc1
Cc1cnc(s1)NC(=O)C1=C(O)c2ccccc2S(=O)(=O)N1C
Cc1ccc(cc1)C1(O)CCN(CC1)CCCC(=O)c1ccc(cc1)F
CCOC(c1ccccc1)CN1CCN(CC1)CC(C(=O)c1ccccc1)C
CCOC(=O)c1ccc(nc1)C#Cc1ccc2c(c1)C(C)(C)CCS2
OC(=O)c1cn2C(C)COc3c2c(c1=O)cc(c3C1(N)CC1)F
O=C1CCC2(C(=C1)C=CC1C2CCC2(C1CCC2C(=O)C)C)C
CCOC(=O)C1N=C(c2c(NC1=O)ccc(c2)Cl)c1ccccc1F
c1ccc(cc1)C=CCN1CCN(CC1)C(c1ccccc1)c1ccccc1
CC[N+](CCOC(=O)C(c1ccccc1)(c1ccccc1)O)(CC)C
O=C1Nc2ccc(cc2C2(N(C1)C(C)CO2)c1ccccc1Cl)Cl
Clc1cc(NCc2cccs2)c(cc1S(=O)(=O)N)c1nnn[nH]1
CCCCCC(C=CC1C(O)CC2C1CC(=CCCCC(=O)[O-])O2)O
CCN1C(=O)c2c(C1Nc1ccc(cc1)OCCN1CCCCC1)cccc2
COc1cc(NC)c(cc1C(=O)NC1CCN(C1C)Cc1ccccc1)Cl
Clc1ccc(c(c1)Cl)C(Cn1cncc1)OCc1c(Cl)cccc1Cl
N#Cc1ccc2c(c1)N(CCCN1CCC(CC1)O)c1c(S2)cccc1
Clc1cc2N(C)C(=O)COc2c(c1)C(=O)NC1CN2CCC1CC2
CC(c1ccc(cc1)C)OC(=O)C1(C)CCC(C1(C)C)C(=O)O
OC(=O)CCCN1CCC(CC1)OC(c1ccccn1)c1ccc(cc1)Cl
COc1cc2nc(nc(c2cc1OC)N)N(CCCNC(=O)C1CCCO1)C
N#CC1(CCC(CC1)C(=O)O)c1ccc(c(c1)OC1CCCC1)OC
CN(c1c(C)n(n(c1=O)c1ccccc1)C)CS(=O)(=O)[O-]
CCN1CC(C(C1=O)(c1ccccc1)c1ccccc1)CCN1CCOCC1
Cc1nc(c(o1)c1ccc(c(c1)F)S(=O)(=O)N)C1CCCCC1
OCC(C(C(C(C(=O)O)O)O)OC1OC(CO)C(C(C1O)O)O)O
CCSc1ccc2c(c1)N(CCCN1CCN(CC1)C)c1c(S2)cccc1
CCC(=O)c1ccc2c(c1)N(CC(N(C)C)C)c1c(S2)cccc1
CN1CCN(CC1)c1c(F)cc2c3c1SCCn3cc(c2=O)C(=O)O
[O-]C(=O)c1ccc(cc1)C=Cc1ccc(o1)[N+](=O)[O-]
O=C1C[N+](=C(c2c(N1)ccc(c2)Cl)c1ccccc1)[O-]
Nc1ccc(cc1)C1=NN(C(=O)C)C(Cc2c1cc1OCOc1c2)C
CCCCC(CC(C=CC1C(O)CC(=O)C1CCCCC=CC(=O)O)O)C
COc1cc(cc(c1OC)OC)C(=O)NCc1ccc(cc1)OCCN(C)C
CC(=O)Nc1ccc(cc1)S(=O)(=O)c1ccc(cc1)NC(=O)C
COc1cc(C)c(c(c1C)C)C=CC(=CC=CC(=CC(=O)O)C)C
OC(=O)C=Cc1cccc(n1)C(=CCN1CCCC1)c1ccc(cc1)C
Cc1ccc2c(c1O)NC(O)C1N(C2=O)C=C(C1)C=CC(=O)N
CCCn1c2nc([nH]c2c(=O)n(c1=O)CCC)C1CCC(=O)C1
OC(C1COc2c(O1)cccc2)CNCC(C1COc2c(O1)cccc2)O
OCC1OC(C(C1O)O)n1cnc2c1nc(NN=CC1CCCCC1)nc2N
Clc1cc(Sc2cc(Cl)cc(c2[O-])Cl)c(c(c1)Cl)[O-]
O=C1CCC2(C(=C1)CC(C1C2CCC2(C1CCC2(C)O)C)C)C
N#Cc1ccccc1OCC(CNC(Cc1c[nH]c2c1cccc2)(C)C)O
CCOC(=O)c1cnc2c(c1O)cc(c(c2)OCC(C)C)OCC(C)C
O=C1NC(=O)N(C1)N=Cc1ccc(o1)c1ccc(c(c1)Cl)Cl
CCOC(=O)c1cnc2c(c1O)cc(c(c2)OCC1CC1)OCC1CC1
OC(=O)c1ccc(cc1)n1nc(nc1c1ccccc1O)c1ccccc1O
[O-]C(=O)C1N2C(=O)CC2S(=O)(=O)C1(C)Cn1ccnn1
NCCCCC(C(=O)O)NC1CCc2c(N(C1=O)CC(=O)O)cccc2
CCOC(=O)C(CC(C(=O)N1C(Cc2c1cccc2)C(=O)O)C)C
CCCCC(CC=CC1C(O)CC(=O)C1CCC=CCCC(=O)OC)(O)C
CCCCCCC(=O)OC1CCC2C1(C)CCC1C2CCc2c1ccc(c2)O
Brc1cc(Br)c(c(c1)C(=O)Nc1cccc(c1)C(F)(F)F)O
CC(=O)C1CCC2C1(C)CCC1C2CCC2C1(C)CCC(C2)(C)O
O=C(NS(=O)(=O)c1ccc(cc1)Cl)Nc1ccc(cc1)N(C)C
O=C(C(c1cccs1)(c1ccccc1)O)OC1CC[N+](C1)(C)C
Clc1ccc(cc1)C(=O)n1cc(c2c1cccc2)Cc1nnn[nH]1
Cc1onc(c1)NC(=O)C1=C(O)c2ccccc2S(=O)(=O)N1C
Cc1ccc(c(c1)NC(c1c(Cl)cccc1Cl)C(=O)N)C(=O)C
OC(=O)CN(CC(=O)Nc1c(C)cc(c(c1C)Br)C)CC(=O)O
O=C(N(c1cnccn1)C1CCN(CC1)CCc1ccccc1)c1ccco1
O=C(c1cccnc1Nc1cccc(c1)C(F)(F)F)OCCN1CCOCC1
COc1c(CC=C(CCC(=O)[O-])C)c(O)c2c(c1C)COC2=O
O=C(NC1C(=O)N2C1SC(C2C(=O)O)(C)C)COc1ccccc1
ClCCCCC(=O)NNC(=O)N1Cc2ccccc2Oc2c1cc(Cl)cc2
O=C(C(c1ccccc1)(c1ccccc1)O)OCC1CCC[N+]1(C)C
O=C(c1cc(c(c(c1)C(C)(C)C)O)C(C)(C)C)c1cccs1
CCCCCC(C=CC1C(O)CC(C1CC=C=CCCC(=O)OC)O)(O)C
FC(Oc1ccc(cc1OCC1CC1)C(=O)Nc1c(Cl)cncc1Cl)F
Cc1cccc(n1)C=NN1CCN(CC1)C(c1ccccc1)c1ccccc1
COc1ccccc1N1CCN(CC1)CCc1c[nH]c2c1cc1OCOc1c2
```

Continued on next page

Table S8 – *Continued from previous page*

COc1ccc(cc1)CCC(NCC(c1ccc(c(c1)S(=O)C)O)O)C
Clc1ccc(cc1)NC(=O)NS(=O)(=O)c1ccc2c(c1)CCC2
O=S1CCC(C1)SC1=C(C(=O)O)N2C(S1)C(C2=O)C(O)C
CC1CC(C)CN(C1)S(=O)(=O)c1ccc(c(c1)C(=O)O)Cl
CCCCCC(=O)CCC1(C)C2Cc3c(C1(C)CCN2C)cc(cc3)O
CCCCCC(CCC1C(O)CC2C1Cc1cccc(c1C2)OCC(=O)O)O
CC(=O)OC1CCC2C1(C)CCC1C2C(C)CC2=CC(=O)CCC12
COc1cc(NCc2ccc3c(c2C)c(N)nc(n3)N)cc(c1OC)OC
O=C(c1cc(c(cc1O)Cl)S(=O)(=O)N)Nc1c(C)cccc1C
O=C(c1cccnc1NCc1ccncc1)Nc1ccc2c(c1)NCC2(C)C
CC[N+](CCOC(=O)C(c1ccccc1)(C1CCCCC1)O)(CC)C
NC(=O)C(c1ccccc1)(c1ccccc1)CC[N+]1(C)CCCCC1
Clc1ccc(cc1)OC(C(=O)OCCCOC(=O)c1cccnc1)(C)C
O=C1CCC2=C3CCC4(C(C3CCC2=C1)CCC4(C)C(=O)C)C
CC(CCOc1ccc(cc1)NC(=S)Nc1ccc(cc1)OCCC(C)C)C
N#CCCN1C(=O)C(O)N=C(c2c1ccc(c2)Cl)c1ccccc1F
COc1ccc2c3c1OC1C43CCN(C(C2)C4CC=C1OC(=O)C)C
OC(=O)COCCN1CCN(CC1)C(c1ccc(cc1)Cl)c1ccccc1
C=CCC1=C(C)C(CC1=O)OC(=O)C1C(C1(C)C)C=C(C)C
CCC(COC(c1ccccc1)(c1ccccc1)C(=O)OCCN(C)C)CC
O=C(Nc1cccc(c1)C1=NCCN1)Nc1cccc(c1)C1=NCCN1
N#Cc1cc(NC(=O)C(=O)O)c(c(c1)NC(=O)C(=O)O)Cl
CCN(CCCC(Nc1c2cc(OC)ccc2nc2c1ccc(c2)Cl)C)CC
COc1cc(Cc2cnc(nc2N)N)c2c(c1OC)OC(C=C2)C1CC1
CC(=CC1C(C1(C)C)C(=O)OCc1coc(c1)Cc1ccccc1)C
CCC(=O)OCC=C(C=CC=C(C=CC1=C(C)CCCC1(C)C)C)C
CCOc1ccc(cc1)c1cc(cn1c1ccc(cc1)S(=O)(=O)N)C
COC(=O)C1(O)CC2(CC)CCCN3C2c2n1c1ccccc1c2CC3
CCCCCCCCC=CCCCCCCCCOC(=O)CCCCCCCC=CCCCCCCCC
OC(=O)COc1cc(C)c(c(c1)C)Cc1ccc(c(c1)C(C)C)O
CCC(C(n1cncn1)c1ccc(cc1)Nc1nc2c(s1)cccc2)CC
O=C(Cc1csc(n1)N)Nc1ccc(cc1)CCNCC(c1ccccc1)O
COc1ccc(cc1c1c(Br)cnn1C)NC(=O)Nc1ccc(cc1F)F
CCC(CC1(CCCCC1)C(=O)Nc1ccccc1SC(=O)C(C)C)CC
O=C(c1ccc2c(c1)NCCCC2)CCC1CCN(CC1)Cc1ccccc1
[O-]C(=O)CSc1nnc(n1c1ccc(c2c1cccc2)C1CC1)Br
ONC(=O)C=Cc1ccc(cc1)CNCCc1c(C)[nH]c2c1cccc2
CC=CC1C(C1(C)C)C(=O)OCc1c(F)c(F)c(c(c1F)F)C
O=C(Nc1cc(Br)c(cc1OCC1CNCCO1)C)Nc1cnc(cn1)C
OCCONC(=O)c1cc2n(C)cnc2c(c1Nc1ccc(cc1F)Br)F
OCC(C(c1ccc(cc1)[N+](=O)[O-])O)NC(=O)C(Cl)Cl
Fc1ccc(cc1)C(=O)CCCN1CCC(CC1)(O)c1ccc(cc1)Cl
CCC1C2CC3N(C1O)C1C2C(O)C2(C3N(C)c3c2cccc3)C1
COC1=CC(=O)CC(C21Oc1c(C2=O)c(OC)cc(c1Cl)OC)C
NC(c1ccccc1)C(=O)NC1C(=O)N2C1SCC(=C2C(=O)O)C
O=C1NC(c2c1cccc2)(O)c1ccc(c(c1)S(=O)(=O)N)Cl
CCN(CCN1C(=O)CN=C(c2c1ccc(c2)Cl)c1ccccc1F)CC
CCOC(=O)N1CCC(=C2c3ccc(cc3CCc3c2nccc3)Cl)CC1
CC1Nc2cc(Cl)c(cc2C(=O)N1c1ccccc1C)S(=O)(=O)N
COc1ccc2c(c1)nc([nH]2)S(=O)Cc1ncc(c(c1C)OC)C
Clc1ccc2c(c1)C(=NCC(=S)N2CC(F)(F)F)c1ccccc1F
[O-][N+](=O)OC(CO[N+](=O)[O-])CO[N+](=O)[O-]
O=C1CN=C(c2c(N1)ccc(c2)[N+](=O)[O-])c1ccccc1
NS(=O)(=O)OCC12OCC3C(C2OC(O1)(C)C)OC(O3)(C)C
C#CC1(O)CCC2C1(C)CCC1C2CCc2c1ccc(c2)OC1CCCC1
O=C1N(Cc2ccccc2)C2C(N1Cc1ccccc1)C[S+]1C2CCC1
CCOC(=O)C(NC(C(=O)N1CCCC1C(=O)O)C)CCc1ccccc1
CNS(=O)(=O)CCc1ccc2c(c1)c(c[nH]2)C1CCN(CC1)C
O=C1CC2(CCCC2)CC(=O)N1CCCCN1CCN(CC1)c1ncccn1
Cc1ccc(cc1)C(=O)c1cc(O)c(c(c1)[N+](=O)[O-])O
OC(c1cc(nc2c1cccc2C(F)(F)F)C(F)(F)F)C1CCCCN1
OC1CCC2(C34C1Oc1c4c(CC2N(CC3)CC2CCC2)ccc1O)O
CCOc1ccccc1OCCNC(Cc1ccc(c(c1)S(=O)(=O)N)OC)C
[O-][N+](=O)c1ccc(c(c1)Oc1ccccc1)NS(=O)(=O)C
Fc1ccc(cc1)C(=O)CCCN1CCC(CC1)(O)c1ccc(cc1)Br
CCCCC(CC(C=CC1C(O)CC2C1CC(C2)CCOCC(=O)OC)O)C
COc1ccc2c(c1)nc([n-]2)S(=O)Cc1ncc(c(c1C)OC)C
Oc1ccc2c(c1)Oc1c(C32OC(=O)c2c3cccc2)ccc(c1)O
OCC(COC(=O)c1ccccc1Nc1ccnc2c1cccc2C(F)(F)F)O
OCCN1C(=O)CN2C(c3c1ccc(c3)Cl)(OCC2)c1ccccc1F
COc1ccccc1N1CCN(CC1)CCCNc1cc(=O)n(c(=O)n1C)C
OC(=O)C1N2C(=O)C(C2SC1(C)C)NC(=O)C1(N)CCCCC1
C=CCN1CCC23C4(C1Cc1c3c(OC2C(=O)CC4)c(cc1)O)O
Clc1ccc(cc1)C(=O)NCCc1ccc(cc1)OC(C(=O)O)(C)C
O=C(C(c1ccccc1)(c1ccccc1)O)OCC1CCCC[N+]1(C)C
CC(c1ccc2c(c1)c(=O)c1c(o2)nc(c(c1)C(=O)O)N)C
C#CCN1CC(=O)N(C1=O)COC(=O)C1C(C1(C)C)C=C(C)C
COc1ccc2c(n1)nc([nH]2)S(=O)Cc1ncc(c(c1C)OC)C
CCC(C(c1ccc(cc1)OC(=O)C)CC)c1ccc(cc1)OC(=O)C
Clc1cc2NC(CC3CCCC3)NS(=O)(=O)c2cc1S(=O)(=O)N
O=C1CCC2(C34C1Oc1c4c(CC2N(CC3)CC2CC2)ccc1O)O

Continued on next page

C=C1CCC2(C34C1Oc1c4c(CC2N(CC3)CC2CC2)ccc1O)O
OCC(c1ccccc1)C(=O)OC1CC2CCC(C1)[N+]2(C)C(C)C
CC#CC1(O)CCC2C1(C)CCC1C2CC(C2=CC(=O)CCC12C)C
CCn1cc(C(=O)O)c(=O)c2c1cc(N1CCN(CC1)C)c(c2)F
COc1cc(ccc1Nc1c2ccccc2nc2c1cccc2)NS(=O)(=O)C
OCC(OCn1cnc2c1[nH]c(N)nc2=O)COC(=O)C(C(C)C)N
O=C1OC(CN1N=Cc1ccc(o1)[N+](=O)[O-])CN1CCOCC1
OCCC1CCN(CC1)CCCN1c2ccccc2Sc2c1cc(cc2)C(=O)C
O=C(c1ccsc1)NCC1CN=C(c2c(N1C)cccc2)c1ccccc1F
CC#CCC(C(C=CC1C(O)CC2C1CC(=CCCCC(=O)O)C2)O)C
COC(=O)CCCC=CCC1C(O)CC(C1C#CC(CCC1CCCCC1)O)O
C=CCSCC1Nc2cc(Cl)c(cc2S(=O)(=O)N1)S(=O)(=O)N
CNn1cc(C(=O)O)c(=O)c2c1cc(N1CCN(CC1)C)c(c2)F
CC(CC1Nc2cc(Cl)c(cc2S(=O)(=O)N1)S(=O)(=O)N)C
OCC(=O)C1(O)CCC2C1(C)CCC1C2CCC2=CC(=O)CCC12C
CC(c1cc(C=C2SC(=N)NC2=O)cc(c1O)C(C)(C)C)(C)C
CC(C1(O)CCN2C(C1)c1cccc3c1C(C2)c1ccccc1CC3)C
CCC1(OCC(c2c1[nH]c1c2cccc1)Cc1ccccc1)CC(=O)O
ONC(=O)C(C(C(=O)NC(C(C)(C)C)C(=O)NC)CC(C)C)O
CC(c1cccc(c1NC(=O)CN(CC(=O)O)CC(=O)O)C(C)C)C
C#CC1(O)CCC2C1(CC)CC(=C)C1C2CCC2=CC(=O)CCC12
COc1ccc(cc1C1(F)C(=O)Nc2c1ccc(c2)C(F)(F)F)Cl
COC(=O)Nc1[nH]c2c(n1)cc(cc2)C(=O)c1ccc(cc1)F
COc1ccc(cc1)c1[nH]c(nc1c1ccc(cc1)OC)C(F)(F)F
Clc1ccc2c(c1)C(=NCC(=O)N2CP(=O)(C)C)c1ccccc1
COc1cccnc1CCCCNc1[nH]cc(c(=O)n1)Cc1ccc(nc1)C
NC(=O)N1C(=O)C(=C(c2cccs2)O)c2c1cc(Cl)c(c2)F
OC(=O)COc1cc2CC(C(=O)c2c(c1Cl)Cl)(C)c1ccccc1
O=C1CC2C(O1)C1=C(C(O2)C)C(=O)c2c(C1=O)cccc2O
CC(N1CCC(CC1)N(c1ccc(cc1)Cl)C(=O)Cc1ccccc1)C
OCC1OC(C(C1O)O)n1c(NC(C)C)nc2c1cc(Cl)c(c2)Cl
CCCCc1cc2c(cc1OCc1ccccc1)[nH]cc(c2=O)C(=O)OC
COc1ccc2c(c1)c1CC(CCc1n2C(=O)c1ccccc1)C(=O)O
CON=C(c1csc(n1)N)C(=O)NC1C(C)N(C1=O)OCC(=O)O
CC(n1c(=O)c2c(ncn2c2c1cccc2)c1noc(n1)C1CC1)C
O=c1[nH]c2ccccc2c(=O)n1CCCN1CCN(CC1)c1ccccc1
CC(=CC=Cc1ccc(cc1)C(=O)O)C=CC1=C(C)CCCC1(C)C
FC(C(C(F)(F)F)(F)F)(C(C(C(F)(F)F)(F)F)(F)F)F
NC(=O)C(c1ccccc1)(c1ccccc1)CCN1CCN2C(C1)CCC2
Clc1cccc(c1)C1CCOP(=O)(O1)COCCn1cnc2c1ncnc2N
CC[n+]1c(C=CC=c2ccc3c(n2CC)cccc3)ccc2c1cccc2
Clc1ccc2c(c1)C(=NCCN2C1=NC(=O)CO1)c1ccccc1Cl
COC(=O)c1c2C3CN(C)CCN3c3c(Cn2c2c1cccc2)cccc3
NC(CC1OC(C(C1O)O)n1cnc2c1ncnc2N)CCC(C(=O)O)N
[S-]CC(=O)[N-]CC(=O)[N-]CC(=O)[N-]CC(=O)[O-]
OCCOC(=O)c1c(C)[n+]([O-])c2c([n+]1[O-])cccc2
Clc1ccc2c(c1)C(=C(c1cccs1)[O-])C(=O)N2C(=O)N
Nc1nc(Sc2n(C)cnc2[N+](=O)[O-])c2c(n1)nc[nH]2
Clc1ccc(cc1)Sc1c(C)cc(cc1C)n1ncc(=O)[nH]c1=O
OCCNCCn1nc2c3c1ccc(c3c(=O)c1c2cncc1)NCCN(C)C
C#CC1(O)C=CC2C1(C)CC(=C)C1C2CCC2=CC(=O)CCC12
OC1CCCCC1N1CCC(CC1)(N(C)C)C(=O)Nc1c(C)cccc1C
CN1C(=O)CC(=O)N(c2c1ccc(c2)C(F)(F)F)c1ccccc1
OC1C(O)C(OC1n1cnc2c1ncnc2N)COP(=O)([O-])[O-]
C=C1CC(C)C2C3(C1)CCN(C2Cc1c3cc(O)cc1)CC1CCC1
CN1C2CCC1CC(C2)NC(=O)c1cc(Cl)cc2c1OC(C2)(C)C
COc1ccc(cc1F)NS(=O)(=O)c1c(F)c(F)c(c(c1F)F)F
OC(=O)C(c1ccc(cc1)OCc1ccc2c(n1)cccc2)C1CCCC1
Clc1ccc(c(c1)Cl)C(Cn1ccnc1)OCc1csc2c1cccc2Cl
Cc1ccccc1CN1CCN(CC1)CCOC(c1ccccc1Cl)c1ccccc1
CCOC(=O)COc1ccc2c(c1Cl)oc(=O)c(c2C)CCN(CC)CC
CC(C(c1ccccc1)O)NCCn1cnc2c1c(=O)n(C)c(=O)n2C
Clc1ccc(c(c1)Cl)Cc1cc(ccc1O)C(CC(C)(C)C)(C)C
OC1CCC2(C(C1)CCC1C2C(=O)CC2(C1CCC2C(=O)C)C)C
C=CCN1CCCC1CNC(=O)c1cc(cc(c1OC)OC)S(=O)(=O)N
COc1cc(cc(c1OCCN1CCOCC1)OC)C(=O)c1ccc(cc1)Cl
O=C1N(COP(=O)(O)O)C(=O)NC1(c1ccccc1)c1ccccc1
OCCNC(=O)c1c(C)[n+]([O-])c2c([n+]1[O-])cccc2
CC(=CC1C(C1(C)C)C(=O)OCc1cccc(c1)Oc1ccccc1)C
O=C(C(c1cccc(c1)C(=O)c1ccccc1)C)Nc1nccc(c1)C
[O-]C(=O)c1cc(O)c2c(n1)c(=O)cc1c2nc2ccccc2o1
CCC(=O)C1(C)CCC2C1(C)CCC1=C3CCC(=O)C=C3CCC21
N#Cc1c(sc(c1CC(=O)O)C(=O)O)N(CC(=O)O)CC(=O)O
Cc1ccc(cc1)S(=O)(=O)Oc1ccc(c(c1)S(=O)(=O)O)O
CN1CCN(CC1)CCCN1c2ccccc2Sc2c1cc(cc2)C(F)(F)F
CCC(C1CCC2C1(C)CCCC2=CC=C1CC(O)C(=C)C(C1)O)C
Clc1ccc(cc1)C(=O)c1ccc(cc1)OC(C(=O)[O-])(C)C
O=C(NC(C(=O)O)Cc1c[nH]c2c1cccc2)CCC(C(=O)O)N
OCC(C(C1OC(=CC(C1NC(=O)C)NC(=N)N)C(=O)O)OC)O
OCCONC(=O)c1cc2n(C)cnc2c(c1Nc1ccc(cc1Cl)Br)F
OCc1cc(ccc1O)C(CNCCCCCCOCCOCc1c(Cl)cccc1Cl)O

Continued on next page

Nc1ccc(cc1)S(=O)(=O)N(c1ccnn1c1ccccc1)C(=O)C
CCCCCCCC(=O)NC(C(c1ccc2c(c1)OCCO2)O)CN1CCCC1
ClCCC(=C(c1ccccc1)c1ccc(cc1)OCCOCCO)c1ccccc1
Clc1ccc2c(c1)C(CC2c1ccccc1)N1CCN(C(C1)(C)C)C
Clc1ccc(cc1)CS(=O)(=O)C=Cc1ccc(cc1)C(=O)[O-]
[O-]C(=O)C1(C)CSC(=N1)c1cccc(c1O)OCCOCCOCCOC
OC(=O)CNC(=O)c1nc(C)c2c(c1O)ccc(c2)Oc1ccccc1
O=C1OC(C(N1)C(=O)NC(C(=O)N1CCCC1C)Cc1cscn1)C
O=C(c1cc2c(o1)cccc2)NC1C2CCN(C1Cc1cccnc1)CC2
CC(CCCC(C1CCC2C1(C)CCCC2=CC=C1CC(O)CCC1=C)C)C
NC(c1ccccc1)C(=O)NC1C(=O)N2C1SC(C2C(=O)O)(C)C
CC(=O)Oc1ccc(cc1)C(c1ccccn1)c1ccc(cc1)OC(=O)C
NC(c1ccccc1)C(=O)NC1C(=O)N2C1SCC(=C2C(=O)O)Cl
COc1ccc(cc1)C(=C(c1ccc(cc1)OC)Cl)c1ccc(cc1)OC
CCOC(=O)C=C(C=CC=C(C=Cc1c(C)cc(c(c1C)C)OC)C)C
O=S(c1nc2c([nH]1)cccc2)Cc1nccc(c1C)OCC(F)(F)F
Clc1ccc(c(c1)C(=O)Nc1ccc(cc1Cl)[N+](=O)[O-])O
COc1cc2nc(nc(c2cc1OC)N)N1CCN(CC1)C(=O)c1ccco1
O=C(c1cc(OCC(F)(F)F)ccc1OCC(F)(F)F)NCC1CCCCN1
ClC(C1Nc2cc(Cl)c(cc2S(=O)(=O)N1)S(=O)(=O)N)Cl
CN1CCCC(CC1)n1nc(Cc2ccc(cc2)Cl)c2c(c1=O)cccc2
OCC(c1ccccc1)C(=O)OC1CC2C3C(C(C1)[N+]2(C)C)O3
O=C(c1cccc2c1oc(c1ccccc1)c(c2=O)C)OCCN1CCCCC1
O=C(C(c1ccccc1)(c1ccccc1)O)OC1CCC[N+](C1)(C)C
[O-]C(=O)c1cc(N=Nc2ccc(c(c2)C(=O)[O-])O)ccc1O
Clc1ccc(c(c1)Cl)CON=C(c1ccc(cc1Cl)Cl)Cn1cncc1
NC(c1ccccc1)C(=O)NC1C2CCC(=C(N2C1=O)C(=O)O)Cl
C#CC1(CCC2C1(C)CCC1C2CCC2=CC(=O)CCC12)OC(=O)C
CCN(CCOc1ccc(cc1)C(=C(c1ccccc1)Cl)c1ccccc1)CC
ClCCC(=C(c1ccccc1)c1ccc(cc1)OCCN(C)C)c1ccccc1
CC(C1C(=O)N2C1CC(=C2C(=O)O)SC1CCN(C1)C(=N)C)O
O=C(Cc1ccccc1)NC1C(=O)N2C1SC(C2C(=O)[O-])(C)C
O=C(CS(=O)Cc1ccco1)NCC=CCOc1nccc(c1)CN1CCCCC1
Oc1ccc(cc1)C1(OS(=O)(=O)c2c1cccc2)c1ccc(cc1)O
COc1cc2c(cc1OC)[nH]c(c2CCN1CCN(CC1)c1ccccc1)C
Clc1cc2NC(Cc3ccccc3)NS(=O)(=O)c2cc1S(=O)(=O)N
Cc1cc(O)c2c(c1)OC1(C3C2OCC23CCCC(C2CC1)(C)C)C
Fc1ccc(c(c1)F)C(OP(=O)(O)O)(Cn1cncn1)Cn1cncn1
CCCCCCCC(=O)CCC1C(O)CC(C1CC=CCCCC(=O)OC(C)C)O
CN(Cc1c(Cl)cccc1NC(=O)c1ccccc1)CC(=O)N1CCOCC1
CN1C(=O)CN=C(c2c1ccc(c2)[N+](=O)[O-])c1ccccc1
CCOC(=O)OC(c1ccnc2c1cc(OC)cc2)C1CC2CCN1CC2C=C
Clc1ccc(cc1)OCCOC(=C(n1cncc1)C)c1ccc(cc1Cl)Cl
[O-]S(=O)(=O)c1c2ccc(cc2ccc1C(C)(C)C)C(C)(C)C
O=C(C1C(C1(C)C)C=C(Cl)Cl)OCc1c(F)c(F)cc(c1F)F
O=C(c1ccccc1)Oc1ccc2c(c1)CCC1C2CCC2(C1CCC2O)C
O=C1ONC(=O)C1Cc1ccc(cc1)OCCc1nc(oc1C)c1ccccc1
CC[N+]1(CCOC(=O)C(c2ccccc2)(c2ccccc2)O)CCCCC1
COCc1c(F)c(F)c(c(c1F)F)COC(=O)C1C(C1(C)C)C=CC
OC(=O)C(CSCCCCC=C(C(=O)[O-])NC(=O)C1CC1(C)C)N
O=C(c1ccc(cc1)F)CCCN1CCC(CC1)(N1CCCCC1)C(=O)N
CC(=O)N(c1cccc(c1)c1ccnc2n1ncc2C(=O)c1cccs1)C
Fc1ccc(cc1)C(=O)CCCN1CCC(CC1)C(=O)c1ccc(cc1)F
CCNC(=O)CCCC=CCC1C(O)CC(C1C=CC(CCc1ccccc1)O)O
CCCCC(C(C=CC1C(O)CC(=C)C1CC=CCCCC(=O)O)O)(C)C
O=C1CCC(C(=O)N1)(C1CCN(CC1)Cc1ccccc1)c1ccccc1
O=C(c1ncn2c1C1CCCN1C(=O)c1c2cccc1Br)OC(C)(C)C
CNC(=O)c1ccccc1Sc1ccc2c(c1)[nH]nc2C=Cc1ccccn1
CC(=O)C(=Cc1cc(O)c(c(c1)[N+](=O)[O-])O)C(=O)C
COc1ccc2c3c1OC1C43CCN(C(C2)C4CCC1=NOCC(=O)O)C
Clc1cnc(c(c1)c1ccc(cc1)S(=O)(=O)C)c1ccc(nc1)C
O=C1OC(C(=C1OCC1CC1)c1ccc(cc1)S(=O)(=O)C)(C)C
CCC(=O)NS(=O)(=O)c1ccc(cc1)c1c(C)onc1c1ccccc1
CCOP(=O)(Cc1ccc(cc1)C(=O)Nc1ccc(cc1C#N)Br)OCC
O=C1Nc2c(C1)cc1c(c2)onc1CCC1CCN(CC1)Cc1ccccc1
OC(=O)Cn1cc(c2c1cccc2)Cc1sc2c(n1)c(F)c(cc2F)F
[O-]C(=O)CN(CC(=O)[O-])CCN(CC(=O)O)CC(=O)[O-]
O=C(Nc1cc(C)cc(c1)C)Cc1ccc(cc1)OC(C(=O)O)(C)C
Clc1ccc(cc1)Cn1c2=NC(Cn2c(=O)c2c1nc[nH]2)(C)C
COC(=O)CCC=C=CCC1C(O)CC(C1C=CC(COc1ccccc1)O)O
SC(C(=O)NC1CCCC(N(C1=O)CC(=O)O)(C)C)Cc1ccccc1
COc1cc2c(cc1OC)ncnc2N1CCN(CC1)C(=O)OCC(O)(C)C
O=C1c2ccccc2S(=O)(=O)N1CCCCN1CCN(CC1)c1ncccn1
O=C(Nc1ccc(cc1)C(=O)[O-])CCn1cnc2c1nc[nH]c2=O
CCOC(=O)C(=O)Nc1cc(C#N)cc(c1Cl)NC(=O)C(=O)OCC
NCC(=O)NCC(=O)N(c1ccc(cc1C(=O)c1ccccc1Cl)Cl)C
CCCCC(C1(O)CCC2C(O1)CC(=O)C2CCCCCCC(=O)O)(F)F
C=CCSCC1Nc2cc(Cl)c(cc2S(=O)(=O)N1C)S(=O)(=O)N
CCCc1c2nc(cc(c2cc2c1oc(cc2=O)C(=O)O)NC)C(=O)O
CCNC(=O)C=C(C=CC=C(C=Cc1c(C)cc(c(c1C)C)OC)C)C
O=C1OC(CN1N=Cc1ncc(n1C)[N+](=O)[O-])CN1CCOCC1

Continued on next page

O=C1NC(=O)C(S1)Cc1ccc2c(c1)ccc(c2)OCc1ccccc1F
Cc1nnc(o1)C(c1ccccc1)(c1ccccc1)CCN1CC2CCC1CC2
O=C(C(c1ccccc1)(c1ccccc1)O)OC1CC[N+](CC1)(C)C
OC(=O)c1ccc(cn1)C(c1ccccc1)(c1ccccc1)c1ccccc1
COc1ccc(cc1)C(=O)[N-]c1on[n+](c1)N1C(C)CCCC1C
Cc1noc(c1)CCCOc1c(C)cc(cc1C)c1noc(n1)C(F)(F)F
COc1ccc(cc1)C(=O)c1c(C)n(c2c1cccc2)CCN1CCOCC1
Fc1ccc(cc1)Nc1nc(c(c(n1)C)C)N1CCc2c(C1C)cccc2
Cn1nc(nc1NCCCOc1cccc(c1)CN1CCCCC1)CS(=O)(=O)C
COP(=S)(Oc1ccc(cc1)Sc1ccc(cc1)OP(=S)(OC)OC)OC
COc1ccc(cc1)n1nc(cc1c1ccc(cc1)Cl)CCC(=O)N(O)C
CCCCCC(C(c1cc(O)c2c(c1)OC(C1=C2CCCS1)(C)C)C)C
CCCCC(C(C=CC1C(C)CC(=O)C1CC=CCCCC(=O)O)O)(C)C
CCCCC(CC=CC1C(O)CC(=O)C1CC=CCCCC(=O)OC)(C=C)O
CC(Oc1ccc2c(c1)c(=O)c1c(o2)ccc(c1)C(=O)[O-])C
CCOC(=O)CC(NC(=O)CCC(=O)Nc1ccc(cc1)C(=N)N)C#C
CC(CCC(=O)CCC1(C)C2Cc3c(C1(C)CCN2C)cc(cc3)O)C
COc1cc2c(ncnc2cc1OCC1CCN(CC1)C)Nc1ccc(cc1F)Br
CONC(=N)c1ccc(cc1)c1ccc(o1)c1ccc(cc1)C(=N)NOC
O=C(C1C(=O)c2c(C1=O)cccc2)C(c1ccccc1)c1ccccc1
CCOC(=O)Nc1ccc2c(c1)N(C(=O)CN(C)C)c1ccccc1CC2
CCCN1CC(CC2C1Cc1cccc(c1C2)O)NS(=O)(=O)N(CC)CC
Clc1ccc(cc1)OCC(=O)N1CCN(CC1)Cc1ccc2c(c1)OCO2
CC(=O)OCC1=C(N2C(SC1)C(C2=O)NC(=O)CC#N)C(=O)O
CC(=O)OC1CCC2C1(C)CCC1C2CCC2=C(C(=O)CCC12C)Cl
[O-][N+](=O)c1cc(C(=O)N)c(c(c1)[N+](=O)[O-])C
OC(=O)CCC(=O)OC1CCC2C1(C)CCC1C2CCc2c1ccc(c2)O
O=C1CCC2(C(=C1)CCC1C2CCC2(C1CCC2(O)C(=O)C)C)C
Clc1ccc(cc1)C(c1ccccc1)N1CCN(CC1)Cc1cccc(c1)C
CN1CCN(CC1)CC(=O)n1c2ccccc2c(=O)[nH]c2c1nccc2
COc1cc2nc(nc(c2cc1OC)N)N1CCN(CC1)C(=O)C1CCCO1
OCC(C(C(F)(F)F)C(F)(F)F)NS(=O)(=O)c1ccc(s1)Cl
CCOc1ccc(cc1)Cc1cc(ccc1Cl)C1OC(CO)C(C(C1O)O)O
O=C(c1ccc(cc1)CNc1nccc(n1)c1cccnc1)Nc1ccccc1N
Clc1ccc(c(c1)Cl)C(=O)[N-]S(=O)(=O)c1ccc(s1)Br
COc1cc(C=Cc2ccc(c(c2)OP(=O)(O)O)OC)cc(c1OC)OC
O=C(N1CCC(C1)(F)F)C1NCC(C1)N1CCN(CC1)c1ncccn1
CC(=O)OCOC(=O)C1=C(COC(=O)N)SC2N1C(=O)C2C(O)C
N#Cc1ccc(cc1Cn1c(cc(=O)n(c1=O)C)N1CCCC(C1)N)F
CCOc1ccc(cc1)Cc1cc(ccc1Cl)C1OC(SC)C(C(C1O)O)O
COc1ccc2c(c1)c(CC(=O)O)c(n2C(=O)c1ccc(cc1)Cl)C
O=C(C(C1=CCC=CC1)N)NC1C(=O)N2C1SCC(=C2C(=O)O)C
O=C1CN=C(c2c(N1)ccc(c2)[N+](=O)[O-])c1ccccc1Cl
O=C1CCC2C1(C)CCC1C2CCc2c1ccc(c2)OS(=O)(=O)[O-]
O=C1CCC2(C(=C1)CCC1C2(F)C(O)CC2(C1CCC2(C)O)C)C
CC1Cc2c(N1NC(=O)c1ccc(c(c1)S(=O)(=O)N)Cl)cccc2
CN1CCN(CC1)c1c(F)cc2c3c1OCC(n3cc(c2=O)C(=O)O)C
O=C(NC1C(=O)N2C1SC(C2C(=O)[O-])(C)C)COc1ccccc1
CC(OC(=O)C(Oc1ccc(cc1)C(=O)c1ccc(cc1)Cl)(C)C)C
CCC(NC(=O)C1CN(C)C2C(=C1)c1cccc3c1c(C2)cn3C)CO
O=C(C(c1ccccc1)(c1ccccc1)O)OC1C[N+]2(C)CCC1CC2
CCCCCCC(=O)OC1CCC2C1(C)CCC1C2CCC2=CC(=O)CCC12C
COc1ccc(cc1)c1nc2c([nH]1)ccc(c2)C1=NNC(=O)CC1C
O=CNc1coc2c(c1=O)cc(c(c2)NS(=O)(=O)C)Oc1ccccc1
OC1CCN(CC1)c1c(F)cc2c3c1CCC(n3cc(c2=O)C(=O)O)C
COC(=O)C1(CCN(CC1)CCC(=O)OC)N(c1ccccc1)C(=O)CC
COc1cc(CNCC(COc2ccc3c(c2)ccc(=O)[nH]3)O)ccc1OC
CN1C(=O)CN=C(c2c1ccc(c2)[N+](=O)[O-])c1ccccc1F
[O-]C(=O)C1=C(COC(=O)C)CSC2N1C(=O)C2NC(=O)CC#N
COC(C1=C(N2CC2)C(=O)C(=C(C1=O)N1CC1)C)COC(=O)N
CC(=O)Oc1ccc(cc1)C(=C1CCCCC1)c1ccc(cc1)OC(=O)C
CN(CC(=O)c1ccc(cc1)Cl)CCCN1c2ccccc2CCc2c1cccc2
CN1CCN(CC1)C(=O)OC1c2nccnc2C(=O)N1c1ccc(cn1)Cl
CC(=O)OC1CCC2C1(C)CCC1C2CCC2C1(C)C(=CC(=O)C2)C
CCCC[N+]1(C)C2CC(CC1C1C2O1)OC(=O)C(c1ccccc1)CO
CC(CCCC(C1CCC2C1(C)CCC1C2CCC2C1(C)CCC(C2)O)C)C
CCC(=O)OC1CCC2C1(C)CCC1C2CCC2C1(C)CC(C)C(=O)C2
COC1(CCCC1)OC1CCC2C1(C)CCC1C2CCC2C1(C)CC1SC1C2
CCC(=O)Oc1ccc2c(c1)CCC1C2CCC2(C1CCC2OC(=O)CC)C
OC(=O)c1cc(N2CCCC2)c(c(c1)S(=O)(=O)N)Oc1ccccc1
COc1cc(N)c(cc1C(=O)NC1CC[NH+](CC1)Cc1ccccc1)Cl
Clc1sc2c(c1)S(=O)(=O)N(C(=C2O)C(=O)Nc1ccccn1)C
Clc1ccc(cc1S(=O)(=O)N)S(=O)(=O)N(CC1(C)CCCO1)C
CCCC(=O)N1CCCN(CC1)c1nc(N)c2c(n1)cc(c(c2)OC)OC
COC(=O)CCC=C=CCC1C(=O)CC(C1C=CC(COc1ccccc1)O)O
CC(N(C)C)CN1c2cc(ccc2Sc2c1cccc2)S(=O)(=O)N(C)C
CCN(CC#CC(OC(=O)C(c1ccccc1)(C1CCCCC1)O)(C)C)CC
OCC(c1ccccc1)C(=O)OC1CC2C3C(C(C1)[N+]2(C)CC)O3
SC(C(=O)NC1CCSC2N(C1=O)C(CCC2)C(=O)O)Cc1ccccc1
CN1CCCC1Cc1c[nH]c2c1cc(cc2)CCS(=O)(=O)c1ccccc1
COc1cc2ncnc(c2cc1OCCCN1CCOCC1)Nc1ccc(c(c1)Cl)F

Continued on next page

O=C1N(CCCCN2CCN(CC2)c2ncccn2)C(=O)C2C1C1CCC2C1
CCCCC(C(C=CC1C(O)CC(=O)C1CCCCC=CC(=O)OC)O)(C)C
OCCN1CCN(CC1)CCCN1c2ccccc2Sc2c1cc(cc2)C(F)(F)F
OC(c1ccc(c(c1)O)O)CNCCCCCCNCC(c1ccc(c(c1)O)O)O
CCN1CCN(CC1)c1cc2c(cc1F)c(=O)c(cn2C1CC1)C(=O)O
Fc1ccc(cc1)C(=O)CCCN1CCC(CC1)C(=O)c1ccc(cc1)Cl
CC(=O)N(c1ccc(cc1)Nc1cc(C)nc2c1c1[nH]cnc1cc2)C
O=C(C12CC3CC(C2)CC(C1)C3)NCCN1CCN(CC1)c1ncccn1
CCCN(C(=O)Cc1c(nc2n1cc(Cl)cc2)c1ccc(cc1)Cl)CCC
OC(=O)CCSC(c1ccccc1CCCCCCCCc1ccccc1)C(C(=O)O)O
O=S(=O)(C(F)(F)F)Nc1cccc(c1)OCc1ccc2c(n1)cccc2
OCCN(CCn1c(Cc2ccccc2)nc2c1c(=O)n(C)c(=O)n2C)CC
CCN(CC(O)(C)C)CCNc1ccc(c2c1c(=O)c1c(s2)cccc1)C
CC[N+]1(CC)CCC(C1)OC(=O)C(c1ccccc1)(c1ccccc1)O
CCn1cc(C(=O)O)c(=O)c2c1cc(c(c2)F)N1CCN2CCC1CC2
COCC(=O)N(c1ccccc1F)C1CCN(CC1C)CCn1nnn(c1=O)CC
CCCC1(CCC1)C(CC=CC1C(O)CC(=O)C1CCCCCCC(=O)OC)O
ClC(=C(c1cc(N)c(cc1S(=O)(=O)N)S(=O)(=O)N)Cl)Cl
CN1CC2CC1CN2c1cc2c(cc1F)c(=O)c(cn2C1CC1)C(=O)O
CC(N(C(C)C)CCC(c1ccccc1Cl)(C(=O)N)CCN1CCCCC1)C
CCC(=C(c1cccc(c1)O)c1ccc(cc1)OCCN(C)C)c1ccccc1
NCCC12CCC3(CC1=CCC1C2CCC2(C1CCC12OCCO1)C)OCCO3
O=C1[N-]C(=O)C(S1)Cc1ccc2c(c1)CCC(O2)Cc1ccccc1
O=C(OC1CCC2C1(C)CCC1C2CCc2c1ccc(c2)O)CCC1CCCC1
CCOS(=O)(=O)c1cc(cc2c1ccc(c2)C(C)(C)C)C(C)(C)C
OC(=O)c1cc(ccc1O)n1c2CCc3c(c2cc1c1ccccc1)cccc3
CCCCc1nc(nn1Cc1ccc(nc1)c1ccccc1c1nnn[nH]1)CCCC
Clc1cc2[nH]c(=O)c(=O)[nH]c2c(c1Cl)[N+](=O)[O-]
CN1CCC(CC1)OC(=O)C(Oc1ccc(cc1)Cl)Oc1ccc(cc1)Cl
CC(NCC(c1ccc(c(c1)C(=O)N)O)O)CCc1ccc2c(c1)OCO2
CCCn1c2nc([nH]c2c(=O)n(c1=O)CCC)C1CC2CC1C1C2O1
OCCN(c1nc(nc2c1cccc2)c1ccc(o1)[N+](=O)[O-])CCO
COc1c(OC)ccnc1CS(=O)c1nc2c([nH]1)ccc(c2)OC(F)F
ClC(=CC1C(C1(C)C)C(=O)OCc1cccc(c1)Oc1ccccc1)Cl
O=C(N1CCN(CC1)C(=O)CCOS(=O)(=O)C)CCOS(=O)(=O)C
CCC1CCNC(C1)C(=O)NC(C1OC(SC)C(C(C1O)O)O)C(Cl)C
CC(=O)Oc1sc2c(c1)CN(CC2)C(c1ccccc1F)C(=O)C1CC1
O=C1C=CC2(C(=C1)CCC1C2CCC2(C1CCC2OC1=CCCC1)C)C
COc1ccccc1OCC(CN1CCN(CC1)CC(=O)Nc1c(C)cccc1C)O
CC(NCCN(S(=O)(=O)c1ccc(cc1)NS(=O)(=O)C)C(C)C)C
NS(=O)(=O)c1ccc(cc1)N1CCN(CC1)CCC1OCCc2c1cccc2
CSCS(=O)CC(NC(=O)C=Cc1c(C)[nH]c(=O)[nH]c1=O)CO
CC(=O)OC1CCC2C1(C)CCC1C2CCC2C1(C)C=C(C)C(=O)C2
CCC(c1ccccc1)NC(=O)c1c(O)c(nc2c1cccc2)c1ccccc1
CCOC(=O)C(c1ccccc1)C(=O)OC1CC2CCC(C1)[N+]2(C)C
O=C(NC1C(=O)N2C1SC(C2C(=O)[O-])(C)C)CSc1ccccc1
OC(=O)C=Cc1nc(ccc1OCCc1ccccc1)CSc1c(Cl)cccc1Cl
OC1C(O)C(OC1n1cc2c3c1ncnc3n(nc2N)C)COP(=O)(O)O
CN(CCOc1ccccc1c1nc(c([nH]1)c1ccccc1)c1ccccc1)C
OC(c1ccc(c(c1)NS(=O)(=O)C)O)CNC(Cc1ccccc1)(C)C
O=C(N(C1CCC2(CC1N1CCCC1)CCCO2)C)Cc1cccc2c1cco2
CCC(=C(c1ccc(cc1)O)c1ccc(cc1)OCCN(C)C)c1ccccc1
COC(=O)C1C(O)CCC2C1CC1N(C2)CCc2c1[nH]c1c2cccc1
CC[N+]1(C)CCCC(C1)OC(=O)C(c1ccccc1)(c1ccccc1)O
O=C1CCC2C(=C1)C(=CC1C2CCC2(C1CCC2(O)C(=O)C)C)C
CC(C(C(=O)N1CCCC1)(c1ccccc1)c1ccccc1)CN1CCOCC1
OC(=O)CCNC(=O)c1ccc(cc1)N=Nc1ccc(c(c1)C(=O)O)O
CON=C(c1csc(n1)N)C(=O)NC1C2SCC=C(N2C1=O)C(=O)O
OC(=O)COc1cc(OCC(=O)O)c2c(c1)oc(cc2=O)c1ccccc1
NCCCCC(C(=O)N1CCCC1C(=O)O)NC(C(=O)O)CCc1ccccc1
OC(COc1cccc(c1)Cl)CSC1C(O)CC(C1CC=CCCCC(=O)O)O
CN1CCN(CC1)c1c(F)cc2c3c1OCN(n3cc(c2=O)C(=O)O)C
Clc1cc(NC(=O)c2c(O)c(Cl)cc(c2Cl)Cl)c(c(c1)Cl)O
CC(C(=O)NC1C(=O)N2C1SC(C2C(=O)O)(C)C)Oc1ccccc1
C=CC=CCC1=C(C)C(CC1=O)OC(=O)C1C(C1(C)C)C=C(C)C
CCCCCC(CCC1C(O)CC2C1Cc1cccc(c1C2)OCC(=O)[O-])O
FCC1CC(=O)N(C1)C1CN2CCc3c(C2CC1N)cc(c(c3)OC)OC
CCOC(=O)COc1ccc2c(c1)CC(CC2)NCC(c1cccc(c1)Cl)O
CCOC(=O)COc1ccc2c(c1)CC(CC2)NCC(c1cccc(c1)Cl)O
O=C(c1ccc(c(c1)S(=O)(=O)N)Cl)NN1Cc2c(C1C)cccc2
COC(C(=O)O)Cc1ccc(cc1)OCCCOc1ccc(cc1)Oc1ccccc1
C1CNCCN(CCCNCCNC1)Cc1ccc(cc1)CN1CCNCCCNCCNCCC1
OCC1OC(C(C1O)O)n1cnc2c1nc(OCCc1ccc(cc1)Cl)nc2N
CC(=O)OCCN1CCN(CC1)CCCN1c2ccccc2Sc2c1cc(Cl)cc2
N#CC(c1ccccc1)(c1ccccc1)CC1CC2CCC(C1)[N+]2(C)C
CCOC(=O)COc1cc(C)c(cc1C)CCNC(C(c1ccc(cc1)O)O)C
CCN(CCCCCC(F)(C)C)CCCC(c1ccc(cc1)NS(=O)(=O)C)O
CSCCC(C(=O)NC1(CS(=O)(=O)C2C1C2C(=O)O)C(=O)O)N
OCC1OC2(OCc3c2cc(cc3)Cc2ccc(cc2)CC)C(C(C1O)O)O
COCCn1c(C)[n+](c2c1C(=O)c1ccccc1C2=O)Cc1cnccn1
ONC(=O)c1cnc(nc1)N1CCC(CC1)CNCc1cn(c2c1cccc2)C

Continued on next page

Table S8 – *Continued from previous page*

CN1Cc2cc(ccc2C(C1)c1ccc2c(c1)cccc2)c1ccc(nn1)N
OCC(=O)C1CCC2C1(C=O)CC(O)C1C2CCC2=CC(=O)CCC12C
NC(=O)c1cnc2c(c1NC1C3CC4CC1CC(C3)(C4)O)cc[nH]2
OC(=O)CN1CCN(CC1)C(c1cccc(c1)C(F)(F)F)c1ccccc1
O=C1CCC2C(=C1)CCC1C2CCC2(C1CC(=C)C2(O)C(=O)C)C
O=C(C=Cc1cccnc1)NCc1ccc(cc1)C(=O)Nc1ccc(cc1N)F
CC(CCCC(C1CCC2C1(C)CCC1C2CC=C2C1(C)CCC(C2)O)C)C
OCC(=O)C1(O)CCC2C1(C)CC(O)C1C2CCC2=CC(=O)CCC12C
OC(=O)CC1=C(C)C(=Cc2ccc(cc2)S(=O)C)c2c1cc(F)cc2
COC12C3NC3CN2C2=C(C1COC(=O)N)C(=O)C(=C(C2=O)C)N
O=C(C(c1ccc(cc1)O)N)NC1C(=O)N2C1SCC(=C2C(=O)O)C
CCOC(=O)C1=C(C)NC(=C(C1c1cccc(c1Cl)Cl)C(=O)OC)C
OC(CCc1ccccc1)CCC1C(O)CC(C1CC=CCCCC(=O)OC(C)C)O
O=C(C1c2ccccc2Oc2c1cccc2)OCC[N+](C(C)C)(C(C)C)C
COc1ccc(cc1)C1Sc2ccccc2N(C(=O)C1OC(=O)C)CCN(C)C
CCn1cc(C(=O)O)c(=O)c2c1c(F)c(N1CCNC(C1)C)c(c2)F
CNCC(c1ccc(c(c1)OC(=O)C(C)(C)C)OC(=O)C(C)(C)C)O
CCCCS(=O)(=O)NC(C(=O)O)Cc1ccc(cc1)OCCCCC1CCNCC1
OCCN1CCN(CC1)CCC=C1c2ccccc2Sc2c1cc(cc2)C(F)(F)F
Fc1ccc(cc1)C(=O)CCCN1CCC2(CC1)C(=O)NCN2c1ccccc1
OC(=O)CCCCCC(C1=C(C)C(=O)C(=C(C1=O)C)C)c1ccccc1
OCC(NC(=O)C1CN(C)C2C(=C1)c1cccc3c1c(C2)c[nH]3)C
CCOC(C(=O)O)Cc1ccc(cc1)OCCc1ccc(cc1)OS(=O)(=O)C
CCN(C(=O)NC1CN(C)C2C(C1)c1cccc3c1c(C2)c[nH]3)CC
CCC(=O)OC1CCC2C(=C1)CCC1C2CCC2(C1CCC2OC(=O)CC)C
CCC(c1ccccc1)(N(C)C)COC(=O)c1cc(OC)c(c(c1)OC)OC
FC(c1cccc(c1)C1=CCN(CC1)CCc1ccc2c(c1)cccc2)(F)F
COc1cccc(c1)CCc1ccccc1OCC(OC(=O)CCC(=O)O)CN(C)C
COC(=O)C1(O)CC2(CC)CCCN3C2c2n1c1cc(Br)ccc1c2CC3
CCN(C(=O)Nc1ccc(c(c1)C(=O)C)OCC(CNC(C)(C)C)O)CC
CCCCC(CC(C=CC1C(O)CC(=O)C1CC(=O)CCCCC(=O)OC)O)C
CNCC(c1ccc(c(c1)OC(=O)C(C)(C)C)OC(=O)C(C)(C)C)O
O=C(c1ccccc1C(=O)O)Nc1ccc(cc1)S(=O)(=O)Nc1nccs1
NC(=N)NCC1CCC(CC1)C(=O)Oc1ccccc1C(=O)OCc1ccccc1
OC(=O)CCC(=O)OC1OC2OC3(C)CCC4C2(C(C1C)CCC4C)OO3
OCC1COc2c(O1)cccc2N1CCN(CC1)CCNC(=O)c1ccc(cc1)F
Clc1ccc2c(c1)N(CCCN1CCC(CC1)C(=O)N)c1c(S2)cccc1
OC1(CCN2C(C1)c1cccc3c1C(C2)c1ccccc1CC3)C(C)(C)C
COc1cc2c(cc1OC)[nH]c(c2CCN1CCN(CC1)c1ccccc1OC)C
CC(N(C(C)C)CCC1(C(=O)N=C(N2C1CCCC2)C)c1ccccc1)C
COc1ccc(cc1F)[N-]S(=O)(=O)c1c(F)c(F)c(c(c1F)F)F
CC(N(C(=O)C)CCC(c1ccccc1Cl)(C(=O)N)CCN1CCCCC1)C
CCCCN(CCCC)CCCOc1ccc(cc1)C(=O)c1c(CC)cc2n1cccc2
CCOC(=O)SC(=C(N(Cc1cnc(nc1N)C)C=O)C)CCOC(=O)OCC
COCCOCC(C(=O)O)CC1(CCCC1)C(=O)NC1CCC(CC1)C(=O)O
O=C1CCC2(C(=C1)C=CC1C2CCC2(C1CCC12CCC(=O)O1)C)C
CCC(=O)N(C1(CCN(CC1)CCc1ccccc1)C(=O)OC)c1ccccc1
NCCCCC(C(=O)N1CCCC1C(=O)O)OP(=O)(CCCCc1ccccc1)O
CCOC(=O)CN1CCN(CC1)C(=O)C=Cc1cc(OC)c(c(c1)OC)OC
N#CC(c1ccc(cc1Cl)n1ncc(=O)[nH]c1=O)c1ccc(cc1)Cl
CCOC(=O)C1=C(C)NC(=C(C1c1cccc2c1non2)C(=O)OCC)C
COc1ccc(cc1F)c1cc(nn1c1ccc(cc1)S(=O)(=O)N)C(F)F
CC(=O)OCC(=O)C1CCC2C1(C)CCC1C2CCC2=CC(=O)CCC12C
OC(=O)C(CC(=O)c1ccc(cc1)c1ccc(cc1)Cl)CSc1ccccc1
OCc1ccc(cc1)C=C(c1ccc2c(c1)C(C)(C)C(CC2(C)C)O)C
OCC(=O)N1CCN(CC1)c1ccc(cc1F)N1CC(OC1=O)CNC(=O)C
NC(C1=CCC=CC1)C(=O)NC1C(=O)N2C1SC(C2C(=O)O)(C)C
Clc1cc2NC(CSCC(F)(F)F)NS(=O)(=O)c2cc1S(=O)(=O)N
CCCCCCCCCCC(=O)OC1CCC2C1(C)CCC1C2CCc2c1ccc(c2)O
COc1cc(C=Cc2nc3c(n2C)c(=O)n(c(=O)n3CC)CC)ccc1OC
Oc1ccc2c(c1)CCC(C2c1ccc(cc1)OCCN1CCCC1)c1ccccc1
O=C1N(c2ccccc2)c2c(C1(Cc1ccncc1)Cc1ccncc1)cccc2
O=C1NC2=Nc3c(CN2C1)cc(cc3)OCCCC(=O)N(C1CCCCC1)C
COc1ccc(cc1)CCNC(C(c1ccc(c(c1)NS(=O)(=O)C)O)O)C
CN1CC(CC2C1Cc1cn(c3c1c2ccc3)C)NC(=O)n1nc(cc1C)C
NC1=NC(NC1(C(F)(F)F)C(F)(F)F)(C(F)(F)F)C(F)(F)F
ON=C1CCC2C(=C1)CCC1C2CCC2(C1CCC2(C#C)OC(=O)C)CC
C#CC1(O)C(O)CC2C1(C)CCC1C2CCc2c1ccc(c2)OC1CCCC1
CON=C(c1csc(n1)N)C(=O)NC1C(C)N(C1=O)OCC(=O)[O-]
CCCCCNC12C=CC(=O)C3C42CCN(C1Cc1c4c(O3)c(cc1)O)C
CCCCCC(C=CC1C(O)Cc2c1cc(n2c1ccccc1)CCCCC(=O)O)O
CCOC(=O)c1nc(=O)c2c([nH]1)nc1c(c2)cc(c(c1)OC)OC
O=C(CCC1(C)C2Cc3c(C1(C)CCN2C)cc(cc3)O)CCC1CCCC1
OCC1OC(C(C1O)O)n1cnc2c1nc(nc2N)n1ncc(c1)C(=O)NC
OC(CN1CCC(CC1)N(c1nc2c(s1)cccc2)C)COc1ccc(cc1)F
Clc1cccc(c1)C(CNCCNc1cccc(c1)c1cccc(c1)C(=O)O)O
O=c1cc(C)c2c(o1)cc(c(c2)OS(=O)(=O)O)OS(=O)(=O)O
O=C1CCC2=C3CCC4(C(C3CCC2=C1)CCC4(C)C(=O)C(O)C)C
N#CC1CC(CN1C(=O)C(C(c1ccc(cc1)F)c1ccc(cc1)F)N)F
N#Cc1c[nH]c2c1cccc2C(=O)N1CCN(CC1)CCc1ccc(cc1)F
COC(C(C(=O)O)Oc1nc(C)cc(n1)C)(c1ccccc1)c1ccccc1

Continued on next page

Table S8 – *Continued from previous page*

OC(c1ccc(c(c1)O)O)CNCCn1cnc2c1c(=O)n(C)c(=O)n2C
C=NC(c1ccccc1)C(=O)NC1C(=O)N2C1SC(C2C(=O)O)(C)C
ONC(=O)Cc1c(C)n(c2c1cc(OC)cc2)C(=O)c1ccc(cc1)Cl
O=c1oc2c(c(=O)n1c1ccccn1)N(C)S(=O)(=O)c1c2cccc1
O=C(C1C(C1(C)C)C=C(C)C)OCN1C(=O)C2=C(C1=O)CCCC2
Clc1cc(C(C)C)c(c(c1C)Cc1c(C)c(Cl)cc(c1O)C(C)C)O
OC(=O)CCCC=CCC1C(O)CC(C1C=CC(COc1cccc(c1)Cl)O)O
Fc1ccc(cc1)C(c1ccc(cc1)F)N1CCN(CC1)CC=Cc1ccccc1
CC(CCCC(OC(=O)Cn1cnc2c1c(=O)n(C)c(=O)n2C)(C)C)N
CCc1c(C(=O)C(=O)N)c2c(n1Cc1ccccc1)cccc2OCC(=O)O
Cc1cc(OS(=O)(=O)[O-])c2c(c1OS(=O)(=O)[O-])cccc2
CCOc1cc(N)c(cc1C(=O)NCC1OCCN(C1)Cc1ccc(cc1)F)Cl
NC(=N)Nc1ccc(cc1)C(=O)Oc1ccc2c(c1)ccc(c2)C(=N)N
CCC(C(=O)NC1C(=O)N2C1SC(C2C(=O)O)(C)C)Oc1ccccc1
COC(=O)C1=COC(C2C1CC1N(C2)CCc2c1[nH]c1c2cccc1)C
CCC(=O)C1(O)CCC2C1(C)CC(O)C1C2CCC2=CC(=O)CCC12C
SCC(=O)C1(O)CCC2C1(C)CC(O)C1C2CCC2=CC(=O)CCC12C
CC(=O)OCCN1CCN(CC1)CCC=C1c2ccccc2Sc2c1cc(Cl)cc2
OCC#CCOc1ccc(cc1)S(=O)(=O)N1CCSC(C1C(=O)NO)(C)C
CN(C(=S)c1ccccc1)NC(=O)CC(=O)NN(C(=S)c1ccccc1)C
CCN(C(=O)c1c([O-])c2c(Cl)cccc2n(c1=O)C)c1ccccc1
O=CNc1cc(ccc1O)C(CNCCc1ccc(cc1)NCC(c1ccccc1)O)O
COC1=CC(=NC1=Cc1[nH]c(cc1C)C)c1cc2c([nH]1)cccc2
N#CC1CC2C(N1C(=O)C(C13CC4CC(C1)CC(C3)(C4)O)N)C2
O=C(C(NC(=O)C(C(C)C)O)C)NC1C(=O)N(C)CCc2c1cccc2
CNc1ncc(cc1C)c1ccc2c(c1C)n(cc(c2=O)C(=O)O)C1CC1
COC(=O)N1CCN(CC1)Cc1cccc(c1F)NC(=O)Nc1ccc(nc1)C
N#CC=Cc1cc(C)c(c(c1)C)Nc1ccnc(n1)Nc1ccc(cc1)C#N
N#Cc1cc(NC(=O)COC(=O)C)c(c(c1)NC(=O)COC(=O)C)Cl
OCC(C(=O)Nc1cc(C=Cc2cc(OC)c(c(c2)OC)OC)ccc1OC)N
O=C(N(C)C)NC1CCC(CC1)CCN1CCN(CC1)c1cccc(c1Cl)Cl
N#CC1=C(N)Oc2c(C1c1cc(Br)c(c(c1)OC)OC)ccc(c2N)N
Clc1ccc(cc1)Cn1cc(c2c1cccc2)C(=O)C(=O)Nc1ccncc1
COc1ccc(cc1)CC(NCC(c1cc(O)cc2c1OCC(=O)N2)O)(C)C
ONC(=O)c1ccc(cc1)OCCNC(=O)c1oc2c(c1CN(C)C)cccc2
OC(=O)C1NCC2C(C1)CC(CC2)Oc1cccc(c1c1nnn[nH]1)Cl
COc1ccc(c2c1nc(s2)NC(=O)N1CCC(CC1)(C)O)N1CCOCC1
COC(=O)CCC1N=C(c2ccccn2)c2c(n3c1ncc3C)ccc(c2)Br
CN1CCC(CC1)C(=O)c1cccc(n1)NC(=O)c1c(F)cc(cc1F)F
Cc1cccc(n1)c1nn2c(c1c1ccnc3c1cc(cc3)C(=O)N)CCC2
O=C1CC2OCC=C3C4C2C2N1c1ccccc1C12CC[NH+](C1C4)C3
Fc1ccc2c(c1)[nH]cc2CCNCc1cccc(c1)OCC(C(F)F)(F)F
O=C(NC(C(=O)O)Cc1c[nH]c2c1cccc2)CCC(C(=O)[O-])N
OCCNC1CCc2c1cccc2c1noc(n1)c1ccc(c(c1)C#N)OC(C)C
CCC1CN(CC1c1cnc2n1c1cc[nH]c1nc2)C(=O)NCC(F)(F)F
CCCCOc1nc2N(CC(=O)Nc2c(n1)N)Cc1cccc(c1)CN1CCCC1
CCCC1CN(C(C1)C(=O)NC(C1OC(SC)C(C(C1O)O)O)C(O)C)C
O=C(C(c1ccc(cc1)O)N)NC1C(=O)N2C1SC(C2C(=O)O)(C)C
Clc1ccc(cc1)C1CCC(CC1)C1=C(O)C(=O)c2c(C1=O)cccc2
CCCCc1nc(c(n1Cc1ccc(cc1)c1ccccc1c1[n-]nnn1)CO)Cl
OC(=O)c1cc(N=Nc2ccc(cc2)S(=O)(=O)Nc2ccccn2)ccc1O
O=C1N(c2ccccc2)N(C(=O)C1CCS(=O)c1ccccc1)c1ccccc1
OCC(=O)C1(O)CCC2C1(C)CC(O)C1C2CCC2=CC(=O)C=CC12C
Cc1ccc(cc1)c1cc(nn1c1ccc(cc1)S(=O)(=O)N)C(F)(F)F
Clc1cc2N=C(CSCc3ccccc3)NS(=O)(=O)c2cc1S(=O)(=O)N
CCC(NC(=O)C1CN(C)C2C(=C1)c1cccc3c1c(C2)c[nH]3)CO
CCN(C(=O)C(=Cc1cc(O)c(c(c1)[N+](=O)[O-])O)C#N)CC
COCC1(CCN(CC1)CCn1nnn(c1=O)CC)N(c1ccccc1)C(=O)CC
CCCCCCCCCC(=O)OC1CCC2C1(C)CCC1C2CCC2=CC(=O)CCC12
CC([N+](C(C)C)(CCC(c1ccccc1)(c1ccccc1)C(=O)N)C)C
N#CC1=C(O)C2C3(C(C1)(C)C1CCC4(C(C1CC3)CCC4O)C)O2
CCC1C(=NN=C(c2c1cc(OC)c(c2)OC)c1ccc(c(c1)OC)OC)C
Clc1cc2NC(NS(=O)(=O)c2cc1S(=O)(=O)N)C1CC2CC1C=C2
O=C(c1ccc(cc1)C(=O)O)Nc1ccc2c(c1)C(C)(C)CCC2(C)C
CCN(C(=O)NC1CN(C)C2C(=C1)c1cccc3c1c(C2)c[nH]3)CC
CC(CCCC(C1CCC2C1(C)CCCC2=CC=C1CC(O)CC(C1=C)O)C)C
FCCn1cc(C(=O)O)c(=O)c2c1c(F)c(N1CCN(CC1)C)c(c2)F
O=C(OC1CCC2C1(C)CCC1C2CCC2=CC(=O)CCC12)CCc1ccco1
O=C(c1ccccc1)OC(COc1cccc2c1cc([nH]2)C)CNC(C)(C)C
O=C(C(c1ccc2c(c1)Cc1c(O2)nc(cc1)C)C)OCC(=O)N(C)C
O=C(c1ccc(c(c1)S(=O)(=O)N)Cl)NN1CC2C(C1)C1CC2CC1
CC(=O)SCC(C(=O)N1CCCC1C(=O)NC(C(=O)O)Cc1ccccc1)C
O=C1Nc2c(C1)cc(c(c2)Cl)CCN1CCN(CC1)c1nsc2c1cccc2
NC(c1ccccc1)C(=O)NC1C(=O)N2C1SC(C2C(=O)[O-])(C)C
CC1NCCN(C1)c1cc2c(c(c1F)C)c(=O)c(cn2C1CC1)C(=O)O
O=C1CCC2(C(=C1)C(C)CC1C2C(O)CC2(C1CCC2C(=O)C)C)C
NC1CCN(C1)c1c(F)cc2c(c1Cl)n(cc(c2=O)C(=O)O)C1CC1
Clc1ccc(c(c1)Cl)C(Cn1cncc1)OCc1ccc(cc1)Sc1ccccc1
CCCC(=O)c1ccc2c(c1)N(CCCN1CCN(CC1)C)c1c(S2)cccc1
O=C1CCC(C(=O)N1)(C1CCN(CC1)CC=Cc1ccccc1)c1ccccc1
COc1cc(ccc1OCCCN1CCC(CC1)c1noc2c1ccc(c2)F)C(=O)C

Continued on next page

CC(Oc1ccccc1N1CCN(CC1)Cc1cccc(c1)C(=O)N1CCCCC1)C
Fc1ccc(cc1)C(=O)C1CCN(CC1)CCc1c(C)nc2n(c1=O)CCS2
CC#CCC(C(C=CC1C(O)CC2C1c1cccc(c1O2)CCCC(=O)O)O)C
CCCc1cc2c(s1)CNC1C2c2cc(OC(=O)C)c(cc2CC1)OC(=O)C
CN1CCC(C(C1)O)c1c(O)cc(c2c1oc(cc2=O)c1ccccc1Cl)O
C=CCNS(=O)(=O)c1cc(c(cc1N=CC=C(O)C)Cl)S(=O)(=O)N
CC(=O)OC1(CCC2C1(C)CCC1C2CC(C2=CCCCC12C)C)C(=O)C
CCNc1cccnc1N1CCN(CC1)C(=O)c1cc2c([nH]1)ccc(c2)OC
OC(=O)CN1C(=O)C(CCc2c1cccc2)NC(C(=O)O)CCc1ccccc1
Cc1cc2c(cc1C(=C)c1ccc(cc1)C(=O)O)C(C)(C)CCC2(C)C
CN1C2CCC(C1CC(C2)OC(=O)C(c1cccs1)(c1cccs1)O)(C)C
O=C(C1N2C(=O)CC2S(=O)(=O)C1(C)C)OCOC(=O)C(C)(C)C
CCC(=O)[N-]S(=O)(=O)c1ccc(cc1)c1c(C)onc1c1ccccc1
O=CC(NC(=O)C1CCCN1C(=O)C(Cc1ccccc1)NC)CCCNC(=N)N
[O-]C(=O)CN(CC(=O)[O-])CCN(CC(=O)[O-])CC(=O)[O-]
O=C(Nc1cc(C)cc(c1)C)Cc1ccc(cc1)OC(C(=O)[O-])(C)C
CCS(=O)(=O)CCN1C(=O)CN=C(c2c1ccc(c2)Cl)c1ccccc1F
CC(CC(c1ccc(cc1)OCCOCCOCCS(=O)(=O)[O-])(C)C)(C)C
CC=C(c1ccc2c(c1)n(c(n2)N)S(=O)(=O)C(C)C)c1ccccc1
ON=C(c1ccc2c(c1)n(c(n2)N)S(=O)(=O)C(C)C)c1ccccc1
ClCCN(C(=O)Oc1ccc2c(c1)CCC1C2CCC2(C1CCC2O)C)CCCl
CC(=O)Oc1ccc2c(c1)CC=C1C2CCC2(C1CCC2(O)c1cocc1)C
COC(=O)Nc1[nH]c2c(n1)cc(cc2)C1(OCCO1)c1ccc(cc1)F
CCCCCCCCCCCCCCCC(=O)OCCOC(=O)CCCCCCCCCCCCCCCC
Clc1c[nH]c2c1cccc2NS(=O)(=O)c1ccc(cc1)S(=O)(=O)N
CCc1cccc2c1nc(s2)Sc1ccc2c(c1)CCC1C2(C)CCC(=O)N1C
CCC1C(=NN=C(c2c1cc(OC)c(c2)OC)c1ccc(c(c1)OC)OC)C
CCC(=O)N(C1(CCN(CC1C)CCc1ccccc1)C(=O)OC)c1ccccc1
C=CCn1c(=O)n(c2c1c(=O)[nH]c(n2)N)C1OC(C(C1O)O)CO
Fc1ccc(cc1)c1cc(nn1c1ccc(cc1)S(=O)(=O)N)C(F)(F)F
COc1ccc(cc1)CC(N(CCCCOC(=O)c1ccc(c(c1)OC)OC)CC)C
CCCCCC1CNC(C1)C(=O)NC(C1OC(SC)C(C(C1O)O)O)C(Cl)C
CCCCCCC(c1cc(O)c2c(c1)OC(C1C2CC(=O)CC1)(C)C)(C)C
CC(=CCN1CCC23C4(C1Cc1c3c(OC2C(=O)CC4)c(cc1)O)O)C
OC(=O)CN(CCN(CC(=O)O)CC(=O)O)CCN(CC(=O)O)CC(=O)O
Oc1cc(O)cc(c1)C(CNCCCn1cnc2c1c(=O)n(C)c(=O)n2C)O
COc1ccc2c(c1)n(C)c(n2)COc1ccc(cc1)CC1SC(=O)NC1=O
OC(=O)CN1CCN(CCN(CCN(CC1)CC(=O)O)CC(=O)O)CC(=O)O
CCSC1(SC)CCC2C1(C)CC(O)C1(C2CCC2=CC(=O)C=CC12C)F
CCC(C(C1Nc2cc(Cl)c(cc2S(=O)(=O)N1)S(=O)(=O)N)C)C
CC(=O)OC1C=CC2C34C1Oc1c4c(CC2N(CC3)C)ccc1OC(=O)C
Clc1ccc2c(c1)C(=NC(C(=O)N2C)OC(=O)N(C)C)c1ccccc1
O=C(N(C)C)Oc1cc(cc(c1)OC(=O)N(C)C)C(CNC(C)(C)C)O
CN1CCC23C4C1Cc1c3c(OC2C(C=C4)O)c(cc1)OCCN1CCOCC1
Cc1cncc(c1)CN1CCC(=C2c3ccc(cc3CCc3c2nccc3)Cl)CC1
O=C(NC1C(=O)N2C1SCC(=C2C(=O)O)CSc1nncs1)Cn1cnnn1
COc1nc(C)cnc1NS(=O)(=O)c1cccnc1c1ccc(cc1)c1nnco1
OCC(=O)C1(O)CCC2C1(C)CC(=O)C1C2CCC2=CC(=O)CCC12C
OC(=O)CCCC=CCC1C(O)CC(C1C=CC1(OCCO1)COc1ccccc1)O
CNC1CN(CC1OC)c1ccc2c(n1)n(cc(c2=O)C(=O)O)c1nccs1
CCCCc1nc(c(n1Cc1ccc(cc1)c1ccccc1c1nnn[nH]1)CO)Cl
O=C1CCC2(C(=C1)C(C)CC1C2CCC2(C1CCC2(O)C(=O)C)C)C
COC(=O)COc1cccc2c1c(C(=O)C(=O)N)c(n2Cc1ccccc1)CC
OCCNCCNc1ccc(c2c1C(=O)c1c(C2=O)c(O)ccc1O)NCCNCCO
Oc1ccc(cc1)C(CN(c1nc2c(n1C)c(=O)n(c(=O)n2C)C)C)O
O=C(C(C)(C)C)CCC1C(=O)N(N(C1=O)c1ccccc1)c1ccccc1
O=C(N1CC(CC1C(=O)O)Sc1ccccc1)C(CSC(=O)c1ccccc1)C
COC(C(=O)O)Cc1ccc(c2c1scc2)OCCc1nc(oc1C)c1ccccc1
CCC(CC(C1(O)CCC2C(O1)CC(=O)C2CCCCCCC(=O)O)(F)F)C
OCCC(Nc1ncc2c(n1)n(C)c(=O)c(c2)Oc1ccc(cc1F)F)CCO
COc1cc(ccc1C(=O)O)c1ccc(cc1)CCNCC(c1cccc(c1)Cl)O
COc1ccc(cc1)S(=O)(=O)n1cc(c2c1ccc(c2)OC)CCC(=O)O
CC(COc1cn2c(c1C)c(ncn2)Oc1ccc2c(c1F)cc([nH]2)C)O
O=C(Nc1cc(C)ccc1F)Nc1ccc(cc1)c1cccc2c1c(N)n[nH]2
OCC1OC(=O)N(C1)c1ccc(c(c1)F)c1ccc(nc1)c1nnn(n1)C
CCCCCC(c1ccc(cc1)N1C(CCCc2ccc(s2)C(=O)O)CCC1=O)O
CCCCCC(c1ccc(cc1)N1C(COCc2ccc(s2)C(=O)O)CCC1=O)O
OCC1OC(c2ccc(c(c2)Cc2cc3c(s2)cccc3)F)C(C(C1O)O)O
NCC(NC(=O)c1sc(c(c1)c1c(Cl)cnn1C)Cl)Cc1cccc(c1)F
[O-][N+](=O)OCC1OC(C(C1O)O)n1cnc2c1ncnc2NC1CCCC1
CC#CCC(C(C=CC1C(O)CC2C1c1cccc(c1O2)CCCC(=O)O)O)C
ONC(=O)CCCCCCNC(=O)c1cnc(nc1)N(c1ccccc1)c1ccccc1
Fc1ccc(cc1)C1Nc2cc(F)cc3c2c(C1c1ncnn1C)n[nH]c3=O
NC(Cc1cc(F)c(cc1F)F)CC(=O)N1CCNC(=O)C1COC(C)(C)C
OC1CCC2(C(C1)CC(C1C2CCC2(C1CCC2C(CCC(=O)O)C)C)O)C
O=C1CCC(N1)C(=O)NC(C(=O)N1CCCC1C(=O)N)Cc1[nH]cnc1
CCN(C(=O)C1CN2CCc3c(C2CC1OC(=O)C)cc(c(c3)OC)OC)CC
CCCC1CN(C(C1)C(=O)NC(C1OC(SC)C(C(C1O)O)O)C(Cl)C)C
CCCCC(COC(=O)CC(S(=O)(=O)[O-])C(=O)OCC(CCCC)CC)CC
COC(=O)C1=C(C)NC(=C(C1c1cccc2c1non2)C(=O)OC(C)C)C
CCOC(=O)C(NC(C(=O)N1C2CCCC2CC1C(=O)O)C)CCc1ccccc1

Continued on next page

Table S8 – *Continued from previous page*

Fc1ccc2c(c1)onc2C1CCN(CC1)CCc1c(C)nc2n(c1=O)CCCC2
OCC(=O)C1(O)CCC2C1(C)CC(=O)C1C2CCC2=CC(=O)C=CC12C
OC(=O)c1cc2ccccc2c(c1O)Cc1c(O)c(cc2c1cccc2)C(=O)O
COc1c(OC)cc2c(c1OC)c1ccc(c(=O)cc1C(CC2)NC(=O)C)OC
NCCOCC1=C(C(=O)OCC)C(C(=C(N1)C)C(=O)OC)c1ccccc1Cl
[O-]C(=O)C1=CCSC2N1C(=O)C2NC(=O)C(=NOC)c1csc(n1)N
O=C(OC1CCC2C1(C)CCC1C2CCC2=CC(=O)CCC12)CCc1ccccc1
O=C(OC1CCC2C1(C)CCC1C2CCC2=CC(=O)CCC12C)CCC1CCCC1
CCOC(=O)C(NC1CCCN2N(C1=O)C(CCC2)C(=O)O)CCc1ccccc1
CCOC1OC(C(C1O)OCc1ccccc1)C(OCc1ccccc1)COCc1ccccc1
Clc1ccc(cc1)C(=O)NC(C(=O)O)Cc1cc(=O)[nH]c2c1cccc2
O=C1CCc2c(N1)cc(cc2)OCCCCN1CCN(CC1)c1cccc(c1Cl)Cl
CC(C(=O)NC1C(=O)N2C1SC(C2C(=O)[O-])(C)C)Oc1ccccc1
CNCc1ccc(o1)CSCCNC(=NS(=O)(=O)C)NCC(c1ccc(cc1)O)O
Cc1ncc(c(n1)N)C[n+]1csc(c1C)CCOP(=O)(OP(=O)(O)O)O
CCCCCC1Nc2cc(c(cc2S(=O)(=O)N1)S(=O)(=O)N)C(F)(F)F
OC1CCC2(C(C1)CCC1C2CCC2(C31OC3CC2c1ccc(=O)oc1)C)C
O=C(OC1CCC2C1(C)CCC1C2CCC2=CC(=O)CCC12)CCC1CCCCC1
N#CC(c1cccc(c1)Oc1ccccc1)OC(=O)C1C(C1(C)C)C=C(C)C
COC1=C(C(=O)O)N2C(SC1)C(C2=O)NC(=O)C(C1=CCC=CC1)N
COc1ccc(cc1OC)C(=O)N1CCN(CC1)c1ccc2c(c1)CCC(=O)N2
[O-]C(=O)CCC1(O)CCC2C1(C)CCC1C2C=CC2=CC(=O)CCC12C
COc1cc(C=CC(=O)N2CCN(CC2)CC(=O)N2CCCC2)cc(c1OC)OC
[O-]c1ccc2c(c1)oc1c(c2c2ccccc2C(=O)[O-])ccc(=O)c1
Fc1ccc(cc1)C(=O)CCCN1CCC(CC1)n1c(=S)[nH]c2c1cccc2
CCCCc1ncc(n1Cc1ccc(cc1)C(=O)O)C=C(C(=O)O)Cc1cccs1
O=C1CN(C(=O)N1)N=Cc1ccc(o1)c1ccc(cc1)[N+](=O)[O-]
NCC1CN(CC1=NOC)c1nc2c(cc1F)c(=O)c(cn2C1CC1)C(=O)O
O=C(C(c1cscc1)C(=O)O)NC1C(=O)N2C1SC(C2C(=O)O)(C)C
OC(=O)CCCCCCNC1c2ccccc2N(S(=O)(=O)c2c1ccc(c2)Cl)C
Fc1ccc(cc1)C(=O)CCCN1CCC(CC1)n1c(=O)[nH]c2c1cccc2
O=C1CC2(CCCC2)CC(=O)N1CCCCN1CCN(CC1)c1nsc2c1cccc2
O=C(Nc1ccc(cc1C(=O)c1ccccc1)Cl)CN(CC(=O)O)CC(=O)O
CCOC(C(=O)N(CCN(CCc1ccccc1)C)C)(c1ccccc1)c1ccccc1
CON=C(c1csc(n1)N)C(=O)NC1C2SCC(=C(N2C1=O)C(=O)O)C
O=C1CCC2(C(=C1)C(Cl)CC1C2CCC2(C1CC(C2C(=O)C)C)C)C
Clc1ccc2c(c1)C(=[N+]([O-])CC(=N2)NCC1CC1)c1ccccc1
Fc1ccc(cc1)C(=O)C1CCN(CC1)CCCn1c(=O)[nH]c2c1cccc2
O=C(N1CC(CC1C(=O)O)C1CCCCC1)CP(=O)(CCCCc1ccccc1)O
OC(=O)C(NC(C(=O)N1Cc2ccccc2CC1C(=O)O)C)CCc1ccccc1
ONC(=O)C1N(CCSC1(C)C)S(=O)(=O)c1ccc(cc1)Oc1ccncc1
Brc1ccc(c(c1)F)Cn1c(=O)c2ccc(cc2n(c1=O)CC(=O)O)Cl
CN1CCC2(C1N(C)c1c2cc(cc1)OC(=O)N1CCc2c(C1)cccc2)C
OC(=O)CC(c1cccnc1)NC(=O)C1CCCN(C1)C(=O)CCC1CCNCC1
CN1CCN(CC1)c1ccccc1C=C1SCCN(C1=O)c1ccc(c(c1)Cl)Cl
O=C1C=CC2(C(=C1)C(C)CC1C2C(O)CC2(C1CCC2C(=O)C)C)C
N#Cc1ccc(cc1)Nc1nc(N)c(c(n1)Oc1c(C)cc(cc1C)C#N)Br
COCC(=O)Nc1cc(ccc1N=C(NC(=O)OC)NC(=O)OC)Sc1ccccc1
O=C(Nc1ccccc1)C=Cc1c([nH]c2c1c(Cl)cc(c2)Cl)C(=O)O
CC(=O)NCCOC(=O)C(c1ccc(cc1)Cl)Oc1cccc(c1)C(F)(F)F
O=C1CCC2(C(=C1)C(F)CC1C2CCC2(C1CCC2(Br)C(=O)C)C)C
OCN1C(=O)NC(=O)C1NC(=O)NCNC(=O)NC1C(=O)NC(=O)N1CO
O=C1CCC2(C(=C1)C(=CC1C2CCC2(C1CCC2(C)C(=O)C)C)C)C
CN1C2CCC1CC(C2)NC(=O)c1cn(C2CCCCC2)c2c(c1=O)cccc2
COc1ccc2c(c1)CCC(=C2c1ccc(cc1)OCCN1CCCC1)c1ccccc1
OC(C1CCc2c(O1)ccc(c2)F)CNCC(C1CCc2c(O1)ccc(c2)F)O
CCCc1c2oc(cc(=O)c2cc2c1n(CC)c(cc2=O)C(=O)O)C(=O)O
Fc1ccc2c(c1)onc2CCCN1CCC(CC1)n1c(=O)[nH]c2c1cccc2
COc1ccc(cc1)C(=O)Oc1ccc(cc1OC(=O)C)C(CNC(C)(C)C)O
O=C1CCCC(=O)C1C(=O)c1ccc(cc1[N+](=O)[O-])C(F)(F)F
OCC(CO[N+](=O)[O-])(CO[N+](=O)[O-])CO[N+](=O)[O-]
Fc1ccc(cc1)C(=O)C1CCN(CC1)CCc1c(C)nc2n(c1=O)cccc2
COc1cc(cc(c1OC)OC)C1C2C(=O)OCC2C(c2c1cc1OCOc1c2)O
CCC(CO[N+](=O)[O-])(CO[N+](=O)[O-])CO[N+](=O)[O-]
CC(=O)C1CCC2C1(C)CCC1C2CC=C2C1(C)CCC(=C2)OC1CCCC1
O=C(N(C1CCC2(CC1N1CCCC1)CCCO2)C)Cc1ccc(c(c1)Cl)Cl
CC(=O)Nc1ccc(cc1)S(=O)(=O)Nc1ccc(cc1)[N+](=O)[O-]
Brc1cnc(c(c1)C)CCCCNc1ncc(c(=O)[nH]1)Cc1ccc(nc1)C
Fc1cc(F)cc(c1)C(=O)NC1c2cc(ccc2OC(C1O)(C)C)C(=O)C
COc1cc2nc(nc(c2cc1OC)N)N1CCN(CC1)C(=O)c1nnc(o1)SC
CCOC(=O)OCC1OC(Oc2ccccc2Cc2ccc(cc2)OC)C(C(C1O)O)O
NC(Cc1cc(F)c(cc1F)F)CC(=O)N1CCn2c(C1)nnc2C(F)(F)F
O=C1OC(CN1c1ccc(cc1)N1CCOCC1=O)CNC(=O)c1ccc(s1)Cl
O=C(c1sccc1S(=O)(=O)Nc1onc(c1Cl)C)Cc1cc2OCOc2cc1C
O=C(OCc1ccccc1)NCC1CN(C)C2C(C1)c1cccc3c1c(C2)cn3C
O=C(C(c1ccccc1)N=N#N)NC1C(=O)N2C1SC(C2C(=O)O)(C)C
CCCCOCC(CN1C(=O)NC(=O)C(C1=O)(CC)c1ccccc1)OC(=O)N
OCC(C(c1ccc(cc1)[N+](=O)[O-])O)NC(=O)CN=[N+]=[N-]
COC1C(O)C(N)C(C(C1N(C(=O)CN)C)O)OC1OC(CCC1N)C(N)C
CC(=O)OCC1=C(C(=O)O)N2C(SC1)C(C2=O)NC(=O)Cc1cccs1
O=C(NC1C(=O)N2C1SCC(=C2C(=O)O)COC(=O)C)CSc1ccncc1

Continued on next page

Cc1ncc(c(n1)N)C[n+]1csc(c1C)CCOP(=O)(OP(=O)(O)O)O
COc1c(N2CCNC(C2)C)c(F)cc2c1n(cc(c2=O)C(=O)O)C1CC1
O=C1CCC2(C(=C1)C(=CC1C2CCC2(C1CCC2(O)C(=O)C)C)C)C
CCCCCCCCCC(=O)OC1CCC2C1(C)CCC1C2CCC2=CC(=O)CCC12C
NCC(=O)OCC(C(c1ccc(cc1)S(=O)(=O)C)O)NC(=O)C(Cl)Cl
O=C(N1CSCC1)C1NCC(C1)N1CCN(CC1)c1cc(nn1c1ccccc1)C
CC(C(OC(=O)C(C)C)OC(=O)NCC(c1ccc(cc1)Cl)CC(=O)O)C
CC(C1C(=O)N2C1SC(=C2C(=O)OCc1oc(=O)oc1C)C1CCCO1)O
CCCn1c2nc([nH]c2c(=O)n(c1=O)CCC)C12CC3CC2CC(C1)C3
CCc1cc2CC(Cc2cc1CC)NCC(c1ccc(c2c1ccc(=O)[nH]2)O)O
FC(=C)c1sc(c(c1Br)C)CNCCCNc1cc(=O)c2c([nH]1)cccc2
COc1ccc(c(c1)C)N1CCc2c1nc(C)cc2n1ccc(n1)N1CCNC1=O
N#CC1CCCN1C(=O)CNC(CNC(=O)c1cnc2n(c1)nc(c2)C)(C)C
NC(=N)Nc1ccc(cc1)C(=O)Oc1ccc(cc1)SCCN1C(=O)CCC1=O
COc1cccc(c1)C(=O)Nc1ccc(c(c1)c1ccnn1C)OCCN1CCOCC1
CCCC(c1cccnc1)Nc1nc(ncc1C)c1ccc(c(c1)OC)NC(=O)NCC
OC1CCC2(C(=CCC3C2CCC2(C3CC=C2n2cnc3c2cccc3)C)C1)C
Oc1cc(O)c(cc1c1n[nH]c(=O)n1c1ccc2c(c1)ccn2C)C(C)C
O=C1NC(=O)C(C1c1c[nH]c2c1cccc2)c1cn2c3c1cccc3CCC2
CCOC(=O)C(CC(Cc1ccc(cc1)c1ccccc1)NC(=O)CCC(=O)O)C
N#CCNC(=O)c1ccc(cc1)c1ccnc(n1)Nc1ccc(cc1)N1CCOCC1
OC1CC(CC1COS(=O)(=O)N)n1ccc2c1ncnc2NC1CCc2c1cccc2
CCOC(=O)Nc1ccc2c(c1)N(C(=O)CCN(CC)CC)c1c(S2)cccc1
Nc1ncc(c(c1)C(F)(F)F)c1cc(nc(n1)N1CCOCC1)N1CCOCC1
CCOc1ncc(c2c1C(C(=C(N2)C)C(=O)N)c1ccc(cc1OC)C#N)C
Fc1cccc(c1)C1=C(OC(C1=O)(C)C)c1ccc(cc1)S(=O)(=O)N
OC(=O)COCC1CCC(CC1)COC(=O)N(c1ccc(cc1)Cl)c1ccccc1
COCCOc1ccc(cc1)Nc1ncc(c(n1)Nc1cccc(c1)NC(=O)C=C)F
OC1CCC2(C(C1)CCC1C2CC(O)C2(C1CCC2C(CCC(=O)O)C)C)C
Fc1ccc(c(c1)F)Sc1ccc2n(n1)cnc(=O)c2c1c(Cl)cccc1Cl
OCC(=O)C1(O)C(C)CC2C1(C)CC=C1C2CCC2=CC(=O)C=CC12C
O=C(C1CC1(COc1cnc(nc1C)C)c1cccc(c1)F)Nc1ccc(cn1)F
OCC(C(C(Cn1c2cc(C)c(cc2nc2c1nc(=O)[nH]c2=O)C)O)O)O
OC1CCC(=C)C(=CC=C2CCCC3(C2CCC3C(CCCC(O)(C)C)C)C)C1
COC1CN(CCCOc2ccc(cc2)F)CCC1NC(=O)c1cc(Cl)c(cc1OC)N
OC1CCC(C(=CC=C2CCCC3(C2CCC3C(C=CC(C(C)C)C)C)C)C1)C
Fc1ccc(cc1)C(=O)CCCN1CCC(=CC1)n1c(=O)[nH]c2c1cccc2
O=C1C=CC2(C(N1)CCC1C2CCC2(C1CCC2C(=O)NC(C)(C)C)C)C
CCOC(=O)C(NC(C(=O)N1C2CCCCC2CC1C(=O)O)C)CCc1ccccc1
CCOC(=O)C(NC1CCc2c(N(C1=O)CC(=O)O)cccc2)CCc1ccccc1
COc1c(CC=C(CCC(=O)OCCN2CCOCC2)C)c(O)c2c(c1C)COC2=O
ON=C(c1csc(n1)N)C(=O)NC1C(=O)N2C1SCC(=C2C(=O)O)C=C
CNCCC(C1NCC(C1)SC1=C(C(=O)O)N2C(C1C)C(C2=O)C(O)C)O
OC(=O)CCn1c2CCC(Cc2c2c1cccc2)NS(=O)(=O)c1ccc(cc1)F
C#CC1(CCC2C1(C)CCC1C2CCC2=CC(CCC12)OC(=O)C)OC(=O)C
NC(=O)C1(CCN(CC1)CCCN1c2ccccc2CCc2c1cccc2)N1CCCCC1
CCC(C(=O)NC1C(=O)N2C1SC(C2C(=O)[O-])(C)C)Oc1ccccc1
CC(CCOC(=O)c1cc(=O)c2c(o1)c1ccc(c(c1[nH]c2=O)C)C)C
CCOC(=O)Nc1ccc2c(c1)N(C(=O)CCN1CCOCC1)c1c(S2)cccc1
O=C1N(COP(=O)([O-])[O-])C(=O)NC1(c1ccccc1)c1ccccc1
COc1cccc(c1C(=O)NC1C(=O)N2C1SC(C2C(=O)[O-])(C)C)OC
Cc1ccc(cc1)S(=O)(=O)NC(=O)NC1C(O)C2(C(C1CC2)(C)C)C
O=c1[nH]c2c(n1CCN1CCN(CC1)c1cccc(c1)C(F)(F)F)cccc2
COCc1c(ncc2c1c1cc(OCc3ccccc3)ccc1[nH]2)C(=O)OC(C)C
Fc1ccc(cc1)C(=O)CCCN1CCC(CC1)(O)c1cccc(c1)C(F)(F)F
Fc1ccc(cc1)N1CCN(CC1)CCCN1c2cccc3c2c(S1(=O)=O)ccc3
Fc1ccc(cc1)C(=O)CCCN1CCC2(CC1)C(=O)NCN2c1ccc(cc1)F
Fc1ccc2c(c1)onc2C1CCN(CC1)CCc1c(C)nc2n(c1=O)cccc2C
O=C(c1cccnc1Nc1cccc(c1)C(F)(F)F)OC1OC(=O)c2c1cccc2
CCCCCC(C=CC1C(O)CC(=C1CCCCCCC(=O)OCCCC)OC(=O)CCC)O
[O-]C(=O)CCCC=CCC1C(O)CC(C1C=CC(COc1cccc(c1)Cl)O)O
CC(=O)[N-]S(=O)(=O)c1cc(N)ccc1S(=O)(=O)c1ccc(cc1)N
CCC(C(=O)O)(CC(=O)Nc1cccc(c1)C=Cc1scc(n1)C1CCC1)CC
O=C(C1CN(C)C2C(C1)c1cccc3c1c(C2)cn3C(C)C)NC1CCCCC1
O=C1CC2CC(C1C)OOC2(C)C=Cc1ccc(cc1C(F)(F)F)C(F)(F)F
CC(=O)OC1C(OC(=O)C)C(OC1n1ncc(=O)[nH]c1=O)COC(=O)C
CCCCOP(=O)(CC(CP(=O)(OCCCC)OCCCC)CCOc1ccccc1)OCCCC
NC(=O)OCc1nc(c(n1Cc1ccncc1)Sc1cc(Cl)cc(c1)Cl)C(C)C
Clc1cccc(c1)N1CCN(CC1)CCCn1c(=O)[nH]c2c(c1=O)cccc2
CCC1CC(=O)C2C34C1C(Cc1c4c(O2)c(cc1)OC)N(CC3)CC1CC1
CCOC(=O)NC1=C(N2CC2)C(=O)C(=C(C1=O)N1CC1)NC(=O)OCC
CC(C(=O)N1CC2(CC1C(=O)O)SCCS2)NC(C(=O)O)CCc1ccccc1
ONC(=O)CC(C(=O)NC(C(=O)NC)Cc1c[nH]c2c1cccc2)CC(C)C
OC(c1cc(nc(c1)c1ccc(cc1)C(F)(F)F)C(F)(F)F)C1CCCCN1
O=C1C(=CNCCNC=C2C(=O)C(OC2(C)C)(C)C)C(OC1(C)C)(C)C
CCCCCNC(=O)c1coc(n1)C1C2CCC(C1Cc1ccccc1CCC(=O)O)O2
CC1=CC(=O)N2C(O1)(c1ccccc1)c1cc(Cl)ccc1N(C(=O)C2)C
OC(CN1CCC(CC1)N(c1nc2c(s1)cccc2)C)COc1ccc(c(c1)F)F
CCOC1CC2(C)C(CC1O)CCC1C2C(CC2(C1CCC2C(=O)C)C)N(C)C
[O-]C(=O)CCC(C(=O)NC(C(=O)[O-])Cc1c[nH]c2c1cccc2)N
Fc1ccc2c(c1)onc2C1CCN(CC1)CCc1c(C)nc2n(c1=O)CCCC2O

Continued on next page

CC(=O)OCOC(=O)C1N2C(=O)C(C2SC1(C)C)NC(=O)Cc1ccccc1
COc1cccc2c1OC1C(C2)C(=O)C2C(C1(C)C)Oc1c(C2)cccc1OC
CC(=CCN1CCN(CC1)Cc1ccc2c(c1)OCO2)CCC=C(CCC=C(C)C)C
CCCCCC(C=CC1C(O)Cc2c1cc(n2c1ccccc1)CCCCC(=O)[O-])O
COC12C3C(N3C)CN2C2=C(C1COC(=O)N)C(=O)C(=C(C2=O)C)N
COC(=O)CCC=CCCC1C(=O)CC(C1C=CCC(C=CC1=CCCC1)(O)C)O
Fc1ccc(cc1)n1cc(C(=O)O)c(=O)c2c1cc(N1CCNCC1)c(c2)F
N#CNC(=Nc1cccc(c1)C(=CCCCC(=O)O)c1cccnc1)NC(C)(C)C
CCCCCCCCCCC(=O)OC1CCC2C1(C)CCC1C2CCC2=CC(=O)CCC12C
CCn1cc2c3c1cccc3C1C(C2)N(C)CC(C1)NC(=O)n1nc(cc1C)C
CCc1c(C(=O)C(=O)N)c2c(n1Cc1ccccc1)cccc2OCC(=O)[O-]
COC(=O)Nc1[nH]c2c(n1)ccc(c2)Sc1ccc(cc1)NC(=O)C(N)C
O=C1CCC2(C34C1Oc1c4c(CC2[N+](CC3)(C)CC2CC2)ccc1O)O
N#Cc1ccc(cc1)Cc1cc(c2n1cccc2)C(=O)C(=O)Nc1snc(c1)C
OCC(c1ccccc1)C(=O)OC1CC2C3C(C(C1)[N+]2(C)CC1CC1)O3
C[S+](CC1OC(C(C1O)O)n1cnc2c1ncnc2N)CCC(C(=O)[O-])N
CC(=O)Oc1cc(cc2c1C(=O)c1c(C2=O)cccc1OC(=O)C)C(=O)O
COc1ccccc1OC(=O)CNC(=O)Cc1ccc(n1C)C(=O)c1ccc(cc1)C
O=C(C(c1ccccc1)C(=O)O)NC1C(=O)N2C1SC(C2C(=O)O)(C)C
O=C1CCC2(C(=C1)C(=CC1C2CCC2(C1CCC2(O)C(=O)C)C)Cl)C
OCC(=O)C1(O)CCC2C1(C)CC(O)C1(C2CCC2=CC(=O)CCC12C)F
ClCOC(=O)C1(O)CCC2C1(C)CC(O)C1C2CCC2=CC(=O)C=CC12C
C=NC(c1ccccc1)C(=O)NC1C(=O)N2C1SC(C2C(=O)[O-])(C)C
CCCCCCCCCCCC(=O)OC1CCC2C1(C)CCC1C2CCC2=CC(=O)CCC12
COc1ccc2c3c1OC1C43CCN(C(C2)C4C=CC1OC(=O)c1cccnc1)C
CCCCCCC(=O)OC1(C#C)CCC2C1(C)CCC1C2CCC2=CC(=O)CCC12
CCCCCCCCN=c1ccn(cc1)CCCCCCCCCCn1ccc(=NCCCCCCCC)cc1
CCC[N+]12C3CC45C(C2CC(C3C4O)C(C1O)CC)N(C)c1c5cccc1
O=C(OC1CCC2C1(C)CCC1C2CCC2=CC(=O)CCC12C)CCc1ccccc1
CN1CCN(CC1)CCCN1c2ccccc2Sc2c1cc(cc2)S(=O)(=O)N(C)C
NC1CCCCN(C1)c1c(F)cc2c(c1Cl)n(cc(c2=O)C(=O)O)C1CC1
CC(=O)Nc1cccc(c1)N1CCN(CC1)CCCCNS(=O)(=O)CC1CCCCC1
COC(=O)N(c1c(N)nc(nc1N)c1nn(c2c1cccn2)Cc1ccccc1F)C
O=C(c1ccc(nc1)c1cc(cc(c1C)F)C(=O)NC1CC1)NCC(C)(C)C
COc1ccc(c(c1)C)c1c(C)nn2c1nc(C)cc2NC(c1onc(n1)C)CC
CC(=O)OC1CCC2(C(=CCC3C2CCC2(C3CC=C2c2cccnc2)C)C1)C
Nc1ncc(cc1OC(c1c(Cl)ccc(c1Cl)F)C)c1cnn(c1)C1CCNCC1
Clc1ccc2c(c1)N(CCC2=NOS(=O)(=O)[O-])C(=O)c1ccccc1C
CC(=O)OC1C(OC(=O)C)C(OC1n1ccc(=O)[nH]c1=O)COC(=O)C
O=C(c1ccc(cc1Cl)S(=O)(=O)C)Nc1ccc(c(c1)c1ccccn1)Cl
ONC(=O)C(C(C(=O)NC(c1ccccc1)C(=O)OC1CCCC1)CC(C)C)O
N#CCC1(CN(C1)S(=O)(=O)CC)n1ncc(c1)c1ncnc2c1cc[nH]2
Fc1ccc2c(c1)c1CN(CCc1n2CC(=O)O)C(=O)c1cccc2c1cccc2
CC1NCCN(C1)Cc1ccc(cc1)CC(=O)N1CCC(CC1)Nc1cccc(c1)F
Cc1ccc(c(c1)C(=O)N1CC(CCC1C)COc1ccc(cn1)F)c1ncccn1
CCCCCCCCCCCCCCCCOCCCOP(=O)(COC(Cn1ccc(nc1=O)N)CO)O
COCCOCCOCCOC(=O)C(=Cc1ccc2c(c1)ccc(c2)N1CCCCC1)C#N
CCC(C(=O)NCC(F)(F)F)(Nc1ccnc(n1)c1c[nH]c2c1cccn2)C
COc1ccc2c3c1OC1C43CCN(C(C2)C4CC=C1OC(=O)c1ccccc1)C
COCc1c(F)c(F)c(c(c1F)F)COC(=O)C1C(C1(C)C)C=C(C#N)C
[O-][N+](=O)c1cn2c(n1)OCC(C2)OCc1ccc(cc1)OC(F)(F)F
CN1CCC(CC1)CCCNc1ncc(c(n1)C)c1nc2c([nH]1)cc(cc2C)C
ONC(=O)CCCCCCNC(=O)c1cnc(nc1)N(c1ccccc1Cl)c1ccccc1
OC1CCC(=C)C(=CC=C2CCCC3(C2CCC3C(C=CC(C(C)C)C)C)C)C1
CN1CCN(CC1)CCC=C1c2ccccc2Sc2c1cc(cc2)S(=O)(=O)N(C)C
OCC(=O)C1(O)CCC2C1(C)CC(O)C1C2CC(C2=CC(=O)C=CC12C)C
CCOC(=O)C(NC(C(=O)N1Cc2ccccc2CC1C(=O)O)C)CCc1ccccc1
Clc1ccc2c(c1)c(cn2c1ccc(cc1)F)C1CCN(CC1)CCN1CCNC1=O
CCOc1cc(ccc1C(=O)O)CC(=O)NC(c1ccccc1N1CCCCC1)CC(C)C
CN(CCc1ccc(cc1)NS(=O)(=O)C)CCOc1ccc(cc1)NS(=O)(=O)C
CCc1nn(c(=O)n1CCOc1ccccc1)CCCN1CCN(CC1)c1cccc(c1)Cl
N#CCSCC(=O)NC1(OC)C(=O)N2C1SCC(=C2C(=O)O)CSc1nnnn1C
OC(=O)CC=C(C(=O)NC1C(=O)N2C1SCC=C2C(=O)O)c1csc(n1)N
CCC(=C(c1ccc(cc1)OP(=O)(O)O)CC)c1ccc(cc1)OP(=O)(O)O
O=C1NC(C(=O)N1c1ccc(c(c1)C(F)(F)F)[N+](=O)[O-])(C)C
COc1ccc2c(c1OC)C(=O)OC2C1N(C)CCc2c1c(OC)c1c(c2)OCO1
CCOC(=O)Cn1cccc1c1nc(c(s1)c1ccc(cc1)OC)c1ccc(cc1)OC
O=C(C(c1ccccc1)(c1ccccc1)O)OC1CC2CCC(C1)[N+]12CCCC1
CCCCCN(C(=O)C(NC(=O)c1ccc(c(c1)Cl)Cl)CCC(=O)O)CCCOC
CCOC(=O)C(NC1CSC(CN(C1=O)CC(=O)O)c1cccs1)CCc1ccccc1
CC(CC(c1ccc(cc1)OCCOCC[N+](Cc1ccccc1)(C)C)(C)C)(C)C
O=C(OC1CCC2C1(C)CCC1C2CCC2=C(C(=O)CCC12)O)CCC1CCCC1
Fc1ccc(cc1)N1CCN(CC1)CCCC(=O)NC1c2ccccc2SCc2c1cccc2
CCCCCCC(=O)OC1CCC2C1(C)CCC1C2CCC2C1(C)C(=CC(=O)C2)C
O=C(C(NC(=O)c1ccccc1)Cc1ccc(cc1)O)Nc1ccc(cc1)C(=O)O
Clc1ccc2c(c1)N(CCCN1CCC3(CC1)SCC(=O)N3)c1c(S2)cccc1
O=C(NC1C(=O)N2C1SCC(=C2C(=O)[O-])CSc1nncs1)Cn1cnnn1
CCOC(=O)C(NC(C(=O)N1C(=O)N(CC1C(=O)O)C)C)CCc1ccccc1
CC#CCC(C(C=CC1C(O)CC2C1c1cccc(c1O2)CCCC(=O)[O-])O)C
CC(=O)OCC(=O)C1(O)CCC2C1(C)CC=C1C2CCC2=CC(=O)CCC12C
CN(C(=O)COC(=O)Cc1ccc(cc1)OC(=O)c1ccc(cc1)NC(=N)N)C

Continued on next page

Oc1ccc(cc1)C1C(CCC(c2ccc(cc2)F)O)C(=O)N1c1ccc(cc1)F
NC1CCN(C1)c1nc2n(cc(c(=O)c2cc1F)C(=O)O)c1ccc(cc1F)F
O=C(NC1C(=O)N2C1SCC(=C2C(=O)O)CSc1nnc(s1)C)Cn1cnnn1
CC(CCCC1(C)CCc2c(O1)c(C)c(c(c2C)O)C)CCCC(CCCC(C)C)C
COC1(NC(=O)Cc2cccs2)C(=O)N2C1SCC(=C2C(=O)O)COC(=O)N
FCC[N+]1(C)C2CCC1CC(C2)OC(=O)C(c1ccccc1)(c1ccccc1)O
COc1ccccc1C(=O)NCCc1ccc(cc1)S(=O)(=O)NC(=O)NC1CCCC1
[O-]S(=O)CNc1ccc(cc1)S(=O)(=O)c1ccc(cc1)NCS(=O)[O-]
CCCSc1ccccc1N1CCN(CC1)CCCCn1c(=O)[nH]c2c(c1=O)cccc2
CCCCCCCCCC=CC=CC(C(c1cccc(c1)c1nnn[nH]1)O)SCCC(=O)O
NC(c1ccc(cc1)O)C(=O)NC1C(=O)N2C1SC(C2C(=O)[O-])(C)C
CCCCCCCCCCCCCCCCCCN(CCCN(CCO)CCO)CCCCCCCCCCCCCCCCCC
Fc1ccc(cc1)CC1CCN(CC1)CCS(=O)c1ccc2c(c1)oc(=O)[nH]2
COc1ccc(cc1)C1Sc2cc(Cl)ccc2N(C(=O)C1OC(=O)C)CCN(C)C
CC(CCCC(C1CC(=O)C2=C3C(CCC12C)C1(C)CCC(CC1CC3)O)C)C
N#Cc1ccc(cc1)OCC(COc1cccc2c1c(=O)cc(o2)c1[n-]nnn1)O
OCC(=O)C1C(C)CC2C1(C)CC(O)C1(C2CCC2=CC(=O)C=CC12C)F
N#CC(c1c(Cl)cc(cc1Cl)n1ncc(=O)[nH]c1=O)c1ccc(cc1)Cl
CN1CCN(CC1)c1cc2c(cc1F)c(=O)c(cn2c1ccc(cc1)F)C(=O)O
O=C(Nc1ccc(cc1)O)C=C(C=CC=C(C=CC1=C(C)CCCC1(C)C)C)C
OCC(=O)C1C(C)CC2C1(C)CC(O)C1C2CC(C2=CC(=O)C=CC12C)F
OCC(=O)C1(O)CCC2C1(C)CC(O)C1C2CC(C2=CC(=O)C=CC12C)F
OC1CCC2C(C1)c1c(NC2C)cc(cc1OC(=O)C)OC(CCCc1ccccc1)C
COc1cc(ccc1c1cnco1)NC(=O)Nc1cccc(c1)CNC(=O)OC1COCC1
NC(=O)C1CCN(CC1)CCCN1c2ccccc2Sc2c1cc(cc2)S(=O)(=O)C
CC(=O)OC1(CCC2C1(C)CC(C)C1C2CCC2=CC(=O)CCC12)C(=O)C
FC(C(C(=O)Nc1cc(cc(c1N)[N+](=O)[O-])C(F)(F)F)(F)F)F
CCCc1c2oc(cc(=O)c2cc2c1oc(cc2=O)C(=O)[O-])C(=O)[O-]
[O-]C(=O)CCC1(O)CCC2C1(C)CCC1C2C2CC2C2=CC(=O)CCC12C
CCC(=O)C1(C)C(C)CC2C1(C)CC(O)C1C2CCC2=CC(=O)C=CC12C
O=c1sc2c([nH]1)c(O)ccc2CCNCCS(=O)(=O)CCCOCCc1ccccc1
CC(=O)SC1CC2=CC(=O)CCC2(C2C1C1CCC3(C1(CC2)C)CCCO3)C
O=C(c1cc(Cl)c(c2c1OCCO2)N)CCC1CCN(CC1)CCNS(=O)(=O)C
CCC(c1ccc(cc1)OCCCCCCN1CCN(CC1)c1ccc(c(c1)Cl)C)(C)C
O=C1CCC2(C(=C1)CCC1C2CCC2(C1CCC2OC(=O)Cc1ccccc1)C)C
CCOC(=O)N(S(=O)(=O)C(F)(F)F)c1cccc(c1)C(=O)c1ccccc1
CC(OC(=O)CCCC=CCC1C(O)CC(C1C=CC(COc1ccccc1)(F)F)O)C
O=C1NC(=O)C2(C1)C(=O)N(Cc1ccc(cc1F)Br)C(=O)c1n2ccc1
O=c1[nH]c2c(o1)c(ccc2)N1CCN(CC1)Cc1cccc(c1)c1ccccc1
OCc1ccc(c(c1)C(c1ccccc1)CCN(C(C)C)C(C)C)OC(=O)C(C)C
CCCC(=O)OC1C2OP(=O)(O)OCC2OC1n1cnc2c1ncnc2NC(=O)CCC
CON=C(c1csc(n1)N)C(=O)NC1C2SCC(=C(N2C1=O)C(=O)O)COC
N#CC(c1cccc(c1)Oc1ccccc1)OC(=O)C1C(C1(C)C)C=C(Cl)Cl
O=C1C=CC2(C(=C1)C(=CC1C2CCC2(C1CCC2(O)C(=O)C)C)Cl)C
N#CC(c1cccc(c1)Oc1ccccc1)OC(=O)C1C(C1(C)C)C=C(Br)Br
OC(=O)C1(C)CCCC2(C1CCc1c2cc(c(c1)C(C)C)S(=O)(=O)O)C
C#CC1(O)CCC2C1(C)CCC1C2CCc2c1ccc(c2)OS(=O)(=O)C(C)C
N#CC(c1cccc(c1)Oc1ccccc1)OC(=O)C(c1ccc(cc1)Cl)C(C)C
O=C1CCC2(C(=C1)CCC1C2(F)C(O)CC2(C1CCC2(O)C(=O)C)C)C
O=C1CCC2(C(=C1)CCC1C2CCC2(C1CCC2(OC(=O)C)C(=O)C)C)C
OC(=O)C1N2C(=O)C(C2SC1(C)C)NC(=O)c1c(C)onc1c1ccccc1
CCCCCCC(=O)OCCN1CCN(CC1)CCCN1c2ccccc2Sc2c1cc(Cl)cc2
CCC1OC23CCC(O1)(C3(C)CCC1C2CCC2=CC(=O)CCC12C)C(=O)C
CCC(C(C)C)CCC(C1CCC2C1(C)CCC1C2CC=C2C1(C)CCC(C2)O)C
CCC(C(=O)OC1C(OC(=O)C)c2c(OC1(C)C)ccc1c2oc(=O)cc1)C
COc1cc(C=Cc2ccc(c(c2)OP(=O)([O-])[O-])OC)cc(c1OC)OC
OCCC1CCCCN1c1cc(NCc2ccc[n+](c2)[O-])n2c(n1)c(CC)cn2
CCCCCCCCC=CCCCCCCCC(=O)OCC1OC(C(C1O)O)n1ccc(nc1=O)N
CCCCCC(c1ccc(cc1)N1C(CCCc2ccc(s2)C(=O)OCCO)CCC1=O)O
S=C(N1CCN(CC1)c1ncnc2c1oc1c2cccc1)NCc1ccc2c(c1)OCO2
COc1cc2nccc(c2cc1C(=O)N)Oc1ccc(c(c1)Cl)NC(=O)NC1CC1
O=C1CCC2(C3(C1)CCN(C2Cc1c3c(O)c(cc1)C(=O)N)CC1CC1)O
N#Cc1ccc(cc1)OCC(C(=O)Nc1ccc(c(c1)C(F)(F)F)C#N)(O)C
O=c1ccc2c([nH]1)cc(cc2)OCCCCN1CCN(CC1)c1cccc2c1ccs2
CCOc1ccc(cc1)Cc1cc(ccc1Cl)C12OCC(O1)(CO)C(C(C2O)O)O
Fc1ccc(c(c1)C1OCC(CC1N)N1Cc2c(C1)cn(n2)S(=O)(=O)C)F
O=C(Nc1cccc(c1C)F)Nc1ccc(c(c1O)S(=O)(=O)C1CCCNC1)Cl
Fc1ccc2c(c1)c1CCOC3(c1[nH]2)CCC(CC3)(N(C)C)c1ccccc1
O=C(C(C)C)OC1C(COC(=O)C(C)C)OC(C1(C)F)n1ccc(nc1=O)N
CCOC(=O)c1c(CSc2ccccc2)n(c2c1c(CN(C)C)c(O)c(c2)Br)C
CCC(c1nc2cccc(c2c(=O)n1c1ccccc1)F)Nc1ncnc2c1[nH]cn2
N#Cc1ccc(cc1)NC(=O)NC1CCN(C(C1)c1nc2c([nH]1)cccc2)C
NCC(NC(=O)c1oc(c(c1)c1c(Cl)cnn1C)Cl)Cc1ccc(c(c1)F)F
COc1cc2c(cc1OC)CCN1C2CC(OC(=O)C(C(C)C)N)C(C1)CC(C)C
CC#CCC(C(C=CC1C(O)CC2C1c1cccc(c1O2)CCCC(=O)[O-])O)C
OC(=O)CNC(=O)C1C(=O)N(C2CCCCC2)C(=O)N(C1=O)C1CCCCC1
CCOC(=O)C[N+]1(C)CCC(C1)OC(=O)C(c1ccccc1)(C1CCCC1)O
COc1ccc(cc1)CCN1CCC(CC1)Nc1nc2c(n1Cc1ccc(cc1)F)cccc2
CC=CC1=C(C(=O)O)N2C(SC1)C(C2=O)NC(=O)C(c1ccc(cc1)O)N
COC(=O)C1=C(C)NC(=C(C1c1ccccc1[N+](=O)[O-])C(=O)OC)C
COc1ccc(cc1OC)CCN(CCCC(c1ccc(c(c1)OC)OC)(C(C)C)C#N)C

Continued on next page

CN1C(CSCC(F)(F)F)Nc2c(S1(=O)=O)cc(c(c2)Cl)S(=O)(=O)N
CC(=O)OCC1=C(C(=O)[O-])N2C(SC1)C(C2=O)NC(=O)Cc1cccs1
O=C(NC1C(=O)N2C1SCC(=C2C(=O)[O-])COC(=O)C)CSc1ccncc1
N#Cc1nn(c(c1S(=O)C(F)(F)F)N)c1c(Cl)cc(cc1Cl)C(F)(F)F
OC(=O)C1N2C(=O)C(C2SC1(C)C)N1C(=O)C(NC1(C)C)c1ccccc1
CCCCCC(=O)OC1(CCC2C1(C)CCC1C2CCC2=CC(=O)CCC12)C(=O)C
CC(=O)Oc1ccc(cc1)C1(Oc2ccccc2NC1=O)c1ccc(cc1)OC(=O)C
CCN(Cc1nccn1c1ccc(cc1C(=O)c1ccccc1Cl)[N+](=O)[O-])CC
O=C(N1CCOCC1)CCc1sc2c(c1)C(=NCc1n2c(C)nn1)c1ccccc1Cl
COc1cc2CC(C)(O)C(C)Cc3c(c2c(c1OC)OC)c(OC)c1c(c3)OCO1
O=c1[nH]c2c(n1CCCN1CCN(CC1)C(c1ccccc1)c1ccccc1)cccc2
O=C(N1CCCC(c2c1cccc2)N(C)C)c1ccc(cc1)NC(=O)c1ccccc1C
CSCCC(C(=O)NCCc1ccc(c(c1)OC(=O)OCC)OC(=O)OCC)NC(=O)C
CCCc1nc2c(n1Cc1ccc(cc1)c1ccccc1c1[nH]nnn1)C(=O)CCCC2
O=C(C(c1cccs1)(c1cccs1)O)OC1CC2C3C(C(C1)[N+]2(C)C)O3
Clc1ccc(cc1)C(c1ccccc1)N1CCN(CC1)Cc1ccc(cc1)C(C)(C)C
Cc1onc(c1)C(=O)NCCc1ccc(cc1)S(=O)(=O)NC(=O)NC1CCCCC1
CC1NCCN(C1)c1cc2n(cc(c(=O)c2cc1F)C(=O)O)c1ccc(cc1F)F
N#Cc1ccc(cc1)c1csc(n1)C(C(c1ccc(cc1F)F)(Cn1cncn1)O)C
CC(CCC(=O)CC1N(c2ccc3c(n2)nc(cc3)Cl)C(=O)c2c1cccc2)C
OC(COc1ccc(cc1)CC(=O)OCCC12CC3CC(C2)CC(C1)C3)CNC(C)C
CCCCC(CNC(=N)NC(=N)NCCCCCCNC(=N)NC(=N)NCC(CCCC)CC)CC
COc1ccc(cc1)c1sc2c(c1Oc1ccc(cc1)OCCN1CCCCC1)ccc(c2)O
Fc1ccc2c(c1)c(C(=O)[O-])c(c(n2)c1ccc(cc1)c1ccccc1F)C
CCC1(O)C(=O)OCc2c1cc1c3nc4ccccc4c(c3Cn1c2=O)CCNC(C)C
CCOC(=O)C(NC(C(=O)N1CC2(CC1C(=O)O)SCCS2)C)CCc1ccccc1
N#CC(c1ccccc1)(c1ccccc1)CCN1CCC(CC1)(C(=O)O)c1ccccc1
O=C1CCC2(C(=C1)C1CC1C1C2CCC2(C1C1CC1C12CCC(=O)O1)C)C
CCOC(=O)Nc1ccc(cc1)SCC1COC(O1)(Cn1cncc1)c1ccc(cc1)OC
COc1ccc(cc1CNC1C2CCN(C1C(c1ccccc1)c1ccccc1)CC2)C(C)C
CCC1C2CC3N(C1O)C1C2C(OC(=O)CCl)C2(C3N(C)c3c2cccc3)C1
OC(=O)CC1Nc2ccc(cc2CN(C1=O)C)C(=O)N1CCC(CC1)C1CCNCC1
Fc1ccc(cc1)C(=O)C1CCN(CC1)CCc1c(C)nc2n(c1=O)cc(cc2)C
C#CC1(CCC2C1(C)CCC1C2CC=C2C1CCC(=C2)OC1CCCC1)OC(=O)C
Clc1ccc(cc1)c1c(C)c(nn1c1ccc(cc1Cl)Cl)C(=O)NN1CCCCC1
CCOC(=O)COC1CCN(CC1)C(=O)C(NC(=O)c1ccc(cc1)C(=NO)N)C
CC(=Cc1ccc(cc1)S(=O)(=O)C)c1ccc2c(c1)C(C)(C)CCC2(C)C
CCOCCN(C(=O)C(Cl)Cl)Cc1ccc(cc1)CN(C(=O)C(Cl)Cl)CCOCC
O=C(C(c1cscc1)C(=O)O)NC1C(=O)N2C1SC(C2C(=O)[O-])(C)C
COc1c(OC)c(OC)cc2c1nc(nc2N)N1CCN(CC1)C(=O)OCC(O)(C)C
OCC(=O)C1(O)C(C)CC2C1(C)CC(=O)C1C2CCC2=CC(=O)C=CC12C
CCCN(C(=O)C(NC(=O)c1ccccc1)Cc1ccc(cc1)OCCN(CC)CC)CCC
COc1cc(C[N+]2(CCOCCC3CCC4CC3C4(C)C)CCOCC2)c(cc1OC)Br
O=C(N1c2ccccc2Sc2c1cccc2)CN1CCN(CC1)Cc1ccc2c(c1)OCO2
Clc1ccc(cc1)C(CC(c1c(=O)oc2c(c1O)cccc2)c1ccc(s1)Cl)O
OCC(=O)C1(O)C(=C)CC2C1(C)CC(O)C1C2CCC2=CC(=O)C=CC12C
N#CC(c1ccccc1)(c1ccccc1)CCN1CCC(CC1)(N1CCCCC1)C(=O)N
OCCN(C(=O)C(Cl)Cl)Cc1ccc(cc1)Oc1ccc(cc1)[N+](=O)[O-]
O=C(C(c1ccccc1)N=N#N)NC1C(=O)N2C1SC(C2C(=O)[O-])(C)C
[O-]C(=O)COc1cc(OCC(=O)[O-])c2c(c1)oc(cc2=O)c1ccccc1
OC(=O)CC(CC(C=Cc1c(c2ccc(cc2)F)c2c(n1C(C)C)cccc2)O)O
O=C(C1CCC(CC1)NS(=O)(=O)C(C)(C)C)Nc1ccc(cn1)C(F)(F)F
CC(C1C(=O)N2C1C(C)C(=C2C(=O)O)SC1CNC(C1)C(=O)N(C)C)O
CCOc1ccc2c(c1C(=O)NC1C(=O)N3C1SC(C3C(=O)O)(C)C)cccc2
CC(=O)OC1(CCC2C1(C)CCC1C2C=C(C2=CC(=O)CCC12)C)C(=O)C
OCCC1CCN(CC1)CCCN1c2ccccc2Sc2c1cc(cc2)S(=O)(=O)N(C)C
[O-]C(=O)CCCCCCNC1c2ccccc2N(S(=O)(=O)c2c1ccc(c2)Cl)C
COc1cc2c(ncnc2cc1OCCCN1CCCC1)Oc1ccc2c(c1F)cc([nH]2)C
O=c1[nH]c(=Cc2nc[nH]c2C(C)(C)C)c(=O)[nH]c1=Cc1ccccc1
COc1cc2c(ccnc2cc1OC)Oc1ccc(c(c1)Cl)NC(=O)Nc1noc(c1)C
CCCCCCCCCCCCCCCC(=O)Nc1ccn(c(=O)n1)C1OC(C(C1C#N)O)CO
COc1cc2ncnc(c2cc1NC(=O)C=CCN1CCCCC1)Nc1ccc(c(c1)Cl)F
CN(CCCOc1ccc2c(c1)c(n[nH]2)S(=O)(=O)c1cccc2c1cccc2)C
CCCNS(=O)(=O)Nc1ncnc(c1c1ccc(cc1)Br)OCCOc1ncc(cn1)Br
O=C(Nc1cccc(c1Cl)F)Nc1ccc(c(c1O)S(=O)(=O)N1CCNCC1)Cl
OCC1OC(c2ccc(c(c2)Cc2ccc(cc2)OC2COCC2)Cl)C(C(C1O)O)O
Fc1ccc(c(c1)F)CNC(=O)c1cn2CC3OCC(N3C(=O)c2c(c1=O)O)C
CNC(=O)c1ccc(cc1F)c1cnc2n(n1)c(cn2)Cc1ccc2c(c1)cccn2
OC1CCC2(C(C1)CC(C1C2CC(O)C2(C1CCC2C(CCC(=O)O)C)C)O)C
N#Cc1ccc(cc1)c1csc(n1)C(C(c1cc(F)ccc1F)(Cn1cncn1)O)C
CC(=O)OC1(C(=O)C)C(=C)CC2C1(C)CCC1C2CCC2=CC(=O)CCC12
OC1CC(O)C(=C)C(=CC=C2CCCC3(C2CCC3C(CCCC(O)(C)C)C)C)C1
CC(=O)OCC(=O)C1(O)CCC2C1(C)CC(O)C1C2CCC2=CC(=O)CCC12C
CON=C(C(=O)NC1C(=O)N2C1SCC(=C2C(=O)O)COC(=O)N)c1ccco1
Cc1ncc(nc1)C(=O)NCCc1ccc(cc1)S(=O)(=O)NC(=O)NC1CCCCC1
CCC(C(=O)OC1CC(C)C=C2C1C(CCC1CC(O)CC(=O)O1)C(C=C2)C)C
CCCCC1=NC2(C(=O)N1Cc1ccc(cc1)c1ccccc1c1nnn[nH]1)CCCC2
OC1CCC(=C)C(=CC=C2CCCC3(C2CCC3C(CCC(C(O)(C)C)O)C)C)C1
O=S1(=O)NC(Cc2ccccc2)Nc2c1cc(c(c2)C(F)(F)F)S(=O)(=O)N
COc1c(N2CC3C(C2)CCCN3)c(F)cc2c1n(cc(c2=O)C(=O)O)C1CC1
OC1CC(O)CC(=CC=C2CCCC3(C2CCC3C(C=CC(C(O)(C)C)C)C)C)C1

Continued on next page

Table S8 – *Continued from previous page*

CCCCCC(=O)OC1(CCC2C1(C)CCC1C2CCC2=CC(=O)CCC12C)C(=O)C
CC(C1C(=O)N2C1C(C)C(=C2C(=O)[O-])SC1C[n+]2n(C1)cnc2)O
OC1CC(O)C(=C)C(=CC=C2CCCC3(C2CCC3C(OCCC(O)(C)C)C)C)C1
Clc1ccc2c(c1)C(O)CCCN2C(=O)c1ccc(cc1C)NC(=O)c1ccccc1C
O=CN(C(=C(SC(=O)c1ccccc1)CCOP(=O)(O)O)C)Cc1cnc(nc1N)C
COC12CC(COC(=O)c3cncc(c3)Br)CN(C1Cc1c3c2cccc3n(c1)C)C
OC1CC(O)C(=C)C(=CC=C2CCCC3(C2CCC3C(CCC(C(C)C)O)C)C)C1
COc1ccc2c(c1)c(CC(=O)OCC(=O)O)c(n2C(=O)c1ccc(cc1)Cl)C
NC(=O)C(c1ccccc1)(c1ccccc1)C1CCN(C1)CCc1ccc2c(c1)CCO2
CC(=O)OCC1=C(C(=O)O)N2C(SC1)C(C2=O)NC(=O)C(c1ccccc1)N
CCC1(O)C(=O)OCc2c1cc1c3nc4ccc(c(c4cc3Cn1c2=O)CN(C)C)O
OC(=O)Cc1nn(Cc2nc3c(s2)ccc(c3)C(F)(F)F)c(=O)c2c1cccc2
OC(C(=O)NC1C(=O)N2C1SCC(=C2C(=O)O)CSc1nnnn1C)c1ccccc1
Fc1ccc(cc1)C(=O)C1CCN(CC1)CCn1c(=O)[nH]c2c(c1=O)cccc2
O=C(C1CSC(C(=O)N1)C)NC(C(=O)N1CCCC1C(=O)N)Cc1cnc[nH]1
Fc1ccc(cc1)C(=O)C1CCN(CC1)CCCn1c(=O)[nH]c2c1ccc(c2)Cl
Fc1ccc(cc1)C(CCCN1CCc2c(C1)c1cc(F)ccc1n2c1ccc(cc1)F)O
Fc1ccc(cc1)C(=O)NCCN1CCC(CC1)n1c(=O)[nH]c2c1ccc(c2)Cl
Fc1ccc(cc1)C(=O)C1CCN(CC1)CCn1c(=S)[nH]c2c(c1=O)cccc2
CC(=O)OC1(CCC2C1(C)CCC1C2C=C(C2=CC(=O)CCC12)Cl)C(=O)C
COc1cncnc1N1CCN(CC1)CCCc1c[nH]c2c1cc(cc2)CS(=O)(=O)NC
CNC(=O)C(NC(=O)C(C(C(=O)NO)CSc1cccs1)CC(C)C)Cc1ccccc1
CCCCc1nc2c(n1CC)ccc(c2)CN1CCN(CC1)C(c1ccccc1)c1ccccc1
O=C(C(c1ccccc1)C(=O)[O-])NC1C(=O)N2C1SC(C2C(=O)O)(C)C
OCC(=O)C1(O)CCC2C1(C)CC(O)C1C2C=C(C2=CC(=O)C=CC12C)Cl
OC1CC(=O)OC(C1)C=CC1=C(CC(CC1(C)C)(C)C)c1ccc(c(c1)C)F
CN(Cc1ccc(o1)CSCCNc1[nH]cc(c(=O)n1)Cc1cc[nH]c(=O)c1)C
FC(c1ccc(cc1)C=Cc1occ(n1)COc1ccc(cc1)CCCCn1nncc1)(F)F
CCS(=O)(=O)c1ccc(cc1)C=C(c1ccc2c(c1)C(C)(C)CCC2(C)C)C
CCCCCNC(=O)c1coc(n1)C1C2CCC(C1Cc1ccccc1CCC(=O)[O-])O2
CC(=NNc1ccc(cc1)S(=O)(=O)C)c1ccc2c(c1)C(C)(C)CCC2(C)C
C#CC1(CCC2C1(C)CC(C)C1C2CCC2=CC(CCC12)OC(=O)C)OC(=O)C
COC(=O)C1C2CC3N(CC2CC(C1OC)OC)CCc1c3[nH]c2c1ccc(c2)OC
COc1cc(ccc1OCCCCCOc1ccc(cc1)C(=N)N)C(=O)N(C(C)C)C(C)C
O=C(OC(C1CCC2C1(C)CCC1C2CCC2=CC(=O)CCC12)C)CCc1ccccc1
CC(=O)Oc1ccc(cc1)C1(c2ccc(cc2)OC(=O)C)C(=O)Nc2c1cccc2
CNC(C1CCN(C1)c1c(F)cc2c(c1OC)n(cc(c2=O)C(=O)O)C1CC1)C
OC(c1ccccn1)(c1ccccn1)C1=CC(=C(c2ccccn2)c2ccccn2)C=C1
COC1(NC(=O)C(c2cscc2)C(=O)O)C(=O)N2C1SC(C2C(=O)O)(C)C
Clc1ccc(cc1)OC(C(=O)OCCn1cnc2c1c(=O)n(C)c(=O)n2C)(C)C
OC(=O)CCC=CCCC1C(OCc2ccc(cc2)c2ccccc2)CC(C1N1CCCCC1)O
O=C1N(CCCCN2CCN(CC2)c2ncccn2)C(=O)C2C1C1C=CC2C2C1C=C2
[N-]=[N+]=Nc1ccc(cc1)C(=O)n1c2ccc(cc2c(c1C)CC(=O)O)OC
CCN(CCNC(=O)c1c(C)[nH]c(c1C)C=C1C(=O)Nc2c1cc(F)cc2)CC
CC(=O)CC(c1c(=O)oc2c(c1O)cccc2)c1ccc(cc1)[N+](=O)[O-]
Cc1onc(c1)C(=O)NCCc1ccc(cc1)S(=O)(=O)NC(=O)NN1CCCCCC1
CCOC(=O)C(c1c(=O)oc2c(c1O)cccc2)c1c(=O)oc2c(c1O)cccc2
OC(=O)CNC(C(=O)N1CCC1C(=O)NCc1ccc(cc1)C(=N)N)C1CCCCC1
COc1cc2CC(c2cc1OC)CN(CCCN1CCc2c(CC1=O)cc(c(c2)OC)OC)C
COCc1c(ncc2c1c1c(cccc1[nH]2)Oc1ccc(cc1)Cl)C(=O)OC(C)C
OC(=O)C1N2C(=O)C(C2SC1(C)C)NC(=O)c1c(C)onc1c1ccccc1Cl
O=C1C=C2C(=CC3C(C2(C2C1C2)C)CCC1(C3CCC1(O)C(=O)C)C)Cl
NCC1CCC(C(O1)OC1C(N)CC(C(C1O)OC1OC(CO)C(C(C1O)N)O)N)N
OCC(=O)C1(O)CCC2C1(C)CC(Cl)C1(C2CCC2=CC(=O)C=CC12C)Cl
CCC(=O)OC1CCC2(C(=CCC3C2CCC2(C3CCC2(C)OC(=O)CC)C)C1)C
OCC1OC(C(C1O)O)OC1C(OC2OC(CN)C(C(C2N)O)O)C(N)CC(C1O)N
OC(=O)CC1CCc2c1n(Cc1ccc(cc1)Cl)c1c2cc(cc1S(=O)(=O)C)F
O=C1CCC(C1)CC(c1ccc(c(c1)Cl)S(=O)(=O)C)C(=O)Nc1cnccn1
OC(COc1cc(CCC(=O)O)cc(c1F)F)CNC(CC1Cc2c(C1)cccc2)(C)C
OC(=O)Cn1c2cc(n3cncc3)c(cc2[nH]c(=O)c1=O)[N+](=O)[O-]
ONC(=O)C1CC2(CC2)CN(C1C(=O)N1CCN(CC1)c1ccccc1)C(=O)OC
CCC1C(O)C2C3CCC(C3(C)CCC2C2(C1CC(O)CC2)C)C(CCC(=O)O)C
OC(=O)c1ccc(cc1)C=Cc1cc2c(cc1Cn1cccn1)C(C)(C)CCC2(C)C
O=C(c1ccc(cc1)C(F)(F)F)NN1C(=O)C2C(C1=O)C1C=CC2C2C1C2
CC1NCC(=C1)c1csc(n1)SC1=C(C(=O)O)N2C(C1C)C(C2=O)C(O)C
CC1NCCN(C1)c1c(F)cc2c(c1OC(F)F)n(cc(c2=O)C(=O)O)C1CC1
O=C(C=Cc1ccc(nc1)C)NCCCCN1CCN(CC1)C(c1ccccc1)c1ccccc1
CCCCCC(c1ccc(cc1)N1C(CCCc2ccc(s2)C(=O)OC(C)C)CCC1=O)O
CC(c1ccc(cc1)c1ccc(cc1)CCNS(=O)(=O)C)CNS(=O)(=O)C(C)C
FCC1(O)OC(=O)CC1NC(=O)C1(ON=C(C1)c1cccc2c1cncc2)C(C)C
CCCCCC(c1ccc(cc1)N1C(COCc2ccc(s2)C(=O)OC(C)C)CCC1=O)O
Fc1ccc(c(c1)F)CNC(=O)c1cn2CC3OCCC(N3C(=O)c2c(c1=O)O)C
CCC(c1ccc(o1)C)NC1=C(C(=O)C1=O)Nc1cccc(c1O)C(=O)N(C)C
NC1CCN(CC1)c1cccc2c1nc(cc2)n1cnc2c1ccc(c2)OCC1(C)COC1
COc1cc(OC)cc(c1C=CS(=O)(=O)Cc1ccc(c(c1)NCC(=O)O)OC)OC
OC(C12CC[N+](CC1)(CC2)CCOCc1ccccc1)(c1ccccc1)c1ccccc1
O=c1ccc2c([nH]1)c(O)ccc2C(CNCCCCCCOCC(c1ccccc1)(F)F)O
C=CC(=O)N1CCCC(C1)n1nc(c2c1ncnc2N)c1ccc(cc1)Oc1ccccc1
CC(Cn1cnc2c1ncnc2N)OCP(=O)(NC(C(=O)OC(C)C)C)Oc1ccccc1
N#Cc1cccnc1Oc1ccc(cc1)CC(NCC(COc1ccccc1c1cccs1)O)(C)C
NCCCC(Nc1cc(OC)c(c2c1nc(OC)cc2C)Oc1cccc(c1)C(F)(F)F)C

Continued on next page

Clc1cccc2c1c(=O)n(c(c2)C(Nc1ncnc2c1[nH]cn2)C)c1ccccc1
CN1CCN(CC1)Cc1ccc2c(c1)CN(C2)C(=O)c1cc(C(C)C)c(cc1O)O
O=C(C1CC1)Nc1nn2c(n1)cccc2c1ccc(cc1)C(=O)N1CC(C1)(C)C
OCC1OC(c2ccc(c(c2)Cc2ccc(cc2)OCCOC2CC2)Cl)C(C(C1O)O)O
CN(C(=O)c1cc2c(n1C1CCCC1)nc(nc2)Nc1ccc(cn1)N1CCNCC1)C
CNC(=O)c1cccc(c1)c1ccc2c(n1)nc(nc2N1CCOCC1C)N1CCOCC1C
OCC(c1cccc(c1)Cl)NC(=O)c1[nH]cc(c1)c1cc(ncc1Cl)NC(C)C
NCC1OC(OC2C(N)CC(C(C2O)OC2OC(CO)C(C(C2O)N)O)N)C(CC1O)N
OCC(=O)C1(O)C(C)CC2C1(C)CC(O)C1(C2CCC2=CC(=O)C=CC12C)F
Clc1ccc(cc1)Nc1cc2nc3ccccc3n(c2cc1=NC(C)C)c1ccc(cc1)Cl
OCC(=O)C1(O)C(O)CC2C1(C)CC(O)C1(C2CCC2=CC(=O)C=CC12C)F
CC1NC(C)CN(C1)c1c(F)c(N)c2c(c1F)n(cc(c2=O)C(=O)O)C1CC1
COc1cc2nc(nc(c2cc1OC)N)N1CCN(CC1)C(=O)C1COc2c(O1)cccc2
O=C(NC1C(=O)N2C1SCC(=C2C(=O)[O-])CSc1nnc(s1)C)Cn1cnnn1
N#CCSCC(=O)NC1(OC)C(=O)N2C1SCC(=C2C(=O)[O-])CSc1nnnn1C
COC1(NC(=O)Cc2cccs2)C(=O)N2C1SCC(=C2C(=O)[O-])COC(=O)N
[O-]C(=O)C1N2C(=O)C(C2SC1(C)C)NC(=O)c1c(C)onc1c1ccccc1
O=C1CCC2(C(=C1)C(C)CC1C2CCC2(C1CCC2(OC(=O)C)C(=O)C)C)C
CC(=O)OCC(=O)C1(O)CCC2C1(C)CC(=O)C1C2CCC2=CC(=O)CCC12C
CC(=O)OCC(=O)C1(O)CCC2C1(C)CC(O)C1C2CCC2=CC(=O)C=CC12C
OC1CC(O)C(=C)C(=CC=C2CCCC3(C2CCC3C(C=CC(C(C)C)C)C)C)C1
O=C(NC1C(=O)N2C1SCC(=C2C(=O)[O-])C[n+]1ccccc1)Cc1cccs1
OC1CC(O)C(=C)C(=CC=C2CCCC3(C2CCC3C(C=CC(C2CC2)O)C)C)C1
C[n+]1cccc(c1)OC(=O)N(CCCCCCN(C(=O)Oc1ccc[n+](c1)C)C)C
CCCC(=O)OC1C2OP(=O)([O-])OCC2OC1n1cnc2c1ncnc2NC(=O)CCC
CCCCC1(COC(=O)CCC(=O)O)C(=O)N(N(C1=O)c1ccccc1)c1ccccc1
CCC(=O)OC1(CCC2C1(C)CC(O)C1C2CCC2=CC(=O)C=CC12C)C(=O)C
O=C(C1N2C(=O)C(C2SC1(C)C)N=CN1CCCCCC1)OCOC(=O)C(C)(C)C
CNCC1CCC(C(O1)OC1C(N)CC(C(C1O)OC1OCC(C(C1O)NC)(C)O)N)N
CC(C1C(=O)N2C1C(C)C(=C2C(=O)O)SC1CNC(C1)CNS(=O)(=O)N)O
OCCN(CCNc1cc(=O)n(c(=O)n1C)C)CCCc1ccc(cc1)[N+](=O)[O-]
OC(=O)COc1cc(OCC=C(C)C)ccc1C(=O)C=Cc1ccc(cc1)OCC=C(C)C
OCCCN1CCc2c1c(cc(c2)CC(NCCOc1ccccc1OCC(F)(F)F)C)C(=O)N
O=C1N(C)CC(=O)N2C1Cc1c(C2c2ccc3c(c2)OCO3)[nH]c2c1cccc2
NC1C2C1CN(C2)c1nc2c(cc1F)c(=O)c(cn2c1ccc(cc1F)F)C(=O)O
NCC1=CCC(C(O1)OC1C(N)CC(C(C1O)OC1OCC(C(C1O)NC)(C)O)N)N
CCOC(=O)c1[nH]c2c(c1CCN1CCN(CC1)c1ccccc1)cc(c(c2)OC)OC
CC[N+](CCSC1=Nc2ccccc2N=C(C1)c1ccc(cc1)Sc1ccccc1)(CC)C
O=C(NS(=O)(=O)C)CCCC=CCC1C(=O)CC(C1C=CC(COc1ccccc1)O)O
CCCCN(CCCC)CCCOc1c(C)cc(cc1C)C(=O)c1cc(=O)c2c(o1)cccc2
OCC1CCCN1c1ncc(c(n1)NCc1ccc(c(c1)Cl)OC)C(=O)NCc1ncccn1
O=C(NC1C(=O)N2C1SCC(=C2C(=O)[O-])CSc1nnnn1C)CSC(F)(F)F
O=C1[N-]C(=O)C(S1)Cc1ccc(cc1)C(=O)CCc1nc(oc1C)c1ccccc1
OCC(=O)C1C(C)CC2C1(C)CC(O)C1(C2CC(C2=CC(=O)C=CC12C)F)F
CCOC(=O)C1(CCN(CC1)CCC(c1ccccc1)(c1ccccc1)C#N)c1ccccc1
CCOP(=O)(NC(=S)Nc1ccc(c(c1)OC)NC(=S)NP(=O)(OCC)OCC)OCC
CN(c1ccc2c3c1cccc3C(=N2)N)Cc1ccc(cc1)S(=O)(=O)N1CCOCC1
CCCSc1ccc(c(c1)N=C(NC(=O)OC)NCCS(=O)(=O)O)[N+](=O)[O-]
C#CC1(O)CCC2C1(C)CCC1C2CCC2=Cc3c(CC12C)cnn3c1ccc(cc1)F
O=C(OC1CCC2(C(=CCC3C2CCC2(C3CCC2C(=O)C)C)C1)C)CCC(=O)O
COC1CCC(CC1)OC(=O)C1CN(C)C2C(C1)c1cccc3c1c(C2)cn3C(C)C
Clc1ccc(cc1)c1cc(cc(c1O)CNC(C)(C)C)Nc1ccnc2c1ccc(c2)Cl
CCC(=O)N(C1(CCN(CC1)CCn1nnn(c1=O)CC)c1ccccc1)c1ccccc1F
CCOC(=O)Nc1ccc(cc1)SCC1COC(O1)(Cn1cncc1)c1ccc(cc1Cl)Cl
C#CC1(O)CCC2C1(C)CCC1C2CCC2C1(C)Cc1cn(nc1C2)S(=O)(=O)C
CN(Cc1ccc(o1)CSCCNC(=C[N+](=O)[O-])NCc1ccc2c(c1)OCO2)C
CCCCCCCCOc1ccccc1C(=O)Nc1ccc(cc1)C(=O)OCC[N+](CC)(CC)C
COC(c1ccc(c(c1)Cl)Cl)C(=O)NC1C(=O)N2C1SC(C2C(=O)O)(C)C
O=C(c1ccc(s1)[N+](=O)[O-])NN=CC=Cc1ccc(o1)[N+](=O)[O-]
CCOCCN(C(=O)C(Cl)Cl)Cc1ccc(cc1)Oc1ccc(cc1)[N+](=O)[O-]
OCC(=O)C1(O)C(C)CC2C1(C)CC(O)C1C2CC(C2=CC(=O)C=CC12C)F
COc1cc(ccc1OC(=O)C)C(=O)Nc1c(Br)cc(cc1CN(C1CCCCC1)C)Br
CCOc1cc(N)c(cc1C(=O)NC1CCN(CC1)CC1CCC=CC1)[N+](=O)[O-]
N#CC(c1ccc(c(c1)Oc1ccccc1)F)OC(=O)C1C(C1(C)C)C=C(Cl)Cl
CCNC1CC(N)C(C(C1OC1OCC(C(C1O)NC)(C)O)O)OC1OC(=CCC1N)CN
CC1NC(C)CN(C1)c1c(F)c(F)c2c(c1F)n(cc(c2=O)C(=O)O)C1CC1
COc1ccc2c(c1)OC(C(C2c1ccc(cc1)OCCN1CCCC1)c1ccccc1)(C)C
CCCCCCCCCC(=O)OCCN1CCN(CC1)CCCN1c2ccccc2Sc2c1cc(Cl)cc2
CCC(C(=O)OC1CC(O)C=C2C1C(CCC(CC(CC(=O)O)O)O)C(C=C2)C)C
O=C(C(S(=O)(=O)O)c1ccccc1)NC1C(=O)N2C1SC(C2C(=O)O)(C)C
Fc1ccc(cc1)C(=O)CCC[NH+]1CCC(CC1)(O)c1cccc(c1)C(F)(F)F
CCC(N(C(CC)C)CC(c1cccn1Cc1ccccc1Cl)OC(=O)c1ccc(cc1)O)C
CC1COC(=N1)Nc1ccc2c(c1)c(ncn2)Nc1ccc(c(c1)Cl)OCc1nccs1
Fc1ccc(c(c1)F)C(C(n1cnc2c(c1=O)ccc(c2)Cl)C)(Cn1ncnc1)O
NC1CN(CC21CC2)c1c(F)c(N)c2c(c1C)n(cc(c2=O)C(=O)O)C1CC1
Nc1nccn2c1c(nc2C1CC(C1)(C)O)c1ccc2c(c1)nc(cc2)c1ccccc1
OC1CCC(CC1)Nc1ncc2c(n1)n(C1COCC1)c(n2)Nc1c(F)cc(cc1F)F
CCCCCCCCC=CCCCCCCCC(=O)OCC1OC(C(C1O)(F)F)n1ccc(nc1=O)N
N#Cc1c(N2CC3C(C2)NCCO3)c(F)cc2c1n(cc(c2=O)C(=O)O)C1CC1
OC(=O)Cc1c(C)n(c2c1cccn2)Cc1ccc(cc1C(F)(F)F)S(=O)(=O)C
CC(NCC(C(=O)N1CCN(CC1)c1ncnc2c1C(C)CC2O)c1ccc(cc1)Cl)C

Continued on next page

Table S8 – *Continued from previous page*

Fc1ccc(cc1)c1noc(c1COc1ccc(cn1)C(=O)N1CCS(=O)(=O)CC1)C
Fc1cccc(c1)c1c(nc2n(c1=O)c(C)cs2)C(Nc1ncnc2c1[nH]cn2)C
CC(CCCC(CCCC(C)C)C)CCCC(=CCC1=C(C)C(=O)c2c(C1=O)cccc2)C
CCCCC(=O)N(C(C(=O)O)C(C)C)Cc1ccc(cc1)c1ccccc1c1nnn[nH]1
CC(=O)SC1CC2=CC(=O)CCC2(C2C1C1CCC3(C1(CC2)C)CCC(=O)O3)C
CCOc1nc2c(n1Cc1ccc(cc1)c1ccccc1c1nnn[nH]1)c(ccc2)C(=O)O
CCCc1c2oc(cc(=O)c2cc2c1n(CC)c(cc2=O)C(=O)[O-])C(=O)[O-]
CCOC(=O)C1=C(C)NC(=C(C1c1cccc(c1)[N+](=O)[O-])C(=O)OC)C
[O-]C(=O)CC(CC(C=Cc1c(c2ccc(cc2)F)c2c(n1C(C)C)cccc2)O)O
CCOc1ccc2c(c1C(=O)NC1C(=O)N3C1SC(C3C(=O)[O-])(C)C)cccc2
O=C1CCC2(C(=C1)C(=CC1C2CCC2(C1CCC2(OC(=O)C)C(=O)C)C)C)C
N#Cc1ccc(cc1C(F)(F)F)NC(=O)C(CS(=O)(=O)c1ccc(cc1)F)(O)C
C[N+](=C1C=CC(=C(c2ccc(cc2)N(C)C)c2ccc(cc2)N(C)C)C=C1)C
OC(=O)C1=CC2=CCC3C(C2(CC1)C)CCC1(C3CCC1C(=O)NC(C)(C)C)C
OCCC(=C(N(Cc1cnc(nc1N)C)C=O)C)SSCCC(SC(=O)C)CCCCC(=O)OC
O=C(C(Oc1ccc(cc1)Cl)(C)C)OCCCOC(=O)C(Oc1ccc(cc1)Cl)(C)C
O=C1C=CC2(C(=C1)C(C)CC1C2(F)C(O)CC2(C1CCC2(O)C(=O)C)C)C
CCCCOc1ccc(cc1)C[N+]1(C)C2CCC1CC(C2)OC(=O)C(c1ccccc1)CO
CCCC(=O)OC1(CCC2C1(C)CC(O)C1C2CCC2=CC(=O)CCC12C)C(=O)CO
OC(=O)CCC(C1CCC2C1(C)C(=O)CC1C2C(=O)CC2C1(C)CCC(=O)C2)C
N#CC1(CCC(CC1)N1CCC(C(C1)C)(C(=O)O)c1ccccc1)c1ccc(cc1)F
[O-]C(=O)C1N2C(=O)C(C2SC1(C)C)N1C(=O)C(NC1(C)C)c1ccccc1
Oc1ccc2c(c1)sc(c2C(=O)c1ccc(cc1)OCCN1CCCCC1)c1ccc(cc1)O
O=C(C(c1cscc1)C(=O)[O-])NC1C(=O)N2C1SC(C2C(=O)[O-])(C)C
CCCCN(CCC(c1cc2c(Cl)cc(cc2c2c1ccc(c2)C(F)(F)F)Cl)O)CCCC
FC(Oc1c(ccc2c1n(cc(c2=O)C(=O)O)C1CC1)c1ccc2c(c1)CNC2C)F
Oc1ccc2c(c1)OC(C(=C2C)c1ccc(cc1)O)c1ccc(cc1)OCCN1CCCCC1
O=C1CCC2(C(=C1)CCC1C2CCC2(C1CC1C2(OC(O1)(C)C)C(=O)C)C)C
Cc1nc2ccccn2c(=O)c1CCN1CCC(CC1)Nc1nc2c(n1Cc1ccco1)nccc2
Oc1ccc2c(c1)c(C)c(n2Cc1ccc(cc1)OCCN1CCCCCC1)c1ccc(cc1)O
N#CC(c1cccc(c1)Oc1ccccc1)OC(=O)C1C2(C1(C)C)C=Cc1c2cccc1
OCCN1CCN(CC1)c1cc(nc(n1)C)Nc1ncc(s1)C(=O)Nc1c(C)cccc1Cl
OC(=O)CNC(=O)c1ccccc1NS(=O)(=O)c1ccc(cc1)OC(=O)C(C)(C)C
O=C1NC(=O)C2(C1)C(=O)N(Cc1ccc(cc1F)Br)C(=O)c1c2cc(F)cc1
Clc1ccc(c(c1)Cl)C1(OCC(O1)COc1ccc(cc1)c1ccccc1)Cn1cncc1
O=C1[N-]C(=O)C(S1)Cc1ccc(c2c1scc2)OCCc1nc(oc1C)c1ccccc1
OCC=CC=CC=CC(CC(C(C=CC1CC=CC(=O)O1)(O)C)OP(=O)(O)[O-])O
O=C1C=C2C(=CC3C(C2(C2C1C2)C)CCC1(C3CC2C1(C2)C(=O)C)C)Cl
COc1ccc(cc1CNC1C2CCN(C1C(c1ccccc1)c1ccccc1)CC2)C(C)(C)C
CCOc1cc2ncc(c(c2cc1NC(=O)C=CCN(C)C)Nc1ccc(c(c1)Cl)F)C#N
OCC1OC(C(C1NC(=O)C(Cc1ccc(cc1)OC)N)O)n1cnc2c1ncnc2N(C)C
CCCC(=O)OC1(CCC2C1(C)CC(O)C1C2CCC2=CC(=O)CCC12C)C(=O)CC
CCCCOC(=O)NC(C(=O)OC)CNC(=O)CC1ON=C(C1)c1ccc(cc1)C(=N)N
COCOC(=O)C1N2C(=O)C(C2SC1(C)C)N1C(=O)C(NC1(C)C)c1ccccc1
CC[n+]1c(C=Cc2ccc(cc2)N2CCCC2)cccc1C=Cc1ccc(cc1)N1CCCC1
Cn1c(=O)n(C)c2c(c1=O)n(CCN1CCC(CC1)c1c[nH]c3c1cccc3)cn2
Cc1nc(C)c2c(n1)N(Cc1ccc(cc1)c1ccccc1c1[nH]nnn1)C(=O)CC2
CCCCCCCCCC(=O)CC(=O)OC1CCC2C1(C)CCC1C2CCC2=CC(=O)CCC12C
Cc1cc(ccc1Oc1ccc(cc1)SC(F)(F)F)n1c(=O)[nH]c(=O)n(c1=O)C
CCOP(=O)(NC(=S)Nc1ccccc1NC(=O)NS(=O)(=O)c1ccc(cc1)C)OCC
OCCN1CCN(CC1)c1cc(nc(n1)C)Nc1ncc(s1)C(=O)Nc1c(C)cccc1Cl
OCC(=O)C1(O)C(C)CC2C1(C)CC(O)C1C2C(Cl)CC2=CC(=O)C=CC12C
OCC(=O)C1(O)C(=C)CC2C1(C)CC(O)C1(C2CCC2=CC(=O)C=CC12C)F
O=C1CCC2(C(=C1)C(CC1C2CCC2(C1CCC2(O)C(=O)C)C)C(F)(F)F)C
OCC(=O)C1(O)C(C)CC2C1(C)CC(O)C1(C2CCC2=CC(=O)C=CC12C)Cl
ClCC(=O)C1(O)C(C)CC2C1(C)CC(O)C1(C2CCC2=CC(=O)C=CC12C)F
OCC(=O)C1C(C)CC2C1(C)CC(O)C1(C2CC(C2=CC(=O)C=CC12C)F)Cl
COC12CCC3(CC1C(O)(C)C)C14C2Oc2c4c(CC3N(CC1)CC1CC1)ccc2O
CCCC(C1CC23C=CC1(OC)C1C43CCN(C2Cc2c4c(O1)c(O)cc2)C)(O)C
CC[NH+](CCOC(=O)C1N2C(=O)C(C2SC1(C)C)NC(=O)Cc1ccccc1)CC
CC(=O)OCC(=O)C1(O)CCC2C1(C)CC(=O)C1C2CCC2=CC(=O)C=CC12C
NCC(=O)NCc1nc(nn1c1ccc(cc1C(=O)c1ccccc1Cl)Cl)C(=O)N(C)C
Fc1ccc2c(c1)C(=Cc1[nH]c(c(c1C)C(=O)NCCN1CCCC1)C)C(=O)N2
CCCCCCCCCC(=O)OCCN1CCN(CC1)CCC=C1c2ccccc2Sc2c1cc(Cl)cc2
O=C(C(c1cccs1)(c1cccs1)O)OC1C[N+]2(CCCOc3ccccc3)CCC1CC2
CCCC(=O)OCOC(=O)C1=C(C)NC(=C(C1c1cccc(c1Cl)Cl)C(=O)OC)C
CCOCCn1c(nc2c1cccc2)C1CCN(CC1)CCc1ccc(cc1)C(C(=O)O)(C)C
CCC(C(=O)OCOC(=O)C1=C(SC2CCS(=O)C2)SC2N1C(=O)C2C(O)C)CC
O=C1OC(CN1c1ccc(c(c1)F)c1ccc(nc1)c1nnn(n1)C)COP(=O)(O)O
CN(CC=CC(=O)Nc1cc2c(ncnc2cc1OC1COCC1)Nc1ccc(c(c1)Cl)F)C
CCC(COC(=O)C1NCC2C(C1)CC(CC2)Oc1cccc(c1c1[nH]nnn1)Cl)CC
CC(=O)c1c(C)c2cnc(nc2n(c1=O)C1CCCC1)Nc1ccc(cn1)N1CCNCC1
O=C(C1CCc2n1c(=O)ccn2)Nc1ccc(cc1)CC1CCC(N1)C(c1ccccc1)O
NCC(=C1CCCN(C1)c1c(F)cc2c(c1OC)n(cc(c2=O)C(=O)O)C1CC1)F
Fc1ccc(c(c1)F)CNC(=O)c1cn2CC3OCC(N3C(=O)c2c(c1=O)[O-])C
CCNC(=O)c1noc(c1c1ccc(cc1)CN1CCOCC1)c1cc(C(C)C)c(cc1O)O
OCC(C(=O)N(CC1CCCCC1)CCN1C2CCC1CC(C2)c1cccc(c1)C(=O)N)O
COc1c(OCCCN2CCOCC2)ccc2c1nc(NC(=O)c1cnc(nc1)N)n1c2=NCC1
O=C(C1CC1)Nc1nc2n(n1)c(ccc2)c1ccc(cc1)CN1CCS(=O)(=O)CC1
Nc1ccc(cn1)C#Cc1cnn2c1nc(cc2C(F)(F)F)c1ccc(cc1)C(F)(F)F
O=c1cnc2c3n1C(CN1CCC(CC1)NCc1ncc4c(c1)CCCO4)Cn3c(=O)cc2

Continued on next page

Table S8 – *Continued from previous page*

COc1cc(OC)cc(c1)N(c1ccc2c(c1)nc(cn2)c1cnn(c1)C)CCNC(C)C
COC(=O)c1cccc(c1)CCN(CCCC(c1ccc(c(c1)OC)OC)(C(C)C)C#N)C
CCCCCCCCCCCCCCCC(=O)OCC=C(C=CC=C(C=CC1=C(C)CCCC1(C)C)C)C
OCCN(c1nc(N2CCCCC2)c2c(n1)c(nc(n2)N(CCO)CCO)N1CCCCC1)CCO
COc1ccc(cc1C(=O)NCCc1ccc(cc1)S(=O)(=O)NC(=O)NC1CCCCC1)Cl
CCC(C(=O)OC1CC(C)C=C2C1C(CCC1CC(O)CC(=O)O1)C(C=C2)C)(C)C
CON=C(C(=O)NC1C(=O)N2C1SCC(=C2C(=O)[O-])COC(=O)N)c1ccco1
CC(=O)OCC(=O)C1(O)CCC2C1(C)CC(O)C1(C2CCC2=CC(=O)CCC12C)F
OC(=O)CCCCCCCCCOC(=O)C=C(CC1OCC(C(C1O)O)CC1OC1C(C(O)C)C)C
COc1ccc(cc1C12CC3CC(C2)CC(C1)C3)c1ccc2c(c1)ccc(c2)C(=O)O
Fc1ccc(cc1)Cn1c(nc2c1cccc2)N1CCC(CC1)N(c1nccc(=O)[nH]1)C
CC(CN1CCN(CCN(CCN(CC1)CC(=O)[O-])CC(=O)[O-])CC(=O)[O-])O
O=C1CCC2(C(=C1)C(=CC1C2CCC2(C1CCC2(OC(=O)C)C(=O)C)C)Cl)C
CCOC(=O)OC(OC1=C(C(=O)Nc2ccccn2)N(C)S(=O)(=O)c2c1cccc2)C
O=C(c1ccc(cc1)C(C)(C)C)CCCN1CCC(CC1)OC(c1ccccc1)c1ccccc1
Nc1cc(C)[n+](c2c1cccc2)CCCCCCCCCC[n+]1c(C)cc(c2c1cccc2)N
CCCCCCCCCCCCCCCCCCCCCC(=O)Nc1ccn(c(=O)n1)C1OC(C(C1O)O)CO
CCOC(=O)C(NC(C(=O)N(C1Cc2c(C1)cccc2)CC(=O)O)C)CCc1ccccc1
OC(COc1ccc(cc1)CCC(=O)OCC1COC(O1)(C)C)CNCCNC(=O)N1CCOCC1
CCCCCCC(COC(=O)c1cccnc1)(COC(=O)c1cccnc1)COC(=O)c1cccnc1
CCCc1ccc(c(c1)OC)OCC(=O)N1CCN(CC1)C(=O)COc1ccc(cc1OC)CCC
COc1ccc(cc1OC)CCN(CCCC(c1cc(OC)c(c(c1)OC)OC)(C(C)C)C#N)C
CCOC(=O)CNC(C(=O)N1CCC1C(=O)NCc1ccc(cc1)C(=N)NO)C1CCCCC1
CC(Cn1cnc2c1ncnc2N)OCP(=O)(OCOC(=O)OC(C)C)OCOC(=O)OC(C)C
CCC(=O)Oc1ccc2c(c1)CCC1C2CCC2(C1CC(C2OC(=O)CC)OC(=O)CC)C
O=C(C(c1ccccc1)C(=O)[O-])NC1C(=O)N2C1SC(C2C(=O)[O-])(C)C
OC(C(=O)NC1C(=O)N2C1SCC(=C2C(=O)[O-])CSc1nnnn1C)c1ccccc1
[O-]C(=O)C1N2C(=O)C(C2SC1(C)C)NC(=O)c1c(C)onc1c1ccccc1Cl
CCCCC(=O)OC1(CCC2C1(C)CC(O)C1C2CCC2=CC(=O)CCC12C)C(=O)CO
Fc1ccc(cc1)C(c1ccc(cc1)F)CCCN1CCC2(CC1)C(=O)NCN2c1ccccc1
[O-]C(=O)CCCC=CCC1C(O)CC(C1C=CC(COc1cccc(c1)C(F)(F)F)O)O
OCCn1c2nc(n(c2c(=O)n(c1=O)CC)Cc1ccc(c(c1)Br)OC)NC1CCCC1O
OCC(=O)C12OC(OC1CC1C2(C)CC(O)C2C1CCC1=CC(=O)C=CC21C)(C)C
O=C1CCC2(C(=C1)CCC1C2CCC2(C1CCC2C(=O)COC(=O)C(C)(C)C)C)C
[O-][N+](=Cc1ccc(cc1S(=O)(=O)[O-])S(=O)(=O)[O-])C(C)(C)C
Clc1ccc2c(c1)C(=C(CCS2(=O)=O)C(=O)Nc1ccc(c(c1)Cl)Cl)[O-]
CCC1(O)C(=O)OCc2c1cc1c3nc4cccc(c4cc3Cn1c2=O)[N+](=O)[O-]
NC(=O)C1=C(O)CC2C(C1=O)(O)C(=C1C(C2)Cc2c(C1=O)c(O)ccc2)O
OCC1(O)CC2OC1(C)n1c3c4n2c2ccccc2c4c2c(c3c3c1cccc3)CNC2=O
Cc1ccc(cc1)c1nc(c([nH]1)CN1CCN(CC1)C(c1ccccc1)c1ccccc1)C
COC1CCCC2C1=C(C(=O)OC(OC(=O)OC1CCCCC1)C)N1C2C(C1=O)C(O)C
O=C1[C-](C=NCCN=C[C-]2C(=O)C(OC2(C)C)(C)C)C(OC1(C)C)(C)C
OC(=O)C(=O)c1cn(c2c1cc(cc2)c1ccc(cc1)OC(F)(F)F)Cc1ccccc1
COc1ccc(cc1)C1=C(C(=O)c2ccc(cc2)OCCN2CCCC2)c2c(CC1)cccc2
COCCNC(=O)CN(CC(=O)O)CCN(CC(=O)O)CCN(CC(=O)NCCOC)CC(=O)O
OC(=O)C(Oc1ccc(cc1C)SCc1sc(nc1C)c1ccc(cc1F)C(F)(F)F)(C)C
OCC(n1cc(C(=O)O)c(=O)c2c1cc(OC)c(c2)Cc1cccc(c1F)Cl)C(C)C
CCC=C(c1csc(n1)N)C(=O)NC1C(=O)N2C1SCC(=C2C(=O)O)COC(=O)N
CON=C(c1csc(n1)N)C(=O)NC1C(=O)N2C1SCC(=C2C(=O)O)COC(=O)C
N=C(NC(=N)Nc1ccc(cc1)Cl)NCCCCCCNC(=N)NC(=N)Nc1ccc(cc1)Cl
ClCC(=O)C1(O)C(C)CC2C1(C)CC(=O)C1(C2CCC2=CC(=O)C=CC12C)F
Clc1ccc2c(c1)N(CCCN1CCC(CC1)(C(=O)N)N1CCCCC1)c1ccccc1CC2
OCCn1nnnc1SCC1=C(C(=O)O)N2C(OC1)C(C2=O)(OC)NC(=O)CSC(F)F
ClCC(=O)C1(O)C(C)CC2C1(C)CC(O)C1(C2CCC2=CC(=O)C=CC12C)Cl
Clc1ccc2c(c1)N(CCCN1CCC3(CC1)C(=O)NC1N3CCCC1)c1ccccc1CC2
OC1C(COP(=O)(OP(=O)(OP(=O)(O)O)O)O)OC(C1O)n1cnc2c1ncnc2N
OCCN(S(=O)(=O)C)c1cc2oc(c(c2cc1C1CC1)C(=O)NC)c1ccc(cc1)F
CCCCCCCC(=O)OCC(C(C1OC(=CC(C1NC(=O)C)NC(=N)N)C(=O)O)OC)O
OCC(Nc1nc(c2ccc(cc2C)F)c2c(n1)n(c(=O)cc2)c1c(F)cccc1F)CO
CCCn1c2nc([nH]c2c(=O)n(c1=O)CCC)C12CCC(CC1)(CC2)CCC(=O)O
OC(COc1cc(Cl)ccc1C#N)CNC(CNc1ccc(cc1)C1=NNC(=O)CC1C)(C)C
N#CC=Cc1cc(C)cc(c1)P(=O)(c1c([nH]c2c1cc(Cl)cc2)C(=O)N)OC
O=C(c1c[nH]c2c(c1=O)cccc2)Nc1cc(O)c(cc1C(C)(C)C)C(C)(C)C
Cc1ccc(c(c1)C(=O)N1CCN(CCC1C)c1oc2c(n1)cc(cc2)Cl)n1nccn1
Fc1ccc(c(c1)F)CNC(=O)c1cn2CC3OCCC(N3C(=O)c2c(c1=O)[O-])C
O=C(OC(COC(=O)CCCc1ccccc1)COC(=O)CCCc1ccccc1)CCCc1ccccc1
COc1cc(OC)cc(c1C=CS(=O)(=O)Cc1ccc(c(c1)NCC(=O)[O-])OC)OC
N#Cc1ccc(cc1)C(N1CCN(C(C1)c1ccc(cc1)Cl)c1ccc(cc1Cl)C#N)C
OCCn1ncc(c1)c1cnc(c2c1scc2c1ccc(cc1)NC(=O)Nc1cccc(c1)F)N
OC1CCC(CC1)N(c1cc(sc1C(=O)O)C#CC(C)(C)C)C(=O)C1CCC(CC1)C
N#Cc1cc(cc(c1)Cl)Oc1c(=O)n(ccc1C(F)(F)F)Cc1n[nH]c(=O)n1C
OC(=O)CC1CCC(CC1)c1ccc(cc1)c1ccc(cn1)Nc1ccc(nc1)C(F)(F)F
CCCC(C(C)(C)C)N(C(=O)c1cc(C)cc(c1)C)NC(=O)c1cccc(c1CC)OC
O=CN(CC(C(=O)NNc1nc(C)nc(c1F)N1CCN2C(C1)COCC2)CC1CCCC1)O
Fc1ccc(nc1)C(=O)Nc1ccc(c(c1)C1(C)N=C(N)N(S(=O)(=O)C1)C)F
NC(C(=O)NCCCN1CCN(CC1)CCCNC(=O)C(CCCNC(=N)N)N)CCCNC(=N)N
CC#CC(=O)N1CCCC1c1nc(c2n1ccnc2N)c1ccc(cc1)C(=O)Nc1ccccn1
COC(=O)CCNC(=O)C(C(COP(=O)(NC(C(=O)OC)C)Oc1ccccc1)(C)C)O
O=C(c1cc(c(cc1F)C)n1cnc(c1)C1CC1)Nc1cccc(n1)c1nncn1C(C)C
NC(=O)C1CCCN1C(=O)Nc1nc(c(s1)c1ccnc(c1)C(C(F)(F)F)(C)C)C
N#Cc1n(C)nc2c1c1cnc(c(c1)OC(C)c1c(C(=O)N(C2)C)ccc(c1)F)N

Continued on next page

NCC(C(=O)Nc1ccc2c(c1)ccnc2)c1ccc(cc1)COC(=O)c1ccc(cc1C)C
Nc1nc(cs1)C(=NOCC(=O)O)C(=O)NC1C2SCC(=C(N2C1=O)C(=O)O)C=C
O=C1NC(=O)C(S1)Cc1ccc(cc1)OCC1(C)CCc2c(O1)c(C)c(c(c2C)O)C
OC(c1ccc(cc1)C(C)(C)C)CCCN1CCC(CC1)C(c1ccccc1)(c1ccccc1)O
COC(=O)C1=C(C)NC(=C(C1c1ccccc1[N+](=O)[O-])C(=O)OCC(C)C)C
CCCCCCC(=O)OCCN1CCN(CC1)CCCN1c2ccccc2Sc2c1cc(cc2)C(F)(F)F
OCC1OC(OC2C(N)CC(C(C2O)OC2OC(CN)C(C(C2O)O)O)N)C(C(C1O)N)O
CCC(C(=O)OC1CC(O)C=C2C1C(CCC(CC(CC(=O)[O-])O)O)C(C=C2)C)C
CC(=O)OCC(=O)C1(O)CCC2C1(C)CC(O)C1C2CC(C2=CC(=O)C=CC12C)C
C=CCN1CC(CC2C1Cc1c[nH]c3c1c2ccc3)C(=O)N(C(=O)NCC)CCCN(C)C
COC(=O)C1=C(C)NC(=C(C1c1cccc(c1Cl)Cl)C(=O)OC(C)C)COC(=O)N
O=C(C(C)(C)C)OCOP(=O)(OCOC(=O)C(C)(C)C)COCCn1cnc2c1ncnc2N
CC(CCCC1(C)CCc2c(O1)c(C)c(c(c2C)OC(=O)C)C)CCCC(CCCC(C)C)C
Clc1ccc2c(c1)[nH]c(=O)n2C1CCN(CC1)CCCn1c(=O)[nH]c2c1cccc2
OC(=O)C1N2C(=O)C(C2SC1(C)C)NC(=O)c1c(C)onc1c1c(Cl)cccc1Cl
CCCCN(CC(c1cc(Cl)cc2c1c1ccc(cc1C2=Cc1ccc(cc1)Cl)Cl)O)CCCC
O=C(c1cc2c([nH]1)cccc2)NC1N=C(c2ccccc2)c2c(N(C1=O)C)cccc2
O=C1C(N=C(c2c(N1C)cccc2)c1ccccc1)NC(=O)c1cc2c3n1CCCc3ccc2
CCCc1c(OCCCCCOc2cc3OC(CCc3cc2C(=O)C)C(=O)O)ccc(c1O)C(=O)C
COCC1(OC)CCC2C1(C)CC(c1ccc(cc1)C=NO)C1=C3CCC(=O)C=C3CCC21
COc1cc2c(cc1OCCCN1CCN(CC1)C)ncc(c2Nc1cc(OC)c(cc1Cl)Cl)C#N
[O-]C(=O)CN1CCN(CCN(CCN(CC1)CC(=O)[O-])CC(=O)O)CC(=O)[O-]
CON=C(C(=O)NC1C(=O)N2C1SCC(=C2C(=O)O)CSc1cnns1)c1csc(n1)N
ONC(=O)C(C(C(=O)N1CCCCC1)CC1CCCC1)CN1C(=O)N(C(C1=O)(C)C)C
O=C1C=CC2(C(=C1)C(=CC1C2CCC2(C1CCC2(OC(=O)C)C(=O)C)C)Cl)C
OC1CC(O)C(=C)C(=CC=C2CCCC3(C2CCC3C(CCC(=O)N2CCCCC2)C)C)C1
OCC(=O)C1(O)C(C)CC2C1(C)CC(O)C1(C2CC(C2=CC(=O)C=CC12C)F)F
O=C1CCC2(C(=C1)CCC1C2(F)C(O)CC2(C1CCC2(OC(=O)C)C(=O)C)C)C
O=C(C(c1ccc(cc1)O)N=Cc1ccco1)NC1C(=O)N2C1SC(C2C(=O)O)(C)C
COc1ncccc1C(=O)NCCC1CCN(CC1)S(=O)(=O)NC(=O)NCC1CC2CC1C=C2
CC(=O)OCC(=O)C1(O)CCC2C1(C)CC(O)C1(C2CCC2=CC(=O)C=CC12C)F
CCOC(=O)C(N(S(=O)(=O)c1ccc(cc1)Cn1c(C)nc2c1ccnc2)C)CC(C)C
O=C(Nc1c(C)cccc1C)CN1CCN(CC1)CCCC(c1ccc(cc1)F)c1ccc(cc1)F
O=C1N(CC2CCCCC2CN2CCN(CC2)c2nsc3c2cccc3)C(=O)C2C1C1CCC2C1
O=C1CCC2C(=C1)CCC1C2CCC2(C1CCC2OC(=O)C12CCC(CC1)(C=C2)C)C
CCCc1nc(c(n1Cc1ccc(cc1)c1ccccc1c1nnn[nH]1)C(=O)O)C(O)(C)C
CN(c1ccc2c(c1)nn(c2C)C)c1ccnc(n1)Nc1ccc(c(c1)S(=O)(=O)N)C
CCCCC(=O)OC1(CCC2C1(C)CC(O)C1C2CCC2=CC(=O)C=CC12C)C(=O)CO
OCC(C(=O)O)NC(=O)Cc1c(C)n(c2c1cc(OC)cc2)C(=O)c1ccc(cc1)Cl
CNC(=O)c1nccc(c1)Oc1ccc(cc1)NC(=O)Nc1ccc(c(c1)C(F)(F)F)Cl
C=CCNc1nc(nc(n1)NCC=C)N1CCN(CC1)C(c1ccc(cc1)F)c1ccc(cc1)F
OCC1OC(OC2C(N)CC(C(C2O)OC2OC(CN)C(C(C2N)O)O)N)C(C(C1O)N)O
COCc1c(c2ccc(cc2)F)c(C=CC(CC(CC(=O)O)O)O)c(nc1C(C)C)C(C)C
OC(=O)CCC(=O)OCC(C(c1ccc(cc1)[N+](=O)[O-])O)NC(=O)C(Cl)Cl
N#CC(c1cccc(c1)Oc1ccccc1)OC(=O)C1C(C1(C)C)C=C(C(F)(F)F)Cl
O=C1C=CC2(C(=C1)C(F)CC1C2C(O)CC2(C1CC(C2C(=O)C(=O)O)C)C)C
CNC(C1CCC(C(O1)OC1C(N)CC(C(C1O)OC1OCC(C(C1O)NC)(C)O)N)N)C
O=C(c1ccc(cc1)OCCCCc1ccccc1)Nc1cccc2c1oc(cc2=O)c1[nH]nnn1
O=CN(C(=C(SC(=O)c1ccccc1)CCOC(=O)c1ccccc1)C)Cc1cnc(nc1N)C
OC1CN(C1)c1c(F)cc2c(c1Cl)n(cc(c2=O)C(=O)O)c1nc(N)c(cc1F)F
CC(=O)NCC1OC(=O)N(C1)c1ccc(c(c1)F)c1ccc(cc1)CNCc1cnn[nH]1
COCCOc1ccc(cc1)N1CCN(CC1)CCn1ncc2c1nc(N)n1c2nc(n1)c1ccco1
[O-]C(=O)CN(CCN(CC(=O)O)CC(=O)[O-])CCN(CC(=O)O)CC(=O)[O-]
CCCCCC(=O)OCC(=O)C1(O)CCC2C1(C)CC(O)C1C2CCC2=CC(=O)CCC12C
COc1ccc(cc1)C(=O)Nc1cccc(c1NC(=O)c1ccc(cc1)N1CCCN(CC1)C)O
OC(=O)COc1cccc(c1)CN(S(=O)(=O)c1cccnc1)Cc1ccc(cc1)n1cccn1
Fc1cc2CCOc2c(c1)C(CC(C(F)(F)F)(CNc1cccc2c1ccc(n2)C)O)(C)C
Clc1ccc(c(c1)F)Cc1c(C)nc2n1nc(Nc1n[nH]c(c1)C)cc2CN1CCOCC1
CC(c1ccccc1c1ccc2c(c1)nc([nH]2)C=Cc1ccc(cc1)C(F)(F)F)(O)C
O=C1CCC2(C3(C1)CC[N+](C2Cc1c3c(O)c(cc1)C(=O)N)(C)CC1CC1)O
COc1cc(OC)c(c(c1)OC)C=CS(=O)(=O)Cc1ccc(c(c1)OP(=O)(O)O)OC
COc1ccc(cc1)OCCCN(c1nc2c(o1)cccc2)Cc1cccc(c1)OC(C(=O)O)CC
FCCn1cc(C(=O)O)c(=O)c2c1c(OC)c(N1CC(C(C1)F)CNC1CC1)c(c2)F
C=CC(=O)Nc1cccc(c1)Oc1nc(Nc2ccc(cc2)N2CCN(CC2)C)nc2c1scc2
OCCn1cc(c(c1c1ccccc1C(F)(F)F)C)C(=O)Nc1ccc(cc1)S(=O)(=O)C
OC(=O)CNc1cccc(n1)CN(S(=O)(=O)c1cccnc1)Cc1ccc(cc1)n1cccn1
NCc1ccccc1CC(=O)NC1C(=O)N2C1SCC(=C2C(=O)O)CSc1nnnn1CC(=O)O
OCC(=O)C12OC(OC1CC1C2(C)CC(O)C2C1CC(C1=CC(=O)CCC21C)F)(C)C
Fc1ccc(cc1)C(c1ccc(cc1)F)CCCN1CCC(CC1)n1c(=O)[nH]c2c1cccc2
CC#CC1(O)CCC2C1(C)CC(c1ccc(cc1)N(C)C)C1=C3CCC(=O)C=C3CCC21
Cc1ccc(cc1)C(=O)Oc1cc(ccc1OC(=O)c1ccc(cc1)C)C(CNC(C)(C)C)O
Clc1ccc(cc1)C1(O)CCN(CC1)CCC(C(=O)N(C)C)(c1ccccc1)c1ccccc1
CCC1=C(CC2NCCc3c2cc(OC)c(c3)OC)CC2N(C1)CCc1c2cc(c(c1)OC)OC
CCOC(=O)OC(OC(=O)C1N2C(=O)C(C2SC1(C)C)NC(=O)C(c1ccccc1)N)C
CCCC1CN(C(C1)C(=O)NC(C1OC(SC)C(C(C1O)O)OP(=O)(O)O)C(Cl)C)C
COC(=O)C1CC2=CC(=O)CCC2(C23C1C1CCC4(C1(C)CC3O2)CCC(=O)O4)C
OCCN(CC(=O)N(C(Cc1ccccc1)(C)C)C)CC(=O)N(C(Cc1ccccc1)(C)C)C
COC(=O)C1=C(C)NC(=C(C1c1ccccc1[N+](=O)[O-])C(=O)OCC(=O)C)C
CON=C(c1csc(n1)N)C(=O)NC1C(=O)N2C1SCC(=C2C(=O)[O-])C1CCCO1
ClCOC(=O)C1(CCC2C1(C)CC(O)C1C2CCC2=CC(=O)C=CC12C)OC(=O)OCC
O=C(C(c1ccccc1)N)NC1C(=O)N2C1SC(C2C(=O)OCc1oc(=O)oc1C)(C)C

Continued on next page

Table S8 – *Continued from previous page*

CCCCCCCCCC(=O)OC1(CCN(CC1)CCCC(=O)c1ccc(cc1)F)c1ccc(cc1)Cl
CC(OC(=O)CCCC=CCC1C(O)CC(C1C=CC(COc1cccc(c1)C(F)(F)F)O)O)C
OC(=O)CCSC(c1cccc(c1)C=Cc1ccc2c(n1)cc(cc2)Cl)SCCC(=O)N(C)C
O=C(C(c1ccc(cc1)O)N)NC1C(=O)N2C1SCC(=C2C(=O)O)CSc1cnn[nH]1
CC(Cc1ccc(cc1)CCc1sc2c(c1)C(=NC(c1n2c(C)nn1)C)c1ccccc1Cl)C
Fc1ccc(cc1)C(=O)CCC[NH+]1CCC(CC1)(O)c1ccc(c(c1)C(F)(F)F)Cl
CCCCCCCCCCCCCCCCCCOP(=O)(OCC1OC(C(C1O)O)n1ccc(nc1=O)N)[O-]
CCNC(=O)C1OC(C(C1O)O)n1cnc2c1nc(C#CCC1CCC(CC1)C(=O)OC)nc2N
OC(CN1CCN(CC1)C(c1ccc(cc1)F)c1ccc(cc1)F)CSc1ncnc2c1[nH]cn2
Oc1ccc(cc1)C(C(=O)NC1C(=O)N2C1SCC(=C2C(=O)O)CSc1nnc(s1)C)N
O=C1C(NC(=O)c2c(C)onc2c2c(F)cccc2Cl)C2N1C(C(=O)O)C(S2)(C)C
COc1ccc(cc1)C(=O)COC(=O)C=C(C=CC=C(C=CC1=C(C)CCCC1(C)C)C)C
N#Cc1ccc(cc1)C(=O)N(c1ccccn1)CC(N1CCN(CC1)c1cccc2c1OCCO2)C
Fc1ccc(cc1)C(=C1CCN(CC1)CCc1c(C)nc2n(c1=O)ccs2)c1ccc(cc1)F
COc1ccc(cc1)C1(NC(=O)N(C1=O)CCCN1CCC(CC1)c1ccccc1)c1ccccc1
COCOC(=O)C1N2C(=O)C(C2SC1(C)C)N1C(=O)C(NC1(C)C)c1ccc(cc1)O
Clc1ccc(c(c1)Cl)C(Cn1cc[n+](c1)CCc1ccccc1)OCc1ccc(cc1Cl)Cl
CCCc1c(OCCCSc2ccc(c(c2CCC)O)C(=O)C)ccc(c1OCCCC(=O)O)C(=O)C
CC=CC1(O)CCC2C1(C)CC(c1ccc(cc1)N(C)C)C1=C3CCC(=O)C=C3CCC21
N=C(N1CCN(CC1)C(=N)NC(=N)Nc1ccc(cc1)Cl)NC(=N)Nc1ccc(cc1)Cl
OCCCOC1C(O)CC(=CC=C2CCCC3(C2CCC3C(CCCC(O)(C)C)C)C)C(=C)C1O
CON=C(C(=O)NC1C(=O)N2C1SCC(=C2C(=O)O)CSc1nnnn1C)c1csc(n1)N
COC1(NC(=O)CSCC(C(=O)O)N)C(=O)N2C1SCC(=C2C(=O)O)CSc1nnnn1C
FCSC(=O)C1(O)C(C)CC2C1(C)CC(O)C1(C2CC(C2=CC(=O)C=CC12C)F)F
OCC(=O)C1(O)CCC2C1(C)CC(O)C1(C2CC(C2=CC(=O)C(=CC12C)Br)F)F
CN1CCN(CC1)CC(=O)C1(O)CCC2C1(C)CC(O)C1C2CCC2=CC(=O)C=CC12C
OCC1Oc2ccc(cc2OC1c1ccc(c(c1)OC)O)C1Oc2cc(O)cc(c2C(=O)C1O)O
Cc1cccc(c1)c1ccc2c(c1)c(cn2Cc1ccc(cc1)C(C)(C)C)C(=O)C(=O)O
ClCC(=O)C1(O)C(C)CC2C1(C)CC(O)C1(C2CC(C2=CC(=O)C=CC12C)F)F
COc1ccc(c(c1)C(=O)Nc1ccc(cn1)Cl)NC(=O)c1ccc(cc1)C(=N)N(C)C
CC(OC(=O)C(N)C)COc1cn2c(c1C)c(ncn2)Oc1ccc2c(c1F)cc([nH]2)C
CN1CCN(CC1)c1ccc2c(c1)[nH]c(n2)c1c(=O)[nH]c2c(c1N)c(F)ccc2
COC1CC(N(C1)C(=O)Nc1ccc(cc1)Cl)C(=O)Nc1ccc(cc1F)n1ccccc1=O
CCNC1(CCN(CC1)c1ncnc2c1nc(n2c1ccc(cc1)Cl)c1ccccc1Cl)C(=O)N
OCCCC1(O)CCC2C1(C)CC(c1ccc(cc1)N(C)C)C1=C3CCC(=O)C=C3CCC21
OC(=O)COc1cccc(c1)CN(S(=O)(=O)c1cccnc1)Cc1ccc(cc1)C(C)(C)C
CCC(C(F)(F)F)(c1nnc(o1)NCc1ccc2c(c1)oc(=O)cc2c1ccc(cc1)F)O
O=C(N1CCN(CC1)C(=O)c1cc(ccc1F)Cc1n[nH]c(=O)c2c1cccc2)C1CC1
COc1nc2ccc(cc2cc1C(C(c1cccc2c1cccc2)(CCN(C)C)O)c1ccccc1)Br
COC1CN(CCCCCC(=O)OC2CN3CCC2CC3)CCC1NC(=O)c1cc(Cl)c(cc1OC)N
COC1CN(CCCCCC(=O)OC2CN3CCC2CC3)CCC1NC(=O)c1cc(Cl)c(cc1OC)N
O=C(NS(=O)(=O)C)COCCCCN(c1cnc(c(n1)c1ccccc1)c1ccccc1)C(C)C
N#Cc1nn(c(c1C1(CC1(F)F)C(F)(F)F)N)c1c(Cl)cc(cc1Cl)C(F)(F)F
O=C(N1CCN(CC1)Cc1sc2c(c1C)nc(nc2N1CCOCC1)c1cnc(nc1)N)C(O)C
CCCN(C(=O)C1=Cc2ccc(cc2N=C(C1)N)c1ccc(cc1)C(=O)N1CCCC1)CCC
OC(=O)COc1cc2onc(c2cc1C)CCc1sc(nc1C(C)C)c1ccc(cc1)C(F)(F)F
CC#Cc1cncc(c1)c1ccc2c(c1)C1(N=C(C(=N1)C)N)C1(C2)CCC(CC1)OC
CCOC(=O)C(NC(C(=O)N1Cc2cc(OC)c(cc2CC1C(=O)O)OC)C)CCc1ccccc1
O=COC(C(=O)NC1C(=O)N2C1SCC(=C2C(=O)[O-])CSc1nnnn1C)c1ccccc1
CON=C(c1csc(n1)N)C(=O)NC1C(=O)N2C1SCC(=C2C(=O)[O-])COC(=O)C
OCC(=O)C12OC(OC1CC1C2(C)CC(O)C2(C1CCC1=CC(=O)C=CC21C)F)(C)C
ClCC(=O)C12OC(OC1CC1C2(C)CC(O)C2(C1CCC1=CC(=O)CCC21C)F)(C)C
CC(=O)OC1(CCC2C1(C)CCC1C2C=C(C2=CC(=O)C3C(C12C)C3)Cl)C(=O)C
OCCn1nnnc1SCC1=C(C(=O)[O-])N2C(OC1)C(C2=O)(OC)CC(=O)CSC(F)F
[O-]S(=O)(=O)Oc1ccc(cc1)C(c1ccccn1)c1ccc(cc1)OS(=O)(=O)[O-]
O=C(c1cc2c([nH]1)cccc2)NC1N=C(c2ccccc2F)c2c3N(C1=O)CCc3ccc2
CCCc1nn(c2c1nc([nH]c2=O)c1cc(ccc1OCC)S(=O)(=O)N1CCN(CC1)C)C
O=C(C(c1ccccc1)NC(=O)N1CCNC1=O)NC1C(=O)N2C1SC(C2C(=O)O)(C)C
CCC1OC(CC=C1C)C(=CC(C=CC1C(C1C)C=CC1OC(CC(=O)O)CC(C1O)O)C)C
CCOC(=O)C(=O)Nc1cc(C)c(c(c1)C)Oc1ccc(c(c1)C(c1ccc(cc1)F)O)O
O=C1CCC2C(=C1)CCC1C2CCC2(C1CCC2OC(=O)C12CC3CC(C2)CC(C1)C3)C
COc1ccc(cc1)n1nc(c2c1C(=O)N(CC2)c1ccc(cc1)N1CCCCC1=O)C(=O)N
O=C1CCC2(C(=C1)C(=CC1C2CCC2(C1CC(C2(OC(=O)C)C(=O)C)C)C)Cl)C
CC(OC(=O)C1CC2=CC(=O)CCC2(C2C1C1CCC3(C1(CC2)C)CCC(=O)O3)C)C
OCC(=O)C12OC(OC1CC1C2(C)CC(O)C2(C1CCC1C2(C)CCC(=O)C1)F)(C)C
Fc1ccc(cc1)C(=O)C1CCN(CC1)CCCN1c2ccccc2Sc2c1cc(cc2)C(F)(F)F
CCCc1c(OCCCOc2cc(O)c(cc2CC)c2ccc(cc2)F)cccc1Oc1ccccc1C(=O)O
CCCCOC(=O)C(=O)C1C(C)CC2C1(C)CC(O)C1C2CC(C2=CC(=O)C=CC12C)F
CNC(=O)CN(CC(=O)[O-])CCN(CC(=O)[O-])CCN(CC(=O)NC)CC(=O)[O-]
CON=C(c1csc(n1)N)C(=O)NC1C(C)N(C1=O)OCC(=O)OCC(=O)OC(C)(C)C
CCOC(=O)C1=C(C)NC(=C(C1c1ccccc1C=CC(=O)OC(C)(C)C)C(=O)OCC)C
Fc1ccc(c(c1)C(=O)Nc1ccc(c(c1)Cl)C(=O)N1Cc2cccn2Cc2c1cccc2)C
O=C1CCC2(C(=C1)C(=CC1C2CCC2(C1CC(=C)C2(OC(=O)C)C(=O)C)C)C)C
COc1ccc(cc1)C(=C(c1ccccc1)[N+](=O)[O-])c1ccc(cc1)OCCN1CCCC1
FC(C(C(C(C(C(C(C(Br)(F)F)(F)F)(F)F)(F)F)(F)F)(F)F)(F)F)(F)F
O=C(OC1=C(C(=O)Nc2ccccn2)N(C)S(=O)(=O)c2c1cccc2)C=Cc1ccccc1
CN(C1C(=C(C(=O)N)C(=O)C2(C1CC1Cc3cccc(c3C(=O)C1=C2O)O)O)O)C
Fc1ccc(cc1)CNC(=O)c1nc(n(c(=O)c1O)C)C(NC(=O)c1nnc(o1)C)(C)C
Cc1c(c2ccccc2)n(c2[n+]1cccc2)N=Nn1c2cccc[n+]2c(c1c1ccccc1)C
Clc1ccc(cc1)OC(C(=O)OC(COC(=O)c1cccnc1)COC(=O)c1cccnc1)(C)C
NC(=O)OCC1C(NC(=O)C(=NOCC(=O)O)c2csc(n2)N)C(=O)N1S(=O)(=O)O

Continued on next page

Table S8 – *Continued from previous page*

CON=C(C(=O)NC1C(=O)N2C1SCC(=C2C(=O)O)Cn1nnc(n1)C)c1csc(n1)N
CC(=O)OCC(=O)C1(O)CCC2C1(C)CC(Cl)C1(C2CCC2=CC(=O)C=CC12C)Cl
O=C(NC(=O)c1c(F)cccc1F)Nc1ccc(c(c1)Oc1ncc(cc1Cl)C(F)(F)F)Cl
CN1CCN(CC1)c1nc(Sc2ccc(cc2)NC(=O)C2CC2)nc(c1)Nc1n[nH]c(c1)C
COC1(NC(=O)C(c2cscc2)C(=O)[O-])C(=O)N2C1SC(C2C(=O)[O-])(C)C
CCOc1cc(ccc1OC)C(N1C(=O)c2c(C1=O)c(ccc2)NC(=O)C)CS(=O)(=O)C
CCOc1nc2c(n1Cc1ccc(cc1)c1ccccc1c1noc(=O)[nH]1)c(ccc2)C(=O)O
CCOC(=O)C(CCC(=O)NC(C(=O)NC(c1ccccc1)C(=O)OCC)CSCc1ccccc1)N
CN1CCN(CC1)CCOc1cc(OC2CCOCC2)c2c(c1)ncnc2Nc1c(Cl)ccc2c1OCO2
CN1CCN(CC1)c1nc2ccccc2c(n1)C1=C(C(=O)NC1=O)c1c[nH]c2c1cccc2
Brc1ccc(cc1)c1ccc(cc1)C1Cc2ccccc2C(C1)c1c(=O)sc2c(c1O)cccc2
Clc1ccc2c(c1)ccc(c2)S(=O)(=O)CC(C(=O)N1CCC(CC1)N1CCCNC1=O)O
CN1CCN(CC1)Cc1ccc(cc1)C(=O)Nc1ccc(c(c1)Nc1scc(n1)c1cccnc1)C
Clc1ccc(c(c1)C(=O)c1ccn(=O)cc1)NS(=O)(=O)c1ccc(cc1)C(C)(C)C
CC(=CCCC(CCC1=C(C)C(=O)C(=C(C1=O)C)C)(O)C)CCC=C(CCC=C(C)C)C
OC(CCc1ccccc1)CCC1C(O)CC(C1CC=CCCCC(=O)OCCCCO[N+](=O)[O-])O
[O-]C(=O)CC1CCC(CC1)c1ccc(cc1)c1ccc(cn1)Nc1ccc(nc1)C(F)(F)F
CCOC(=O)C(NP(=O)(NC(C(=O)OCC)C)COCCn1cnc2c1nc(N)nc2NC1CC1)C
Oc1cc(cc(c1O)[N+](=O)[O-])c1onc(n1)c1c(C)c(Cl)c(n(=O)c1Cl)C
CC(CNc1nc(Nc2ccnc(c2)C(F)(F)F)nc(n1)c1cccc(n1)C(F)(F)F)(O)C
Fc1cc(F)c(c(c1)F)CNC(=O)c1cn2CC3OC4CCC(N3C(=O)c2c(c1=O)O)C4
O=C(CC1N=C(c2ccc(cc2)Cl)c2c(n3c1nnc3C)sc(c2C)C)Nc1ccc(cc1)O
OC(=O)CC1CCc2c1[nH]c1c2cc(cc1)OCc1ccc(c(c1)C(F)(F)F)C1CCCC1
OC(=O)CCC(C(=O)O)NC(=O)c1ccc(cc1)N(Cc1cnc2c(n1)c(N)nc(n2)N)C
COCc1c(c2ccc(cc2)F)c(C=CC(CC(CC(=O)[O-])O)O)c(nc1C(C)C)C(C)C
CC(CN(S(=O)(=O)c1ccc(cc1)N)CC(C(Cc1ccccc1)NC(=O)OC1CCOC1)O)C
CC(Nc1cccnc1N1CCN(CC1)C(=O)c1cc2c([nH]1)ccc(c2)NS(=O)(=O)C)C
CC(=O)OCC(=O)C1(O)C(C)CC2C1(C)CC(O)C1C2CC(C2=CC(=O)C=CC12C)F
OCC(C(C(=O)NCCC(=O)NCCSSCCNC(=O)CCNC(=O)C(C(CO)(C)C)O)O)(C)C
CCC(C(=O)O)(Oc1ccc(cc1)C1(CCCCC1)c1ccc(cc1)OC(C(=O)O)(CC)C)C
CC(=O)OCC(=O)C1(O)C(C)CC2C1(C)CC(O)C1(C2CCC2=CC(=O)C=CC12C)F
CN1CCN(CC1)Cc1ccc(cc1)C(=O)Nc1ccc(c(c1)Nc1nccc(n1)c1cccnc1)C
OC(=O)CCC(=O)OCC(=O)C1(O)CCC2C1(C)CC(O)C1C2CCC2=CC(=O)CCC12C
O=C(C(S(=O)(=O)[O-])c1ccccc1)NC1C(=O)N2C1SC(C2C(=O)[O-])(C)C
CON=C(C(=O)NC1C(=O)N2C1SCC(=C2C(=O)[O-])CSc1cnns1)c1csc(n1)N
COC(=O)C1=C(C#N)NC(=C(C1c1cccc(c1)[N+](=O)[O-])C(=O)OC(C)C)C
ON=C(c1csc(n1)N)C(=O)NC1C(=O)N2C1SCC(=C2C(=O)O)SCSc1cnn[nH]1
CC(=O)OC1CC2C(C1OC(=O)C)(C)CCC1C2CCc2c1ccc(c2)OC(=O)c1ccccc1
[O-]C(=O)C1N2C(=O)C(C2SC1(C)C)NC(=O)c1c(C)onc1c1c(Cl)cccc1Cl
[O-]C(=O)CCC(=O)OCC(C(c1ccc(cc1)[N+](=O)[O-])O)NC(=O)C(Cl)Cl
COc1ccc(cc1C(NC(=O)Cc1ccc(cc1)S(=O)(=O)Nc1ncc(cn1)CC(C)C)C)F
COc1ccc(cc1NC(=O)Cc1ccc(cc1)S(=O)(=O)[N-]c1ncc(cn1)CC(C)C)Cl
O=C(C(c1ccccc1)N)NC1C(=O)N2C1SC(C2C(=O)OCOC(=O)C(C)(C)C)(C)C
NC(=N)NN=C(C=Cc1ccc(o1)[N+](=O)[O-])C=Cc1ccc(o1)[N+](=O)[O-]
CCCc1c(OCCCCCOc2cc3OC(CCc3cc2C(=O)C)C(=O)[O-])ccc(c1O)C(=O)C
CC(=O)OC1C=COC=C2C1N1C(=O)C34SSC1(C2)C(=O)N4C1C(=COC=CC1O)C3
C=CC1(C)CC(=O)C2(C(O1)(C)C(OC(=O)C)C(C1C2(C)C(O)CCC1(C)C)O)O
CC(=O)OCC(=O)C12N=C(OC1CC1C2(C)CC(O)C2C1CCC1=CC(=O)C=CC21C)C
[O-][N+](=O)OC(C(O[N+](=O)[O-])CO[N+](=O)[O-])CO[N+](=O)[O-]
Fc1ccc2c(c1)[nH]c(=O)n2CCCN1CCC(CC1)n1c(=O)[nH]c2c1ccc(c2)Cl
CC(=O)OC(C(=O)C1(O)CCC2C1(C)CC(O)C1(C2CCC2=CC(=O)C=CC12C)F)C
CCCCC(=O)OC1(CCC2C1(C)CC(O)C1C2CC(C2=CC(=O)C=CC12C)F)C(=O)CO
OC1CN(CC1O)C(=O)C(C(NC(=O)c1cc2c([nH]1)ccc(c2)Cl)Cc1ccccc1)O
OC(=O)COC1CCN(CC1)C(=O)C(NC(=O)c1ccc(cc1)C(=N)N)Cc1ccc(cc1)O
COc1ccc(cc1)OC(=O)N(Cc1ccc(cc1)OCCc1nc(oc1C)c1ccccc1)CC(=O)O
OCC(=O)C12Cc3ccccc3CC1CC1C2(C)CC(O)C2(C1CCC1=CC(=O)C=CC21C)F
CSC(=O)C1(OC(=O)C)C(C)CC2C1(C)CC(O)C1(C2CCC2=CC(=O)C=CC12C)F
CCOP(=O)(N=C(SCc1ccccc1)SCCSC(=NP(=O)(OCC)OCC)SCc1ccccc1)OCC
CCOCc1cc(OC)c(c(c1)OC)c1ccc(cc1)CC(C(=O)O)NC(=O)c1c(F)cccc1F
CCC(=O)OC1(CCC2C1(C)CC(O)C1C2CCC2=CC(=O)CCC12C)C(=O)COC(=O)C
OC(C(C(OS(=O)(=O)C)COS(=O)(=O)C)O)C(OS(=O)(=O)C)COS(=O)(=O)C
CN(CCn1nnnc1SCC1=C(C(=O)O)N2C(SC1)C(C2=O)NC(=O)Cc1csc(n1)N)C
OC(COc1cccc2c1c(=O)cc(o2)C(=O)O)COc1cccc2c1c(=O)cc(o2)C(=O)O
O=C(NC(=O)c1c(F)cccc1F)Nc1cc(Cl)c(cc1Cl)OC(C(C(F)(F)F)F)(F)F
CC1CCC2(OC1)OC1C(C2C)C2(C(C1)C1CC=C3C(C1CC2)(C)C(O)CC(C3)O)C
CCCSc1nc(NC2CC2c2ccc(c(c2)F)F)c2c(n1)n(nn2)C1CC(C(C1O)O)OCCO
CNC(=NS(=O)(=O)c1ccc(cc1)Cl)N1N=C(C(C1)c1ccccc1)c1ccc(cc1)Cl
O=C1CCC2=C3C(CC4(C(C3CCC2=C1)CCC4(O)C(=O)C)C)c1ccc(cc1)N(C)C
O=C(c1ccc(cc1)Cc1c(=O)oc2c(c1O)cccc2)OC(C(F)(F)F)(C(F)(F)F)C
N#Cc1ccc2c(c1)c(CCCCN1CCN(CC1)c1ccc3c(c1)cc(o3)C(=O)N)c[nH]2
OC(=O)CCN(C(=O)c1ccc2c(c1)nc(n2C)CNc1ccc(cc1)C(=N)N)c1ccccn1
CC1OC(C)CN(C1)c1ccc(cn1)NC(=O)c1cccc(c1C)c1ccc(cc1)OC(F)(F)F
CNC(=O)c1nccc(c1)Oc1ccc(c(c1)F)NC(=O)Nc1ccc(c(c1)C(F)(F)F)Cl
O=C(CN(c1c(C)cccc1C)C(=O)C1CCS(=O)(=O)CC1)Nc1ccc(cc1)c1nocn1
CCc1nc2c(n1c1ccc(cc1)CCNC(=O)NS(=O)(=O)c1ccc(cc1)C)cc(nc2C)C
Fc1ccc(cc1)c1nc([nH]c1c1ccc2c(n1)n(CC(C)(C)C)c(n2)N)C(C)(C)C
COc1ncc(cc1NS(=O)(=O)c1ccc(cc1F)F)c1ccc2c(c1)c(ccn2)c1ccnnc1
OC(=O)CCC(C(=O)O)NC(=O)c1ccc(cc1)NCc1cnc2c(n1)c(=O)[nH]c(n2)N
CCCC1OC2C(O1)(C(=O)CO)C1(C(C2)C2CCC3=CC(=O)C=CC3(C2C(C1)O)C)C
COCCOC(=O)C1=C(C)NC(=C(C1c1cccc(c1)[N+](=O)[O-])C(=O)OC(C)C)C
O=C(OCC(=O)C1(O)CCC2C1(C)CC(O)C1C2CCC2=CC(=O)CCC12C)CCC1CCCC1

Continued on next page

O=c1oc(c(o1)CN1CCN(CC1)c1cc2c(cc1F)c(=O)c(c1n2C(C)S1)C(=O)O)C
O=C1C(NC(=O)c2c(C)onc2c2c(F)cccc2Cl)C2N1C(C(=O)[O-])C(S2)(C)C
[O-][N+](=O)OCC(CO[N+](=O)[O-])(CO[N+](=O)[O-])CO[N+](=O)[O-]
OC(=O)CCC(=O)OCC(=O)C1(O)CCC2C1(C)CC(O)C1C2CCC2=CC(=O)C=CC12C
O=C(C(C(=O)Oc1ccccc1)c1ccccc1)NC1C(=O)N2C1SC(C2C(=O)[O-])(C)C
CC[N+](CCOc1c(OCC[N+](CC)(CC)CC)cccc1OCC[N+](CC)(CC)CC)(CC)CC
OCC(=O)C12OC(OC1CC1C2(C)CC(O)C2(C1CCC1=CC(=O)C=CC21C)F)(CC)CC
CCCCN(C(=O)CN1CC(C(C1c1ccc(cc1)OC)C(=O)O)c1ccc2c(c1)OCO2)CCCC
CC(=O)OCC(=O)C1C(C)CC2C1(C)CC(O)C1(C2CC(C2=CC(=O)C=CC12C)F)Cl
CC(=O)OC1CCC2(C(=C1)C(=CC1C2CCC2(C1CCC2(OC(=O)C)C(=O)C)C)Cl)C
Clc1ccc(cc1)C(c1cncn1C)(c1ccc2c(c1)c(cc(=O)n2C)c1cccc(c1)Cl)N
CCCCc1c(c2ccc3c(c2)ccc(c3)OCc2nnn[nH]2)n(c2c1cccc2)Cc1ccccc1
CCC(c1ccc(cc1)C(=O)NC(C(=O)O)CCC(=O)O)Cc1cnc2c(n1)c(N)nc(n2)N
CCOC(=O)C1(CCC2C1(C)CC(O)C1C2CCC2=CC(=O)C=CC12C)OC(=O)C(Cl)Cl
CCCCCC(=O)OCC(=O)C1C(C)CC2C1(C)CC(O)C1C2CC(C2=CC(=O)C=CC12C)F
O=C1C=CC2(C(=C1)C(C)CC1C2(F)C(O)CC2(C1CCC2(OC(=O)C)C(=O)C)C)C
CCCCCCCC(c1cc(OC(=O)CCCN(CC)CC)c2c(c1)OC(C1=C2CC(C)CC1)(C)C)C
Clc1ccc2c(n1)nc(cc2)N1C(CC(=O)N2CCC3(CC2)OCCO3)c2c(C1=O)cccc2
O=C(C(c1ccccc1)NS(=O)(=O)[O-])NC1C(=O)N2C1SC(C2C(=O)[O-])(C)C
CN(C1CCC2(C34C1Oc1c4c(CC2N(CC3)CC2CC2)ccc1O)O)C(=O)C=Cc1ccoc1
OCC(=O)C1(O)C(C)CC2C1(C)CC(O)C1(C2CC(C2=CC(=O)C(=CC12C)Cl)F)F
CCCC(=O)C1=C(O)C(C(=C(C1=O)Cc1c(OC)cc(c(c1O)C(=O)CCC)O)O)(C)C
CCOc1c2C(OC(=O)c2c(c(c1OCC)OCC)N)C1N(C)CCc2c1c(OC)c1c(c2)OCO1
OCC(C(N1CCN(CCN(CCN(CC1)CC(=O)[O-])CC(=O)[O-])CC(=O)[O-])CO)O
OC(=O)CCC(C(=O)O)NC(=O)c1ccc(cc1)CCc1c[nH]c2c1c(=O)nc([nH]2)N
OC(=O)CCC(=O)OC1CC2C(C1OC(=O)CCC(=O)O)(C)CCC1C2CCc2c1ccc(c2)O
CCCCCCCCCC(=O)OCCN1CCN(CC1)CCC=C1c2ccccc2Sc2c1cc(cc2)C(F)(F)F
CC(=O)OCC(=O)C1(O)C(=C)CC2C1(C)CC(O)C1(C2CCC2=CC(=O)C=CC12C)F
O=C1C(NC(=O)c2c(C)onc2c2c(F)cccc2Cl)C2N1C(C(=O)[O-])C(S2)(C)C
OC(CC(CC(=O)O)O)C=Cc1c(nc(nc1c1ccc(cc1)F)N(S(=O)(=O)C)C)C(C)C
[O-]C(=O)COc1cccc(c1)CN(S(=O)(=O)c1cccnc1)Cc1ccc(cc1)C(C)(C)C
CCOC(=O)NC1CCC2C(C1)CC1C(C2C=Cc2ccc(cn2)c2cccc(c2)F)C(OC1=O)C
COc1cc2c(ccnc2cc1OC)Oc1ccc(cc1)NC(=O)C1(CC1)C(=O)Nc1ccc(cc1)F
Nc1nccc(n1)c1sc(nc1c1cccc(c1F)NS(=O)(=O)c1c(F)cccc1F)C(C)(C)C
CCCCCC(=O)OCN1C(=O)CCc2c1cc(OCCCCN1CCN(CC1)c1cccc(c1Cl)Cl)cc2
NC(CN1CC(F)(F)CCC1=O)CC(=O)N1CCc2c(C1)nc(nc2C(F)(F)F)C(F)(F)F
CCCCc1nc(C)c(c(=O)n1Cc1ccc(cc1)c1ccccc1c1nnn[nH]1)CC(=S)N(C)C
O=C(c1ccc2c(c1)CCC1C2(CCC(C1)(O)C(F)(F)F)Cc1ccccc1)Nc1cccnc1C
Cc1cnc(nc1Nc1cccc(c1)S(=O)(=O)NC(C)(C)C)Nc1ccc(cc1)OCCN1CCCC1
C=CC(=O)Nc1cc(Nc2nccc(n2)c2cn(c3c2cccc3)C)c(cc1N(CCN(C)C)C)OC
OC1C(COP(=O)(OP(=O)(OCC[N+](C)(C)C)[O-])O)OC(C1O)n1ccc(nc1=O)N
NC(=N)NCCCC(C(=O)N1CCC(CC1C(=O)O)C)NS(=O)(=O)c1cccc2c1NCC(C2)C
CCC1=C(C)CN(C1=O)C(=O)NCCc1ccc(cc1)S(=O)(=O)NC(=O)NC1CCC(CC1)C
CCCc1nc2c(n1Cc1ccc(cc1)c1ccccc1C(=O)O)cc(cc2C)c1nc2c(n1C)cccc2
OC(=O)C(c1ccc(cc1)C(CCCN1CCC(CC1)C(c1ccccc1)(c1ccccc1)O)O)(C)C
COC12CCC3(CC1C(C(C)(C)C)(O)C)C14C2Oc2c4c(CC3N(CC1)CC1CC1)ccc2O
Clc1ccc(c(c1)Cl)C1(OCC(O1)COc1ccc(cc1)N1CCN(CC1)C(C)C)Cn1cncn1
O=C(CC(C)(C)C)OCC(=O)C1(O)CCC2C1(C)CC(O)C1C2CCC2=CC(=O)C=CC12C
CC[N+](Cc1ccccc1Cl)(CCNC(=O)C(=O)NCC[N+](Cc1ccccc1Cl)(CC)CC)CC
COCCOCC(C(=O)Oc1ccc2c(c1)CCC2)CC1(CCCC1)C(=O)NC1CCC(CC1)C(=O)O
COCC(=O)C1(OC(=O)CC)C(C)CC2C1(C)CC(O)C1(C2CCC2=CC(=O)C=CC12C)F
CON=C(c1csc(n1)N)C(=O)NC1C(=O)N2C1SCC(=C2C(=O)O)C[N+]1(C)CCCC1
CCC(=O)OC1(C(C)CC2C1(C)CC(O)C1(C2CCC2=CC(=O)C=CC12C)F)C(=O)CCl
OCC(=O)C12OC(OC1CC1C2(C)CC(O)C2(C1CC(C1=CC(=O)C=CC21C)F)F)(C)C
CCC(=C(c1ccc(cc1)OP(=O)(O)O)c1ccc(cc1)OCCN(C)C)c1ccc(cc1)C(C)C
CCCC(=O)OC1(CCC2C1(C)CC(O)C1C2CCC2=CC(=O)CCC12C)C(=O)COC(=O)CC
O=C(C(c1ccccc1)N)NC1C(=O)N2C1SC(C2C(=O)OC1OC(=O)c2c1cccc2)(C)C
CCCCc1oc2c(c1C(=O)c1ccc(cc1)OCCCN(CCCC)CCCC)cc(cc2)NS(=O)(=O)C
Fc1ccc(cc1)C(c1ccc(cc1)F)CCCN1CCC(CC1)n1c(=O)[nH]c2c1ccc(c2)Cl
O=C1CCC2(C(=C1)CCC1C2CCC2(C1CC1C2(OC(O1)(C)c1ccccc1)C(=O)C)C)C
CN(C1C(=C(C(=O)N)C(=O)C2(C1CC1Cc3ccc(c(c3C(=O)C1=C2O)O)N)O)O)C
O=C(C(c1ccccc1)NC(=O)N1CCNC1=O)NC1C(=O)N2C1SC(C2C(=O)[O-])(C)C
OCCC(=C(N(Cc1cnc(nc1N)C)C=O)C)SSC(=C(N(Cc1cnc(nc1N)C)C=O)C)CCO
CON=C(c1csc(n1)N)C(=O)NC1C(=O)N2C1SCC(=C2C(=O)O)CSC(=O)c1ccco1
CN(C1C(=C(C(=O)N)C(=O)C2(C1CC1C(O)c3cccc(c3C(=O)C1=C2O)O)O)O)C
O=C1C=CC2(C(=C1)CCC1C2(F)C(O)CC2(C1CC1C2(OC(O1)(C)C)C(=O)C)C)C
O=C(Nc1cc(nn1c1ccc(cc1)C)C(C)(C)C)Nc1ccc(c2c1cccc2)OCCN1CCOCC1
OC(=O)C(Oc1c(C)cc(cc1C)c1c2ccccc2c(c2c1c(C)c(s2)C)Br)Cc1ccccc1
CCOC(=O)C1=C(C)N(CCN2CCOCC2)C(=C(C1c1ccccc1C(F)(F)F)C(=O)OCC)C
NCCCN(C(c1nc2cc(Cl)ccc2c(=O)n1Cc1ccccc1)C(C)C)C(=O)c1ccc(cc1)C
COc1cc2c(cc1OCCCN1CCCCC1)ncnc2N1CCN(CC1)C(=O)Nc1ccc(cc1)OC(C)C
O=C1CCC2(C(=C1)CCC1C2C(O)CC2(C1CCC2(O)C(=O)CSC(=O)C(C)(C)C)C)C
O=C(C1CCC(CC1)(F)F)NC(c1ccccc1)CCN1C2CCC1CC(C2)n1c(C)nnc1C(C)C
Fc1ccc(cc1)CNC(=O)c1nc(n(c(=O)c1[O-])C)C(NC(=O)c1nnc(o1)C)(C)C
O=C(c1cccnc1)OC1C=CC2C34C1Oc1c4c(CC2N(CC3)C)ccc1OC(=O)c1cccnc1
CCC(=O)n1c(=O)n(c2c1cccc2)C1CCN(CC1)CCC(c1ccccc1)(c1ccccc1)C#N
OC(=O)CCCCN(Cc1ccc(cc1)C(=O)O)CCc1ccccc1OCc1ccc(cc1)CCc1ccccc1
O=C(NC1C(=O)N2C1SCC(=C2C(=O)[O-])C[n+]1ccc(cc1)C(=O)N)Cc1cccs1
CCN(CC(C[N+]12C(O)C(CC)C3C4C1CC1(C(C2C3)N(C)c2c1cccc2)C4O)O)CC
COCC(=O)OC1(CCN(CCCc2nc3c([nH]2)cccc3)C)CCc2c(C1C(C)C)ccc(c2)F
CC(OC(=O)C1=CN(CC(c2c1[nH]c1c2cccc1)(C)C)C(=O)c1ccc(c(c1)F)F)C

Continued on next page

OC1C(COP(=O)(OP(=O)(OP(=O)([O-])[O-])O)O)OC(C1O)n1cnc2c1ncnc2N
CCOc1ccc(cc1c1nc(=O)c2n([nH]1)c(CCC)nc2C)S(=O)(=O)N1CCN(CC1)CC
OC(=O)CCC(C(=O)O)NC(=O)c1ccc(cc1)NCC1CNc2c(N1C)c(=O)nc([nH]2)N
Oc1ccc(cc1)C(=NNc1ccc(cc1[N+](=O)[O-])[N+](=O)[O-])c1ccc(cc1)O
CC=C(C(=O)OC1C(=CC23C1(O)C(O)C(=CC(C3=O)C1C(CC2C)C1(C)C)CO)C)C
CC#CCn1c(nc2c1c(=O)n(Cc1nc(C)c3c(n1)cccc3)c(=O)n2C)N1CCCC(C1)N
COCCn1nc2c(c1CC)nc([nH]c2=O)c1cc(cnc1OCC)S(=O)(=O)N1CCN(CC1)CC
NC(=O)C(N(S(=O)(=O)c1ccc(cc1)Cl)Cc1ccc(cc1F)c1nocn1)CCC(F)(F)F
CNC(=O)C(N1CCc2c(C1CCc1ccc(cc1)C(F)(F)F)cc(c(c2)OC)OC)c1ccccc1
CC(OC(=O)COc1cccc(c1)CN(S(=O)(=O)c1cccnc1)Cc1ccc(cc1)n1cccn1)C
CCCS(=O)(=O)Nc1ccc(c(c1F)C(=O)c1c[nH]c2c1cc(cn2)c1ccc(cc1)Cl)F
CCCOc1ccc(cc1c1nc(=O)c2c([nH]1)c(CCC)nn2C)S(=O)(=O)NCCC1CCCN1C
CCOC(=O)OCC1OC(Oc2nn(c(c2Cc2ccc(cc2)OC(C)C)C)C(C)C)C(C(C1O)O)O
OC(CNC(Cc1ccc(c(c1)F)Cl)(C)C)COC(c1ccccc1c1ccc(c(c1)C)C(=O)O)C
CNC(=O)c1ccc(cc1F)N1C(=S)N(C(=O)C1(C)C)c1ccc(c(c1)C(F)(F)F)C#N
OC(=O)CC1COc2c1ccc(c2)OCc1cccc(c1)c1c(C)cc(cc1C)OCCCS(=O)(=O)C
Clc1ccc(c(c1)C(=O)c1ccn(=O)cc1)[N-]S(=O)(=O)c1ccc(cc1)C(C)(C)C
CNC(=O)c1ccc(cc1)Nc1ncc(c(n1)NCc1nccnc1N(S(=O)(=O)C)C)C(F)(F)F
Fc1cc(F)c(c(c1)F)CNC(=O)c1cn2CC3OC4CCC(N3C(=O)c2c(c1=O)[O-])C4
CCCC(C(=O)Nc1ncn(c1)C(CNCC(C)(C)C)(C)C)NC1CCc2c(C1)c(F)cc(c2)F
CC(OC(=O)CNc1cccc(n1)CN(S(=O)(=O)c1cccnc1)Cc1ccc(cc1)n1cccn1)C
Clc1ccc(c(c1)Cl)C1(OCC(O1)COc1ccc(cc1)N1CCN(CC1)C(=O)C)Cn1cncc1
[O-]C(=O)CCC(=O)OCC(=O)C1(O)CCC2C1(C)CC(O)C1C2CCC2=CC(=O)CCC12C
OC(=O)CCC(C(=O)O)NC(=O)c1ccc(s1)N(Cc1ccc2c(c1)c(=O)[nH]c(n2)C)C
OCCOc1nc(nc(c1Oc1ccccc1OC)NS(=O)(=O)c1ccc(cc1)C(C)(C)C)c1ncccn1
CCCCC(=O)OC1(C(C)CC2C1(C)CC(O)C1(C2CCC2=CC(=O)C=CC12C)F)C(=O)CO
NCCC(C(=O)NC1CC(N)C(C(C1OC1OC(CO)C(C(C1O)N)O)O)OC1OC(CN)CCC1N)O
CCCCC(=O)OCC(=O)C1C(C)CC2C1(C)CC(O)C1(C2CC(C2=CC(=O)C=CC12C)F)F
[O-]C(=O)CCC(C(=O)O)NC(=O)c1ccc(cc1)N(Cc1cnc2c(n1)c(N)nc(n2)N)C
ClCCN(C(=O)Oc1ccc2c(c1)CCC1C2CCC2(C1CCC2OP(=O)([O-])[O-])C)CCCl
[O-]C(=O)CCC(C(=O)[O-])NC(=O)c1ccc(cc1)NCc1cnc2c(n1)c(N)nc(n2)N
Fc1ccc(cc1)C(c1ccc(cc1)F)CCCN1CCC(CC1)(O)c1ccc(c(c1)C(F)(F)F)Cl
CCCCC(=O)OC1(CCC2C1(C)CC(O)C1C2CCC2=CC(=O)C=CC12C)C(=O)COC(=O)C
OC(=O)C(Nc1ccccc1C(=O)c1ccccc1)Cc1ccc(cc1)OCCc1nc(oc1C)c1ccccc1
N#CC(c1ccccc1)(c1ccccc1)CCN1CCC(CC1)(C(=O)OCCOc1ccccc1)c1ccccc1
CC(=O)OCC(=O)C12N=C(OC1CC1C2(C)CC(O)C2(C1CCC1=CC(=O)C=CC21C)F)C
O=C1c2c(cccc2C(=O)c2c1cn1c2C(C)c2c(C1)cccc2)OP(=O)(OCc1ccccc1)O
COc1ccc(cc1)OC(=O)N(C(c1ccc(cc1)OCCc1nc(oc1C)c1ccccc1)C)CC(=O)O
OC(=O)CCC(C(=O)O)NC(=O)c1cc(c(s1)CCC1CNc2c(C1)c(=O)[nH]c(n2)N)C
[O-]C(=O)CN(CCN(CC(=O)[O-])CC(=O)[O-])CCN(CC(=O)[O-])CC(=O)[O-]
C#CCC(c1ccc(cc1)C(=O)NC(C(=O)O)CCC(=O)O)Cc1cnc2c(n1)c(N)nc(n2)N
CCOC(=O)OC1(CCC2C1(C)CC(O)C1C2CCC2=CC(=O)C=CC12C)C(=O)COC(=O)CC
CCC(=O)N1CCN(CC1)C(=O)OC1N(c2ccc3c(n2)nc(cc3)Cl)C(=O)C2=C1SCCS2
COc1ccc2c(c1)c(CC(=O)OC1OC(=O)c3c1cccc3)c(n2C(=O)c1ccc(cc1)Cl)C
CCN(CCSCC(=O)OC1CC(C)(C=C)C(O)C(C23C(C1(C)C(C)CC2)C(=O)CC3)C)CC
COc1ccc(cc1OC)CCN(CCCC1(c2ccc(c(c2)OC)OC)S(=O)(=O)CCCS1(=O)=O)C
O=C(C(C(=O)Oc1ccc(cc1)C)c1cscc1)NC1C(=O)N2C1SC(C2C(=O)[O-])(C)C
Fc1ccc(c(c1)C)C1CNCCN1C(=O)N(C(c1cc(cc(c1)C(F)(F)F)C(F)(F)F)C)C
O=C(C(c1ccccc1)O)NC1C(=O)N2C1SCC(=C2C(=O)O)CSc1nnnn1CS(=O)(=O)O
CCCCC(=O)OCC(=O)C1(O)C(C)CC2C1(C)CC(O)C1(C2CCC2=CC(=O)C=CC12C)F
CCC(=C(c1ccc(cc1)OP(=O)([O-])[O-])CC)c1ccc(cc1)OP(=O)([O-])[O-]
OCC(c1ccccc1)C(=O)OC1CC2CCC(C1)[N+]2(C)CC(=O)c1ccc(cc1)c1ccccc1
O=C1C=CC2(C(=C1)CCC1C2C(O)CC2(C1CCC2(O)C(=O)COC(=O)C(C)(C)C)C)C
CCOC1OC(=O)CC1NC(=O)C1CCCN2N1C(=O)C(CCC2=O)NC(=O)c1nccc2c1cccc2
CC1=NN(C(=O)C1N=Nc1cccc(c1O)c1cccc(c1)c1[n-]nnn1)c1ccc(c(c1)C)C
CCNCCS(=O)(=O)NCC1(SC(=NN1C(=O)C(C)(C)C)NC(=O)C(C)(C)C)c1ccccc1
O=C(c1ccc(cc1)Cc1c(=O)oc2c(c1[O-])cccc2)OC(C(F)(F)F)(C(F)(F)F)C
COCCOCCOCCOCCOC(=O)OC[n+]1ccc(cc1)NC(=NC#N)NCCCCCCOc1ccc(cc1)Cl
OC(CN(S(=O)(=O)C)C)CN1C2CCC1CC(C2)NC(=O)c1cc2ccccc2n(c1=O)C(C)C
Fc1ccccc1c1nc2c(n1)cn(cc2)Cc1ccc(nn1)c1ccc(cc1C(F)(F)F)C(F)(F)F
O=C(C1(CC1)c1ccc2c(c1)OC(O2)(F)F)Nc1ccc(c(n1)c1cccc(c1)C(=O)O)C
COCCOCCOCCOCCOCCOCCOC1CCC2(C34C1Oc1c4c(CC2N(CC3)CC=C)ccc1O)O
CCc1cc2C(=O)c3c4ccc(cc4[nH]c3C(c2cc1N1CCC(CC1)N1CCOCC1)(C)C)C#N
COc1cc(OC)c(c(c1)OC)C=CS(=O)(=O)Cc1ccc(c(c1)OP(=O)([O-])[O-])OC
COc1cc(ccc1N1CCC(CC1)N1CCN(CC1)C)Nc1nc(NC2CCOCC2)c(nc1C(=O)N)CC
N#Cc1ccc(cc1)c1csc(n1)C(C(c1ccc(cc1F)F)(Cn1cncn1)OCOP(=O)(O)O)C
COC(=O)OCOc1c(=O)ccn2c1C(=O)N1CCOCC1N2C1c2ccccc2SCc2c1ccc(c2F)F
OC(C(NC(=O)c1cccc(c1C)O)CSc1ccccc1)CN1CC2CCCCC2CC1C(=O)NC(C)(C)C
CON=C(c1ccco1)C(=O)NC1C(=O)N2C1SCC(=C2C(=O)OC(OC(=O)C)C)COC(=O)N
O=C1CCC2(C(=C1)CCC1C2C(O)CC2(C1CCC2(O)C(=O)COP(=O)([O-])[O-])C)C
CCCCCCCCCCCCCCCC(=O)OCC(C(c1ccc(cc1)[N+](=O)[O-])O)NC(=O)C(Cl)Cl
O=C(NC1C(=O)N2C1SC(C2C(=O)Oc1cccc(c1)COC(=O)C(C)C)(C)C)Cc1ccccc1
[O-]C(=O)CCC(=O)OCC(=O)C1(O)CCC2C1(C)CC(O)C1C2CCC2=CC(=O)C=CC12C
CCCC(=O)OC1(C(C)CC2C1(C)CC(=O)C1(C2CCC2=CC(=O)C=CC12C)F)C(=O)CCl
CC(COC(=O)OCN1C(=O)CN(CC1=O)CCN1CC(=O)N(C(=O)C1)COC(=O)OCC(C)C)C
Cc1cc2NCCCCCCCCCCNc3cc([n+](CCCCCCCCCC[n+]1c1c2cccc1)c1ccccc31)C
CC(CCCC1(C)CCc2c(O1)c(C)c(c(c2C)OC(=O)c1cccnc1)C)CCCC(CCCC(C)C)C
CON=C(c1csc(n1)N)C(=O)NC1C(=O)N2C1SCC(=C2C(=O)OCOC(=O)C(C)(C)C)C
O=C(NS(=O)(=O)Oc1c(cccc1C(C)C)C(C)C)Cc1c(cc(cc1C(C)C)C(C)C)C(C)C
Brc1ccc(c(c1)C(F)(F)F)NC(=O)c1c(O)c(cc(c1C)[N+](=O)[O-])C(C)(C)C
CCON=C(c1ccc(cc1)Br)C1CCN(CC1)C1(C)CCN(CC1)C(=O)c1c(C)ccn(=O)c1C

Continued on next page

Table S8 – *Continued from previous page*

CC(=O)C1=C(O)C(N)C2C(C1=O)(O)C(=O)c1c(C2)c(C)c2c(c1O)c(O)c(cc2)C
CCCCCCCN(C(=O)Nc1ccc(cc1F)F)CCCCCSc1nc(c([nH]1)c1ccccc1)c1ccccc1
OCC(=O)C12OC(OC1CC1C2(C)CC(Cl)C2(C1CC(C1=CC(=O)C=CC21C)F)Cl)(C)C
C[N+](C1c2ccccc2c2c1cccc2)(CCCCCC[N+](C1c2ccccc2c2c1cccc2)(C)C)C
OC(=O)CCC(=O)OCC(=O)C1(O)CCC2C1(C)CC(O)C1C2CC(C2=CC(=O)C=CC12C)C
OCC(NC1CC(N)C(C(C1OC1OC(CO)C(C(C1O)N)O)O)OC1OC(CN)C(C(C1N)O)O)CO
CCOC(=O)C1=C(C)NC(=C(C1c1ccccc1C=CC(=O)OC(C)(C)C)C(=O)OCC)CN(C)C
CCC(=O)OC1(C(C)CC2C1(C)CC(O)C1(C2CC(C2=CC(=O)C=CC12C)F)F)C(=O)SC
FCC(=O)C12OC(OC1CC1C2(C)CC(Cl)C2(C1CC(C1=CC(=O)C=CC21C)F)Cl)(C)C
COc1cc2c(cc1N1CCC(CC1)N1CCCC1)ncc(c2Nc1ccc(c(c1)Cl)Sc1nccn1C)C#N
OC1COC(CC1O)OC1CC(N)(Cc2c1c(O)c1c(c2O)C(=O)c2c(C1=O)cccc2)C(=O)C
CCC(=O)OC1(CCC2C1(C)CC(O)C1C2CC(C2=CC(=O)C=CC12C)C)C(=O)COC(=O)C
O=C(C1NCC(C1)SC1=C(C(=O)O)N2C(C1C)C(C2=O)C(O)C)Nc1cccc(c1)C(=O)O
N#CC(c1ccc(c(c1)Oc1ccccc1)F)OC(=O)C1C(C1(C)C)C=C(c1ccc(cc1)Cl)Cl
O=CN1C(CNc2ccc(cc2)C(=O)NC(C(=O)O)CCC(=O)O)CNc2c1c(=O)nc([nH]2)N
OCC(C(C(C(NC(=O)Cc1c2cc(OC)ccc2n(c1C)C(=O)c1ccc(cc1)Cl)C=O)O)O)O
FC12C(F)(C(F)(F)C(C(C2(F)F)(F)F)(F)F)C(F)(F)C(C(C1(F)F)(F)F)(F)F
CC(CCCC1(C)CCc2c(O1)c(C)c(c(c2C)OC(=O)CCC(=O)O)C)CCCC(CCCC(C)C)C
CCOc1cc2ncc(c(c2cc1NC(=O)C=CCN(C)C)Nc1ccc(c(c1)Cl)OCc1ccccn1)C#N
O=C1CCC2(N1)CCC(NC2)(COC(c1cc(cc(c1)C(F)(F)F)C(F)(F)F)C)c1ccccc1
CC(C1C(=O)N2C1C(C)C(=C2C(=O)OCOC(=O)C(C)(C)C)SC1CN(C1)C1=NCCS1)O
CC(CN(CC(Cc1[nH]c2c(n1)n(C)c(=O)n(c2=O)C)OC(=O)c1ccccc1)CC(C)C)C
OC1CC(O)C(=C)C(=CC=C2CCCC3(C2CC=C3C(CC=CS(=O)(=O)C(C)(C)C)C)C)C1
COc1cccc(c1C1=NCc2c(c3c1cc(Cl)cc3)nc(nc2)Nc1ccc(c(c1)OC)C(=O)O)F
CS(=O)(=O)N1CCN(CC1)Cc1cc2c(s1)c(nc(n2)c1cccc2c1cn[nH]2)N1CCOCC1
CCOC1OC(=O)CC1NC(=O)C1CCCN1C(=O)C(C(C)(C)C)NC(=O)c1ccc(c(c1)Cl)N
N#CC(c1ccc(cc1)n1c(=O)n(c2c1c1cc(ccc1nc2)c1cnc2c(c1)cccc2)C)(C)C
O=CNC(Cc1c[nH]c2c1cccc2)NC(=O)C(NC(=O)C(N)(C)C)Cc1c[nH]c2c1cccc2
Fc1ccc(c(c1)C(F)(F)F)C(=O)N(C1CCN(CC1)c1nnc(c2c1cccc2)c1ccnn1C)C
CN(C1C(=C(C(=O)N)C(=O)C2(C1CC1C(=C2O)C(=O)c2c(C1(C)O)cccc2O)O)O)C
CN(C1C(=C(C(=O)N)C(=O)C2(C1C(O)C1C(C)c3cccc(c3C(=O)C1=C2O)O)O)O)C
O=C1C=CC2(C(=C1)CCC1C2C(O)CC2(C1CCC2(O)C(=O)COP(=O)([O-])[O-])C)C
Cc1[nH]c2c(n1)CCN(c1c2cccc1)C(=O)c1ccc(cc1)NC(=O)c1ccccc1c1ccccc1
NC(=O)OCC1C(NC(=O)C(=NOCC(=O)[O-])c2csc(n2)N)C(=O)N1S(=O)(=O)[O-]
O=C1C(=C2Nc3c(C2=O)cc(cc3)S(=O)(=O)[O-])Nc2c1cc(cc2)S(=O)(=O)[O-]
COCCNC(=O)CN(CC(=O)[O-])CCN(CC(=O)[O-])CCN(CC(=O)NCCOC)CC(=O)[O-]
CON=C(c1csc(n1)N)C(=O)NC1C(=O)N2C1SCC(=C2C(=O)[O-])CSC(=O)c1ccco1
FCSC(=O)C1(OC(=O)CC)C(C)CC2C1(C)CC(O)C1(C2CC(C2=CC(=O)C=CC12C)F)F
O=C(c1cccnc1)OCC(COC(=O)c1cccnc1)(COC(=O)c1cccnc1)COC(=O)c1cccnc1
OCCn1c(=N)ccn1CC1=C(C(=O)O)N2C(SC1)C(C2=O)NC(=O)C(=NOC)c1csc(n1)N
CON=C(c1nsc(n1)N)C(=O)NC1C(=O)N2C1SCC(=C2C(=O)O)C[n+]1ccn2c1cccn2
CNC1C(OC2C(C1O)OC(C(C2)N)OC1C(N)CC(C(C1O)O)N)OC1OC(CO)C(C(C1O)O)N
CON=C(c1csc(n1)N)C(=O)NC1C(=O)N2C1SCC(=C2C(=O)[O-])C[N+]1(C)CCCC1
O=C1C=CC2(C(=C1)CCC1C2(F)C(O)CC2(C1CC(C2(O)C(=O)COCc1ccccc1)C)C)C
NCCC(CNC1CC(N)C(C(C1OC1OC(CO)C(C(C1O)N)O)O)OC1OC(CN)C(C(C1O)O)O)O
COCCCOc1cc(ccc1OC)CC(C(C)C)CC(C(CC(C(=O)NCC(C(=O)N)(C)C)C(C)C)O)N
O=C(N1CCC2=NN(C(=O)C2(C1)Cc1ccccc1)C)C(NC(=O)C(N)(C)C)COCc1ccccc1
OC1C(COP(=O)(OP(=O)(OCC[N+](C)(C)C)[O-])[O-])OC(C1O)n1ccc(nc1=O)N
CC(CN(S(=O)(=O)c1ccc(cc1)N)CC(C(Cc1ccccc1)NC(=O)OC1COC2C1CCO2)O)C
N#CC(c1ccccc1)(c1ccccc1)CCN1CCC(CC1)(C(=O)ON1C(=O)CCC1=O)c1ccccc1
CN(C(=O)CC(C)(C)C)Cc1cc(ccc1c1ccccc1S(=O)(=O)Nc1noc(c1C)C)c1ncco1
OC(=O)CN(c1c(F)c(Oc2cccc(c2)C2=NCCN2C)nc(c1F)Oc1cc(ccc1O)C(=N)N)C
Clc1ccc(c(c1)Cl)C(Cn1cc[n+](c1)CC(=O)c1ccc(cc1)F)OCc1ccc(cc1Cl)Cl
CC(=O)OCC(=O)C12OC(OC1CC1C2(C)CC(O)C2C1CC(C1=CC(=O)C=CC21C)F)(C)C
CCC(=O)OC1(C(C)CC2C1(C)CC(O)C1(C2CC(C2=CC(=O)C=CC12C)F)F)C(=O)CCl
CCCCCC(C(c1cc(OC(=O)CCCN2CCCCCC2)c2c(c1)OC(C1=C2CCC(C1)C)(C)C)C)C
Nc1cccc(c1)CN1C(Cc2ccccc2)C(O)C(C(N(C1=O)Cc1cccc(c1)N)Cc1ccccc1)O
ClCC(=O)C12OC(OC1CC1C2(C)CC(Cl)C2(C1CC(C1=CC(=O)C=CC21C)F)Cl)(C)C
COCC(N1CCN(CC1C)C1(C)CCN(CC1)C(=O)c1c(C)ncnc1C)c1ccc(cc1)C(F)(F)F
CC=C1NC(=O)C2CSSCCC=CC(OC(=O)C(NC1=O)C(C)C)CC(=O)NC(C(=O)N2)C(C)C
O=c1oc2ccccc2c(c1C1CC(Cc2c1cccc2)c1ccc(cc1)OCc1ccc(cc1)C(F)(F)F)O
CN1CCN(CC1)C=C1N=C2N(C1=O)c1ccc(cc1C(=NC2)c1ccccc1Cl)[N+](=O)[O-]
CNC(=O)CC(C(=O)NC(c1ccc(cc1)O)C(=O)NC1C(=O)N2C1SC(C2C(=O)O)(C)C)N
O=C(C(C(=O)Oc1ccc2c(c1)CCC2)c1ccccc1)NC1C(=O)N2C1SC(C2C(=O)O)(C)C
O=C1C=CC2(C(=C1)CCC1C2(F)C(O)CC2(C1CC(C2(O)C(=O)COP(=O)(O)O)C)C)C
CCC(=O)OC(C(C)C)OP(=O)(CC(=O)N1CC(CC1C(=O)O)C1CCCCC1)CCCCc1ccccc1
Fc1cccc(c1)COc1ccc(cc1Cl)Nc1ncnc2c1cc(cc2)c1ccc(o1)CNCCS(=O)(=O)C
COC1(NC(=O)C(c2ccc(cc2)O)C(=O)O)C(=O)N2C1OCC(=C2C(=O)O)CSc1nnnn1C
CCC(C1C(=O)NC(C(=O)N1C(C(=O)N1CCOCC1)c1coc(n1)C)C1Cc2c(C1)cccc2)C
N#Cc1cccc(c1)C(C(NC(=O)C(Oc1ccc(cn1)C(F)(F)F)(C)C)C)Cc1ccc(cc1)Cl
CC(OC(=O)C(C(C=CC(C1CCC2C1(C)CCCC2=CC=C1CC(O)CC(C1=C)O)C)O)(C)C)C
CN1C(=O)C23CC4C1(CN2CCC3(C)O)CC1(C4(C)C)CNc2c1ccc1c2OC=CC(O1)(C)C
CCc1cc(CCC2(CC(=C(C(=O)O2)Cc2nn3c(n2)nc(cc3C)C)O)C2CCCC2)cc(n1)CC
CCOC(=O)Oc1ccc(cc1OC)C=CC=CC(=O)NCCN1CCC(CC1)OC(c1ccccc1)c1ccccc1
CN1CCN(CC1)Cc1ccc(cc1C(F)(F)F)NC(=O)c1ccc(c(c1)C#Cc1cnc2n1nccc2)C
COCC(C(=O)NC(C(=O)C1(C)OC1)Cc1ccccc1)NC(=O)C(NC(=O)c1cnc(s1)C)COC
COC(=O)N1CCC(CC1)CC#Cc1nc(N)c2c(n1)n(cn2)C1OC(C(C1O)O)C(=O)NC1CC1
CC(Oc1cc(C2CCNCC2)c(cc1Nc1ncc(c(n1)Nc1ccccc1S(=O)(=O)C(C)C)Cl)C)C
O=C(NCC(C(F)(F)F)(F)F)COC(C1=CCC2C1(C)CCCC2=CC=C1CC(O)CC(C1=C)O)C
CCN1CCN(CC1)Cc1ccc(nc1)Nc1ncc(c(n1)c1cc(F)c2c(c1)n(C(C)C)c(n2)C)F
COc1cnc(c2c1c(c[nH]2)C(=O)C(=O)N1CCN(CC1)C(=O)c1ccccc1)n1cnc(n1)C

Continued on next page

CC1C(NC(=O)C(=NOC(C(=O)O)(C)C)c2csc(n2)[NH3+])C(=O)N1S(=O)(=O)[O-]
CN(C1C(=C(C(=O)N)C(=O)C2(C1CC1C(=C2O)C(=O)c2c(C1O)c(Cl)ccc2O)O)O)C
OC(COc1cccc2c1c(=O)cc(o2)C(=O)[O-])COc1cccc2c1c(=O)cc(o2)C(=O)[O-]
CN(C1C(=C(C(=O)N)C(=O)C2(C1C(O)C1C(=C)c3cccc(c3C(=O)C1=C2O)O)O)O)C
CN(C1C(=C(C(=O)N)C(=O)C2(C1CC1Cc3c(C(=O)C1=C2O)c(O)ccc3N(C)C)O)O)C
CC(=O)OC1CC2C(C1(O)C(=O)COC(=O)C)(C)CC(C1(C2CCC2=CC(=O)C=CC12C)F)O
O=S(CCCC(C(F)(F)F)(F)F)CCCCCCCCCC1Cc2cc(O)ccc2C2C1C1CCC(C1(CC2)C)O
OCC1OC(OC2CCC3(C(C2)CCC2C3CCC3(C2(O)CCC3C2=CCOC2=O)C)C)C(C(C1O)O)O
OCC(=O)C12OC(OC1CC1C2(C)CC(O)C2(C1CCC1=CC(=O)C=CC21C)F)(C)c1ccccc1
O=C(C(c1ccccc1)O)NC1C(=O)N2C1SCC(=C2C(=O)O)CSc1nnnn1CS(=O)(=O)[O-]
CON=C(c1csc(n1)N)C(=O)NC1C(=O)N2C1SCC(=C2C(=O)O)C[n+]1cccc2c1CCCC2
ClCSC(=O)C1(OC(=O)CC)C(C)CC2C1(C)CC(O)C1(C2CC(C2=CC(=O)C=CC12C)F)F
CC(=O)OCC(=O)C1(O)C(C)CC2C1(C)CC(O)C1(C2CC(C2=CC(=O)C=CC12C)(F)F)F
[O-][N+](=O)c1cccc(c1)S(=O)(=O)NCCNS(=O)(=O)c1cccc(c1)[N+](=O)[O-]
O=C1NC(=O)C(=C1c1cn(c2c1cccc2)C1CCN(CC1)Cc1ccccn1)c1cn(c2c1cccc2)C
COc1cc(cc(c1OC)OC)C(=O)OCCCN(CCN(CCCOC(=O)c1cc(OC)c(c(c1)OC)OC)C)C
O=C1NC(CC2OC2(C)CCCC(C(C(C(=O)C(C(C1)O)(C)C)C)O)C)C(=Cc1csc(n1)C)C
O=CNc1cn(c(c1)C(=O)Nc1cn(c(c1)C(=O)Nc1cn(c(c1)C(=O)NCCC(=N)N)C)C)C
CNC(C(c1ccccc1)(C)C)C(=O)NC(C(C)(C)C)C(=O)N(C(C(C)C)C=C(C(=O)O)C)C
CCCCCCCCCCCCCCCCCC(=O)OCC(Cn1cnc2c1[nH]c(N)nc2=O)CCOC(=O)C(C(C)C)N
[O-]C(=O)CCC(=O)OC1C2(OC2C2C3(C41OC4CC1C3(C)CCC3=C1COC3=O)O2)C(C)C
Cc1nnc(s1)SCC1=C(C(=O)O)N2C(SC1)C(C2=O)NC(=O)Cn1cc(Cl)c(=O)c(c1)Cl
CCCCCCCCCCCCCCCCCC(=O)OCC(C(c1ccc(cc1)[N+](=O)[O-])O)NC(=O)C(Cl)Cl
O=C1C=CC2(C(=C1)C(F)CC1C2C(O)CC2(C1CC(C2C(=O)COC(=O)C(C)(C)C)C)C)C
CSCC(NC(=O)C(C(=O)OCC(C(c1ccc(cc1)S(=O)(=O)C)O)NC(=O)C(Cl)Cl)N)C=O
COC(=O)CC(C(=O)OC1C(=CC23C1c1cc4OCOc4cc1CCN3CCC2)OC)(CCCC(O)(C)C)O
CCNC(=O)Nc1ncnc2c1ncn2C1OC(C2C1OC(O2)C=Cc1ccccc1)COP(=O)([O-])[O-]
Fc1ccc(cc1)CN1CC(C)N(CC1C)C(=O)c1cc2c(cn(c2cc1Cl)C)C(=O)C(=O)N(C)C
CC(=O)OC1(CCC2C1(C)CC(c1ccc(cc1)N(C)C)C1=C3CCC(=O)C=C3CCC21)C(=O)C
CC1CNC2C(C1)OC1(C2C)CCC2C(=C(C1)C)CC1C2CCC2C1(C)CCC(C2)NS(=O)(=O)C
O=C(C1CC1)Nc1nccc(c1)Oc1cc(F)c(cc1F)NC(=O)C1(CC1)C(=O)Nc1ccc(cc1)F
COc1cc(ccc1Nc1ncc(c(n1)Nc1ccccc1P(=O)(C)C)Cl)N1CCC(CC1)N1CCN(CC1)C
OCC1OC(CC1OP(=O)(OCC1OC(CC1O)n1cnc2c1[nH]c(N)nc2=O)O)n1cnc(nc1=O)N
O=C1C=C2C3CC(C)(CCC3(C)CCC2(C2(C1C1(C)CCC(C(C1CC2)(C)C)O)C)C)C(=O)O
[O-]C(=O)CCC(=O)OCC(=O)C1(O)CCC2C1(C)CC(O)C1C2CC(C2=CC(=O)C=CC12C)C
CC(=O)OCC(=O)C12OC3(OC1CC1C2(C)CC(O)C2(C1CCC1=CC(=O)C=CC21C)F)CCCC3
OC1CC(O)C(=C)C(=CC=C2CCCC3(C2CCC3C(CCCC(C(F)(F)F)(C(F)(F)F)O)C)C)C1
OC1C(N)CC(OC1C)OC1CC(O)(Cc2c1c(O)c1c(c2O)C(=O)c2c(C1=O)cccc2)C(=O)C
CCCCCCCCCCCCCCCC(=O)OC1C(SC)OC(C(C1O)O)C(C(Cl)C)NC(=O)C1CC(CN1C)CCC
OCC(=O)C1(OC(=O)c2ccccc2)C(C)CC2C1(C)CC(O)C1(C2CCC2=CC(=O)C=CC12C)F
CCCCCCC[n+]1c(C)csc1C(=CC=c1scc(n1CCCCCCC)C)C=Cc1scc([n+]1CCCCCCC)C
CNC(=O)C(C(C)(C)C)NC(=O)C(NC(=O)C(CCN1C(=O)N(C(C1=O)(C)C)C)S)CC(C)C
[O-]C(=O)CCC(C(=O)[O-])NC(=O)c1ccc(cc1)CCc1c[nH]c2c1c(=O)[nH]c(n2)N
CNCC(=O)NC(C(C)(C)C)C(=O)NC(C(CN(C(=O)NC(C)(C)C)CCC(C)C)O)Cc1ccccc1
COc1cc2CN(CCc3ccc(cc3)NC(=O)c3cccc4c3[nH]c3c(OC)cccc3c4=O)CCc2cc1OC
CN(Cc1nccn1c1ccc(c(c1)F)NC(=O)c1cc(nn1c1ccc2c(c1)c(N)no2)C(F)(F)F)C
O=C(C1NCC(C1)SC1=C(C(=O)O)N2C(C1C)C(C2=O)C(O)C)Nc1cccc(c1)C(=O)[O-]
COc1cccc2c1C1CN(CC1CO2)CCCCn1c(=O)[nH]c2c(c1=O)sc1c2nc(cn1)c1ccccc1
COC(C(=O)C12OC(OC1CC1C2(C)CC(O)C2(C1CC(C1=CC(=O)C=CC21C)F)F)(C)C)OC
O=C(C(NC(=O)C(N)(C)C)COCc1ccccc1)N1CCC2(CC1)CN(c1c2cccc1)S(=O)(=O)C
[O-]C(=O)CCC(C(=O)[O-])NC(=O)c1ccc(cc1)CCC1CNc2c(C1)c(=O)nc([nH]2)N
Clc1cc(Br)c2c(c1)CCc1c(C2C2CCN(CC2)C(=O)CC2CCN(CC2)C(=O)N)ncc(c1)Br
CCCCCC(C(c1cc(OC(=O)CCCN2CCCCC2)c2c(c1)OC(C1=C2CN(CC#C)CC1)(C)C)C)C
[O-][N+](=O)c1ccc(o1)C=NNC(=O)c1cc(cc(c1O)[N+](=O)[O-])[N+](=O)[O-]
C=CC1(C)CC(OC(=O)CSC2CC3CCC(C2)N3C)C2(C)C(C)CCC3(C(C1O)C)C2C(=O)CC3
OCC1OC(O)C(C(C1O)OC(C(=O)NC(C(=O)NC(C(=O)N)CCC(=O)O)C(O)C)C)NC(=O)C
Clc1ccc(cc1)C1(O)CC[N+](CC1)([O-])CCC(C(=O)N(C)C)(c1ccccc1)c1ccccc1
OCC1OC(Oc2cc3CCC(c4c(c3c(c2OC)OC)ccc(c(=O)c4)SC)NC(=O)C)C(C(C1O)O)O
COc1cc(cc(c1OC)OC)C(=O)OCCCN1CCCN(CC1)CCCOC(=O)c1cc(OC)c(c(c1)OC)OC
[O-]C(=O)CCC(=O)OC1CC2C(C1OC(=O)CCC(=O)[O-])(C)CCC1C2CCc2c1ccc(c2)O
[O-]C(=O)CCC(C(=O)[O-])NC(=O)c1ccc(cc1)NCc1cnc2c(n1)c(=O)[nH]c(n2)N
ON=C(c1nsc(n1)N)C(=O)NC1C(=O)N2C1SCC(=C2C(=O)O)C=C1CCN(C1=O)C1CNCC1
CNc1cc2[nH]c(=O)n(c(=O)c2cc1F)c1ccc(cc1)NC(=O)NS(=O)(=O)c1ccc(s1)Cl
O=C(Nc1noc(c1)C(C)(C)C)Nc1ccc(cc1)c1cn2c(n1)sc1c2ccc(c1)OCCN1CCOCC1
COc1cccc(c1C1=NCc2c(c3c1cc(Cl)cc3)nc(nc2)Nc1ccc(c(c1)OC)C(=O)[O-])F
CCC(C1C(=O)NC(C(=O)N1C(c1ccc(nc1C)C)C(=O)N1CCOCC1)C1Cc2c(C1)cccc2)C
NCCC(C(=O)NC1CC(N)C(C(C1OC1OCC(C(C1O)NC)(C)O)O)OC1OC(=CCC1N)CNCCO)O
Fc1cc(cnc1N1CCN(CC1)C(=O)c1cc(ccc1OC(C(F)(F)F)C)S(=O)(=O)C)C(F)(F)F
CCCCCCCCCCCCCC(=O)OCN1C(=O)CCc2c1cc(OCCCCN1CCN(CC1)c1cccc(c1Cl)Cl)cc2
CC(OC(=O)C(NP(=O)(Oc1ccccc1)OCC1OC(C(C1O)(C)F)n1ccc(=O)[nH]c1=O)C)C
C=CC(=O)Nc1cccc(c1)Nc1nc(ncc1C(F)(F)F)Nc1ccc(cc1OC)N1CCN(CC1)C(=O)C
CC(=O)OCC(=O)C12OC(OC1CC1C2(C)CC(O)C2(C1CC(C1=CC(=O)C=CC21C)F)F)(C)C
COC(=O)C1=C(C)NC(=C(C1c1cccc(c1)[N+](=O)[O-])C(=O)OCCN(Cc1ccccc1)C)C
CCC(=O)OC(C(C)C)OP(=O)(CC(=O)N1CC(CC1C(=O)[O-])C1CCCCC1)CCCCc1ccccc1
ClCC(=O)C1(OC(=O)c2ccco2)C(C)CC2C1(C)CC(O)C1(C2CCC2=CC(=O)C=CC12C)Cl
CON=C(c1nsc(n1)N)C(=O)NC1C(=O)N2C1SCC(=C2C(=O)[O-])C[n+]1ccn2c1cccn2
COCCOC(=O)C1=C(C)NC(=C(C1c1cccc(c1)[N+](=O)[O-])C(=O)OCC=Cc1ccccc1)C
CCCC(=O)OC1(CCC2C1(C)CC(O)C1(C2CC(C2=CC(=O)C=CC12C)F)F)C(=O)COC(=O)C
O=C(C(C(=O)Oc1ccc2c(c1)CCC2)c1ccccc1)NC1C(=O)N2C1SC(C2C(=O)[O-])(C)C
OC1C(OC2CCC3(C(=C2)CCC2C3CCC3(C2(O)CCC3c2ccc(=O)oc2)C)C)OC(C(C1O)O)C
CON=C(C(=O)NC1C(=O)N2C1SCC(=C2C(=O)[O-])C[n+]1cccc2c1CCC2)c1csc(n1)N

Continued on next page

CN(C1C(=C(C(=O)N)C(=O)C2(C1C(O)C1C(=C2O)C(=O)c2c(C1(C)O)cccc2O)O)O)C
O=c1[nH]c(=O)c2c(n1)n(CC(C(C(COP(=O)(O)[O-])O)O)O)c1c(n2)cc(c(c1)C)C
CCC(=O)OC1(C(C)CC2C1(C)CC(O)C1(C2CCC2=CC(=O)C=CC12C)F)C(=O)COC(=O)CC
CCN1CCN(C(=O)C1=O)C(=O)NC(C(=O)NC1C(=O)N2C1SC(C2C(=O)O)(C)C)c1ccccc1
COc1ccc2c(c1)C(=O)N(C(=O)C2(C)C)CCc1ccc(cc1)S(=O)(=O)NC(=O)NC1CCCCC1
NCC(C(=O)NC1CC(N)C(C(C1OC1OCC(C(C1O)NC)(C)O)O)OC1OC(CN)C(C(C1O)O)O)O
O=C1C=CC2(C(N1)CCC1C2CCC2(C1CCC2C(=O)Nc1cc(ccc1C(F)(F)F)C(F)(F)F)C)C
O=C1C=CC2(C(=C1)C(C)CC1C2C(O)CC2(C1CCC2(O)C(=O)COP(=O)([O-])[O-])C)C
O=C(NC(C(=O)NC1C(=O)N2C1SC(C2C(=O)[O-])(C)C)c1ccccc1)CNC(=N)c1ccncc1
Cc1ncn(c1)c1cc(NC(=O)c2ccc(c(c2)Nc2nccc(n2)c2cccnc2)C)cc(c1)C(F)(F)F
O=C(CC(C)(C)C)OCC(=O)C1(O)C(C)CC2C1(C)CC(O)C1(C2CCC2=CC(=O)C=CC12C)F
[O-]C(=O)CCC(C(=O)[O-])NC(=O)c1ccc(cc1)NCC1CNc2c(N1C)c(=O)nc([nH]2)N
CN1CCc2c(C1)sc(n2)C(=O)NC1CC(CCC1NC(=O)C(=O)Nc1ccc(cn1)Cl)C(=O)N(C)C
CCOc1c(OCC)cc2c(c1F)C(=N)N(C2)CC(=O)c1cc(N2CCOCC2)c(c(c1)C(C)(C)C)OC
COCC(=O)C1(CCC2C1(C)CC(c1ccc(cc1)N(C)C)C1=C3CCC(=O)C=C3CCC21)OC(=O)C
Nc1nc(cc(n1)c1ccc(cc1)CC(C(=O)O)N)OC(C(F)(F)F)c1ccc(cc1n1ccc(n1)C)Cl
CC1CNC(C2CC2)C(=O)N(C)C(C)C(=O)NC(C(=O)NCCCc2c(O1)cccc2)Cc1ccc(cc1)F
CCN(CCOC(=O)C(NC(=O)c1c(C)cccc1Cl)Cc1ccc(cc1)NC(=O)c1c(Cl)cccc1Cl)CC
O=C1NC(=O)C2(N1)CCC(NC2)(COC(c1cc(cc(c1)C(F)(F)F)C(F)(F)F)C)c1ccccc1
CCCCCCOC(c1cccc(c1OC)c1csc(n1)NC(=O)c1cc(Cl)c(c(c1)Cl)C=C(C(=O)O)C)C
COc1c(cc(cc1C(C)(C)C)n1ccc(=O)[nH]c1=O)c1ccc2c(c1)ccc(c2)NS(=O)(=O)C
O=C(N1CCC(CC1)n1c(=O)[nH]c2c1cccn2)OC1CCC(C(c2c1nccc2)N)c1cccc(c1F)F
CON(Cc1ccc(c2c1CC1CC3C(N(C)C)C(=C(C(=O)C3(C(=C1C2=O)O)O)C(=O)N)O)O)C
CC(OC(=O)C(NP(=O)(Oc1ccccc1)OCC1OC(C(C1O)(C)Cl)n1ccc(=O)[nH]c1=O)C)C
COc1cc(cc(c1O)OC)C1C2C(=O)OCC2C(c2c1cc1OCOc1c2)OC1OC2COC(OC2C(C1O)O)C
COC1(NC(=O)C2SC(=C(C(=O)O)C(=O)N)S2)C(=O)N2C1SCC(=C2C(=O)O)CSc1nnnn1C
COc1cc(ccc1Cc1cn(c2c1cc(cc2)NC(=O)OC1CCCC1)C)C(=O)NS(=O)(=O)c1ccccc1C
CCC(=O)OC1(C(C)CC2C1(C)CC(O)C1(C2CCC2=CC(=O)C=CC12C)Cl)C(=O)COC(=O)CC
C=CC[N+]1(CCCC1)C1CC2C(C1OC(=O)C)(C)CCC1C2CCC2C1(C)CC(C(C2)O)N1CCOCC1
O=C(C(c1ccccc1)O)NC1C(=O)N2C1SCC(=C2C(=O)[O-])CSc1nnnn1CS(=O)(=O)[O-]
COCC1=C(C(=O)OC(OC(=O)OC(C)C)C)N2C(SC1)C(C2=O)NC(=O)C(=NOC)c1csc(n1)N
CC(=O)OC1(C(C)CC2C1(C)CC(O)C1(C2CC(C2=CC(=O)C=CC12C)F)F)C(=O)COC(=O)C
CCC(=O)OC1(C(C)CC2C1(C)CC(O)C1C2C(Cl)CC2=CC(=O)C=CC12C)C(=O)COC(=O)CC
CCCC(=O)OC1(C(C)CC2C1(C)CC(O)C1(C2CCC2=CC(=O)C=CC12C)F)C(=O)COC(=O)CC
CN(C1C(=C(C(=O)N)C(=O)C2(C1CC1C(=C2O)C(=O)c2c(C1(C)O)c(Cl)ccc2O)O)O)C
CC(CN(S(=O)(=O)c1ccc(cc1)N)CC(C(Cc1ccccc1)NC(=O)OC1CCOC1)OP(=O)(O)O)C
NCCC(C(=O)NC1CC(N)C(C(C1OC1OC(CO)C(C(C1O)N)O)O)OC1OC(CN)C(C(C1O)O)O)O
O=C1C=CC2(C(=C1)C(F)CC1C2(F)C(O)CC2(C1CC(C2C(=O)COC(=O)C(C)(C)C)C)C)C
O=C(Nc1c(Cl)cc(cc1Cl)N)CN1CCN(CC1C(=O)N)CCCCC(c1ccc(cc1)F)c1ccc(cc1)F
CCc1cc(cc2c1OCO2)C1=C(C(=O)[O-])N(S(=O)(=O)c2c1cccc2)c1ccccc1C(F)(F)F
CC(=O)OC1CC2(C(C1=C(C(=O)O)CCC=C(C)C)CC(C1C2(C)CCC2C1(C)CCC(C2C)O)O)C
CC(=O)OC1CCC2C1(C)CCC1C2CCC2C1(C)CC(Cl)C(=NOc1ccc(cc1)[N+](=O)[O-])C2
CCC(=O)OCC(=O)C12OC(OC1CC1C2(C)CC(O)C2(C1CC(C1=CC(=O)C=CC21C)F)F)(C)C
COC1=C(N)C(=O)c2c(C1=O)ccc(n2)c1nc(C(=O)O)c(c(c1N)c1ccc(c(c1O)OC)OC)C
COC(=O)N(C(=O)N1COC2(C(=N1)c1ccc(cc1C2)Cl)C(=O)OC)c1ccc(cc1)OC(F)(F)F
CC(CC(C(=O)NC1CCC(N(CC1=O)S(=O)(=O)c1ccccn1)C)NC(=O)c1cc2c(o1)cccc2)C
OC(CC(CC(=O)O)O)CCn1c(C(C)C)c(c(c1c1ccc(cc1)F)c1ccccc1)C(=O)Nc1ccccc1
CON=C(c1csc(n1)N)C(=O)NC1C(=O)N2C1SCC(=C2C(=O)O)CSc1nc(c(s1)CC(=O)O)C
CON=C(c1csc(n1)N)C(=O)NC1C(=O)N2C1SCC(=C2C(=O)[O-])C[n+]1cccc2c1CCCC2
O=C1C=CC2(C(=C1)CCC1C2(F)C(O)CC2(C1CC(C2(O)C(=O)COC(=O)c1ccncc1)C)C)C
O=C1C=CC2(C(=C1)CCC1C2(F)C(O)CC2(C1CC(C2(O)C(=O)COS(=O)(=O)[O-])C)C)C
O=C(CCc1ccccc1)OCC(=O)C1(O)C(C)CC2C1(C)CC(O)C1(C2CCC2=CC(=O)C=CC12C)F
C=CCC1C(O)C(C)CCCC2(C)OC2CC(OC(=O)CC(C(C1=O)(C)C)O)c1ccc2c(c1)nc(s2)C
O=C(NCC(F)(F)F)CNC(=O)c1ccc(cc1C)C1=NOC(C1)(c1cc(Cl)cc(c1)Cl)C(F)(F)F
COc1cccc(c1)N1CCN(CC1)C1=Nc2c(F)cccc2C(N1c1cc(ccc1OC)C(F)(F)F)CC(=O)O
OCC1OC(CC1OP(=O)(OCC1OC(CC1O)n1cnc2c1[nH]c(N)nc2=O)[O-])n1cnc(nc1=O)N
CCN(CCNC(=O)c1c(C)[nH]c(c1C)C=C1C(=O)Nc2c1cc(cc2)c1csc(n1)c1ccccc1)CC
CCCc1nc(c(n1Cc1ccc(cc1)c1ccccc1c1nnn[nH]1)C(=O)OCc1oc(=O)oc1C)C(O)(C)C
O=CN1C(CNc2ccc(cc2)C(=O)NC(C(=O)[O-])CCC(=O)[O-])CNc2c1c(=O)nc([nH]2)N
COc1ccc2c(c1)c(CC(=O)OCC=C(CCC=C(CCC=C(C)C)C)C)c(n2C(=O)c1ccc(cc1)Cl)C
O=C(C(C)C)OCC(=O)C12OC(OC1CC1C2(C)CC(O)C2C1CCC1=CC(=O)C=CC21C)C1CCCCC1
CC(=O)OC1(CCC2C1(C)CC(O)C1(C2CC(C2=CC(=O)C(=CC12C)Br)F)F)C(=O)COC(=O)C
O=C1C=CC2(C(=C1)C(F)CC1C2(Cl)C(O)CC2(C1CC(C2C(=O)COC(=O)C(C)(C)C)C)C)C
O=C(C(c1ccccc1)NC(=O)c1cnc2c(c1O)nccc2)NC1C(=O)N2C1SC(C2C(=O)[O-])(C)C
OC1C2C(=C(C3(C1C(N(C)C)C(=C(C3=O)C(=O)N)O)O)O)C(=O)c1c(C2=C)c(Cl)ccc1O
N#[N+]C=C(CCC(C(=O)NC(C(=O)O)CCC(=C[N+]#N)[O-])NC(=O)CCC(C(=O)O)N)[O-]
CCCCC(COc1ccc(c(c1)O)c1nc(nc(n1)c1ccc(cc1)OC)c1ccc(cc1O)OCC(CCCC)CC)CC
NC1CC(OC(C1O)C)OC1CC(O)(Cc2c1c(O)c1c(c2O)C(=O)c2c(C1=O)c(O)ccc2)C(=O)C
Fc1ccc(cc1)S(=O)(=O)N1CCN(CC1)C(=O)c1ccc(cc1)Nc1ccnc2c1ccc(c2)C(F)(F)F
CCCCCCCCCCCCCCCC(=O)OC1CCCn2c1nc(C)c(c2=O)CCN1CCC(CC1)c1noc2c1ccc(c2)F
CCC(C(C)C)CCC(C1CCC2C1(C)CCC1C2CC=C2C1(C)CCC(C2)OC1OC(CO)C(C(C1O)O)O)C
FCSC(=O)C1(OC(=O)c2ccco2)C(C)CC2C1(C)CC(O)C1(C2CC(C2=CC(=O)C=CC12C)F)F
[O-]S(=O)(=O)C1=CC2=NC3=CC4C(=Nc5c(N4)ccc(c5)S(=O)(=O)[O-])C=C3NC2C=C1
CON=C(c1csc(n1)N)C(=O)NC1C(=O)N2C1SCC(=C2C(=O)O)CSc1nc(=O)c(=O)[nH]n1C
OC(=O)CC1(CSC(c2cccc(c2)C=Cc2ccc3c(n2)cc(cc3)Cl)CCc2ccccc2C(O)(C)C)CC1
[O-]C(=O)CCC(C(=O)[O-])NC(=O)c1ccc(cc1)N1CC2N(C1)c1c(NC2)[nH]c(nc1=O)N
N#CC1(CC1)NC(=O)C(CC(F)(C)C)NC(C(F)(F)F)c1ccc(cc1)c1ccc(cc1)S(=O)(=O)C
CNc1cc2[nH]c(=O)n(c(=O)c2cc1F)c1ccc(cc1)NC(=O)[N-]S(=O)(=O)c1ccc(s1)Cl
CCOC(=O)C(Cc1ccc(cc1)c1cc(nc(n1)N)OC(C(F)(F)F)c1ccc(cc1n1ccc(n1)C)Cl)N
COC(=O)C(c1ccccc1)N1CCC(CC1)c1ccc(cc1)NC(=O)c1ccccc1c1ccc(cc1)C(F)(F)F
O=C1CCC2=C3C(CC4(C(C3CCC2=C1)CCC4(O)C(C(F)(F)F)(F)F)C)c1ccc(cc1)C(=O)C

Continued on next page

COC(=O)c1ccc2c(c1)NC(=O)C2=C(c1ccccc1)Nc1cccc(c1)N(C(=O)CN1CCN(CC1)C)C
COC(=O)c1ccc2c(c1)NC(=O)C2=C(c1ccccc1)Nc1ccc(cc1)N(C(=O)CN1CCN(CC1)C)C
O=C(c1ccc2c(c1)CCC1C2(CCC(C1)(OP(=O)(O)O)C(F)(F)F)Cc1ccccc1)Nc1cccnc1C
C=CC(=O)N1CCC(C1)Oc1nc(Nc2ccc(cc2)N2CCC(CC2)N2CCN(CC2)C)c(nc1CC)C(=O)N
CCN1CCN(C(=O)C1=O)C(=O)NC(C(=O)NC1C(=O)N2C1SC(C2C(=O)[O-])(C)C)c1ccccc1
CC(Sc1cc(c(c(c1)C(C)(C)C)O)C(C)(C)C)(Sc1cc(c(c(c1)C(C)(C)C)O)C(C)(C)C)C
CC(=O)OC1CC2CCC3C(C2(CC1N1CCCCC1)C)CCC1(C3CC(C1OC(=O)C)[N+]1(C)CCCCC1)C
O=C1C=CC2(C(=C1)CCC1C2(F)C(O)CC2(C1CC(C2(O)C(=O)COP(=O)([O-])[O-])C)C)C
COc1cc2CCN(C3c2cc1Oc1c2c(CCN(C2Cc2ccc(Oc4cc(C3)ccc4OC)cc2)C)cc2c1OCO2)C
COC(=O)C1=C(C)NC(=C(C1c1cccc(c1)[N+](=O)[O-])C(=O)OC1CCN(C1)Cc1ccccc1)C
CCC=C(c1csc(n1)N)C(=O)NC1C(=O)N2C1SCC(=C2C(=O)OCOC(=O)C(C)(C)C)COC(=O)N
COC1(NC(=O)C(c2ccc(cc2)O)C(=O)[O-])C(=O)N2C1OCC(=C2C(=O)[O-])CSc1nnnn1C
OCC(C(C(C(C(C1OC(C(C(C1N)O)O)n1ccc(nc1=O)N)OC1OC(CO)C(C(C1O)N)O)O)O)O)O
CC(=CC=CC=C(C=CC=C(C=CC1=C(C)CCCC1(C)C)C)C)C=CC=C(C=CC1=C(C)CCCC1(C)C)C
O=C(C(c1cc(cc(c1)C(F)(F)F)C(F)(F)F)(C)C)N(c1cnc(cc1c1ccccc1C)N1CCOCC1)C
CC(=O)OCC(=O)C1(O)C(C)CC2C1(C)CC(O)C1C2C=C(C2=Cc3c(CC12C)cnn3c1ccccc1)C
CSCC(C(=O)NC(C(C(=O)OC(C)C)O)CC1CCCCC1)NC(=O)C(NC(=O)N1CCOCC1)Cc1ccccc1
CCOCn1cc(F)c(=O)n(c1=O)C(=O)c1cccc(c1)C(=O)Oc1nc(ccc1C#N)OC(=O)c1ccccc1
OCC(=O)C1(O)CC(OC2CC(N)CC(O2)C)c2c(C1)c(O)c1c(c2O)C(=O)c2c(C1=O)cccc2OC
O=C(C(c1ccccc1)NC(=O)N1CCN(C1=O)S(=O)(=O)C)NC1C(=O)N2C1SC(C2C(=O)O)(C)C
COC1C(CC2OC1(C)n1c3ccccc3c3c1c1n2c2ccccc2c1c1c3CNC1=O)N(C(=O)c1ccccc1)C
O=CCC1CC(C)C(=O)C=CC(=CC(C(OC(=O)CC(C(C1OC1OC(C)CC(C1O)N(C)C)C)O)CC)C)C
OC1C=CC(=CC(NC(=O)C(=O)C)C2(C)C(=O)OC(CC(C=CC(=CC1)C)OC(=O)C)C(C2=O)C)C
CCCCN1C(=O)C(NC(=O)C21CCN(CC2)Cc1ccc(cc1)Oc1ccc(cc1)C(=O)O)C(C1CCCCC1)O
COc1ccc(nn1)NS(=O)(=O)c1ccc(cc1)NC(S(=O)(=O)O)c1c(C)n(n(c1=O)c1ccccc1)C
CN1CCCC1CCNC(=O)c1cn2c3cc4ccccc4cc3oc3c2c(c1=O)cc(c3N1CCC(C1)c1cnccn1)F
NC(=N)NCC(=O)NC1CCN(C1)C(=O)C1CC(CN1C)SC1=C(C(=O)O)N2C(C1C)C(C2=O)C(O)C
[N-]=[N+]=NC1(COC(=O)C(C)C)OC(C(C1OC(=O)C(C)C)OC(=O)C(C)C)n1ccc(nc1=O)N
CON=C(C(=O)NC1C(=O)N2C1SCC=C2C(=O)OCOC(=O)C(C)(C)C)c1csc(n1)NC(=O)C(N)C
COC(=O)C(NC(=O)c1c(Cl)cccc1Cl)Cc1ccc(cc1)n1c(=O)c2cc(ccc2n(c1=O)C)N(C)C
CN(C1CCN(C1)Cc1ccc(cc1C(F)(F)F)C(=O)Nc1ccc(c(c1)Nc1nccc(n1)c1cncnc1)C)C
CNC(=O)c1nccc(c1)Oc1ccc(c(c1)F)NC(=O)Nc1cc(nn1c1ccc2c(c1)cccn2)C(C)(C)C
COc1c(cc(cc1C(C)(C)C)n1ccc(=O)[nH]c1=O)c1ccc2c(c1)ccc(c2)[N-]S(=O)(=O)C
CN1CCN(CC1)c1ccc(c(c1)NC1CCOCC1)C(=O)Nc1n[nH]c2c1cc(cc2)Cc1cc(F)cc(c1)F
O=C(Nc1ccccc1c1ccccc1)OC1CCN(CC1)CCN(C(=O)c1ccc(cc1)CN1CCC(CC1)C(=O)N)C
O=C(NC(C(=O)NCC(=O)O)CSSCC(C(=O)NCC(=O)O)NC(=O)CCC(C(=O)O)N)CCC(C(=O)O)N
CC(=O)OC1CC2(C(C1=C(C(=O)[O-])CCC=C(C)C)CC(C1C2(C)CCC2C1(C)CCC(C2C)O)O)C
O=C(N(CCCCCCCCCCN(C(=O)Oc1cccc(c1)[N+](C)(C)C)C)C)Oc1cccc(c1)[N+](C)(C)C
O=C1C=CC2(C(=C1)C(F)CC1C2(F)C(O)CC2(C1CC(C2(O)C(=O)COC(=O)C(C)(C)C)C)C)C
C=CC1(C)CC(=O)C2(C(O1)(C)C(OC(=O)C)C(C1C2(C)C(O)CCC1(C)C)OC(=O)CCN(C)C)O
COC(=O)C1=C(C)NC(=C(C1c1cccc(c1)[N+](=O)[O-])C(=O)OC1CCCN(C1)Cc1ccccc1)C
CC(=O)OCC(=O)C1(OC(=O)c2ccco2)C(C)CC2C1(C)CC(O)C1(C2CCC2=CC(=O)C=CC12C)F
CCCCCCCCCCCCCCCC(=O)OCCC1CCN(CC1)CCCN1c2ccccc2Sc2c1cc(cc2)S(=O)(=O)N(C)C
Fc1ccc(cc1)C1C(OCCN1Cc1n[nH]c(=O)[nH]1)OC(c1cc(cc(c1)C(F)(F)F)C(F)(F)F)C
CON=C(C(=O)NC1C(=O)N2C1SCC(=C2C(=O)OC(OC(=O)C(OC)(C)C)C)COC(=O)N)c1ccco1
CCCC(=O)OC1(C(C)CC2C1(C)CC(Cl)C1(C2CCC2=CC(=O)C=CC12C)Cl)C(=O)COC(=O)CCC
OCC1OC(C(C1OC1OC(CN)C(C(C1N)O)O)O)OC1C(O)C(N)CC(C1OC1OC(CN)C(C(C1N)O)O)N
[O-]P(=O)(CN(CP(=O)([O-])[O-])CCN(CP(=O)([O-])[O-])CP(=O)([O-])[O-])[O-]
OC(=O)C(NC(=O)c1ccc(cc1)NCc1cnc2c(n1)c(N)nc(n2)N)CCCNC(=O)c1ccccc1C(=O)O
O=C(c1cccnc1)OC1C(COC(=O)c2cccnc2)OC(C1OC(=O)c1cccnc1)(O)COC(=O)c1cccnc1
OCC1OC(C(C1OC1OC(CN)C(C(C1N)O)O)O)OC1C(O)C(N)CC(C1OC1OC(CO)C(C(C1N)O)O)N
OCC1OC(OC2C(OC3C(O)C(O)C(C(C3NC(=N)N)O)NC(=N)N)OC(C2(O)CO)C)C(C(C1O)O)NC
CCOc1nc2c(n1Cc1ccc(cc1)c1ccccc1c1noc(=O)[nH]1)c(ccc2)C(=O)OCc1oc(=O)oc1C
CCC1(O)C(=O)OCc2c1cc1c3nc4ccc(cc4c(c3Cn1c2=O)CC)OC(=O)N1CCC(CC1)N1CCCCC1
CCOc1nc2c(n1Cc1ccc(cc1)c1ccccc1c1noc(=O)[n-]1)c(ccc2)C(=O)OCc1oc(=O)oc1C
COc1cc(Nc2ncc(c(n2)Nc2ccc3c(n2)N(COP(=O)(O)O)C(=O)C(O3)(C)C)F)cc(c1OC)OC
O=C(N1CCC(CC1)n1c(=O)[nH]c2c1cccn2)NC1CCC(CN(C1=O)CC(F)(F)F)c1cccc(c1F)F
O=C(N1CCC(CC1)n1c(=O)[n-]c2c1cccn2)NC1CCC(CN(C1=O)CC(F)(F)F)c1cccc(c1F)F
COc1cc2c(ccnc2cc1OCCCN1CCOCC1)Oc1ccc(cc1F)NC(=O)C1(CC1)C(=O)Nc1ccc(cc1)F
OC(=O)CCNC(=O)c1ccc(cc1)C(Oc1cc(C)c(c(c1)C)c1ccc(cc1)C(C)(C)C)CCC(F)(F)F
[O-]C(=O)CC1(CSC(c2cccc(c2)C=Cc2ccc3c(n2)cc(cc3)Cl)CCc2ccccc2C(O)(C)C)CC1
OC(CN1CCN(CC1C(=O)NC(C)(C)C)Cc1cccnc1)CC(C(=O)NC1C(O)Cc2c1cccc2)Cc1ccccc1
O=C(CC(C)(C)C)OCC(=O)C12OC(OC1CC1C2(C)CC(O)C2(C1CCC1=CC(=O)C=CC21C)F)(C)C
COc1cccc2c1C(=O)c1c(C2=O)c(O)c2c(c1O)C(OC1CC(N)C(C(O1)C)O)CC(C2)(O)C(=O)C
O=C(NC(C(CC(Cc1ccccc1)NC(=O)C(N1CCCNC1=O)C(C)C)O)Cc1ccccc1)COc1c(C)cccc1C
CN(C1C(=C(C(=O)NCN2CCCC2)C(=O)C2(C1CC1C(=C2O)C(=O)c2c(C1(C)O)cccc2O)O)O)C
Oc1ccc(cc1)C(C(=O)NC1C(=O)N2C1SCC(=C2C(=O)O)CSc1nnnn1C)NC(=O)c1cnc(cc1O)C
CCCCCCCCCCCCCCCC(=O)OCC(OC(=O)CCCCCCCCCCCCCCC)COP(=O)(OCC[N+](C)(C)C)[O-]
O=C(CC(C(=O)N(C1CC1)CC(=O)O)NS(=O)(=O)c1ccc2c(c1)cccc2)NCC1CCCN(C1)C(=N)N
CSCCC(N(C(=O)C(Cc1ccccc1)NC(=O)CNC(=O)C(NC(=O)C(Cc1ccc(cc1)O)N)C)C)C(=O)N
CN(C1C(=C(C(=O)N)C(=O)C2(C1CC1Cc3c(C(=O)C1=C2O)c(O)ccc3[N+](=O)[O-])O)O)C
O=CCC1CC(C)C(=O)C=CC2(C)OC2C(C(OC(=O)CC(C(C1OC1OC(C)CC(C1O)N(C)C)C)O)CC)C
OCNC(=O)C1=C(O)C(N(C)C)C2C(C1=O)(O)C(=C1C(C2)C(C)(O)c2c(C1=O)c(O)ccc2Cl)O
COc1cccc2c1C(=O)c1c(C2=O)c(O)c2c(c1O)C(OC1CC(N)C(C(O1)C)O)CC(C2)(O)C(=O)C
O=C1C=CC2(C(=C1)CCC1C2C(O)CC2(C1CCC2(O)C(=O)COC(=O)C1CC=CCC1C(=O)[O-])C)C
OCC1OC(OC2C(OC3C(O)C(O)C(C(C3NC(=N)N)O)NC(=N)N)OC(C2(O)C=O)C)C(C(C1O)O)NC
FC(C(C(C(C(C(C(C(C(Br)(F)F)(F)F)(F)F)(F)F)(F)F)(F)F)(F)F)(F)F)(C(F)(F)F)F
FC(Oc1ccc(cc1)OC1CCN(CC1)c1ccc(cc1)OCC1(C)Oc2n(C1)cc(n2)[N+](=O)[O-])(F)F
OC(=O)CC(C(=O)COc1c(F)c(F)cc(c1F)F)NC(=O)C(NC(=O)C(=O)Nc1ccccc1C(C)(C)C)C
O=C(c1cnc2c(c1)CC1(C2)C(=O)Nc2c1cccn2)NC1CC(c2ccccc2)C(N(C1=O)CC(F)(F)F)C
COc1cnc(c2c1c(cn2COP(=O)(O)O)C(=O)C(=O)N1CCN(CC1)C(=O)c1ccccc1)n1cnc(n1)C

Continued on next page

C#CCN(c1ccc(c(c1)F)C(=O)NC(C(=O)O)CCc1nnn[nH]1)Cc1cc2c(=O)nc([nH]c2cc1C)C
N#CCC1(CN(C1)C1CCN(CC1)C(=O)c1ccnc(c1F)C(F)(F)F)n1ncc(c1)c1ncnc2c1cc[nH]2
CCOc1nc2c(n1Cc1ccc(cc1)c1ccccc1c1nnn[nH]1)c(ccc2)C(=O)OC(OC(=O)OC1CCCCC1)C
COc1ccc(cc1O)C1CC(=O)c2c(O1)cc(cc2O)OC1OC(COC2OC(C)C(C(C2O)O)O)C(C(C1O)O)O
FCON=C(c1nsc(n1)N)C(=O)NC1C(=O)N2C1SCC(=C2C(=O)[O-])C=CC[N+](CC(=O)N)(CC)C
OCC(=O)C1(O)CC(OC2CC(N)C(C(O2)C)O)c2c(C1)c(O)c1c(c2O)C(=O)c2c(C1=O)cccc2OC
CSCCC(C(=O)NC(C(=O)NC(C(=O)N)Cc1ccccc1)CC(=O)O)NC(=O)C(Cc1c[nH]c2c1cccc2)N
CCCCCCCCCCCCCCCCCC(=O)OCC(=O)C1(O)C(C)CC2C1(C)CC(O)C1(C2CCC2=CC(=O)C=CC12C)F
CON=C(c1csc(n1)N)C(=O)NC1C(=O)N2C1SCC(=C2C(=O)OCOC(=O)C(C)(C)C)C=Cc1scnc1C
CON=C(c1csc(n1)N)C(=O)NC1C(=O)N2C1SCC(=C2C(=O)OCOC(=O)C(C)(C)C)Cn1nnc(n1)C
O=C(C(c1ccccc1)NC(=O)N1CCN(C1=O)S(=O)(=O)C)NC1C(=O)N2C1SC(C2C(=O)[O-])(C)C
OC(=O)CN(Cc1c(cnc(c1O)C)COP(=O)(O)O)CCN(Cc1c(cnc(c1O)C)COP(=O)(O)O)CC(=O)O
CN(C1C(=C(C(=O)N)C(=O)C2(C1C(O)C1C(=C2O)C(=O)c2c(C1(C)O)cccc2[O-])O)[O-])C
CCCCCCOC(=O)N=C(c1ccc(cc1)NCc1nc2c(n1C)ccc(c2)C(=O)N(c1ccccn1)CCC(=O)OCC)N
COc1ccc(cc1O)c1cc(=O)c2c(o1)cc(cc2O)OC1OC(COC2OC(C)C(C(C2O)O)O)C(C(C1O)O)O
O=C(N1CCCC(C1)(Cc1ccccc1)C(=O)N(N(C)C)C)C(NC(=O)C(N)(C)C)Cc1c[nH]c2c1cccc2
O=C(NC(C)(C)C)NC(C(C)(C)C)C(=O)N1CC2C(C1C(=O)NC(C(=O)C(=O)N)CC1CCC1)C2(C)C
N#CC1=CC2(C)C(C(C1=O)(C)C)CCC1(C2=CC(=O)C2C1(C)CCC1(C2CC(C)(C)CC1)C(=O)O)C
OC(=O)Cc1cc(Cl)c(cc1F)Oc1ccc(cc1NS(=O)(=O)c1ccc(cc1Cl)C1CC1)C(=O)NC(C)(C)C
OC(=O)C1CCN(CC1)c1ncc(cc1Cl)C(=O)Nc1sc(c(n1)c1scc(c1)Cl)N1CCN(CC1)C1CCCCC1
CCCCCCCCCCCCCCCCCC(=O)OC(COC(=O)CCCCCCCCCCCCCCCCC)COC(=O)CCCCCCCCCCCCCCCCC
OCC12C(O)CC(CC2(O)CCC2C1C(O)CC1(C2(O)CCC1C1=CC(=O)OC1)C)OC1OC(C)C(C(C1O)O)O
OC1C(CCCC1(COC(=O)c1cccnc1)COC(=O)c1cccnc1)(COC(=O)c1cccnc1)COC(=O)c1cccnc1
CON=C(c1csc(n1)N)C(=O)NC1C(=O)N2C1SCC(=C2C(=O)[O-])CSc1nc(c(s1)CC(=O)[O-])C
COC1(NC(=O)C2SC(=C(C(=O)N)C(=O)[O-])S2)C(=O)N2C1SCC(=C2C(=O)[O-])CSc1nnnn1C
COc1cc(cc(c1O)OC)C1C2C(=O)OCC2C(c2c1cc1OCOc1c2)OC1OC2COC(OC2C(C1O)O)c1cccs1
OC(=O)CC1NC(=O)CNC(=O)C(CCCNC(=N)N)NC(=O)C(N(C(=O)C(NC1=O)Cc1ccccc1)C)C(C)C
CC(CN(S(=O)(=O)c1ccc(cc1)N)CC(C(Cc1ccccc1)NC(=O)OC1CCOC1)OP(=O)([O-])[O-])C
COC1CC(C)(O)Cc2c1c(O)c1c(c2)C(=O)c2c(C1=O)c(O)cc1c2OC2OC1(C)C(C(C2O)N(C)C)O
OCC(C(=O)NC(C(=O)N1CCCC1C(=O)NC(C(=O)O)CCCNC(=N)N)CC(=O)O)NC(=O)C(CC(=O)O)N
CCOC(=O)C=CC(CC1CCNC1=O)NC(=O)C(Cc1ccc(cc1)F)CC(=O)C(C(C)C)NC(=O)c1noc(c1)C
O=C(C(S(=O)(=O)O)c1ccccc1)NC1C(=O)N2C1SCC(=C2C(=O)[O-])C[n+]1ccc(cc1)C(=O)N
OCC1OC(OP(=O)(OP(=O)(OCC2OC(C(C2O)O)n2ccc(=O)[nH]c2=O)[O-])[O-])C(C(C1O)O)O
COC(=O)C1C(OC)C(CC2C1CC1N(C2)CCc2c1[nH]c1c2cccc1)OC(=O)c1cc(OC)c(c(c1)OC)OC
C=CC1(C)CC(OC(=O)CSC(CNC(=O)C(C(C)C)N)(C)C)C2(C)C(C)CCC3(C(C1O)C)C2C(=O)CC3
O=C1CCC(=C1)N1CC2C(C1)C(C(CC2)OC(c1cc(cc(c1)C(F)(F)F)C(F)(F)F)C)c1ccc(cc1)F
N#CC1=CC2(C)C(C(C1=O)(C)C)CCC1(C2=CC(=O)C2C1(C)CCC1(C2CC(C)(C)CC1)C(=O)OC)C
CCC1N(C(C)C)c2nc(ncc2N(C1=O)C)Nc1ccc(cc1OC)C(=O)NC1CCC(CC1)N1CCN(CC1)CC1CC1
O=C(Nc1ccc(cc1)c1nc(nc(n1)N1CCOCC1)N1CCOCC1)Nc1ccc(cc1)C(=O)N1CCC(CC1)N(C)C
CONC(=O)Nc1ccc(cc1)c1sc2c(c1CN(C)C)c(=O)n(c(=O)n2Cc1c(F)cccc1F)c1ccc(nn1)OC
Oc1ccc(cc1)C(C(=O)NC1C(=O)N2C1SCC(=C2C(=O)[O-])CSc1nnnn1C)NC(=O)c1cnc(cc1O)C
CC(=CC(=O)OCC(=O)C1(O)CCC2C1(C)CC(O)C1C2CCC2=CC(=O)C=CC12C)CCC=C(CCC=C(C)C)C
O=C1C=CC2(C(=C1)C(F)CC1C2(F)C(O)CC2(C1CC1C2(OC(O1)(C)C)C(=O)COC(=O)C1CC1)C)C
CCCCCC(C1C(O)CC(O)CC(O)CC(O)CC(O)CC(O)CC(O)C(=CC=CC=CC=CC=CC(C(OC1=O)C)O)C)O
CCC(C(=O)C(C(C(CCc1ccc(c(c1C(=O)O)O)C)C)O)C)C1OC(CC1C)(CC)C1CCC(C(O1)C)(O)CC
CN1CCN(CC1)c1ncc(c(c1)c1ccccc1C)N(C(=O)C(c1cc(cc(c1)C(F)(F)F)C(F)(F)F)(C)C)C
Clc1ccc(cc1)Cn1c(CC(C(=O)[O-])(C)C)c(c2c1ccc(c2)OCc1ccc2c(n1)cccc2)SC(C)(C)C
OC(=O)COc1c(cc(cc1C(C)(C)C)SC(Sc1cc(c(c(c1)C(C)(C)C)O)C(C)(C)C)(C)C)C(C)(C)C
CCCC1(CCc2ccccc2)CC(=C(C(=O)O1)C(c1cccc(c1)NS(=O)(=O)c1ccc(cn1)C(F)(F)F)CC)O
COc1cccc(c1F)c1c(=O)n(CC(c2ccccc2)NCCCC(=O)O)c(=O)n(c1C)Cc1c(F)cccc1C(F)(F)F
C=CCNc1c(O)cc2c(c1CC(C)CC(OC)C(O)C(C)C=C(C)C(C(C=CC=C(C(=O)N2)C)OC)OC(=O)N)O
COc1ccc(cc1C(=O)O)CN(C(c1[nH]cc(n1)c1ccccc1)C)C(=O)C(Cc1c(C)cc(cc1C)C(=O)N)N
OCC1OC(OC2C(CO)OC(C(C2O)O)O)C(C(C1OC1OC(C)C(C(C1O)O)NC1C=C(CO)C(C(C1O)O)O)O)O
CC(=O)OC1CC2CCC3C(C2(CC1[N+]1(C)CCCCC1)C)CCC1(C3CC(C1OC(=O)C)[N+]1(C)CCCCC1)C
COc1cc(C=CC(=O)OC2CCC34C(C2(C)C)CCC2C4(C3)CCC3(C2(C)CCC3C(CCC=C(C)C)C)C)ccc1O
COc1ccc2c(c1)[nH]c1c2CCN2C1CC1C(C2)CC(C(C1C(=O)OC)OC)OC(=O)C=Cc1ccc(c(c1)OC)O
CCN1CCN(C(=O)C1=O)C(=O)NC(C(=O)NC1(OC)C(=O)N2C1SCC(=C2C(=O)O)CSc1nnnn1C)C(O)C
ClCCOC1=CC2=C(C=O)CC3C(C2(CC1)C)(F)C(O)CC1(C3CC2C1(OC(O2)(C)C)C(=O)COC(=O)C)C
ClCCN(c1ccc(cc1)CCCC(=O)OCC(=O)C1(O)CCC2C1(C)CC(O)C1C2CCC2=CC(=O)C=CC12C)CCCl
C=CC[N+]1(CCCCC1)C1CC2C(C1OC(=O)CC)(C)CCC1C2CCC2C1(C)CC(C(C2)OC(=O)C)N1CCCCC1
CCc1nc(c(n1Cc1ccc2c(c1)c(Br)c(o2)c1cccc(c1)[N-]S(=O)(=O)C(F)(F)F)C(=O)N)C1CC1
CCOC(=O)N1C(CC)CC(c2c1ccc(c2)C(F)(F)F)N(C(=O)OC)Cc1cc(cc(c1)C(F)(F)F)C(F)(F)F
O=C1C(NC(=O)C(=NOC(C(=O)O)(C)C)c2csc(n2)N)C2N1C(=C(CS2)C[n+]1ccccc1)C(=O)[O-]
CCCCCCCCCCCCCCCCCC(=O)OCC(=O)OCC(=O)C1(O)CCC2C1(C)CC(O)C1C2CCC2=CC(=O)C=CC12C
CCCCOCCOc1ccc(cc1)c1ccc2c(c1)C=C(CCCN2CC(C)C)C(=O)Nc1ccc(cc1)S(=O)Cc1cncn1CCC
Fc1ccc(cc1)CN1C2C3CCC(C2C(=C(C1=O)C1=NS(=O)(=O)c2c(N1)ccc(c2)NS(=O)(=O)C)O)C3
O=C(N1CC2(C1)OCc1c2ccc(c1)C1=NOC(C1)(c1cc(Cl)c(c(c1)Cl)F)C(F)(F)F)CS(=O)(=O)C
NCCCCC(C(=O)NC(C(=O)N)Cc1ccccc1)NC(=O)C(Cc1c(C)cc(cc1C)O)NC(=O)C(CCCNC(=N)N)N
O=C(C(S(=O)(=O)[O-])c1ccccc1)NC1C(=O)N2C1SCC(=C2C(=O)[O-])C[n+]1ccc(cc1)C(=O)N
Fc1ccc(c(c1)F)n1cc(C(=O)O)c(=O)c2c1nc(N1CC3C(C1)C3NC(=O)C(NC(=O)C(N)C)C)c(c2)F
NCCCCC(C(=O)OC(COc1cccc2c1c(=O)cc(o2)C(=O)OCC)COc1cccc2c1c(=O)cc(o2)C(=O)OCC)N
CC(CN(C(=O)NC(C)(C)C)CC(C(NC(=O)C(NC(=O)c1ccc2c(n1)cccc2)CC(=O)N)Cc1ccccc1)O)C
COc1cc(cc(c1OP(=O)(O)O)OC)C1C2C(=O)OCC2C(c2c1cc1OCOc1c2)OC1OC2COC(OC2C(C1O)O)C
COC1C(OC(C(C1O)O)C)OC1c2c(O)c3c(cc2C(=O)C(C1OC)(C)O)C(=O)c1c(C3=O)c(O)cc(c1)OC
O=C1C=CC2(C(=C1)CCC1C2(F)C(O)CC2(C1CC1C2(OC(O1)(C)C)C(=O)COP(=O)([O-])[O-])C)C
OC(=O)CCCOc1c(cc(cc1C(C)(C)C)SC(Sc1cc(c(c(c1)C(C)(C)C)O)C(C)(C)C)(C)C)C(C)(C)C
C=CCNC1=C2CC(C)CC(OC)C(O)C(C)C=C(C)C(C(C=CC=C(C(=O)NC(=CC1=O)C2=O)C)OC)OC(=O)N
COc1ccc2cc1Oc1cc3c(cc1OC)CCN(C3Cc1ccc(Oc3c4C(C2)[N+](C)(C)CCc4cc(c3OC)OC)cc1)C
O=C1C=CC2(C(=C1)CCC1C2C(O)CC2(C1CCC2(O)C(=O)COC(=O)c1cccc(c1)S(=O)(=O)[O-])C)C
Oc1cc(O)c2c(c1)oc(c(c2=O)OC1OC(COC2OC(C)C(C(C2O)O)O)C(C(C1O)O)O)c1ccc(c(c1)O)O
CCCC(=O)OCCC(=C(N(Cc1cnc(nc1N)C)C=O)C)SSC(=C(N(Cc1cnc(nc1N)C)C=O)C)CCOC(=O)CCC

Continued on next page

COc1ccc2c(c1)[nH]c1c2CCN2C1CC1C(C2)CC(C(C1C(=O)OC)OC)OC(=O)c1cc(OC)c(c(c1)OC)OC
CCCCCC(C1C(O)CC(O)CC(O)CC(O)CC(O)CC(O)C(O)C(O)C(=CC=CC=CC=CC=CC(C(OC1=O)C)O)C)O
CN1CC(CC2C1Cc1c[nH]c3c1c2ccc3)C(=O)NC1(C)OC2(N(C1=O)C(Cc1ccccc1)C(=O)N1C2CCC1)O
CCN(CCN(C(=O)Cn1c(SCc2ccc(cc2)F)nc(=O)c2c1CCC2)Cc1ccc(cc1)c1ccc(cc1)C(F)(F)F)CC
O=C(C(c1ccccc1)N)NC1C(=O)N2C1SC(C2C(=O)OCOC(=O)C1N2C(=O)CC2S(=O)(=O)C1(C)C)(C)C
O=C1C=CC2(C(=C1)CCC1C2(F)C(O)CC2(C1CC(C2(OC(=O)C1CC1)C(=O)COC(=O)C1CCCCC1)C)C)C
CCN1CCN(C(=O)C1=O)C(=O)NC(c1ccc(cc1)O)C(=O)NC1C(=O)N2C1SCC(=C2C(=O)O)CSc1nnnn1C
Oc1cc(O)c2c(c1)[o+]c(c(c2)OC1OC(COC2OC(C)C(C(C2O)O)O)C(C(C1O)O)O)c1ccc(c(c1)O)O
Brc1c(Br)c(Br)c2c(c1Br)C(=O)OC2(c1ccc(c(c1)S(=O)(=O)O)O)c1ccc(c(c1)S(=O)(=O)O)O
COc1cccc(c1F)c1c(=O)n(CC(c2ccccc2)NCCCC(=O)[O-])c(=O)n(c1C)Cc1c(F)cccc1C(F)(F)F
CN(C1C(=C(C(=O)N)C(=O)C2(C1CC1Cc3c(cc(c(c3C(=O)C1=C2O)O)CNCC(C)(C)C)N(C)C)O)O)C
CCOc1ccc(cn1)c1ccc(cc1)Cn1c(CC(C(=O)O)(C)C)c(c2c1ccc(c2)OCc1ccc(cn1)C)SC(C)(C)C
OC1C(O)C(OC1[n+]1cccc(c1)C(=O)N)COP(=O)(OP(=O)(OCC1OC(C(C1O)O)n1cnc2c1ncnc2N)O)O
CCN1CCN(C(=O)C1=O)C(=O)NC(C(=O)NC1(OC)C(=O)N2C1SCC(=C2C(=O)[O-])CSc1nnnn1C)C(O)C
O=C(N1CC2C3(C1=CC(=O)c1c3c(C)c[nH]1)C2)c1[nH]c2c(c1)cc(cc2)NC(=O)c1cc2c(o1)cccc2
O=C1C(=Cc2ccc(cc2)C=C2C3CCC(C2=O)(C3(C)C)CS(=O)(=O)O)C2C(C1(CC2)CS(=O)(=O)O)(C)C
CC[N+](=C1C=CC(=C(c2cc(ccc2S(=O)(=O)[O-])S(=O)(=O)[O-])c2ccc(cc2)N(CC)CC)C=C1)CC
O=CN(C(=C(SSC(=C(N(Cc1cnc(nc1N)C)C=O)C)CCOP(=O)(O)O)CCOP(=O)(O)O)C)Cc1cnc(nc1N)C
O=CCC1CC(C)C(=O)C=CC2(C)OC2C(C(OC(=O)CC(C(C1OC1OC(C)CC(C1OC(=O)CC)N(C)C)C)O)CC)C
CCC(n1ncn(c1=O)c1ccc(cc1)N1CCN(CC1)c1ccc(cc1)OCC1COC(O1)(Cn1cncn1)c1ccc(cc1F)F)C
COc1cc(C(=O)Nc2ccc(cc2)CCN2CCc3c(C2)cc(c(c3)OC)OC)c(cc1OC)NC(=O)c1cnc2c(c1)cccc2
COc1cccc(c1OC)C1OC(CC(=O)N2CCC(CC2)CC(=O)O)C(=O)N(c2c1cc(Cl)cc2)CC(COC(=O)C)(C)C
O=C(OC1CCC2(C(C1(C)C)CCC1(C2C(=O)C=C2C1(C)CCC1(C2CC(C)(CC1)C(=O)O)C)C)C)CCC(=O)O
COc1cc2CC[NH+](C3c2cc1Oc1cc(ccc1O)CC1c2c(CC[N+]1(C)C)cc(c(c2Oc1ccc(C3)cc1)O)OC)C
O=C(NCC(F)(F)F)CNC(=O)c1ccc(c2c1cccc2)C1=NOC(C1)(c1cc(Cl)cc(c1)C(F)(F)F)C(F)(F)F
O=C(Nc1cc(F)c2c(c1O)C(=O)C1=C(O)C3(C(CC1C2)C(N(C)C)C(=C(C3=O)C(=O)N)O)O)CN1CCCC1
OC(=O)C(NC(=O)c1c(Cl)cc2c(c1Cl)CCN(C2)C(=O)c1ccc2c(c1)occ2)Cc1cccc(c1)S(=O)(=O)C
OC(=O)C=Cc1ccc2c(c1)nc(n2C)C1(CCC1)NC(=O)c1ccc2c(c1)n(C)c(c2C1CCCC1)c1ncc(cn1)Br
Fc1ccc(cc1)C1C(OCCN1Cc1[nH]c(=O)n(n1)P(=O)(O)O)OC(c1cc(cc(c1)C(F)(F)F)C(F)(F)F)C
CSCCNc1nc(SCCC(F)(F)F)nc2c1ncn2C1OC(C(C1O)O)COP(=O)(OP(=O)(C(P(=O)(O)O)(Cl)Cl)O)O
OCC(Nn1c(=O)c2c(c1=O)c1c(c3c2c2ccc(cc2n3C2OC(CO)C(C(C2O)O)O)O)[nH]c2c1ccc(c2)O)CO
O=CCC1CC(C)C(=O)C=CC2(C)OC2C(C(OC(=O)CC(C(C1OC1OC(C)CC(C1OC(=O)CCC)N(C)C)C)O)CC)C
OC1CC(N(C1)C(=O)C1CCCN1C(=O)C(Cc1ccc(cc1)O)NC(=O)C)C(=O)NCC(=O)NC(C(=O)NCC(=O)N)C
COC(C1CC(C(O1)C(C=CC1CCC(C(O1)C(=CC1CCCC(C1C(C(=O)C1=C([O-])COC1=O)C)C)CO)C)C)C)C
COC1CC(C)CC2=C(NCCN(C)C)C(=O)C=C(C2=O)NC(=O)C(=CC=CC(C(C(=CC(C1O)C)C)OC(=O)N)OC)C
Fc1ccc(c(c1)C)C1CC(CCN1C(=O)N(C(c1cc(cc(c1)C(F)(F)F)C(F)(F)F)C)C)N1CCN(CC1)C(=O)C
Fc1ccc(c(c1)F)C1(COC(O1)COc1ccc(cc1)N1CCN(CC1)c1ccc(cc1)N1CCN(C1=O)C(C)C)Cn1cncn1
COc1ccc2c(c1)c1CCN3C(c1[nH]2)CC1C(C3)CC(C(C1C(=O)OC)OC)OC(=O)c1cc(OC)c(c(c1)OC)OC
OCCOc1cc(O)c2c(c1)oc(c(c2=O)OC1OC(COC2OC(C)C(C(C2O)O)O)C(C(C1O)O)O)c1ccc(c(c1)O)O
COc1cc(F)c(cc1c1ccc(cc1CN1C(=O)OC(C1C)c1cc(cc(c1)C(F)(F)F)C(F)(F)F)C(F)(F)F)C(C)C
O=C(C(Cc1cnc[nH]1)NC(=O)C(CS(=O)(=O)C(C)(C)C)Cc1ccccc1)NC(C(C(C1CC1)O)O)CC1CCCCC1
CN1CCN(CC1)C1CCN(CC1)C(=O)Nc1nccc(c1)Oc1ccc(c(c1)F)NC(=O)C1(CC1)C(=O)Nc1ccc(cc1)F
COc1ccc2c(c1)[nH]c1c2CCN2C1CC1C(C2)CC(C(C1C(=O)OC)OC)OC(=O)C=Cc1cc(OC)c(c(c1)OC)OC
CCC(n1ncn(c1=O)c1ccc(cc1)N1CCN(CC1)c1ccc(cc1)OCC1COC(O1)(Cn1cncn1)c1ccc(cc1Cl)Cl)C
CCN1CCN(C(=O)C1=O)C(=O)NC(c1ccc(cc1)O)C(=O)NC1C(=O)N2C1SCC(=C2C(=O)[O-])CSc1nnnn1C
O=CN(C(=C(SSC(=C(N(Cc1cnc(nc1N)C)C=O)C)CCOC(=O)C(C)C)CCOC(=O)C(C)C)C)Cc1cnc(nc1N)C
CCOC(=O)Oc1c(OC)cc(cc1OC)C(=O)OC1CC2CN3CCc4c(C3CC2C(C1OC)C(=O)OC)[nH]c1c4ccc(c1)OC
OCC(=O)C1(O)CC(OC2CC(N)C(C(O2)C)OC2CCCCO2)c2c(C1)c(O)c1c(c2O)C(=O)c2c(C1=O)cccc2OC
CCCC(=O)OCC(C(C(Cn1c2cc(C)c(cc2nc2c1nc(=O)[nH]c2=O)C)OC(=O)CCC)OC(=O)CCC)OC(=O)CCC
CC(CC(C(C(NC(=O)C(Cc1cscn1)NC(=O)C(CS(=O)(=O)N1CCN(CC1)C)Cc1ccccc1)CC1CCCCC1)O)O)C
CCCC1(CCc2ccccc2)CC(=C(C(=O)O1)C(c1cccc(c1)[N-]S(=O)(=O)c1ccc(cn1)C(F)(F)F)CC)[O-]
CCC(n1ncn(c1=O)c1ccc(cc1)N1CCN(CC1)c1ccc(cc1)OCC1COC(O1)(CSc1nncn1C)c1ccc(cc1)Cl)C
COC1CC(C)OC(C1O)OC1CCC(C)(O)C(=O)C=CC2OC2C(C(OC(=O)C=CC1C)C)COC1OC(C)C(C(C1OC)OC)O
CC(C(C(=O)N1CCCC1C(=O)N1CCCC1C(=O)NC(C)(C)C)N(C(=O)C(C(C)C)NC(=O)C(N(C)C)C(C)C)C)C
OC(=O)CCC(=O)Oc1c(cc(cc1C(C)(C)C)SC(Sc1cc(c(c(c1)C(C)(C)C)O)C(C)(C)C)(C)C)C(C)(C)C
O=C(c1ccccc1c1ccc(cc1)C(F)(F)F)NC1CCN(CC1)CCCCC1(C(=O)NCC(F)(F)F)c2ccccc2c2c1cccc2
Fc1ccc(c(c1)C)C1CC(CCN1C(=O)N(C(c1cc(cc(c1)C(F)(F)F)C(F)(F)F)C)C)N1CCN2C(C1)CCC2=O
OC(=O)C1CCC(CC1)CN1CCCC(c2c1c(C)cc(c2)C)N(c1nnn(n1)C)Cc1cc(cc(c1)C(F)(F)F)C(F)(F)F
CCOc1ccc(cn1)c1ccc(cc1)Cn1c(CC(C(=O)[O-])(C)C)c(c2c1ccc(c2)OCc1ccc(cn1)C)SC(C)(C)C
O=C(C1=C(O)C2C34C(C1)(O)C(Cc1c4c(O2)c(O)cc1)N(CC3)CC1CC1)NC(c1onc(n1)c1ccccc1)(C)C
N#CC1=CC2(C)C(C(C1=O)(C)C)CCC1(C2=CC(=O)C2C1(C)CCC1(C2CC(C)(C)CC1)NC(=O)C(F)(F)C)C
O=C(NC(C(CC(Cc1ccccc1)NC(=O)C(C(C)C)NC(=O)N(Cc1csc(n1)C(C)C)C)O)Cc1ccccc1)OCc1cncs1
NC(=O)CC(C(=O)NC(C(CN1CC2CCCCC2CC1C(=O)NC(C)(C)C)O)Cc1ccccc1)NC(=O)c1ccc2c(n1)cccc2
CC(=O)OC1CC2CCC3C(C2(CC1N1CC[N+](CC1)(C)C)C)CCC1(C3CC(C1OC(=O)C)N1CC[N+](CC1)(C)C)C
COC1C(OC(=O)N)C(O)C(OC1(C)C)Oc1ccc2c(c1C)oc(=O)c(c2[O-])NC(=O)c1ccc(c(c1)CC=C(C)C)O
O=C1C=CC2(C(=C1)CCC1C2=CCC2(C1CC(C2C(=O)CN1CCN(CC1)c1cc(nc(n1)N1CCCC1)N1CCCC1)C)C)C
CC(CN(S(=O)(=O)c1ccc2c(c1)OCO2)CC(C(Cc1ccc(cc1)OCc1csc(n1)C)NC(=O)OC1COC2C1CCO2)O)C
COc1ccc(cc1)CC(C(=O)NC(C(=O)NC(C(C(CC(C)C)O)O)CC1CCCCC1)Cc1c[nH]cn1)NC(=O)CC(N)(C)C
COC1C(N)C(OC(C1O)C)OC1C(OC(C(C1(C)O)O)C)Oc1cccc2c1c1oc(=O)c3c4c1c(c2O)c(=O)oc4ccc3C
CCCCCCCCCCCCCCCCC(=O)OCC(C1OCC(C1OC(=O)CCCCCCCCCCCCCCCCC)O)OC(=O)CCCCCCCCCCCCCCCCCC
OCCN1CCN(CC1)CNC(=O)C1=C(O)C(N(C)C)C2C(C1=O)(O)C(=C1C(C2)C(C)(O)c2c(C1=O)c(O)ccc2)O
CN1CC(CC2C1Cc1c[nH]c3c1c2ccc3)C(=O)NC1(OC2(N(C1=O)C(Cc1ccccc1)C(=O)N1C2CCC1)O)C(C)C
OCC(=O)C1(O)CC(OC2CC(N)C(C(O2)C)OCc2ccccc2)c2c(C1)c(O)c1c(c2O)C(=O)c2c(C1=O)cccc2OC
[O-]C(=O)C(NC(=O)c1c(Cl)cc2c(c1Cl)CCN(C2)C(=O)c1ccc2c(c1)occ2)Cc1cccc(c1)S(=O)(=O)C
[O-]C(=O)C=Cc1ccc2c(c1)nc(n2C)C1(CCC1)NC(=O)c1ccc2c(c1)n(C)c(c2C1CCCC1)c1ncc(cn1)Br
CCCCC1(CCCC)CN(c2ccccc2)c2c(S(=O)(=O)C1)cc(c(c2)SC)OCC(=O)NC(c1ccccc1)C(=O)NCC(=O)O
COc1ccc2cc1Oc1cc3c(cc1OC)CC[N+](C3Cc1ccc(Oc3c4C(C2)[N+](C)(C)CCc4cc(c3OC)OC)cc1)(C)C
CC1CC=CC=CC=CC=CC(OC2OC(C)C(C(C2O)N)O)CC2OC(CC(CC3C(C=CC(=O)O1)O3)O)(O)CC(C2C(=O)O)O
CC(OC(=O)C1=C(C)NC(=C(C1c1cccc(c1)[N+](=O)[O-])C(=O)OC1CN(C1)C(c1ccccc1)c1ccccc1)N)C
O=C1C=CC2(C(=C1)CCC1C2(F)C(O)CC2(C1CC(C2(O)C(=O)COC(=O)c1cccc(c1)S(=O)(=O)[O-])C)C)C

Continued on next page

CCC(n1ncn(c1=O)c1ccc(cc1)N1CCN(CC1)c1ccc(cc1)OCC1COC(C1)(Cn1cncn1)c1ccc(cc1F)F)C(O)C
CC(C=C(C1OC2(CC3CC(O2)CC=C(C)CC(C)C=CC=C2C4(C(C(=O)O3)C=C(C)C(C4OC2)O)O)CC(C1C)O)C)C
CC(CC1C(=O)N2CCCC2C2(N1C(=O)C(O2)(NC(=O)C1CN(C)C2C(C1)c1cccc3c1c(C2)c[nH]3)C(C)C)O)C
O=C(C1CN(C)C2C(=C1)c1cccc3c1c(C2)c[nH]3)NC1(C)OC2(N(C1=O)C(Cc1ccccc1)C(=O)N1C2CCC1)O
COC(=O)C1=C(C)NC(=C(C1c1cccc(c1)[N+](=O)[O-])C(=O)OCCN1CCN(CC1)C(c1ccccc1)c1ccccc1)C
Brc1c(Br)c(Br)c2c(c1Br)C(=O)OC2(c1ccc(c(c1)S(=O)(=O)[O-])O)c1ccc(c(c1)S(=O)(=O)[O-])O
CCN(CCn1c(=O)c2c(c1=O)c1c(c3c2c2cccc(c2[nH]3)Cl)n(c2c1cccc2Cl)C1OC(CO)C(C(C1O)O)OC)CC
CCCCCCN(C(=C1C(=O)OC(OC1=O)(C)C)Nc1cc(c(c(c1)C(C)(C)C)O)C(C)(C)C)Cc1ccc(cc1)CC(C)(C)C
CN(C(=O)C(c1ccccc1)NC(=O)c1cc2c(n1C)ccc(c2)NC(=O)c1ccccc1c1ccc(cc1)C(F)(F)F)Cc1ccccc1
NCCCCC(C(=O)NC(C(=O)NC(C(=O)NC(C(=O)O)Cc1ccc(cc1)O)C(C)C)CC(=O)O)NC(=O)C(CCCNC(=N)N)N
COc1cccc2c1C(=O)c1c(O)c3C(OC4CC(N)C(C(O4)C)O)CC(Cc3c(c1C2=O)O)(O)C(=NNC(=O)c1ccccc1)C
NC(C(=O)O)CCCCNCNC(=O)C1=C(O)C(N(C)C)C2C(C1=O)(O)C(=C1C(C2)C(C)(O)c2c(C1=O)c(O)ccc2)O
CCN(CCn1c2cc(OC)ccc2c2c1C1CC3C(CN1CC2)CC(C(C3C(=O)OC)OC)OC(=O)c1cc(OC)c(c(c1)OC)OC)CC
CCON=C(c1nsc(n1)NP(=O)(O)O)C(=O)NC1C(=O)N2C1SCC(=C2C(=O)[O-])Sc1scc(n1)c1cc[n+](cc1)C
OCC(C(C1OC(OC)(CC(C1NC(=O)C)O)C(=O)NC1CCC2(C(C1)CCC1C2CCC2(C1CCC2C(CCCC(C)C)C)C)C)O)O
O=C(NC(Cc1ccccc1)CCC(Cc1ccccc1)NC(=O)C(NC(=O)N(Cc1csc(n1)C(C)C)C)CCN1CCOCC1)OCc1cncs1
CCCCC1(CCCC)CS(=O)(=O)c2c(C(C1O)c1ccc(cc1)OCc1ccc(cc1)C[N+]13CCN(CC1)CC3)cc(cc2)N(C)C
O=C(Nc1cc(N(C)C)c2c(c1O)C(=O)C1=C(O)C3(C(CC1C2)C(N(C)C)C(=C(C3=O)C(=O)N)O)O)CNC(C)(C)C
COC(=O)C1=C(C)NC(=C(C1c1cccc(c1)[N+](=O)[O-])C(=O)OC(CN(CCC(c1ccccc1)c1ccccc1)C)(C)C)C
O=C(OC1CCC2(C(C1(C)C)CCC1(C2C(=O)C=C2C1(C)CCC1(C2CC(C)(CC1)C(=O)[O-])C)C)C)CCC(=O)[O-]
ClCCN(P(=O)(N(CCCl)CCCl)OCCS(=O)(=O)CC(C(=O)NC(c1ccccc1)C(=O)O)NC(=O)CCC(C(=O)O)N)CCCl
COCCN1CCC(CC1)N(C(=O)Cn1c(CCc2cccc(c2F)F)cc(=O)c2c1nccc2)Cc1ccc(cc1)c1ccc(cc1)C(F)(F)F
O=C(OCC(=O)C1(O)CCC2C1(C)CC(O)C1C2CC(C2=CC(=O)C=CC12C)C)CCCCCCC(=O)N(CCS(=O)(=O)[O-])C
COCCN1CCC(CC1)N(C(=O)Cn1c(SCc2cccc(c2F)F)cc(=O)c2c1cccc2)Cc1ccc(cc1)c1ccc(cc1)C(F)(F)F
CCCCCCCCC=CCCCCCCCC(=O)OCC(C1OCC(C1OC(=O)CCCCCCCC=CCCCCCCCC)O)OC(=O)CCCCCCCC=CCCC-CCCCC
COC(C(C1OC2(CCC(O2)(C)C2CCC(O2)(CC)C2OC(CC2C)C2OC(O)(CO)C(CC2C)C)CC(C1C)O)C)C(C(=O)O)C
CCN(CCS(=O)(=O)C1CCN2C1C(=O)OC(C(C)C)C(C)C=CC(=O)NCC=CC(=CC(CC(=O)Cc1nc(C2=O)co1)O)C)CC
CNC1C(OC2C(C=C3C2CC#CC2C(C#C3)(O2)C2COC(=O)O2)OC(=O)c2c(O)ccc3c2cc(OC)cc3C)OC(C(C1O)O)C
OC(C(Cc1ccccc1)NC(=O)C(C(C)C)NC(=O)c1ccc2c(n1)cccc2)CN1CCC(CC1C(=O)NC(C)(C)C)OCc1ccncc1
CON=C1CC2(CC3CC(O2)CC=C(C)CC(C)C=CC=C2C4(C(C(=O)O3)C=C(C)C(C4OC2)O)O)OC(C1C)C(=CC(C)C)C
CC(=O)OCC1(OC2OC(COC(=O)C)C(C(C2OC(=O)C)OC(=O)C)OC(=O)C)OC(C(C1OC(=O)C)OC(=O)C)COC(=O)C
OCCOc1cc(O)c2c(c1)oc(c(c2=O)OC1OC(COC2OC(C)C(C(C2O)O)O)C(C(C1O)O)O)c1ccc(c(c1)OCCO)OCCO
NCCC(C(CC(=O)NC1CNC(=O)C(NC(=O)C(=CNC(=O)N)NC(=O)C(NC(=O)C(NC1=O)CO)CO)C1CCNC(=N)N1)N)O
CCCC(C(=O)C(=O)NC1CC1)NC(=O)C1C2CCCC2CN1C(=O)C(C(C)(C)C)NC(=O)C(C1CCCCC1)NC(=O)c1nccnc1
COc1ccc2c(c1)C1CC1(Cn1c2c(C2CCCCC2)c2c1cc(cc2)C(=O)NS(=O)(=O)N(C)C)C(=O)N1C2CCC1CN(C2)C
O=CN(C(=C(SSC(=C(N(Cc1cnc(nc1N)C)C=O)C)CCOC(=O)c1ccccc1)CCOC(=O)c1ccccc1)C)Cc1cnc(nc1N)C
CC1=C(C(=O)OCCN(c2ccccc2)Cc2ccccc2)C(C(=C(N1)C)P1(=O)OCC(CO1)(C)C)c1cccc(c1)[N+](=O)[O-]
CCC(C(N(C(=O)C(C(C)C)NC(=O)C(N(C)C)C(C)C)C)C(CC(=O)N1CCCC1C(C(C(=O)NCCc1ccccc1)C)OC)OC)C
COC1CC(OC2C(C)C(=O)OC(C)C(C)C(O)C(C(=O)C3(CC(C(C2C)OC2OC(C)CC(C2O)N(C)C)C)CO3)C)OC(C1O)C
Oc1cc(cc2c1c(N=Nc1ccc(c3c1cccc3S(=O)(=O)[O-])Nc1ccccc1)cc(c2)S(=O)(=O)[O-])S(=O)(=O)[O-]
Clc1ccc2c(c1)c(CCOc1ccc(cc1)C(=O)O)c(n2C(c1ccccc1)c1ccccc1)CCNS(=O)(=O)Cc1ccc(c(c1)Cl)Cl
COc1cc2cc([nH]c2c(c1OC)OC)C(=O)N1CC(c2c1cc(OC(=O)N1CCN(CC1)C)c1c2c(C(=O)OC)c([nH]1)C)CBr
Clc1ccc2c(c1)c(CCCc1ccc(cc1)C(=O)O)c(n2C(c1ccccc1)c1ccccc1)CCNS(=O)(=O)Cc1ccccc1C(F)(F)F
Clc1ccc2c(c1)c(CCCc1ccc(cc1)C(=O)O)c(n2C(c1ccccc1)c1ccccc1)CCNS(=O)(=O)Cc1ccc(c(c1)Cl)Cl
CCC1OC(=O)CC(O)C(C)C(OC2OC(C)C(C(C2O)N(C)C)O)C(CCN2CCCCC2)CC(C(=O)C=CC(=CC1CN1CCCCC1)C)C
COc1cc(NC(=O)CCN2CCC(CC2)OC(=O)Nc2ccccc2c2ccccc2)c(cc1CNCC(c1ccc(c2c1ccc(=O)[nH]2)O)O)Cl
CC(CC1C(=O)N2CCCC2C2(N1C(=O)C(O2)(NC(=O)C1CN(C)C2C(=C1)c1cccc3c1c(C2)c([nH]3)Br)C(C)C)O)C
CSCCC(C(=O)NC(C(=O)NC(C(=O)N)Cc1ccccc1)CC(=O)O)NC(=O)C(Cc1c[nH]c2c1cccc2)NC(=O)OC(CC)(C)C
COC(=O)NC(C(C)(C)C)C(=O)NN(Cc1ccc(cc1)c1ccccn1)CC(C(Cc1ccccc1)NC(=O)C(C(C)(C)C)NC(=O)OC)O
Oc1c(cc(cc1n1nc2c(n1)cccc2)C(CC(C)(C)C)(C)C)Cc1cc(cc(c1O)n1nc2c(n1)cccc2)C(CC(C)(C)C)(C)C
OC(=O)CN(C(CN(CC(=O)O)CCN(CC(=O)O)CC(=O)O)COP(=O)(OC1CCC(CC1)(c1ccccc1)c1ccccc1)O)CC(=O)O
CC(CC(C(=O)NC(C(=O)NC(C(=O)C1(C)CO1)CC(C)C)Cc1ccccc1)NC(=O)C(NC(=O)CN1CCOCC1)CCc1ccccc1)C
NCCNC(=O)Nc1c[n+](n(c1N)C)CC1=C(C(=O)O)N2C(SC1)C(C2=O)NC(=O)C(=NOC(C(=O)O)(C)C)c1nsc(n1)N
CNCC(=O)OCc1cccnc1N(C(=O)OC([n+]1cnn(c1)CC(c1cc(F)ccc1F)(C(c1scc(n1)c1ccc(cc1)C#N)C)O)C)C
CC(CCCC1(C)CCc2c(O1)c(C)c(c(c2C)OC(=O)C=C(C=CC=C(C=CC1=C(C)CCCC1(C)C)C)C)C)CCCC(CCCC(C)C)C
CC(CC(C(CC(=O)NC(C(=O)NC(C(CC(=O)O)O)CC(C)C)C)O)NC(=O)C(C(C)C)NC(=O)C(C(C)C)NC(=O)CC(C)C)C
CCN(C1CC(C)OC(C1O)OC1C(C)C(OC2OC(C)CC(C2)(C)OC)C(C)C(=O)OC(C(C(C(C2=C(CC1(C)O2)C)C)O)C)CC)C
ON=C(c1nsc(n1)N)C(=O)NC1C(=O)N2C1SCC(=C2C(=O)[O-])C=C1CCN(C1=O)C1CCN(C1)C(=O)OCc1oc(=O)oc1C
CNC(C(=O)NC(C(=O)N(C(C(CC(=O)N1CCCC1C(C(C(=O)NC(C(c1ccccc1)O)C)C)OC)OC)C(CC)C)C)C(C)C)C(C)C
CCCCC(COC(=O)c1ccc(cc1)Nc1nc(Nc2ccc(cc2)C(=O)OCC(CCCC)CC)nc(n1)Nc1ccc(cc1)C(=O)NC(C)(C)C)CC
CCC1=C(C)C2=Cc3[n-]c(c(c3CC)C)C=C3N=C(C=c4[n-]c(=CC1=N2)c(C)c4CCC(=O)[O-])C(=C3C)CCC(=O)[O-]
OCC1OC(OC2CCC3(C(C2)CCC2C3CCC3(C2CC2C3C(C)C3(O2)CCC(CO3)C)C)C)C(C(C1OC1OC(CO)C(C(C1O)O)O)O)O
OS(=O)(=O)c1cc(cc2c1[n-]c(n2)c1ccc(cc1)c1nc2c([n-]1)c(cc(c2)S(=O)(=O)O)S(=O)(=O)O)S(=O)(=O)O
NCCNC(=O)Nc1c[n+](n(c1N)C)CC1=C(C(=O)[O-])N2C(SC1)C(C2=O)NC(=O)C(=NOC(C(=O)O)(C)C)c1nsc(n1)N
O=C(C(c1ccccc1)NC(=O)c1[nH]cnc1C(=O)O)NC1C(=O)N2C1SCC(=C2C(=O)[O-])C[n+]1ccc(cc1)CCS(=O)(=O)O
C=CC1=C(C)C2=Cc3[nH]c(c(c3C=C)C)C=C3N=C(C=c4[nH]c(=CC1=N2)c(C)c4CCC(=O)[O-])C(=C3C)CCC(=O)[O-]
COC1C=COC2(C)Oc3c(C2=O)c2c(O)cc(c(c2c(c3C)O)O)NC(=O)C(=CC=CC(C(C(C(C(C(C1C)OC(=O)C)C)O)C)O)C)C
CCC1OC(=O)C(C)C(OC2CC(C)(OC)C(C(O2)C)O)C(C)C(OC2OC(C)CC(C2O)N(C)C)C(CC(C(=O)C(C(C1C)O)C)C)(C)O
NCC(=O)NCC(=O)NC(C(=O)NC(C(=O)NC(C(=O)NC(C(=O)N1CCCC1C(=O)NCC(=O)O)CCC(=O)N)C(C)C)CC(C)C)-C(C)C
COC1C=CC=C(C)Cc2cc(OC)c(c(c2)N(C(=O)CC(C2(C(C(C3CC1(O)NC(=O)O3)C)O2)C)OC(=O)C(N(C(=O)C)C)C)C)Cl
NCC(CC1OC2C(C1OC)CC(=O)CC1CCC3C(O1)C1OC4(CCC5OC(CCC6OC(C2)C(=C)C(C6)C)C(=C)C5)OC2C(O3)C1OC-2C4)O
C=CC[N+]12CCC34C2CC(C(=CCO)C1)C1=CN2c5ccccc5C56C2C(=CN(C31)c1c4cccc1)C1CC6[N+](CC1=CCO)(CC5)CC=-C
O=C(C(c1ccccc1)NC(=O)c1[nH]cnc1C(=O)O)NC1C(=O)N2C1SCC(=C2C(=O)[O-])C[n+]1ccc(cc1)CCS(=O)(=O)[O-]
CSCCC(C(=O)NC(C(=O)NC(C(=O)N)Cc1ccccc1)CC(=O)O)NC(=O)C(Cc1c[nH]c2c1cccc2)NC(=O)CCNC(=O)OC(C)(C)C
CCCCC(=O)OCC(=O)C1(O)CC(OC2CC(NC(=O)C(F)(F)F)C(C(O2)C)O)c2c(C1)c(O)c1c(c2O)C(=O)c2c(C1=O)cccc2OC
COc1ccc2c(c1)nc(cc2OC1CC2N(C1)C(=O)C(CCCCCC=CC1C(NC2=O)(C1)C(=O)O)NC(=O)OC1CCCC1)c1csc(n1)NC(C)-C

Continued on next page

OCC1OC(OC2CCC3(C(C2)CCC2C3C(=O)CC3(C2CC2C3C(C)C3(O2)CCC(CO3)C)C)C)C(C(C1OC1OC(CO)C(C(C1O)O)O)O)O
COc1cc2c(cc1O)CCNC12CSC2c3c(OC(=O)C)c(C)c4c(c3C(COC1=O)N1C2C2N(C(C1O)Cc1c2c(O)c(c(c1)C)OC)C)OCO4
CCCCC(C(=O)C(=O)NC1CC1)NC(=O)C1N(CC2C1C2(C)C)C(=O)C(C(C)(C)C)NC(=O)NC1(CCCCC1)CS(=O)(=O)C(C)(C)C
COc1ccc2c(c1C)nc(cc2OC1CC2C(C1)C(=O)N(C)CCCCC=CC1C(NC2=O)(C1)C(=O)NS(=O)(=O)C1CC1)c1scc(n1)C(C)C
CCC1OC(=O)C(C)C(OC2OC(C)C(C(C2)(C)OC)O)C(C)C(OC2OC(C)CC(C2O)N(C)C)C(CC(C(=O)C(C(C1(C)O)O)C)C)(C)O
COC1CC(OC(C1O)C)OC1C(C)C=CC=C2COC3C2(O)C(C=C(C3=NO)C)C(=O)OC2CC(CC=C1C)OC1(C2)CCC(C(O1)C1CCCCC1)C
CCC1OC(=O)C(C)C(OC2OC(C)C(C(C2)(C)OC)O)C(C)C(OC2OC(C)CC(C2O)N(C)C)C(CC(CN(C(C(C1(C)O)O)C)C)C)(C)O
O=C(OC(C)(C)C)NC1CCCCCC=CC2C(NC(=O)C3N(C1=O)CC(C3)OC(=O)N1Cc3c(C1)cccc3F)(C2)C(=O)NS(=O)(=O)C1CC1
O=C(C(Cc1c[nH]c2c1cccc2)NC(=O)C(Cc1csc2c1cccc2)NC(=O)C1CCCNC1)NC(C(=O)NC1(CCNCC1)C(=O)N)Cc1ccccc1
CCC1OC(=O)C(C)C(OC2CC(C)(OC)C(C(O2)C)O)C(C)C(OC2OC(C)CC(C2O)N(C)C)C(CC(C(=O)C(C(C1(C)O)O)C)C)(C)OC
CCCN(C(=O)C(NC(=O)c1ccccc1)CCC(=O)OCCCN1CCN(CC1)CCOC(=O)Cc1c2cc(OC)ccc2n(c1C)C(=O)c1ccc(cc1)Cl)CCC
COC1C(O)CC(OC1C)OC1C(O)CC(OC1C)OC1C(O)CC(OC1C)OC1CCC2(C(C1)CCC1C2CC(O)C2(C1(O)CCC2C1=CC(=O)OC1)C)C
Cc1ncc(nc1)C(=O)NC1CCCCCC=CC2C(NC(=O)C3N(C1=O)CC(C3)Oc1nc3ccccc3c3c1cccc3)(C2)C(=O)NS(=O)(=O)C1CC1
CCCCC1(CC)CS(=O)(=O)c2c(C(C1O)c1ccc(cc1)NC(=O)NC1OC(COS(=O)(=O)O)C(C(C1O)OCc1ccccc1)O)cc(cc2)N(C)C
CCCCCCCCCCCCCCCC(=O)NCCCCC(C(=O)O)NC(=O)CCC(C(=O)N)NC(=O)C(NC(=O)C(OC1C(NC(=O)C)C(O)OC(C1O)CO)C)C
OCC(C(C(C(C(S(=O)(=O)[O-])Nc1ccc(cc1)S(=O)(=O)c1ccc(cc1)NC(S(=O)(=O)[O-])C(C(C(C(CO)O)O)O)O)O)O)O)O
CON=C1C(C)CC(C)(O)C(OC2OC(C)CC(C2O)N(C)C)C(C)C(OC2OC(C)C(C(C2)(C)OC)O)C(C(=O)OC(C(C(C1C)O)(C)O)CC)C
CCCN1CC(C)C(O)C(C)(O)C(CC)OC(=O)C(C(C(C(C(CC1C)(C)O)OC1OC(C)CC(C1O)N(C)C)C)OC1OC(C)C(C(C1)(C)OC)O)C
C=CC1CC1(NC(=O)C1CC(CN1C(=O)C(C(C)(C)C)NC(=O)OC(C)(C)C)Oc1ncc(c2c1cc(Cl)cc2)OC)C(=O)NS(=O)(=O)C1CC1
OCC(=NNC(=O)CCCCCN1C(=O)C=CC1=O)C1(O)CC(OC2CC(N)C(C(O2)C)O)c2c(C1)c(O)c1c(c2O)C(=O)c2c(C1=O)cccc2OC
COc1ccc2c(c1C)nc(cc2OC1CC2C(C1)C(=O)N(C)CCCCC=CC1C(NC2=O)(C1)C(=O)[N-]S(=O)(=O)C1CC1)c1scc(n1)C(C)C
C=CC1CC1(NC(=O)C1CC2CN1C(=O)C(NC(=O)OC1CC1CCCCc1c(O2)nc2cc(OC)ccc2n1)C(C)(C)C)C(=O)NS(=O)(=O)C1CC1
O=C1OCC(=C1)C1CCC2(C1(C)CCC1C2CCC2C1(C)CCC(C2)OC1CC(O)C(C(O1)C)OC1CC(O)C(C(O1)C)OC1CC(O)C(C(O1)C)O)O
O=C(c1cccnc1)OC1C(OC(=O)c2cccnc2)C(OC(=O)c2cccnc2)C(C(C1OC(=O)c1cccnc1)OC(=O)c1cccnc1)OC(=O)c1cccnc1
CCC1OC(=O)C(C)C(OC2OC(C)C(C(C2)(C)OC)O)C(C)C(C2(OC(=C(C)C2)C(C(C1(C)O)O)C)C)OC1OC(C)CC(C1O)N(C(C)C)C
CC(=O)OC1CC(OC2C(O)CC(OC2C)OC2C(O)CC(OC2C)OC2CCC3(C(C2)CCC2C3CCC3(C2(O)CCC3C2=CC(=O)OC2)C)C)OC(C1O)C
CCC1OC(=O)C(C)C(OC2OC(C)C(C(C2)(C)OC)O)C(C)C(OC2OC(C)CC(C2O)N(C)C)C(CC(C(=O)C(C(C1(C)O)O)C)(C)F)(C)O
CCC(C1OC2(C=CC(C3(O2)CCC(O3)(C)C2CCC(C(O2)C)(O)CC)O)C(CC1C)C)C(=O)C(C(C(C1OC(CCC1C)C(C(=O)O)C)C)C)O)C
CCC1CC1(NC(=O)C1CCC2N1C(=O)C(NC(=O)OCC(C)(C)CCCCc1c3CN(C(=O)O2)Cc3ccc1)C(C)(C)C)C(=O)NS(=O)(=O)C1CC1
COC(=O)NC(C(=O)N1CCCC1c1ncc([nH]1)c1ccc(cc1)c1ccc(cc1)c1cnc([nH]1)C1CCCN1C(=O)C(C(C)C)NC(=O)OC)C(C)C
O=CCC1CC(C)C(O)C=CC=CCC(OC(=O)CC(C(C1OC1OC(C)C(C(C1O)N(C)C)OC1OC(C)C(C(C1)(C)O)OC(=O)CC)OC)OC(=O)CC)C
ClCC1CN(c2c1c1c(C)c[nH]c1c(c2)OC(=O)Nc1ccccc1)C(=O)c1cc2c([nH]1)ccc(c2)NC(=O)c1cc2c(o1)cc(cc2)N(CC)CC
O=C(N1CC(C1=O)NC(=O)C(=NOC(C(=O)[O-])(C)C)c1csc(n1)N)NS(=O)(=O)N1CCN(C1=O)NC(=O)c1[nH]cc(c(=O)c1)[O-]
OCCN(S(=O)(=O)c1ccc(cc1)c1ccc(c(=O)[nH]1)C(=O)NC(c1ccc(cc1)O)C(=O)NC1C(=O)N2C1SC(C2C(=O)[O-])(C)C)CCO
OC(=O)CC1NC(=O)CNC(=O)C(CCCCNC(=N)N)NC(=O)CCSSCC(NC(=O)C2N(C(=O)C(NC1=O)Cc1c[nH]c3c1cccc3)CCC2)C(=O)N
CCCCC1(CC)CS(=O)(=O)c2c(C(C1O)c1ccc(cc1)NC(=O)NC1OC(COS(=O)(=O)[O-])C(C(C1O)OCc1ccccc1)O)cc(cc2)N(C)C
O=c1[nH]c(=O)c2c(n1)n(CC(C(C(COP(=O)(OP(=O)(OCC1OC(C(C1O)O)n1cnc3c1ncnc3N)O)O)O)O)O)c1c(n2)cc(c(c1)C)C
O=CCC1CC(C)C(O)C=CC=CCC(OC(=O)CC(C(C1OC1OC(C)C(C(C1O)N(C)C)OC1OC(C)C(C(C1)(C)OC(=O)CC)OC(=O)CCC)OC)O)C
CCC1OC(=O)C(C)C(=O)C(C)C(OC2OC(C)CC(C2O)N(C)C)C(CC(C(=O)C(C2C1(C)OC(=O)N2)C)C)(C)OCC=Cc1cnc2c(c1)cccc2
CCC1=C(C)C2=Cc3[n-]c(c(c3C=C)C)C=C3N=C(C(=c4[n-]c(=CC1=N2)c(C)c4C(=O)[O-])CC(=O)[O-])C(CCC(=O)[O-])C3C
O=C1OCC(=C1)C1CCC2(C1(C)C(O)CC1C2CCC2C1(C)CCC(C2)OC1CC(O)C(C(O1)C)OC1CC(O)C(C(O1)C)OC1CC(O)C(C(O1)C)O)O
[O-]S(=O)(=O)CCCCN1c2ccc3c(c2C(C1=CC=CC=CC=CC1=[N+](CCCCS(=O)(=O)[O-])c2c(C1(C)C)c1ccccc1cc2)(C)C)cccc3
COc1cc2N(C)C3C4(c2cc1C1(CC2CN(CCc5c1[nH]c1c5cccc1)CC(C2)(O)CC)C(=O)OC)CCN1C4C(C(C3(O)C(=O)N)O)(CC)C=CC1
CCC1OC(=O)C(C)C(OC2OC(C)C(C(C2)(C)OC)O)C(C)C(OC2OC(C)CC(C2OC(=O)C)N(C)C)C(CC(C(=O)C(C(C1(C)O)O)C)C)(C)O
CCC(C1OC2(C=CC(C3(O2)CCC(O3)(C)C2CCC(C(O2)C)(O)CC)O)C(CC1C)C)C(=O)C(C(C(C1OC(C(CC1C)C)C(C(=O)O)CC)C)O)C

Continued on next page

Table S8 – *Continued from previous page*

CCC1C=C(C)CC(C)CC(OC)C2OC(O)(C(CC2OC)C)C(=O)C(=O)N2C(C(=O)OC(C(C(CC1=O)O)C)C(=CC1CCC(C(C1)-OC)Cl)C)CCCC2
Oc1cccc(c1)C1=C2CCC(=N2)C(=c2ccc(=C(C3=NC(=C(c4[nH]c1cc4)c1cccc(c1)O)C=C3)c1cccc(c1)O)[nH]2)c1cccc(c1)O
CC(=O)OC1CC(OC2C(O)CC(OC2C)OC2C(O)CC(OC2C)OC2CCC3(C(C2)CCC2C3CC(O)C3(C2(O)CCC3C2=CC(=O)OC-2)C)C)OC(C1O)C
CC(=O)OC1C(O)CC(OC1C)OC1C(O)CC(OC1C)OC1C(O)CC(OC1C)OC1CCC2(C(C1)CCC1C2CC(O)C2(C1(O)CCC2C1=-CC(=O)OC1)C)C
C=CCC1C=C(C)CC(C)CC(OC)C2OC(O)(C(CC2OC)C)C(=O)C(=O)N2C(C(=O)OC(C(C(CC1=O)O)C)C(=CC1CCC(C(C-1)OC)O)C)CCCC2
CCC(=O)OC1C(OC(CC1N(C)C)C)OC1C(C)C(OC2OC(C)C(C(C2)(C)OC)O)C(C)C(=O)OC(CC)C(C(C(C(=O)C(CC1(C)O-)C)C)O)(C)O
CCC1OC(=O)C(C)C(=O)C(C)C(OC2OC(C)CC(C2O)N(C)C)C(CC(C(=O)C(C2C1(C)OC(=O)N2CCCCn1cnc(c1)c1cccnc1)-C)C)(C)OC
O=CCC1CC(C)C(O)C=CC=CCC(OC(=O)CC(C(C1OC1OC(C)C(C(C1O)N(C)C)OC1OC(C)C(C(C1)(C)O)OC(=O)CC(C)-C)OC)OC(=O)C)C
COCCOCON=C1C(C)CC(C)(O)C(OC2OC(C)CC(C2O)N(C)C)C(C)C(OC2OC(C)C(C(C2)(C)OC)O)C(C(=O)OC(C(C(C1C-)O)(C)O)CC)C
NCCCCC1NC(=O)C(NC(=O)C(Cc2ccc(cc2)O)NC(=O)C(N(C(=O)C(NC(=O)C(NC1=O)C(C)C)Cc1ccccc1)C)C)Cc1c[nH]c2-c1cccc2
COCCOCC1NC2C(C)CC(C)(O)C(OC3OC(C)CC(C3O)N(C)C)C(C)C(C(C(=O)OC(C(C(O1)C2C)(C)O)CC)C)OC1OC(C)C(-C(C1)(C)OC)O
CCC(=O)OCC1(O)OC(C(CC1C)C)C1CC(C(O1)C1(C)CCC(O1)C1(C)CCC2(O1)CC(O)C(C(O2)C(C(C(C(=O)[O-])C)OC(=-O)CC)C)C)C
COC(=O)C1c2cc3c(c(c2C(CC1(C)O)OC1OC(C)C(C(C1OC)(C)OC)OC)O)C(=O)c1c(C3=O)c2OC3OC(c2cc1O)(C)C(C(C3O-)N(C)C)O
CCC1C=CC=CCC(C)C(O)C(C)(O)C(=O)C(C)C(O)C(C)C(=O)C(C(C(C=CC(=O)OC2C(C(CC1)OC1(CCC(C(O1)CC(O)-C)C)C2)C)C)O)C
COC1CC(OC(C1OC(=O)C)C)OC1C(C)C(OC2OC(C)CC(C2OC(=O)C)N(C)C)C(C)CC2(OC2)C(=O)C(C(C(C(OC(=O)C1-C)C)C)OC(=O)C)C
COc1ccc2c(c1)nc(cc2OC1CC(N(C1)C(=O)C(C(C)(C)C)CC(=O)N1CCCCC1)C(=O)NC1(CC1C=C)C(=O)NS(=O)(=O)C1C-C1)c1ccccc1
CCC1OC(=O)CC(O)C(C)C(OC2OC(C)C(C(C2O)N(C)C)O)C(CCN2CC(C)CC(C2)C)CC(C(=O)C=CC(=CC1COC1OC(C)-C(C(C1OC)OC)O)C)C
COC1C=COC2(C)Oc3c(C2=O)c2c(c(c3C)O)c(O)c(c3c2nc(s3)N(CC)CC)NC(=O)C(=CC=CC(C(C(C(C(C(C1C)OC(=O)C)-C)O)C)O)C)C
COC1C=COC2(C)Oc3c(C2=O)c2c(OCC(=O)N(CC)CC)cc(c(c2c(c3C)O)O)NC(=O)C(=CC=CC(C(C(C(C(C(C1C)OC(=O)-C)C)O)C)O)C)C
COC1C=COC2(C)Oc3c(C2=O)c2c(c(c3C)O)c(O)c(c3c2nc2n3ccc(c2)C)NC(=O)C(=CC=CC(C(C(C(C(C(C1C)OC(=O)C)C)-O)C)O)C)C
[O-]C(=O)CN(C(CN(CC(=O)[O-])CCN(CC(=O)[O-])CC(=O)[O-])COP(=O)(OC1CCC(CC1)(c1ccccc1)c1ccccc1)[O-])CC(=-O)[O-]
CCC1=CC2CN(C1)Cc1c3ccccc3[nH]c1C(C2)(C(=O)OC)c1cc2c(cc1OC)N(C1C32CCN2C3C(CC)(C=CC2)C(C1(O)C(=O)OC-)OC(=O)C)C
C=CC1CC1(NC(=O)C1CC(CN1C(=O)C(C(C)(C)C)NC(=O)OC1CCCC1)Oc1cc(nc2c1ccc(c2Br)OC)c1csc(n1)NC(=O)C(C)-C)C(=O)[O-]
COC(=O)C1c2cc3c(c(c2C(CC1(O)CC)OC1OC(C)C(C(C1)N(C)C)OC1CC(O)C(C(O1)C)OC1CCC(=O)C(O1)C)O)C(=O)c1-c(C3=O)cccc1O
O=c1[nH]c(=O)c2c(n1)n(CC(C(C(COP(=O)(OP(=O)(OCC1OC(C(C1O)O)n1cnc3c1ncnc3N)[O-])[O-])O)O)O)c1c(n2)cc(c(c-1)C)C
OCC1OC(Oc2ccc(cc2c2cccc(c2)CC(=O)[O-])CCCCCCc2ccc(c(c2)c2cccc(c2)CC(=O)[O-])OC2OC(CO)C(C(C2O)O)O)C(C(C-1O)O)O
Nc1ccn(c(=O)n1)C1OC(C(C1)O)COP(=O)(OP(=O)(OP(=O)(OP(=O)(OCC1OC(C(C1O)O)n1ccc(=O)[nH]c1=O)[O-])[O-])-[O-])[O-]
OCC(C(=O)NC(C(=O)N1CCCC1C(=O)NC(C(=O)O)CCC(=O)N)C(CC)C)NC(=O)C(C(C)C)NC(=O)C1CCCN1C(=O)C(-NC(=O)C(CC(=O)N)N)C
CCC(C(=O)N1CC(CC1Cc1c([nH]c2c1ccc(c2)F)c1[nH]c2c(c1CC1CC(CN1C(=O)C(NC(=O)C(NC)C)CC)O)ccc(c2)F)O)NC(=-O)C(NC)C
CCC1OC(=O)C(C)C(=O)C(C)C(OC2OC(C)CC(C2O)N(C)C)C2(CC(C(=NC(=O)C)C(C(C1(C)O)OCC(=NOCc1ccc(nc1)n1-cccn1)CO2)C)C)C
COC1C=COC2(C)Oc3c(C2=O)c2c(O)c(C=NN4CCN(CC4)C)c(c(c2c(c3C)O)O)NC(=O)C(=CC=CC(C(C(C(C(C(C1C)OC(-=O)C)C)O)C)O)C)C
COc1cc2N(C)C3C4(c2cc1C1(CC2CN(CCc5c1[nH]c1c5cccc1)CC(C2)(O)CC)C(=O)OC)CCN1C4C(C(C3(O)C(=O)OC)OC(=-O)C)(CC)C=CC1
COc1cc(ccc1[n+]1nc(nn1c1ccc(cc1)[N+](=O)[O-])c1ccccc1)c1ccc(c(c1)OC)[n+]1nc(nn1c1ccc(cc1)[N+](=O)[O-])c1ccccc1
OCC1OC(Oc2cccc3c2C(=O)c2c(C3C3c4cc(cc(c4C(=O)c4c3cccc4OC3OC(CO)C(C(C3O)O)O)O)C(=O)O)cc(cc2O)C(=O)O)-C(C(C1O)O)O
CC(=O)OC12COC1CC(C1(C2C(OC(=O)c2ccccc2)C2(O)CC(OC(=O)C(C(c3ccccc3)NC(=O)OC(C)(C)C)O)C(=C(C2(C)C)-C(C1=O)O)C)C)O
CCCCCCCCC=CCCCCCCCC(=O)Oc1c(OC(=O)CCCCCCCC=CCCCCCCCC)cc(cc1OC(=O)CCCCCCCC=CCCCCCCCC-)C(=O)Oc1cc(cc(c1O)O)C(=O)O
CCC1OC(=O)C(C)(F)C(=O)C(C)C(OC2OC(C)CC(C2O)N(C)C)C(CC(C(=O)C(C2C1(C)OC(=O)N2CCCCn1nnc(c1)c1cccc-(c1)N)C)C)(C)OC
O=CCC1CC(C)C(C=CC=CCC(OC(=O)CC(C(C1OC1OC(C)C(C(C1O)N(C)C)OC1CC(C)(O)C(C(O1)C)O)OC)OC(=O)C)-C)OC1CCC(C(O1)C)N(C)C
O=CCC1CC(C)C(C=CC=CCC(OC(=O)CC(C(C1OC1OC(C)C(C(C1O)N(C)C)OC1OC(C)C(C(C1)(C)O)OC(=O)CC(C)C)-OC)OC(=O)C)C)OC(=O)CC
CCC1CN2CCc3c(C(CC(C1)C2)(C(=O)OC)c1cc2c(cc1OC)N(C1C42CCN2C4C(CC)(C=CC2)C(C1(O)C(=O)OC)OC(=O)C)-C=O)[nH]c1c3cccc1
CCOC(=O)CCC(=O)OC1C(OC(CC1N(C)C)C)OC1C(C)C(OC2OC(C)C(C(C2)(C)OC)O)C(C)C(=O)OC(CC)C(C(C(C(=O)-C(CC1(C)O)C)C)O)(C)O

Continued on next page

COC1C=COC2(C)Oc3c(C2=O)c2c(O)c(C=NN=C(N(CC)CC)C)c(c(c2c(c3C)O)O)NC(=O)C(=CC=CC(C(C(C(C(C(C1C)O-<br>C(=O)C)C)O)C)O)C)C
[O-]P(=O)(OC1C(OP(=O)([O-])[O-])C(OP(=O)([O-])[O-])C(C(C1OP(=O)([O-])[O-])OP(=O)([O-])[O-])OP(=O)([O-])[O-])[-<br>O-]
COC1CCC(OC1C)OC1CC(OC1C1(C)CCC(O1)C1(C)CCC2(O1)CC(O)C(C(O2)C(C1OC(O)(CC(=O)O)C(C(C1OC)O)C)C)-<br>C)C1OC(C)(O)C(CC1C)C
OCC1OC2OC3C(CO)OC(C(C3O)O)OC3C(CO)OC(C(C3O)O)OC3C(OC(OC4C(OC(OC5C(OC(OC1C(C2O)O)C(O)C5O)C-<br>O)C(O)C4O)CO)C(C3O)O)CO
CCc1c2cc(ccc2nc2c1Cn1c2cc2c(c1=O)COC(=O)C2(O)CC)OC(=O)CCC(=O)Oc1c(C)c(C)c2c(c1C)CCC(O2)(C)CCCC(CCC-<br>C(CCCC(C)C)C)C
CCC1CC1(NC(=O)C1CC(CN1C(=O)C(C(C)(C)C)NC(=O)OC1CC2C(C1)C2)Oc1cc(nc2c1ccc(c2Cl)OCCN1CCOCC1)c1csc(-<br>n1)NC(C)C)C(=O)O
NC(=N)NCCCCC(=O)Nc1cc(cc(c1OC1CNCC1)NC(=O)c1ncnc(c1)C(=O)Nc1cc(cc(c1OC1CNCC1)NC(=O)CCCCNC(=N)N-<br>)C(F)(F)F)C(F)(F)F
OC1C(OC2OC(C(=O)O)C(C(C2O)O)O)C(OC(C1O)C(=O)O)OC1CCC2(C(C1(C)C)CCC1(C2C(=O)C=C2C1(C)CCC1(C2-<br>CC(C)(CC1)C(=O)O)C)C)C
COc1cc2c(cc1OC)CC[N+](C2Cc1ccc(c(c1)OC)OC)(C)CCC(=O)OCCCCCOC(=O)CC[N+]1(C)CCc2c(C1Cc1ccc(c(c1)OC)O-<br>C)cc(c(c2)OC)OC
O=CCC1CC(C)C(C=CC=CCC(OC(=O)CC(C(C1OC1OC(C)C(C(C1O)N(C)C)OC1OC(C)C(C(C1)(C)OC(=O)C)OC(=O)C-<br>C)OC)OC(=O)CC)C)OC(=O)C
COC(=O)NC(C(=O)N1CCCC1c1nc2c([nH]1)cc(cc2)c1csc2c1scc2c1ccc(cc1)c1cnc([nH]1)C1CCCN1C(=O)C(c1ccccc1)NC(=O-<br>)OC)C(C)C
O=C1OC2CCCC2OCC=CC(F)(F)c2c(OC3CN(C(=O)C(N1)C(C)(C)C)C(C3)C(=O)NC1(CC1C(F)F)C(=O)NS(=O)(=O)C1-<br>(C)CC1)nc1c(n2)cccc1
OC1CCC(O)C(O)CC(=O)CC(O)C(C(=O)O)C(O)CC(C=CC=CC=CC=CCCC=CC=CC(C(C(C(OC(=O)CC(CC(C1)O)O)-<br>C)C)O)C)OC1OC(C)C(C(C1O)N)O
COC1CC(OC(C1OC1CC(OC)C(C(O1)C)O)C)OC1C(C)C=CC=C2COC3C2(O)C(C=C(C3O)C)C(=O)OC2CC(CC=C1C)O-<br>C1(C2)C=CC(C(O1)C1CCCCC1)C
O=CN1c2cc(OC)c(cc2C23C1C(O)(C(=O)OC)C(OC(=O)C)C1(C3N(CC2)CC=C1)CC)C1(CC2CN(CCc3c1[nH]c1c3cccc1)CC-<br>(C2)(O)CC)C(=O)OC
OCCC1CC(C)C(=O)C=CC(=CC(C(OC(=O)CC(C(C1OC1OC(C)C(C(C1O)N(C)C)OC1OC(C)C(C(C1)(C)O)O)C)O)CC)C-<br>OC1OC(C)C(C(C1OC)OC)O)C
CCOc1ccc(cc1)Nc1ccc(cc1)C(=C1C=CC(=[N+](Cc2cccc(c2)S(=O)(=O)[O-])CC)C=C1C)c1ccc(cc1C)N(Cc1cccc(c1)S(=O)(-<br>=O)[O-])CC
CCC1C2CN(C1C(=O)NC1(CC1C(F)F)C(=O)NS(=O)(=O)C1(C)CC1)C(=O)C(NC(=O)OC1CC1CCCCC(c1c(O2)nc2cc(OC-<br>)ccc2n1)(F)F)C(C)(C)C
CCC1C2=NC(=Cc3[n-]c(c(c3C(=O)C)C)C=C3N=C(C(=c4[n-]c(=C2)c(C)c4C(=O)NCCS(=O)(=O)[O-])CC(=O)OC)C(CC-<br>C(=O)[O-])C3C)C1C
COC1CCC(OC1C)OC1CC(OC1C1(C)CCC(O1)C1(C)CCC2(O1)CC(O)C(C(O2)C(C1OC(O)(CC(=O)[O-])C(C(C1OC)O)C)-<br>C)C)C1OC(C)(O)C(CC1C)C
O=CCC1CC(C)C(=O)C=CC(=CC(C(OC(=O)CC(C(C1OC1OC(C)C(C(C1O)N(C)C)OC1OC(C)C(C(C1)(C)O)O)C)O)CC)-<br>COC1OC(C)C(C(C1OC)OC)O)C
COC(=O)NC(C(=O)N1CCCC1C(=O)Nc1ccc(cc1)C1CCC(N1c1ccc(cc1)C(C)(C)C)c1ccc(cc1)NC(=O)C1CCCN1C(=O)C(C(-<br>C)C)NC(=O)OC)C(C)C
OC1CCC(O)C(O)CC(O)CC2(O)CC(O)C(C(O2)CC(C=CC=CC=CC=CC=CC=CC(C(C(C(OC(=O)CC(C1)O)C)C)O)-<br>C)OC1OC(C)C(C(C1O)N)O)C(=O)O
COC1C=COC2(C)Oc3c(C2=O)c2C4=NC5(NC4=C(C(=O)c2c(c3C)O)NC(=O)C(=CC=CC(C(C(C(C(C(C1C)OC(=O)C)C)-<br>O)C)O)C)C)CCN(CC5)CC(C)C
COC1CC(CCC1O)CC(C1OC(=O)C2CCCCN2C(=O)C(=O)C2(O)OC(CCC2C)CC(OC)C(=CC=CC=CC(CC(C(=O)C(C(C(-<br>=CC(C(=O)C1)C)C)O)OC)C)C)C)C
COC1C=COC2(C)Oc3c(C2=O)c2c(O)c(C=NN4CCN(CC4)C4CCCC4)c(c(c2c(c3C)O)O)NC(=O)C(=CC=CC(C(C(C(C(C(C-<br>1C)OC(=O)C)C)O)C)O)C)C
OCC1OC(OC2C(O)CC(OC2C)OC2C(O)CC(OC2C)OC2C(O)CC(OC2C)OC2CCC3(C(C2)CCC2C3CC(O)C3(C2(O)CCC3C2-<br>=CC(=O)OC2)C)C)C(C(C1O)O)O
O=COC1CC(OC2CCC3(C(C2)CCC2C3CCC3(C2(O)CC(C3C2=CC(=O)OC2)OC=O)C)C)OC(C1OC1CC(OC=O)C(C(O1)-<br>C)OC1CC(OC=O)C(C(O1)C)OC=O)C
COC1C(=O)C2(C)C(OC)CC3C(C2C(C2(C(C1=C(C)C(OC(=O)C(C(c1ccccc1)NC(=O)OC(C)(C)C)O)C2)(C)C)O)OC(=O)c-<br>1ccccc1)(CO3)OC(=O)C
CCC(C1NC(=O)C(NC(=O)CCCCCC(NC(=O)C(NC(=O)C(NC1=O)CCC(=O)N)CC(=O)N)C(=O)NCC(=O)NC(C(=O)NC-<br>C(=O)N)CC(C)C)Cc1ccc(cc1)O)C
COC1CC(CCC1O)CC(C1CCC(C)C=C(C)C(O)C(OC)C(=O)C(C)CC(C)C=CC=CC=C(C(CC2OC(C(=O)C(=O)N3C(C(=O-<br>)O1)CCCC3)(O)C(C)CC2)OC)C)C
COc1ccc2c(c1C)nc(cc2OC1CC2N(C1)C(=O)C(CCCCCC=CC1C(NC2=O)(C1)C(=O)NS(=O)(=O)C1CC1)CC(=O)N1CCCC-<br>(C1)(F)F)c1csc(n1)C(C)C
COC1CC(OC(C1OC)C)OC1CC(OC1C1(C)CCC(O1)C1(C)CCC2(O1)CC(O)C(C(O2)C(C1OC(O)(CC(=O)O)C(C(C1OC)OC-<br>)C)C)C)C1OC(C)(O)C(CC1C)C
CC(OC(=O)NC(C(C(=O)OC1CC2(O)C(OC(=O)c3ccccc3)C3C4(COC4CC(C3(C(=O)C(C(=C1C)C2(C)C)OC(=O)C1CCCC-<br>1)C)O)OC(=O)C)O)c1cccs1)C
Clc1ccc(cc1)C1=C(CN2CCN(CC2)c2ccc(cc2)C(=O)NS(=O)(=O)c2ccc(c(c2)S(=O)(=O)C(F)(F)F)NC(CSc2ccccc2)CCN2CC-<br>OCC2)CC(CC1)(C)C
COC(=O)NC(C(=O)N1CCCC1C1=NCC(=N1)c1ccc2c(c1)OC(n1c2cc2c1ccc(c2)c1cnc([nH]1)C1CCCN1C(=O)C(C(C)C)NC(-<br>=O)OC)c1ccccc1)C(C)C
CC(=O)OC1C(=O)C2(C)C(O)CC3C(C2C(C2(C(C1=C(C)C(OC(=O)C(C(c1ccccc1)NC(=O)c1ccccc1)O)C2)(C)C)O)OC(=O-<br>)c1ccccc1)(CO3)OC(=O)C
CCCCCCCCCCCCCCCC(=O)OC1CC(=C(C(C1)(C)C)C=CC(=CC=CC(=CC=CC=C(C=CC=C(C=CC1C(=CC(CC1(C)-<br>C)OC(=O)CCCCCCCCCCCCCCC)C)C)C)C)C)C
OC1C(OC2OC(C(=O)[O-])C(C(C2O)O)O)C(OC(C1O)C(=O)[O-])OC1CCC2(C(C1(C)C)CCC1(C2C(=O)C=C2C1(C)CCC1-<br>(C2CC(C)(CC1)C(=O)O)C)C)C
OCCOC1CCC(CC1OC)CC(C1OC(=O)C2CCCCN2C(=O)C(=O)C2(O)OC(CCC2C)CC(OC)C(=CC=CC=CC(CC(C(=O)C(-<br>C(C(=CC(C(=O)C1)C)C)O)OC)C)C)C)C

Continued on next page

Table S8 – *Continued from previous page*

CCC(C(N(C(=O)C(C(C)C)NC(=O)C(N(C(=O)CCCCCN1C(=O)C=CC1=O)C)C(C)C)C)C(CC(=O)N1CCCC1C(C(C(=O)-NC(C(=O)O)Cc1ccccc1)C)OC)OC)C
COc1ccc2c(c1C)nc(cc2OC1CC2N(C1)C(=O)C(CCCCCC=CC1C(NC2=O)(C1)C(=O)[N-]S(=O)(=O)C1CC1)CC(=O)N1CC-CC(C1)(F)F)c1csc(n1)C(C)C
OC(=O)CCC(C(=O)O)NC(=O)NC(C(=O)O)CCC(=O)NC(C(=O)O)CCCCN(Cc1nccn1CC(=O)N(CC(=O)O)CC(=O)O)Cc-1nccn1CC(=O)N(CC(=O)O)CC(=O)O
NCCCC(C(=O)NCC(=O)N)NC(=O)C1CCCN1C(=O)C1CSSCCC(=O)NC(Cc2ccc(cc2)OCC)C(=O)NC(C(=O)NC(C(=O)N-C(C(=O)N1)CC(=O)N)C(O)C)C(CC)C
CCC(C(C(=O)NCc1ccccn1)NC(=O)C(C(C)C)CC(C(NC(=O)C(N(C(=O)C(NC(=O)C1CCCN1C(=O)OC(C)(C)C)Cc1ccccc1-)C)Cc1nc[nH]c1)CC(C)C)O)C
ClCCN1C(=O)OC2(C1=O)C1N(C)c3c(C41CCN1C4C(C2OC(=O)C)(CC)C=CC1)cc(c(c3)OC)C1(CC2CN(CCc3c1[nH]c1c3cc-cc1)CC(C2)(O)CC)C(=O)OC
Clc1ccc(cc1)C1=C(CCC(C1)(C)C)CN1CCN(CC1)c1ccc(c(c1)Oc1cnc2c(c1)cc[nH]2)C(=O)NS(=O)(=O)c1ccc(c(c1)[N+](=O)[-O-])NCC1CCOCC1
NCCCCC1NC(=O)C(NC(=O)C(Cc2ccccc2)NC(=O)C(CSSCC(NC(=O)C(NC1=O)C(O)C)C(=O)NC(C(O)C)CO)NC(=O)C(-Cc1ccccc1)N)Cc1c[nH]c2c1cccc2
CCC1=C(C)C2=Cc3[nH]c(c(c3C=C)C)C=C3N=C(C(=c4[nH]c(=CC1=N2)c(C)c4C(=O)[O-])CC(=O)NC(C(=O)[O-])CC(=-O)[O-])C(CCC(=O)[O-])C3C
ClCC1CN(c2c1c1c(C)c[nH]c1c(c2)O)C(=O)c1[nH]c2c(c1)cc(cc2)NC(=O)Nc1ccc2c(c1)cc([nH]2)C(=O)N1CC(c2c1cc(O)c1c2c(-C)c[nH]1)CCl
CCC(C1NC(=O)C(NC(=O)CCSSCC(NC(=O)C(NC(=O)C(NC1=O)CCC(=O)N)CC(=O)N)C(=O)N1CCCC1C(=O)NC(C(-=O)NCC(=O)N)CC(C)C)Cc1ccc(cc1)O)C
COCC1CC(N(C1)C(=O)C(c1ccccc1)NC(=O)OC)c1ncc([nH]1)c1ccc2c(c1)COc1c2cc2ccc3c(c2c1)[nH]c(n3)C1CCC(N1C(=O)C-(C(C)C)NC(=O)OC)C
OCC1OC(OC2C(C)OC(CC2OC(=O)C)OC2C(O)CC(OC2C)OC2C(O)CC(OC2C)OC2CCC3(C(C2)CCC2C3CC(O)C3(C2(O)-CCC3C2=CC(=O)OC2)C)C)C(C(C1O)O)O
COC1CC(CCC1n1cnnn1)CC(C1OC(=O)C2CCCCN2C(=O)C(=O)C2(O)OC(CCC2C)CC(OC)C(=CC=CC=CC(CC(C(=O)-C(C(C(=CC(C(=O)C1)C)C)O)OC)C)C)C)C
CCC(C1NC(=O)C(NC(=O)CCCSCC(NC(=O)C(NC(=O)C(NC1=O)CCC(=O)N)CC(=O)N)C(=O)N1CCCC1C(=O)NC(C(-=O)NCC(=O)N)CC(C)C)Cc1ccc(cc1)OC)C
CCC(C1NC(=O)C(Cc2ccc(cc2)O)NC(=O)C(N)CSSCC(NC(=O)C(NC(=O)C(NC1=O)CC(=O)N)CC(=O)N)C(=O)N1CCC-C1C(=O)NC(C(=O)NCC(=O)N)CC(C)C)C
OCC1OC(OCC2OC(OC(=O)C34CCC(C(C4C4=CCC5C(C4(CC3)C)(C)CCC3C5(C)CC(C(C3(C)CO)O)O)C)C)C(C(C2O)O)-O)C(C(C1OC1OC(C)C(C(C1O)O)O)O)O
CCC(C1NC(=O)C(Cc2ccc(cc2)O)NC(=O)C(N)CSSCC(NC(=O)C(NC(=O)C(NC1=O)CCC(=O)N)CC(=O)N)C(=O)N1CC-CC1C(=O)NC(C(=O)NCC(=O)N)CC(C)C)C
COc1cc2CC[N+](C(c2cc1OC)Cc1cc(OC)c(c(c1)OC)OC)(C)CCCOC(=O)C(=CC(=O)OCCC[N+]1(C)CCc2c(C1c1cc(OC)c(c(-c1)OC)OC)cc(c(c2)OC)OC)Cl
COc1cc2c(cc1OC)CC[N+](C2Cc1cc(OC)c(c(c1)OC)OC)(C)CCCOC(=O)CCC=CCCC(=O)OCCC[N+]1(C)CCc2c(C1Cc1cc(-OC)c(c(c1)OC)OC)cc(c(c2)OC)OC
COC1C=COC2(C)Oc3c(C2=O)c2c4nc5c(O)cc(cc5oc4c(c(=O)c2c(c3C)O)NC(=O)C(=CC=CC(C(C(C(C(C(C1C)OC(=O)C)-C)O)C)O)C)C)N1CCN(CC1)CC(C)C
COC1CC(CCC1OP(=O)(C)C)CC(C1OC(=O)C2CCCCN2C(=O)C(=O)C2(O)OC(CCC2C)CC(OC)C(=CC=CC=CC(CC(C(-=O)C(C(C(=CC(C(=O)C1)C)C)O)OC)C)C)C)C
CCOCCOC1CCC(CC1OC)CC(C1CC(=O)C(C)C=C(C)C(O)C(OC)C(=O)C(C)CC(C)C=CC=CC=C(C(CC2OC(C(=O)C(=-O)N3C(C(=O)O1)CCCC3)(O)C(C)CC2)OC)C)C
NC(=N)NCCCC(C(=O)NC(C(=O)NC(C(=O)NC(C(=O)N)CCCNC(=N)N)C)CCCNC(=N)N)NC(=O)C(NC(=O)C(NC(=O-)C(NC(=O)C)CSSCC(C(=O)O)N)C)CCCNC(=N)N
CC(CC(C(=O)NC(C(=O)N1CCCC1C(=O)NCC(=O)N1CCCC1C(=O)NC(C(=O)NC(C(=O)NC(C(=O)O)C(C)C)C(O)C)C(-C)C)CCC(=O)O)NC(=O)C(Cc1ccc(cc1)O)N)C
NCCCC(C(=O)NCC(=O)N)NC(=O)C1CCCN1C(=O)C1CSSCC(N)C(=O)NC(Cc2ccc(cc2)O)C(=O)NC(C(=O)NC(C(=O)N-C(C(=O)N1)CC(=O)N)CCC(=O)N)Cc1ccccc1
NC(=O)CC1NC(=O)C(CCC(=O)N)NC(=O)C(Cc2ccccc2)NC(=O)C(NC(=O)CCSSCC(NC1=O)C(=O)N1CCCC1C(=O)NC-(C(=O)NCC(=O)N)CCCNC(=N)N)Cc1ccc(cc1)O
NCCCCC(C(=O)NCC(=O)N)NC(=O)C1CCCN1C(=O)C1CSSCC(N)C(=O)NC(Cc2ccc(cc2)O)C(=O)NC(C(=O)NC(C(=O)-NC(C(=O)N1)CC(=O)N)CCC(=O)N)Cc1ccccc1
NCCCCC1NC(=O)C(NC(=O)C(NC(=O)C2N(C(=O)C(NC(=O)C(NC1=O)Cc1ccc(cc1)OCc1ccccc1)Cc1ccccc1)CC(C2)OC(-=O)NCCN)c1ccccc1)Cc1c[nH]c2c1cccc2
COC(=O)NC(C(=O)N1CC2(CC1c1[nH]cc(n1)c1ccc3c(c1)C(F)(F)c1c3ccc(c1)c1ccc3c(c1)nc([nH]3)C1C3CCC(N1C(=O)C(C(-C)C)NC(=O)OC)C3)CC2)C(C)C
COc1c(OC)cc2c(c1OC)C(Cc1cc(OC)c(c(c1)OC)OC)[N+](CC2)(C)CCCOC(=O)CCC(=O)OCCC[N+]1(C)CCc2c(C1Cc1cc(O-C)c(c(c1)OC)OC)c(OC)c(c(c2)OC)OC
CCCCC(C(=O)NC1CC(=O)NCCCCC(NC(=O)C(NC(=O)C(NC(=O)C(NC(=O)C(NC1=O)Cc1[nH]cnc1)Cc1ccccc1)CCCNC-(=N)N)Cc1c[nH]c2c1cccc2)C(=O)O)NC(=O)C
CCC1NC(=O)C(NC(=O)c2ncccc2O)C(C)OC(=O)C(NC(=O)C2N(C(=O)C(N(C(=O)C3N(C1=O)CCC3)C)Cc1ccc(cc1)N(C)-C)CC(CSC1CN3CCC1CC3)C(=O)C2)c1ccccc1
OCC1OC2OC3C(CO)OC(C(C3O)O)OC3C(CO)OC(C(C3O)O)OC3C(CO)OC(C(C3O)O)OC3C(OC(OC4C(OC(OC5C(OC(O-C1C(C2O)O)C(O)C5O)CO)C(O)C4O)CO)C(O)C3O)CO
NC(=O)CC1NC(=O)C(CCC(=O)N)NC(=O)C(Cc2ccccc2)NC(=O)C(Cc2ccc(cc2)O)NC(=O)C(CSSCC(NC1=O)C(=O)N1C-CCC1C(=O)NC(C(=O)NCC(=O)N)CCCNC(=N)N)N
COC1CC(CCC1OC(=O)C(CO)(CO)C)CC(C1OC(=O)C2CCCCN2C(=O)C(=O)C2(O)OC(CCC2C)CC(OC)C(=CC=CC=C-C(CC(C(=O)C(C(C(=CC(C(=O)C1)C)C)O)OC)C)C)C)C
COc1ccc(cc1)CC(NC(=O)C1CCCN1C(=O)C(NC(=O)C(Cc1cccs1)NC(=O)CNC(=O)C1CC(CN1C(=O)C1CCCN1C(=O)C(-CCCNC(=N)N)N)O)CO)CNC(C(=O)O)CCCNC(=N)N
CCCCCCCCOc1ccc(cc1)C(=O)NC1CC(O)C(O)NC(=O)C2C(O)C(CN2C(=O)C(NC(=O)C(NC(=O)C2N(C(=O)C(NC1=O)-C(O)C)CC(C2)O)C(C(c1ccc(cc1)O)O)O)C(O)C)C
O=CCC1CC(C)C(=O)C=CC(=CC(C(OC(=O)CC(C(C1OC1OC(C)C(C(C1O)N(C)C)OC1OC(C)C(C(C1)(C)O)OC(=O)CC(-C)C)C)OC(=O)C)CC)COC1OC(C)C(C(C1OC)OC)O)C
NCCCCC(C(=O)Nc1ccc(c(c1)C(=O)NC(C(=O)Nc1ccc(c(c1)C(=O)N)OC)CCCCN)OC)NC(=O)c1cc(ccc1OC)NC(=O)C(NC-(=O)c1cc(ccc1OC)NC(=O)C(CCCCN)N)CCCCN

Continued on next page

NCCNC1NC(=O)C2C(O)CCN2C(=O)C(NC(=O)C(NC(=O)C2N(C(=O)C(NC(=O)C(CC1O)NC(=O)CCCCCCCCC(CC(C-C)C)C)C(O)C)CC(C2)O)C(C(c1ccc(cc1)O)O)O)C(CCN)O
COC1C(OCC2=CC=CCC(O)C(=CC(CC)C(C(=CC(=CCC(OC2=O)C(O)C)C)C)OC2OC(C)(C)C(C(C2O)O)OC(=O)C(C)-C)C)OC(C(C1O)OC(=O)c1c(O)c(Cl)c(c(c1CC)Cl)O)C
NC(=N)NCCCC(C(=O)NC(C(=O)NC(C(=O)NC(C(=O)NC(C(=O)N1CCCC1C(=O)NC(C(=O)O)Cc1ccccc1)Cc1[nH]cnc1)-C(C)C)Cc1ccc(cc1)O)C(C)C)NC(=O)C(CC(=O)N)N
CCC(C(C(=O)NC(C(=O)N1CCCC1C(=O)NC(C(=O)O)Cc1ccccc1)Cc1[nH]cnc1)NC(=O)C(NC(=O)C(C(C)C)NC(=O)C(N-C(=O)C(CC(=O)O)N)CCCNC(=N)N)Cc1ccc(cc1)O)C
CCC(C(C(=O)O)NC(=O)C(NC(=O)C(CC(=O)N)NC(=O)CNC(=O)C(NC(=O)C1CCCN1C(=O)C(C(C)C)NC(=O)C(NC(-=O)C(CCCNC(=N)N)N)Cc1ccccc1)CC(=O)O)CCCNC(=N)N)C
CNC(=N)NCCCC=CCCCC(CC(C1OC(=O)C(C)C(O)C=CC(C)C(O)CC(O)C(C)C(O)CCC(C)C(O)CC2(OC(CC(CC(CC(C(-C(C=CC=CC1C)O)C)O)O)OC(=O)CC(=O)O)CC(O)C2O)O)C)C
CCCCCCCCCCOC1C(O)C(COC2OC(COC)C(C(C2NC(=O)CCCCCCCCCC=CCCCCCC)OCCC(CCCCCCC)OC)OP(=O)(-[O-])[O-])OC(C1NC(=O)CC(=O)CCCCCCCCCCC)OP(=O)([O-])[O-]
NCCCCC1NC(=O)C(NC(=O)C(Cc2ccc(cc2)O)NC(=O)C(CSSCC(NC(=O)C(NC1=O)C(C)C)C(=O)NC(C(=O)N)Cc1c[nH]c-2c1cccc2)NC(=O)C(Cc1ccccc1)N)Cc1c[nH]c2c1cccc2
CSCCC(C(=O)NCC(=O)NC(C(=O)NC(C(=O)NC(C(=O)NC(C(=O)N)Cc1ccccc1)CC(=O)O)CCSC)Cc1c[nH]c2c1cccc2)NC-(=O)C(NC(=O)C(CC(=O)O)N)Cc1ccc(cc1)OS(=O)(=O)O
CCC(C(C(=O)N1CCCC1C(=O)N1CCCC1C(=O)O)NC(=O)C(NC(=O)C1CCCN1C(=O)C(NC(=O)C1CCCN1C(=O)C(Cc1c-[nH]c2c1cccc2)NC(=O)C1CCC(=O)N1)CCCNC(=N)N)CCC(=O)N)C
OCC(C(=O)NC(C(=O)NCC(=O)NC(C(=O)NC(C(=O)NC(C(=O)NC(C(=O)NC(C(=O)NC(C(CC)C)C(=O)O)CCC(=O)O)-Cc1c[nH]c2c1cccc2)CC(C)C)CC(C)C)C(C)C)Cc1ccc(cc1)O)N
COC(C1Cc2cc3cc(OC4OC(C)C(C(C4)OC4CC(O)C(C(O4)C)O)O)c(c(c3c(c2C(=O)C1OC1OC(C)C(C(C1)OC1OC(C)C(C(C1-)OC1OC(C)C(C(C1)(C)O)O)O)O)O)O)C)C(=O)C(C(O)C)O
CC=CC=CC1OC(O)(C(C(=O)NCC=CC=C(C(C(C2OC(C(C2O)O)C=CC=CC=C(C(=O)c2c(O)ccn(c2=O)C)C)C)OC)C)C-C)C(C(C1(C)C)OC1OC(C)C(C(C1O)OC)OC1OC(C)C(C(C1OC)O)OC)O
CCCCC(C(=O)NC(C(=O)NC(C(=O)NCC(=O)NC(C(=O)N)Cc1ccc(cc1)O)CCCC)CCCC)NC(=O)C(NC(=O)C(NC(=O)C(-NC(=O)C(NC(=O)C(CCCNC(=N)N)N)CCCC)CCCC)CCCC)CCCNC(=N)N
OCC1OC(OC2C(OC(C(C2O)OC2OC(CO)C(C(C2O)O)O)C(=O)O)OC2CCC3(C(C2(C)CO)CCC2(C3CC=C3C2(C)CC(C2(C-3CC(C)(C)C(C2OC(=O)C)OC(=O)C(=CC)C)CO)O)C)C)C(C(C1O)O)O
COC(=O)NC(C(=O)N1CCCC1c1nc2c([nH]1)cc(c(c2)C1CCC(N1c1cc(F)c(c(c1)F)N1CCC(CC1)c1ccc(cc1)F)c1cc2nc([nH]c2cc-1F)C1CCCN1C(=O)C(C(OC)C)NC(=O)OC)F)C(OC)C
NCCCCC(C(=O)NC(C(=O)NC(C(=O)NC(C(=O)NC(C(=O)NCC(=O)NC(C(=O)NC(C(=O)N)CCSC)CC(C)C)C(CC)C)Cc-1ccccc1)C)CC(=O)O)NC(=O)C(NC(=O)C1CCCN1C(=O)C1CCC(=O)N1)CO
CCC(C1NC(=O)C(NC(=O)C(N(C(=O)C2CCCN2C(=O)C(=O)C)C)CC(C)C)C(C)OC(=O)C(Cc2ccc(cc2)OC)N(C)C(=O)C-2CCCN2C(=O)C(NC(=O)C(C(=O)C(OC(=O)CC1O)C(C)C)C)CC(C)C)C
CCCCCOc1ccc(cc1)c1ccc(cc1)c1ccc(cc1)C(=O)NC1CC(O)C(O)NC(=O)C2C(O)C(CN2C(=O)C(NC(=O)C(NC(=O)C2N(C(-=O)C(NC1=O)C(O)C)CC(C2)O)C(C(c1ccc(cc1)O)O)O)C(O)C)C
NCCCCC(C(=O)NCC(=O)N)NC(=O)C1CCCN1C(=O)C1CSSCC(NC(=O)CNC(=O)CNC(=O)CN)C(=O)NC(Cc2ccc(cc2)O-)C(=O)NC(C(=O)NC(C(=O)NC(C(=O)N1)CC(=O)N)CCC(=O)N)Cc1ccccc1
CCC(C1OC(=O)C(N(C)C(=O)C(CC(C)C)NC(=O)C(C(C)C)N(C)C(=O)C(NC(=O)C2N(C(=O)C(N(C(=O)C(NC(=O)C(N-(C1=O)C)C(C)C)Cc1ccccc1)C)Cc1ccccc1)CCC2)C(CC)C)C(O)(C)C)C
CCNC(=O)C1CCCN1C(=O)C(NC(=O)C(NC(=O)CNC(=O)C(Cc1ccc(cc1)O)NC(=O)C(NC(=O)C(Cc1c[nH]c2c1cccc2)NC(-=O)C(Cc1cnc[nH]1)NC(=O)C1CCC(=O)N1)CO)CC(C)C)CCCNC(=N)N
COC1C(OC(=O)c2ccc([nH]2)C)C(O)C(OC1(C)C)Oc1ccc2c(c1C)oc(=O)c(c2O)NC(=O)c1c[nH]c(c1C)C(=O)Nc1c(=O)oc2c(c-1O)ccc(c2C)OC1OC(C)(C)C(C(C1O)OC(=O)c1ccc([nH]1)C)OC
OCC(C(=O)NC(C(=O)NCC(=O)NC(C(=O)NC(C(=O)N1CCCC1C(=O)NCC(=O)N)CCCNC(=N)N)CC(C)C)Cc1ccc(cc1)O-)NC(=O)C(Cc1c[nH]c2c1cccc2)NC(=O)C(NC(=O)C1CCC(=O)N1)Cc1[nH]cnc1
COC(C1Cc2cc3cc(OC4CC(OC5CC(O)C(C(O5)C)OC)C(C(O4)C)OC(=O)C)c(c(c3c(c2C(=O)C1OC1CC(OC2CC(OC3OC(C)-C(C(C3)(C)O)OC(=O)C)C(C(O2)C)O)C(C(O1)C)O)O)O)C)C(=O)C(C(O)C)O
CC=C1NC(=O)C(NC(=O)c2csc(n2)c2cc(O)c(nc2c2csc(n2)C2NC(=O)c3nc(C(NC(=O)c4nc1sc4)CC(O)C(=O)OCc1cccc4c1c(-C)c([nH]4)C(=O)SC2)sc3)c1scc(n1)C(=O)NC(=C)C(=O)N)C(O)C
CCNC(=O)C1CCCN1C(=O)C(NC(=O)C(NC(=O)C(NC(=O)C(Cc1ccc(cc1)O)NC(=O)C(NC(=O)C(Cc1c[nH]c2c1cccc2)NC(-=O)C(Cc1cnc[nH]1)NC(=O)C1CCC(=O)N1)CO)CC(C)C)CC(C)C)CCCNC(=N)N
NCCCCC(C(=O)NC(C(=O)NC(C(=O)NC(C(=O)NC(C(=O)NC(C(=O)NC(C(=O)N1CCCC1C(=O)NC(C(=O)NC(C(=O)-O)CC(C)C)CC(C)C)CCCNC(=N)N)C(C)C)CCCNC(=N)N)CC(C)C)Cc1ccc(cc1)O)C(C)C)N
O=C1CNC(=O)C(NC(=O)C2CSSCC3NC(=O)C(CSSCC(NC(=O)C(CSSCC(N1)C(=O)O)NC(=O)C(CC(C)C)NC(=O)C(N-C3=O)COP(=O)(O)O)C(=O)NC(CC(=O)N)C(=O)N1C(C(=O)NC(C(=O)N2)C)CCC1)N)C(O)C
CCNC(=O)C1CCCN1C(=O)C(NC(=O)C(NC(=O)C(NC(=O)C(Cc1ccc(cc1)O)NC(=O)C(NC(=O)C(Cc1c[nH]c2c1cccc2)NC(-=O)C(Cc1cnc[nH]1)NC(=O)C1CCC(=O)N1)CO)COC(C)(C)C)CC(C)C)CCCNC(=N)N
NCCCCC(C(=O)NC(C(=O)NC(C(=O)N)CCCCN)CS)NC(=O)C(CNC(=O)CSCCC1NC(=O)C(NC(=O)C(CCCCN)NC(=O)-C(NC(=O)C(NC(=O)C(N(C1=O)C)Cc1ccccc1)Cc1ccc(cc1)O)Cc1c[nH]c2c1cccc2)C(C)C)N
CC=CCC(C(C1C(=O)NC(CC)C(=O)N(C)CC(=O)N(C)C(CC(C)C)C(=O)NC(C(C)C)C(=O)N(C)C(CC(C)C)C(=O)NC(C(-=O)NC(C(=O)N(C(C(=O)N(C(C(=O)N(C(C(=O)N1C)C(C)C)C)CC(C)C)C)CC(C)C)C)C)C)O)C
OCC(C(=O)NC(C(=O)NC(C(=O)NC(C(=O)NC(C(=O)N1CCCC1C(=O)NNC(=O)N)CCCNC(=N)N)CC(C)C)COC(C)(C)-C)Cc1ccc(cc1)O)NC(=O)C(Cc1c[nH]c2c1cccc2)NC(=O)C(NC(=O)C1CCC(=O)N1)Cc1nc[nH]c1
CCCCCOc1ccc(cc1)c1onc(c1)c1ccc(cc1)C(=O)NC1CC(O)C(O)NC(=O)C2C(O)C(CN2C(=O)C(NC(=O)C(NC(=O)C2N(C(=-O)C(NC1=O)C(O)C)CC(C2)O)C(C(c1ccc(c(c1)OS(=O)(=O)O)O)O)O)C(CC(=O)N)O)C
NCCCCC1NC(=O)C(NC(=O)C(Cc2ccccc2)NC(=O)C(CSSCC(NC(=O)C(NC1=O)C(O)C)C(=O)NC(C(O)C)CO)NC(=O)C(-Cc1ccccc1)NC(=O)CN(CC(=O)O)CCN(CC(=O)O)CCN(CC(=O)O)CC(=O)O)Cc1c[nH]c2c1cccc2
CCC(CCCCCC=CCC(=O)NC(C(=O)N(C(C(=O)NC(C(=O)NCC(=O)NC(C(=O)NCC(=O)NC(C(=O)NC(C(=O)N1CCCC-1C(=O)NC1C(C)NC(=O)C2N(C1=O)CCCC2)C(C)C)C(N)C)CC(=O)O)CC(=O)O)CC(=O)O)C)CC(=O)O)C
CCNC(=O)C1CCCN1C(=O)C(NC(=O)C(NC(=O)C(Cc1c[nH]c2c1cccc2)NC(=O)C(Cc1ccc(cc1)O)NC(=O)C(NC(=O)C(Cc1-c[nH]c2c1cccc2)NC(=O)C(Cc1cnc[nH]1)NC(=O)C1CCC(=O)N1)CO)CC(C)C)CCCNC(=N)N
COc1c(cc2c(c1Cc1c(OC)c(cc3c1cccc3)C(=O)OCC(=O)C1C3OC(OC3C3C1(C)CC(O)C1(C3CCC3=CC(=O)C=CC13C)F)(C)-C)cccc2)C(=O)OCC(=O)C1C2OC(OC2C2C1(C)CC(O)C1(C2CCC2=CC(=O)C=CC12C)F)(C)C
C=CC=CCC(C(C1C(=O)NC(CC)C(=O)N(C)CC(=O)N(C)C(CC(C)C)C(=O)NC(C(C)C)C(=O)N(C)C(CC(C)C)C(=O)NC-(C(=O)NC(C(=O)N(C(C(=O)N(C(C(=O)N(C(C(=O)N1C)C(C)C)C)CC(C)C)C)CC(C)C)C)C)C)O)C
CCCCCOc1ccc(cc1)c1onc(c1)c1ccc(cc1)C(=O)NC1CC(O)C(O)NC(=O)C2C(O)C(CN2C(=O)C(NC(=O)C(NC(=O)C2N(C(=-O)C(NC1=O)C(O)C)CC(C2)O)C(C(c1ccc(c(c1)OS(=O)(=O)[O-])O)O)O)C(CC(=O)N)O)C

Continued on next page

CC=CCC(C(C1C(=O)NC(CC)C(=O)N(C)C(C)C(=O)N(CC)C(C(C)C)C(=O)NC(C(C)C)C(=O)N(C)C(CC(C)C)C(=O)NC-(C(=O)NC(C(=O)N(C(C(=O)N(C(C(=O)N(C(C(=O)N1C)C(C)C)C)CC(C)C)C)CC(C)C)C)C)C)O)C
CCNC(=O)C1CCCN1C(=O)C(NC(=O)C(NC(=O)C(Cc1ncn(c1)Cc1ccccc1)NC(=O)C(Cc1ccc(cc1)O)NC(=O)C(NC(=O)C(-Cc1c[nH]c2c1cccc2)NC(=O)C(Cc1c[nH]cn1)NC(=O)C1CCC(=O)N1)CO)CC(C)C)CCCNC(=N)N
NCCCCC1NC(=O)C(NC(=O)C(Cc2ccc(cc2)O)NC(=O)C(CSSCC(NC(=O)C(NC1=O)C(O)C)C(=O)NC(C(O)C)CO)NC(=O-)C(Cc1ccccc1)NC(=O)CN1CCN(CCN(CCN(CC1)CC(=O)O)CC(=O)O)CC(=O)O)Cc1c[nH]c2c1cccc2
CC=CCC(C(=O)C1C(=O)NC(C(C)C)C(=O)N(C)CC(=O)N(C)C(CC(C)C)C(=O)NC(C(C)C)C(=O)N(C)C(CC(C)C)C(=O-)NC(C(=O)NC(C(=O)N(C(C(=O)N(C(C(=O)N(C(C(=O)N1C)C(C)C)C)CC(C)C)C)CC(C)C)C)C)C)C
CCNC(=O)C1CCCN1C(=O)C(NC(=O)C(N(C(=O)C(Cc1c[nH]c2c1cccc2)NC(=O)C(Cc1ccc(cc1)O)NC(=O)C(NC(=O)C(Cc-1c[nH]c2c1cccc2)NC(=O)C(Cc1cnc[nH]1)NC(=O)C1CCC(=O)N1)CO)C)CC(C)C)CCCNC(=N)N
NCCCCC(C(=O)[N-]C(C(=O)NC(C(=O)N)CCCCN)C[S-])[N-]C(=O)C(CNC(=O)CSCCC1NC(=O)C(NC(=O)C(CCCCN)N-C(=O)C(NC(=O)C(NC(=O)C(N(C1=O)C)Cc1ccccc1)Cc1ccc(cc1)O)Cc1c[nH]c2c1cccc2)C(C)C)N
NCCCSCC1NC(=O)C(NC(=O)CSCC(NC(=O)C(NC(=O)CNC1=O)CC(=O)[O-])C(=O)NCC(=O)NCC(=O)NC(C(=O)NC-C(=O)NC(C(=O)[N-]CC(=O)[N-]CC(=O)[N-]C(C(=O)N)C[S-])CSCNC(=O)C)CSCNC(=O)C)Cc1ccc(cc1)O
OCC(C(=O)NC(C(=O)NC(C(=O)NC(C(=O)NC(C(=O)N1CCCC1C(=O)NCC(=O)N)CCCNC(=N)N)CC(C)C)Cc1c[nH]c2c-1cccc2)Cc1ccc(cc1)O)NC(=O)C(Cc1c[nH]c2c1cccc2)NC(=O)C(NC(=O)C1CCC(=O)N1)Cc1[nH]cnc1
OCC(C(=O)NC(C(=O)NC(C(=O)NC(C(=O)NC(C(=O)N1CCCC1C(=O)NCC(=O)N)CCCNC(=N)N)CC(C)C)Cc1ccc2c(c1-)cccc2)Cc1ccc(cc1)O)NC(=O)C(Cc1c[nH]c2c1cccc2)NC(=O)C(NC(=O)C1CCC(=O)N1)Cc1nc[nH]c1
O=C1NC(C(C)C)C(=O)N2CCCC2C(=O)N(C)CC(=O)N(C(C(=O)OC(C1NC(=O)c1c2nc3c(ccc(c3oc2c(c(=O)c1N)C)C)C(=-O)NC1C(C)OC(=O)C(C(C)C)N(C)C(=O)CN(C(=O)C2N(C(=O)C(NC1=O)C(C)C)CCC2)C)C)C(C)C)C
CSCCC(C(=O)NC(C(=O)NC(C(=O)N)Cc1ccccc1)CC(=O)O)NC(=O)C(Cc1c[nH]c2c1cccc2)NC(=O)CNC(=O)C(C(O)C)NC-(=O)C(NC(=O)C(NC(=O)C(NC(=O)C1CCC(=O)N1)CCC(=O)N)CC(=O)O)Cc1ccc(cc1)OS(=O)(=O)O
CCCCCCCC(=O)OC1C(OC(=O)C(=CC)C)C(=C2C1C(C)(OC(=O)C)CC(C1(C2OC(=O)C1(C)O)O)OC(=O)CCCCCCCC-CCCNC(=O)CC(C(=O)NC(C(=O)O)CCC(=O)NC(C(=O)O)CCC(=O)NC(C(=O)O)CCC(=O)NC(C(=O)O)CCC(=O)O)N)-C
NCCCCC1NC(=O)C(NC(=O)C(Cc2ccccc2)NC(=O)C(CSSCC(NC(=O)C(NC1=O)C(O)C)C(=O)NC(C(O)C)CO)NC(=O)C(-Cc1ccccc1)NC(=O)CN(CC(=O)[O-])CCN(CC(=O)[O-])CCN(CC(=O)[O-])CC(=O)[O-])Cc1c[nH]c2c1cccc2
OCC(C(=O)N(C(C(=O)NC(C(=O)NC(C(=O)NC(C(=O)N1CCCC1C(=O)NC(C(=O)N)C)CCCCNC(C)C)CC(C)C)CC(=O)-N)Cc1ccc(cc1)O)C)NC(=O)C(NC(=O)C(NC(=O)C(Cc1ccc2c(c1)cccc2)NC(=O)C)Cc1ccc(cc1)Cl)Cc1cccnc1
OCC(C(=O)NC(C(=O)NC(C(=O)NC(C(=O)NC(C(=O)N1CCCC1C(=O)NC(C(=O)N)C)CCCNC(=N)N)CC(C)C)CCCNC(-=O)N)Cc1ccc(cc1)O)NC(=O)C(Cc1cccnc1)NC(=O)C(Cc1ccc(cc1)Cl)NC(=O)C(Cc1ccc2c(c1)cccc2)NC(=O)C
CC=CCC(C(C1C(=O)NC(CC)C(=O)N(C)C(OCCN2CCOCC2)C(=O)N(C)C(CC(C)C)C(=O)NC(C(C)C)C(=O)N(C)C(CC(-C)C)C(=O)NC(C(=O)NC(C(=O)N(C(C(=O)N(C(C(=O)N(C(C(=O)N1C)C(C)C)C)CC(C)C)C)CC(C)C)C)C)C)O)C
OCC1OC(C(C1OP(=O)(OC(CNC(=O)CCC1(C)C2=C(C)C3=NC(=CC4=NC(=C(C5=NC(C(C1CC(=O)N)[N-]2)(C)C(C)(-CC(=O)N)C5CCC(=O)N)C)C(C4CCC(=O)N)(C)CC(=O)N)C(C3CCC(=O)N)(C)C)C)[O-])O)n1cnc2c1cc(C)c(c2)C
OCC1OC(OC(C(C(=O)NC(C(C(C(=O)NC(C(=O)NCCc2scc(n2)c2scc(n2)C(=O)NCCCNC(c2ccccc2)C)C(O)C)C)O)C)NC(-=O)c2nc(nc(c2C)N)C(CC(=O)N)NCC(C(=O)N)N)c2[nH]cnc2)C(C(C1O)O)OC1OC(CO)C(C(C1O)OC(=O)N)O
OC(=O)CCC1NC(=O)C2CSSCC3NC(=O)C(C)NC(=O)C4N(C(=O)C(NC(=O)C(NC(=O)C(NC(=O)C(NC1=O)Cc1ccc(cc1-)O)CSSCC(NC(=O)CNC(=O)C(NC3=O)C(O)C)C(=O)NC(C(=O)O)Cc1ccc(cc1)O)CSSCC(C(=O)N2)N)CC(=O)N)CCC4
OCC1OC(OC(C(C(=O)NC(C(C(C(=O)NC(C(=O)NCCc2scc(n2)c2scc(n2)C(=O)NCCCNC(c2ccccc2)C)C(O)C)C)O)C)NC(-=O)c2nc(nc(c2C)N)C(CC(=O)N)NCC(C(=O)N)N)c2nc[nH]c2)C(C(C1O)O)OC1OC(CO)C(C(C1O)OC(=O)N)O
OCC1OC(OC2CC(CC(C2OC2OC(C)C(C(C2O)O)O)NC(=O)c2cc(=O)[nH]c(=O)[nH]2)C(=O)NCCNC(=O)COCCOCC(=O)-Nc2cc(cc3c2c(cc(c3)S(=O)(=O)O)S(=O)(=O)O)S(=O)(=O)O)C(C(C1O)OC(C(=O)O)CC1CCCCC1)OC(=O)c1ccccc1
NCCCCC1NC(=O)C(NC(=O)C(Cc2ccc(cc2)O)NC(=O)C(CSSCC(NC(=O)C(NC1=O)C(O)C)C(=O)NC(C(=O)[O-])C(O)C)-NC(=O)C(Cc1ccccc1)NC(=O)CN1CCN(CCN(CCN(CC1)CC(=O)[O-])CC(=O)[O-])CC(=O)[O-])Cc1c[nH]c2c1cccc2
OCC1OC(Oc2c3cc4cc2Oc2ccc(cc2Cl)C(O)C2NC(=O)C(NC(=O)C4NC(=O)C(CC(=O)N)NC(=O)C(C(c4ccc(O3)c(Cl)c4)O)-NC(=O)C(CC(C)C)NC)c3ccc(c(c3)c3c(O)cc(cc3C(NC2=O)C(=O)O)O)O)C(C(C1O)O)OC1OC(C)C(C(C1)(C)N)O
CCNC(=NCCCCC(C(=O)NC(C(=O)NC(C(=O)N1CCCC1C(=O)NC(C(=O)N)C)CCCCN=C(NCC)NCC)CC(C)C)NC(=O)-C(NC(=O)C(NC(=O)C(NC(=O)C(NC(=O)C(Cc1ccc2c(c1)cccc2)NC(=O)C)Cc1ccc(cc1)Cl)Cc1cccnc1)CO)Cc1ccc(cc1)O)NC-C
O=C(Nc1cccc(c1)C(=O)Nc1cc(ccc1C)C(=O)Nc1ccc(c2c1c(cc(c2)S(=O)(=O)[O-])S(=O)(=O)[O-])S(=O)(=O)[O-])Nc1cccc(c-1)C(=O)Nc1cc(ccc1C)C(=O)Nc1ccc(c2c1c(cc(c2)S(=O)(=O)[O-])S(=O)(=O)[O-])S(=O)(=O)[O-]
O=C(COCC(=O)NCCOCCOCCOCCOCCOCCNC(=O)CON=Cc1ccc(cc1)F)NCCCCC1NC(=O)CSCC(NC(=O)C(Cc2ccccc2-)NC(=O)C2CSSCC(NC1=O)C(=O)NC(CCCNC(=N)N)C(=O)NCC(=O)NC(C(=O)N2)CC(=O)O)C(=O)NCCOCCOCCOC-CNC(=O)COCC(=O)N
ON=C(C(NCCC(CCNC(C(=NO)C)(C)C)CCNC(=O)CCCC(=O)NCCCCC1NC(=O)CSCC(NC(=O)C(Cc2ccccc2)NC(=O)C-2CSSCC(NC1=O)C(=O)NC(CCCNC(=N)N)C(=O)NCC(=O)NC(C(=O)N2)CC(=O)O)C(=O)NCCOCCOCCOCCNC(=O)-COCC(=O)N)(C)C)C
NCCCC1NC(=O)C(CCN)NC(=O)C(NC(=O)C(NC(=O)C(NC(=O)C2CCCN2C(=O)C2CCCN2C(=O)C(NC(=O)C(NC(=O)-C(NC(=O)C(NC(=O)C(NC(=O)C(NC(=O)C(NC1=O)CCN)CCN)Cc1c[nH]c2c1cccc2)CCN)CCN)C)CO)C(O)C)Cc1c[nH]c2c-1cccc2)C(CC)C
NCCCC1NC(=O)CNC(=O)C(NC(=O)C(NC(=O)C(NC(=O)C(Cc2c[nH]c3c2cccc3)NC(=O)CCCCCCCCC)CC(=O)N)CC(=-O)O)C(C)OC(=O)C(NC(=O)C(NC(=O)C(NC(=O)CNC(=O)C(NC(=O)C(NC(=O)C(NC1=O)CC(=O)O)C)CC(=O)O)CO)-C(CC(=O)O)C)CC(=O)c1ccccc1N
OCC(C(=O)NC(C(=O)NC(C(=O)NC(C(=O)NC(C(=O)N1CCCC1C(=O)NC(C(=O)N)C)CCCCNC(C)C)CC(C)C)Cc1ccc(c-c1)NC(=O)N)Cc1ccc(cc1)NC(=O)C1CC(=O)NC(=O)N1)NC(=O)C(NC(=O)C(NC(=O)C(Cc1ccc2c(c1)cccc2)NC(=O)C)Cc-1ccc(cc1)Cl)Cc1cccnc1
COC1C(OC)C(OC2C(COS(=O)(=O)[O-])OC(C(C2OS(=O)(=O)[O-])OS(=O)(=O)[O-])OC2C(OC(C(C2OC)OC)OC2C(COS-(=O)(=O)[O-])OC(C(C2OS(=O)(=O)[O-])OS(=O)(=O)[O-])OC)C(=O)[O-])OC(C1OC1OC(COS(=O)(=O)[O-])C(C(C1OC)-OC)OC)C(=O)[O-]
CCCCC(C(=O)NC(C(=O)NC(C(=O)NC(C(=O)NC(C(=O)NC(C(=O)NCC(=O)NC(C(=O)N1CCCC1C(=O)NC(C(=O)N)-C(C)C)CCCCN)Cc1c[nH]c2c1cccc2)CCCNC(=N)N)Cc1ccccc1)Cc1[nH]cnc1)CCC(=O)O)NC(=O)C(NC(=O)C(NC(=O)C(N-C(=O)C)CO)Cc1ccc(cc1)O)CO
NCCCCNCCCNC(=O)CC(CCCNC(=O)c1csc(n1)c1csc(n1)C(C(NC(=O)C(C(O)C)NC(=O)CC(C(NC(=O)C(C(c1nc[nH]c1)-OC1OC(CO)C(C(C1OC1OC(CO)C(C(C1O)OC(=O)N)O)O)O)NC(=O)c1nc(nc(c1C)N)C(CC(=O)N)NCC(C(=O)N)N)C)O)-OC1OC(C)C(C(C1O)O)N)O)N
CCCCCCCCCCNCCNC1(C)CC(OC2C(OC(C(C2O)O)CO)Oc2c3cc4cc2Oc2ccc(cc2Cl)C(O)C2NC(=O)C(NC(=O)C4NC(=O)-C(CC(=O)N)NC(=O)C(C(c4ccc(O3)c(Cl)c4)O)NC(=O)C(CC(C)C)NC)c3ccc(c(c3)c3c(C(NC2=O)C(=O)O)cc(c(c3O)CNCP-(=O)(O)O)O)O)OC(C1O)C

Continued on next page

COC1OC(COS(=O)(=O)[O-])C(C(C1NS(=O)(=O)[O-])O)OC1OC(C(=O)[O-])C(C(C1OS(=O)(=O)[O-])O)OC1OC(COS(=-O)(=O)[O-])C(C(C1NS(=O)(=O)[O-])OS(=O)(=O)[O-])OC1OC(C(=O)[O-])C(C(C1O)O)OC1OC(COS(=O)(=O)[O-])C(C(-C1NS(=O)(=O)[O-])O)O
NCCCC1NC(=O)CNC(=O)C(NC(=O)C(NC(=O)C(NC(=O)C(Cc2c[nH]c3c2cccc3)NC(=O)C=C(c2ccc(cc2)CCCCC)C)CC(-=O)N)CC(=O)O)C(C)OC(=O)C(NC(=O)C(NC(=O)C(NC(=O)CNC(=O)C(NC(=O)C(NC(=O)C(NC1=O)CC(=O)O)C)C-C(=O)O)CO)C(CC(=O)O)C)CC(=O)c1ccccc1N
OCC1OC(Oc2cc(O)cc3c2c2cc(ccc2O)C2NC(=O)C4NC(=O)C5NC(=O)C(Cc6ccc(Oc7cc4cc(Oc4ccc(C(C(C(=O)NC3C(=O)N-CCCN(C)C)NC2=O)O)cc4Cl)c7OC2OC(C(=O)O)C(C(C2NC(=O)CCCCCCCCC(C)C)O)O)cc6)NC(=O)C(NC)c2cc(Oc3cc5-c(Cl)c(c3)O)c(O)cc2)C(C(C1O)O)O
OCC1OC(Oc2c3Oc4ccc(cc4)C(O)C(NC(=O)C(c4ccc(cc4)OC4OC(C)C(C(C4O)O)O)NC)C(=O)NC(C(=O)NC4c(c3)cc2Oc2cc-c(cc2Cl)C(OC2CC(N)C(C(O2)C)O)C2NC(=O)C(NC4=O)c3ccc(c(c3)c3c(O)cc(cc3C(NC2=O)C(=O)O)O)O)c2ccc(cc2)O)C(-C(C1O)O)OC1CC(N)C(C(O1)C)O
NCCCCC(C(=O)N)NC(=O)C(NC(=O)C(NC(=O)C(NC(=O)C(NC(=O)C(NC(=O)CNC(=O)C(Cc1ccc(cc1)O)NC(=O)C(C(-O)C)NC(=O)C(Cc1ccccc1)NC(=O)C(C(CC)C)NC(=O)C1CCCN1C(=O)C(C(C)C)NC(=O)C([N+](C)(C)C)Cc1ccccc1)CCC(-=O)O)CC(C)C)CCC(=O)N)CCCNC(=N)N)CC(C)C
CC=C1NC(=O)C(NC(=O)c2csc(n2)C23CCC(=NC3c3csc(n3)C(NC(=O)c3nc(C(NC(=O)C4N=C1SC4)C(C(O)C)(O)C)sc3)C-(C)OC(=O)c1cc(C(O)C)c3c(n1)C(O)C(C=C3)NC(C(=O)NC(C(=O)NC(=C)C(=O)NC(C(=O)N2)C)C)C(CC)C)c1scc(n1)C-(=O)NC(=C)C(=O)NC(=C)C(=O)N)C(O)C
[O-]C(=O)CCSCC1OC2OC3C(CSCCC(=O)[O-])OC(C(C3O)O)OC3C(CSCCC(=O)[O-])OC(C(C3O)O)OC3C(CSCCC(=O)[-O-])OC(C(C3O)O)OC3C(OC(OC4C(OC(OC5C(OC(OC6C(OC(OC1C(C2O)O)C(O)C6O)CSCCC(=O)[O-])C(O)C5O)CSCC-C(=O)[O-])C(O)C4O)CSCCC(=O)[O-])C(C3O)O)CSCCC(=O)[O-]
O=C(c1cc(O)c(c(c1)OC(=O)c1cc(O)c(c(c1)O)O)O)OC1C(COC(=O)c2cc(O)c(c(c2)OC(=O)c2cc(O)c(c(c2)O)O)O)OC(C(C1-OC(=O)c1cc(O)c(c(c1)OC(=O)c1cc(O)c(c(c1)O)O)O)OC(=O)c1cc(O)c(c(c1)OC(=O)c1cc(O)c(c(c1)O)O)O)OC(=O)c1cc(O)-c(c(c1)OC(=O)c1cc(O)c(c(c1)O)O)O
COc1cc2N(C)C3C4(c2cc1C1(CC2CN(CCc5c1[nH]c1c5cccc1)CC(C2)(O)CC)C(=O)OC)CCN1C4C(C(C3(O)C(=O)NNC(=O)-OCCSSCC(C(=O)O)NC(=O)C(NC(=O)C(NC(=O)C(NC(=O)C(NC(=O)CCC(C(=O)O)NC(=O)c2ccc(cc2)NCc2cnc3c(n2)c(-=O)[nH]c(n3)N)CC(=O)O)CCCNC(=N)N)CC(=O)O)CC(=O)O)O)(CC)C=CC1
OC(=O)CCC(C(=O)NC(C(=O)NC(C(=O)NC(C(=O)NC(C(=O)NC(C(=O)NC(C(=O)NC(C(=O)O)C(O)C)CCC(=O)O)Cc-1ccc(cc1)O)CSCNC(=O)C)C(O)C)CSCNC(=O)C)C(O)C)NC(=O)C(NC(=O)C(NC(=O)C(NC(=O)C(C(O)C)NC(=O)C(NC-(=O)C(Cc1c[nH]c2c1cccc2)NC(=O)C(CCC(=O)O)N)CCC(=O)N)CC(=O)O)CC(=O)N)CSCNC(=O)C
NCCCCC(C(=O)NC(C(=O)NC(C(=O)NC(C(=O)NC(C(=O)N1CCCC1C(=O)NC(C(=O)NC(C(=O)O)C(O)C)CCCNC(=N)-N)C(O)C)C(C)C)C(CC)C)CC(=O)N)NC(=O)C(NC(=O)C(NC(=O)C(NC(=O)C(C(C)C)NC(=O)C(C(C)C)NC(=O)C1CCC-N1C(=O)C(NC(=O)C(NC(=O)C(CC(=O)O)N)CCC(=O)O)CC(=O)N)Cc1cnc[nH]1)Cc1ccccc1)Cc1ccccc1
NCCCCC(C(=O)N1CCCC1C(=O)NC(C(=O)NCC(=O)NC(C(=O)NC(C(=O)NC(C(=O)NC(C(=O)N)CCCNC(=N)N)CCC-NC(=N)N)CCCCN)CCCCN)C(C)C)NC(=O)CNC(=O)C(Cc1c[nH]c2c1cccc2)NC(=O)C(NC(=O)C(NC(=O)C(NC(=O)C(NC-(=O)C(NC(=O)C(NC(=O)C(Cc1ccc(cc1)O)NC(=O)CN)CO)CCSC)CCC(=O)O)Cc1[nH]cnc1)Cc1ccccc1)CCCNC(=N)N
NCCCCC(C(=O)N1CCCC1C(=O)NC(C(=O)NCC(=O)NC(C(=O)NC(C(=O)NC(C(=O)NC(C(=O)NC(C(=O)NC(C(=O)O-)Cc1ccc(cc1)O)CCC(=O)O)C(CC)C)Cc1ccccc1)C)Cc1ccc(cc1)O)CCCNC(=N)N)NC(=O)CNC(=O)C(NC(=O)C(NC(=O)C(-NC(=O)C(NC(=O)C(NC(=O)C(C(C)C)NC(=O)C(NC(=O)C(NC(=O)C(C(CC)C)NC(=O)C(CCCNC(=N)N)N)Cc1cnc[nH]-1)CCSC)Cc1ccc(cc1)O)CO)CCCCN)CCCNC(=N)N)COP(=O)(O)O
NCCCCC(C(=O)N1CCCC1C(=O)NC(C(=O)NCC(=O)NC(C(=O)NC(C(=O)NC(C(=O)NC(C(=O)N1CCCC1C(=O)NC(C(-=O)NC(C(=O)NC(C(=O)NC(C(=O)N1CCCC1C(=O)O)Cc1ccc(cc1)O)C(C)C)CCCCN)C(C)C)CCCNC(=N)N)CCCNC(=N-)N)CCCCN)CCCCN)C(C)C)NC(=O)CNC(=O)C(Cc1c[nH]c2c1cccc2)NC(=O)C(NC(=O)C(NC(=O)C(NC(=O)C(NC(=O)C-(NC(=O)C(NC(=O)C(NC(=O)C(CO)N)Cc1ccc(cc1)O)CO)CCSC)CCC(=O)O)Cc1c[nH]cn1)Cc1ccccc1)CCCNC(=N)N
NCCCCC(C(=O)NC(C(=O)NC(C(=O)NCC(=O)NC(C(=O)NC(C(=O)NC(C(=O)NC(C(=O)NC(C(=O)NC(C(=O)NC(C(=-O)NC(C(=O)NC(C(=O)NC(C(=O)NC(C(=O)NC(C(=O)NC(C(=O)NC(C(=O)N)CCCNC(=N)N)CO)CCSC)C(CC)C)CC(=-O)O)CCC(=O)N)CC(C)C)CC(C)C)CCCCN)CCCNC(=N)N)C)CO)CC(C)C)CCC(=O)N)CC(C)C)C(C)C)NC(=O)C(NC(=-O)C(NC(=O)C(NC(=O)C(NC(=O)C(C(O)C)NC(=O)C(NC(=O)C(C(CC)C)NC(=O)C(NC(=O)C(NC(=O)C(NC(=O)C(Cc-1ccc(cc1)O)N)C)CC(=O)O)C)Cc1ccccc1)CC(=O)N)CO)Cc1ccc(cc1)O)CCCNC(=N)N
NCCCCC(C(=O)NC(C(=O)NC(C(=O)NC(C(=O)NC(C(=O)N1CCCC1C(=O)NC(C(=O)NC(C(=O)NC(C(=O)NC(C(=O)-NCC(=O)NC(C(=O)NCC(=O)NC(C(=O)N1CCCC1C(=O)N)C(O)C)CO)C(O)C)CC(=O)N)C(O)C)CCCNC(=N)N)Cc1ccc(-cc1)O)C(O)C)CCC(=O)N)CC(C)C)NC(=O)C(NC(=O)C(NC(=O)C(NC(=O)C(NC(=O)C(NC(=O)C(NC(=O)C(NC(=O)C-NC(=O)C(NC(=O)C(C(C)C)NC(=O)C1CSSCC(N)C(=O)NC(CO)C(=O)NC(C(=O)NC(C(=O)NC(C(=O)NC(C(=O)N1)C(-O)C)CO)CC(C)C)CC(=O)N)CC(C)C)CCCCN)CC(C)C)CO)CCC(=O)N)CCC(=O)O)CC(C)C)Cc1[nH]cnc1
NCCCCC(C(=O)NC1CSSCC(NC(=O)C(NC(=O)C(NC(=O)C(NC(=O)C(NC1=O)CC(=O)N)C(O)C)C)C(O)C)C(=O)NC(C-(=O)NC(C(=O)NC(C(=O)NC(C(=O)NC(C(=O)NC(C(=O)NC(C(=O)NC(C(=O)NC(C(=O)NC(C(=O)NC(C(=O)NC(C(=-O)NC(C(=O)NC(C(=O)NC(C(=O)NC(C(=O)NCC(=O)NC(C(=O)NC(C(=O)NC(C(=O)NC(C(=O)NC(C(=O)NC(C(=O)-NC(C(=O)NC(C(=O)NCC(=O)NC(C(=O)NC(C(=O)NC(C(=O)NC(C(=O)N)Cc1ccc(cc1)O)C(O)C)CC(=O)N)CO)C(C)C)-CC(=O)N)C(O)C)CO)CO)CC(C)C)C(CC)C)C)Cc1ccccc1)CC(=O)N)CC(=O)N)CO)CO)Cc1[nH]cnc1)C(C)C)CC(C)C)Cc1c-cccc1)CC(=O)N)C)CC(C)C)CCCNC(=N)N)CCC(=O)N)C(O)C)C)N
